# Coordinating activation strategy for C(*sp*^3^)–H/C(*sp*^3^)–H cross-coupling to access β-aromatic α-amino acids

**DOI:** 10.1038/ncomms9404

**Published:** 2015-09-29

**Authors:** Kaizhi Li, Qian Wu, Jingbo Lan, Jingsong You

**Affiliations:** 1 Key Laboratory of Green Chemistry and Technology of Ministry of Education, College of Chemistry, and State Key Laboratory of Biotherapy, West China Hospital, West China Medical School, Sichuan University, 29 Wangjiang Road, Chengdu 610064, PR China

## Abstract

The past decade has witnessed significant advances in C–H bond functionalizations with the discovery of new mechanisms. Non-precious transition-metal-catalysed radical oxidative coupling for C(*sp*^3^)–H bond transformations is an appealing strategy for C–C bond formations. The radical oxidative C(*sp*^3^)–H/C(*sp*^3^)–H cross-coupling reactions of α-C(*sp*^3^)–H bonds of amines with free radicals represent a conceptual and practical challenge. We herein develop the coordinating activation strategy to illustrate the nickel-catalysed radical oxidative cross-coupling between C(*sp*^3^)–H bonds and (hetero)arylmethyl free radicals. The protocol can tolerate a rich variety of α-amino acids and (hetero)arylmethanes as well as arylmethylenes and arylmethines, affording a large library of α-tertiary and α-quaternary β-aromatic α-amino acids. This process also features low-cost metal catalyst, readily handled and easily removable coordinating group, synthetic simplicity and gram-scale production, which would enable the potential for economical production at commercial scale in the future.

## Abstract (web summary)

Oxidative coupling of two C(sp3)-H groups is an attractive approach for the formation of carbon-carbon bonds, building structural complexity while avoiding the formation of waste salts. Here, the authors report such a transformation, using a nickel catalyst to functionalise amino acids with benzylic radicals.

## Graphical abstract

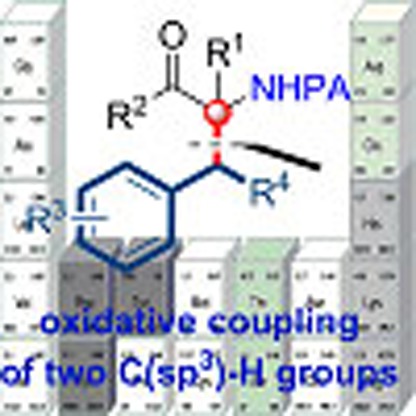

Transition-metal-catalysed functionalization of C(*sp*^3^)–H bonds is an appealing, yet challenging strategy for C(*sp*^3^)–C bond formations^1^,^2^,^3^,^4^,^5^,^6^,^7^,^8^,^9^,^10^. This attractive tactics in the rapid synthesis and late-stage diversification of organic scaffolds ranging from functional organic molecules to natural products and pharmaceutical candidates has drawn extensive attention^11^,^12^,^13^. Aromatic α-amino acids, especially β-arylalanine derivatives, are common structural units frequently found in natural products, pharmaceuticals, biologically active molecules, glycopeptides and proteins, and are also important synthetically useful compounds (Fig. 1a)^14^,^15^. Although a number of strategies have been used for the preparation of non-proteinogenic amino acids, traditional methodologies typically encounter highly hazardous reagents, tedious multiple synthesis and purification, rigorous reaction conditions such as very strong bases that may be intolerant of sensitive functionalities, and/or limited substrate scope^16^. Thus, there remains a great need for a general, diversity-orientated, economically attractive and environmentally benign protocol to prepare aromatic α-amino acids. In recent years, the chelation-directed palladium-catalysed remote methyl and methene C(*sp*^3^)–H arylation of amino-acid derivatives with aryl haildes and arylsilane reagents through C–H activation has illustrated the potential to synthesize diverse collections of non-proteinogenic β-aromatic α-amino acids (Fig. 1b, left)^17^,^18^,^19^,^20^,^21^,^22^,^23^,^24^,^25^,^26^. Despite significant progress, one of the main disadvantages of these strategies is the use of noble metal catalysts typically in combination with stoichiometric silver salts as the additive, which would diminish the appeal of practical application. Given that both α-amino acids and benzylic units^27^,^28^,^29^,^30^,^31^,^32^ are among the most widespread scaffolds, we surmised that the oxidative benzylation of α-C(*sp*^3^)–H bonds of α-amino acids with benzylic C(*sp*^3^)–H bonds would be a distinct, straightforward gateway to long-range unnatural β-arylalanine derivatives involving both α-tertiary and α-quaternary carbon centers (Fig. 1b, right).

In recent years, the oxidative C(*sp*^3^)–H/C(*sp*^3^)–H cross-coupling reactions of α-C(*sp*^3^)–H bonds of amines with a variety of nucleophilic C(*sp*^3^)–H centers typically via aza-Henry-type or Mannich-type reactions have been developed as an appealing and powerful strategy for the synthesis of amine derivatives^6^,^7^,^8^,^9^. Despite substantial progress, these reactions often exhibit a significantly narrow substrate scope and are very sensitive to steric hindrance to the approach of the imine (or imine ion) intermediate, which puts constraints on the construction of the α-quaternary center. On the other hand, the scopes of nucleophiles are typically restricted to α-C(*sp*^3^)–H of electron-withdrawing groups, organometallic reagents and C(*sp*^2^)–H of electron-rich (hetero)aromatics, in which the functionalities are not commonly found in natural products and pharmaceutical agents. Thus, it is highly desirable to develop an innovative strategy to overcome these daunting restrictions to enrich structural diversity of coupled products, albeit it is thought as one of the most challenging tasks in the organic synthesis community. In principle, it should be possible to perform the cross-coupling between α-C(*sp*^3^)–H bonds of α-amino acids and arylmethyl free radicals, easily generated from arylmethyl C(*sp*^3^)–H bond by peroxide via single-electron transfer (SET), to produce β-arylalanine derivatives (Fig. 1c, right). However, although a variety of nucleophiles have been well explored in recent years, expanding this chemistry from nucleophiles to free radicals as the coupling partner represents a conceptual and practical challenge.

The development of conceptually new strategies for the formation of new C–C or C–X bonds has been a crucial topic in synthetic organic chemistry community^33^. Recently, we disclosed the iron(III)-catalysed oxidative C(*sp*^3^)–H/C(*sp*^3^)–H cross-coupling of α-tertiary α-amino acids with various nucleophiles to deliver α-quaternary α-amino-acid derivatives by tethering the coordinating 2-pyridinecarbonyl group at the nitrogen atom of α-tertiary α-amino acids, in which the coordination of a metal center towards the coordinating group could promote the formation of a ketimine intermediate and subsequential nucleophilic attack (Fig. 1c, left)^34^. Inspired by this work, we presumed that the coordination activation of a suitable coordinating group could enable α-C(*sp*^3^)–H bond of amine substrate for directly trapping a radical species^35^ and further stabilize the resulting radical intermediate for a SET (Fig. 1c, right)^36^. In this context, activated α-amino-acid substrate could catch the in situ generated benzyl free radical to produce β-arylalanine derivative. Here we would seek to address the discovery, development, and solution to the significant challenge. In this work, using the coordinating activation strategy, we demonstrate a practical gateway to β-aromatic α-amino-acid derivatives through the nickel-catalysed radical oxidative C(*sp*^3^)–H/C(*sp*^3^)–H cross-coupling between α-amino acids and (hetero)arylmethanes as well as arylmethylenes and arylmethines.

## Results

### Optimization of the reaction conditions

To illustrate the feasibility of the coordinating activation strategy, the coordinating 2-pyridinecarbonyl group was first assembled on phenylalanine ethyl ester. The cross-coupling reaction of the resulting ethyl 3-phenyl-2-(picolinamido)propanoate **1a** with toluene **2a** was then conducted as a model reaction (Table 1). In the absence of any metal catalyst, only a trace amount of ethyl 2-benzyl-3-phenyl-2-(picolinamido)propanoate **3a** was detected (Table 1, entry 1). The use of commonly used iron, copper and ruthenium salts as the catalyst was also deeply disappointed (Table 1, entry 2). Fortunately, Ni(II) salts displayed the catalytic activity and 20 mol% of Ni(acac)_2_ gave the desired product **3a** in 83% yield in the presence of DTBP (di-*tert*-butyl peroxide, 4.0 equiv) at 140 °C under N_2_ for 18 h (Table 1, entry 7, for detailed optimization see Supplementary Table 1). Subsequently, a series of *N*-protecting groups (PGs) on phenylalanine ethyl ester were examined under the optimized conditions (Table 2). It was found that 2-quinolinecarbonyl, 1-isoquinolinecarbonyl, 2-pyrimidinecarbonyl, and 2-imidazolecarbonyl groups were also effective auxiliaries to promote the coupling reaction, but resulted in diminished yields. Other *N*-protecting groups such as benzoyl, 2-benzo[*d*]thiazolecarbonyl, 3-pyridinecarbonyl, 4-pyridinecarbonyl, phenyl and acetyl were incapable of promoting the coupling.

### Scope of benzylic substrates

With the optimal system in hand, we examined the scope of benzylic substrates with ethyl 3-phenyl-2-(picolinamido)propanoate **1a** as illustrated in Table 3. To our delight, the current catalytic system was suitable for a wide range of arylmethanes (Table 3, **3a**–**3k)**. No matter whether the benzene ring of toluene is substituted with either electron-donating, electron-withdrawing or sterically hindered group, all of them delivered the desired products in synthetically useful yields. This protocol was tolerant of synthetically valuable functional groups on the phenyl moiety (for example, chloro, bromo, alkoxy and phenoxy groups), which could allow an opportunity for further transformations. The oxidative process also occurred well with a heteroarylmethane coupling partner, affording the targeted compound **3l** in 61% yield. In addition, both 2-methylnaphthalene and 1-methylnaphthalene smoothly underwent the coupling process to yield the desired products **3m** and **3n**. In addition to (hetero)arylmethanes, we were also pleased to find that arylmethylene groups could also smoothly afford β,β-disubstituted β′-aryl α-amino-acid derivatives (Table 3, **3o**–**3t**). For example, the oxdative cross-coupling reaction of diarylmethylenes with **1a** afforded β,β′-diaryl α-amino acids **3o**–**3p**. Ethylbenzene, butylbenzene and 1,2,3,4-tetrahydronaphthalene formed the coupled products in good yields with *dr* values of ∼1:1 (Table 3, **3q**–**3s**). Gratifyingly, (methoxymethyl)benzene could also couple with **1a** to afford β-methoxyl β,β′-diaryl α-amino-acid derivative **3t**. Besides (hetero)arylmethanes and arylmethylenes, arylmethine groups could couple with **1a** and exhibited good compatibility (Table 3, **3u**–**3v**). α-Amino-acid amide could also smoothly react with toluene to afford the desired product (Table 3, **3w**). More importantly, the synthesis of α-quaternary β,β′-diaryl α-amino acid **3a** was conducted without problems on an ∼1.5-g scale in the presence of a lower catalyst loading (10 mol %; 86% yield on 1 mmol scale; 78% yield on 5-mmol scale; for details, see the Supplementary Information), which would enable the potential for economical production at commercial scale.

### Scope of α-tertiary α-amino-acid substrates

We next turned our attention to the scope of α-amino-acid substrates (Table 4). To our surprise, the widespread natural and non-proteinogenic α-amino acids did smoothly couple with toluene to produce a greatly widened range of α-quaternary β-aromatic α-amino acids, which clearly exhibited the power of coordinating activation strategy. For example, the reactions of α-alkyl-substituted α-amino-acid substrates (for example, alanine, valine, tertiary leucine, leucine and isoleucine) with toluene afforded the coupled products in good yields (Table 4, **4b**–**4f**). It is worth noting that a variety of α-amino-acid derivatives with other reactive functionalities (for example, aspartic acid, glutamic acid, methionine, benzoylglycine, tyrosine, tryptophane, lysine and serine derivatives) could also react with toluene in satisfactory yields (Table 4, **4g**–**4p**). Moreover, the phenylalanine substrates with either an electron-donating or electron-withdrawing group on the phenyl ring gave the targeted compounds in synthetically useful yields (Table 4, **4l**, and **4p**–**4r**). Notably, the proline substrate failed to produce the desired product under the optimal reaction conditions (Table 4, **4s**).

### Synthesis of α-tertiary β-aromatic α-amino-acid derivatives

Finally, we examined the feasibility of using glycine as a substrate. This simplest amino acid could react with a variety of (hetero)arylmethanes at slightly elevated temperature. The coordinating activation strategy could offer a distinct gateway to obtain a wide range of natural and unnatural α-tertiary β-arylalanine derivatives (Table 5).

### Deprotection of the coordinating group

**Figure 2:**
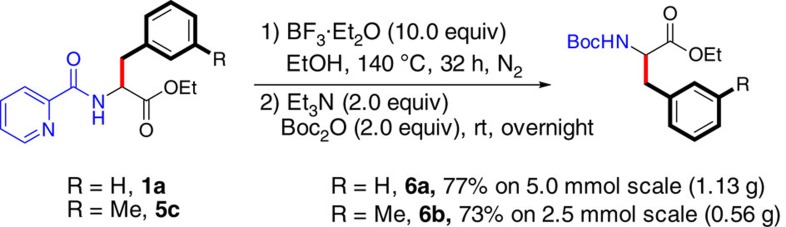
Removal of the coordinating group. Deprotection of the picolinamido group by treatment with BF_3_·Et_2_O.

The coordinating group could be easily removed in good yields by treatment with BF_3_·Et_2_O in enthanol at 140 °C for 32 h (Fig. 2). Thus, our protocol has provided an efficient, practical and reliable strategy for the synthesis of various natural and unnatural α-tertiary and α-quaternary β-aromatic α-amino acids.

## Discussion

**Figure 3:**
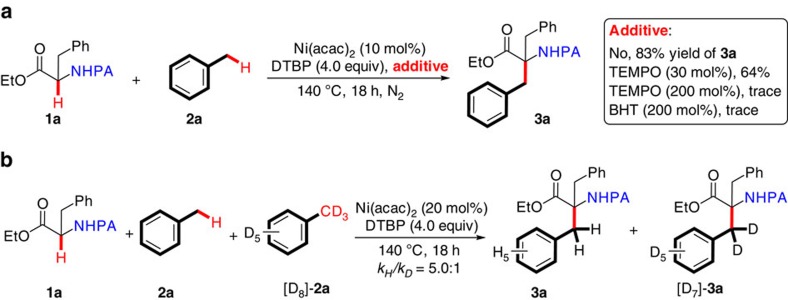
Mechanistic discussion. (**a**) The coupling reaction of **1a** and **2a** with radical trapping reagents. (**b**) The coupling reaction of **1a** with **2a** and [D_8_]-**2a**.

To gain insight into the possible mechanism of this oxidative cross-coupling process, the following competition experiments were performed. First, a radical trapping reagent (for example, 2,2,6,6-tetramethylpiperidine oxide, and 2,6-di-*tert*-butyl-4-methylphenol) could markedly suppress the coupling reaction of **1a** with **2a** (Fig. 3a), suggesting that a free radical intermediate was involved in this reaction. Second, kinetic isotope effects (KIE) were investigated with regard to the C(*sp*^3^)–H/D bonds for the toluene substrates. A clear KIE value of 5.0 was observed in a 1:1 mixture of toluene **2a** and toluene-D_8_ ([D_8_]-**2a**) (Fig. 3b), indicating that the benzylic C(*sp*^3^)–H bond breaking might be the rate-limiting step^37^.

**Figure 4:**
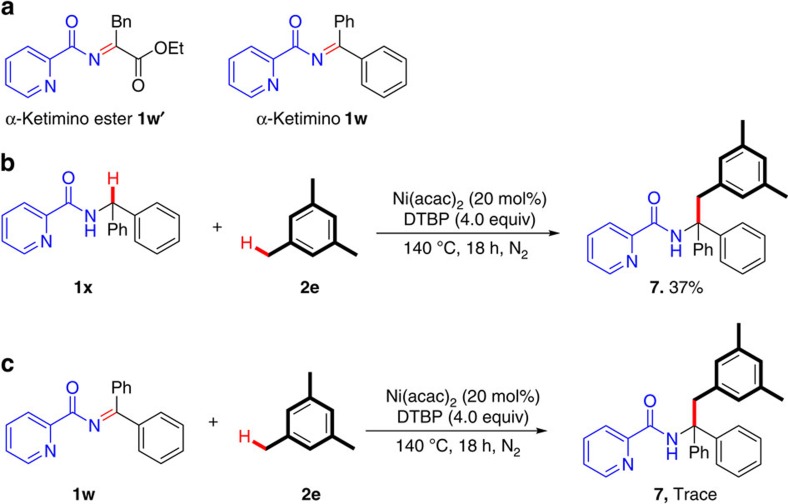
Mechanistic discussion. (**a**), Left, α-ketimine ester. Right, an alternative to α-ketimine ester. (**b**), The coupling reaction of *N*-benzhydrylpicolinamide **1x** with mesitylene **2e** under the standard reaction conditions. (**c**) The coupling reaction of *N*-(diphenylmethylene)picolinamide **1w** with mesitylene **2e** under the standard reaction conditions.

Considering that this type of oxidative C(*sp*^3^)–H/C(*sp*^3^)–H cross-coupling reactions may involve the α-ketimine ester intermediate, generated from the metal-coordinating amino-acid radical species via an intramolecular SET^34^, we attempted to synthesize α-ketimine ester **1w′**, but all efforts to isolate this compound failed (Fig. 4a). Given that the coupling reaction of *N*-benzhydrylpicolinamide **1x** with mesitylene **2e** could afford the desired product **7** in 37% yield under the standard reaction conditions (Fig. 4b), we tried to attain *N*-(diphenylmethylene)picolinamide **1w**, an alternative to α-ketimine ester. However, the reaction of **1w** with **2e** gave only a trace amount of **7** under the standard conditions (Fig. 4c). These results implied that the oxidative C(*sp*^3^)–H/C(*sp*^3^)–H cross-coupling reaction developed herein did not involve the direct formation of imine intermediate.

**Figure 5:**
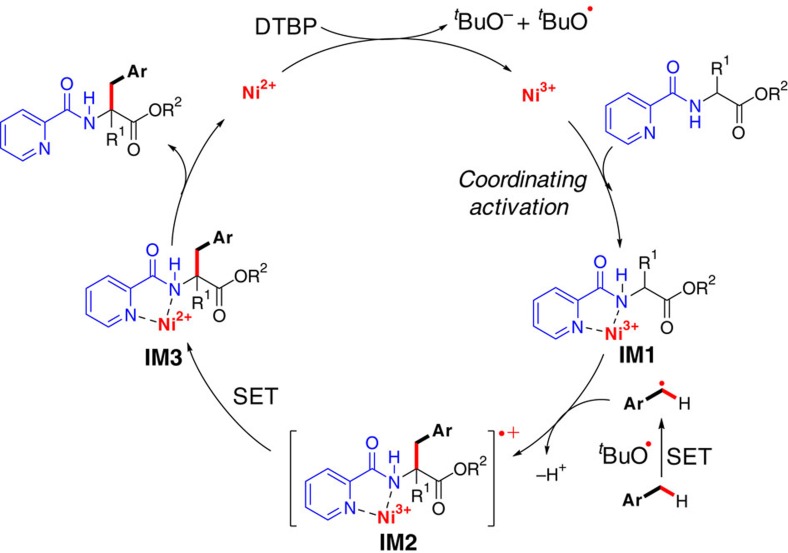
Proposed catalytic cycle. The possible mechanism involves the oxidation of the catalyst/coordinating activation/attacking α-carbon by benzylic radical/intramolecular SET.

On the basis of the above observations, the plausible catalytic path is proposed in Fig. 5. First, a *tert*-butoxy radical (*t*BuO^˙^) is generated from DTBP with the aid of the low-valent Ni^2+^ species with concomitant formation of the high-valent Ni^3+^ species^38^,^39^. Subsequently, 2-picolinamido α-amino-acid ester coordinates with the high-valent nickel catalyst to yield the intermediate **IM1**. The benzylic radical, formed through the C(*sp*^3^)–H homolytic cleavage of arylmethane by a *tert*-butoxy radical, attacks α-carbon of **IM1**. The resulting radical cation intermediate **IM2** (refs 40, 41) undergoes an intramolecular SET by the high valent Ni^3+^ to give the intermediate **IM3**. Finally, the release of the low-valent Ni^2+^ species delivers the targeted product and fulfills a catalytic cycle. The role of the coordination of metal towards the picolinamido group is assumed to involve the following aspects. First, the coordination of Ni^3+^ toward the picolinamido group can enable the activation of α-C(*sp*^3^)–H bond and makes it susceptible to an attack by the benzylic radical^35^. Second, the coordination enables to stabilize the resulting radical cation intermediate **IM2** (ref. 36). Third, an intramolecular SET process is achieved more easily.

In summary, we have developed the coordinating activation strategy to allow the radical oxidative benzylation of an α-C(*sp*^3^)–H bond of α-amino acid with a simple benzylic C(*sp*^3^)–H bond by utilizing a low-cost and easily available nickel catalyst. In previous reports, the oxidative C(*sp*^3^)–H/C(*sp*^3^)–H cross-coupling reactions of α-C(*sp*^3^)–H bonds of amines with a variety of nucleophilic C(*sp*^3^)–H centers typically undergo aza-Henry-type or Mannich-type reactions through imine (or imine ion) intermediate. In the current work, the coordination-activated α-C(*sp*^3^)–H bonds of amine substrates directly trap a benzylic radical species rather than the formation of imine intermediate. Considering that the free radical acts as a real coupling partner, the transformation reported herein has further enriched the type of the oxidative cross-coupling reactions. The protocol can tolerate a broad range of α-amino acids and (hetero)arylmethanes as well as arylmethylenes and arylmethines, which would offer a great opportunity to assemble a large library of both α-tertiary and α-quaternary β-aromatic α-amino acids. More importantly, this reaction can proceed well on a larger scale, and the coordinating group can be handled readily and removed easily. We anticipate that this process would enable the potential for economical production at commercial scale in the future.

## Methods

### General methods

For ^1^H and ^13^C nuclear magnetic resonance (NMR) spectra of compounds in this manuscript and details of the synthetic procedures, see Supplementary Figs 2–100 and Supplementary Methods.

### General procedure for the synthesis of β-arylalanines

*Reaction conditions A*. An oven-dried Schlenk tube with a magnetic stir bar was charged with amino-acid derivative **1** (0.25 mmol), arylmethane **2** (1.0 ml) and Ni(acac)_2_ (12.8 mg, 0.05 mmol) under N_2_ atmosphere. The reaction solution was stirred at room temperature for several minutes and DTBP (182.6 μl, 1.0 mmol) was then added. The tube was sealed with a teflon-coated cap and the mixture was stirred at 140 °C for 18 h. After being cooled to ambient temperature, the solution was diluted with 20 ml of CH_2_Cl_2_, filtered through a celite pad and washed with 10–20 ml of CH_2_Cl_2_. The combined organic phases were concentrated and the residue was purified by column chromatography on silica gel to provide the desired product.

*Reaction conditions B*. An oven-dried Schlenk tube with a magnetic stir bar was charged with amino-acid derivative **1** (0.25 mmol), arylmethane **2** (2.5 mmol), Ni(acac)_2_ (12.8 mg, 0.05 mmol) and benzene (0.5 ml) under an N_2_ atmosphere. The reaction solution was stirred at room temperature for several minutes and DTBP (182.6 μl, 1.0 mmol) was then added. The tube was sealed with a teflon-coated cap and the mixture was stirred at 140 °C for 18 h. After being cooled to ambient temperature, the solution was diluted with 20 ml of CH_2_Cl_2_, filtered through a celite pad, and washed with 10–20 ml of CH_2_Cl_2_. The combined organic phases were concentrated and the residue was purified by column chromatography on silica gel to provide the desired product.

## Additional information

**How to cite this article:** Li, K. *et al*. Coordinating activation strategy for C(*sp*^3^)–H/C(*sp*^3^)–H cross-coupling to access β-aromatic α-amino acids. *Nat. Commun*. 6:8404 doi: 10.1038/ncomms9404 (2015).

## Supplementary Material

Supplementary Information Supplementary Figures 1-100, Supplementary Table 1, Supplementary Methods and Supplementary References.

## Figures and Tables

**Figure 1:**
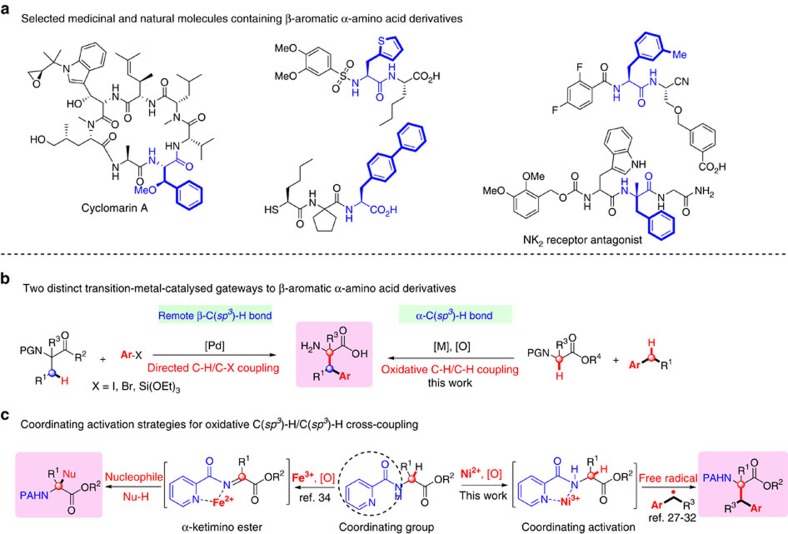
Substituted β-aromatic α-amino acids derivatives. (**a**) Selected medicinal and natural molecules containing β-aromatic α-amino-acid derivatives. (**b**), Transition-metal-catalysed two distinct gateways to β-aromatic α-amino-acid derivatives. Left, in previous work, the synthesis of β-arylalanine blocks through the palladium-catalysed chelation-directed β-C(sp^3^)–H activation strategy. Right, in the current work, the preparation of β-arylalanine blocks through oxidative C(*sp*^3^)–H/C(*sp*^3^)–H cross-coupling. (**c**), Coordinating activation strategies for oxidative C(*sp*^3^)–H/C(*sp*^3^)–H cross-coupling. Left, in our previous work, iron(III)-catalyzed oxidative C(*sp*^3^)–H/C(*sp*^3^)–H cross-coupling of α-tertiary α-amino acids with various nucleophiles to deliver α-quaternary α-amino acids derivatives. Right, in this report, the oxidative C(*sp*^3^)–H/C(*sp*^3^)–H cross-coupling of α-amino acids with a radical species through coordinating activation.

**Table 1:**
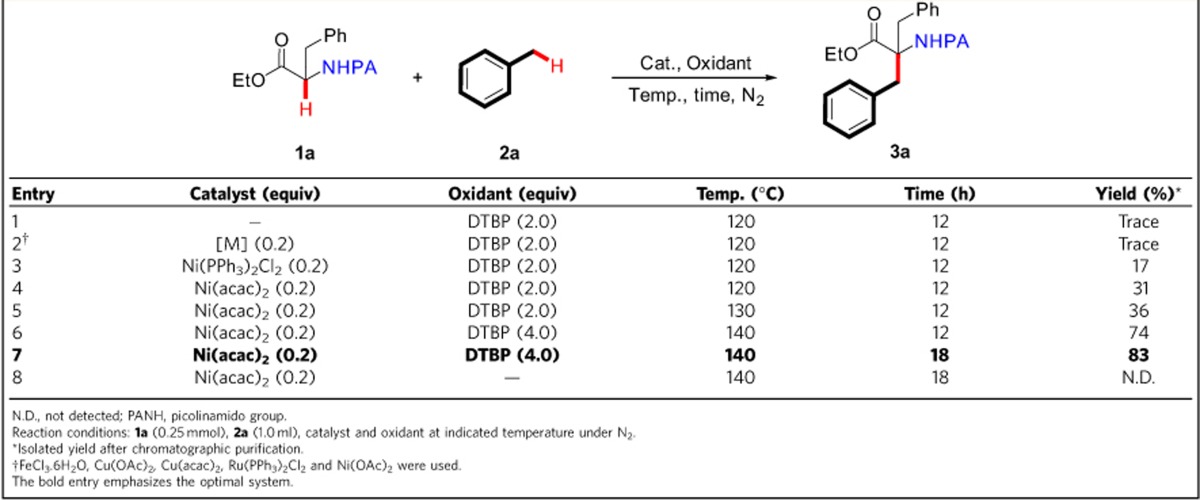
Optimization of reaction conditions.

**Table 2:**
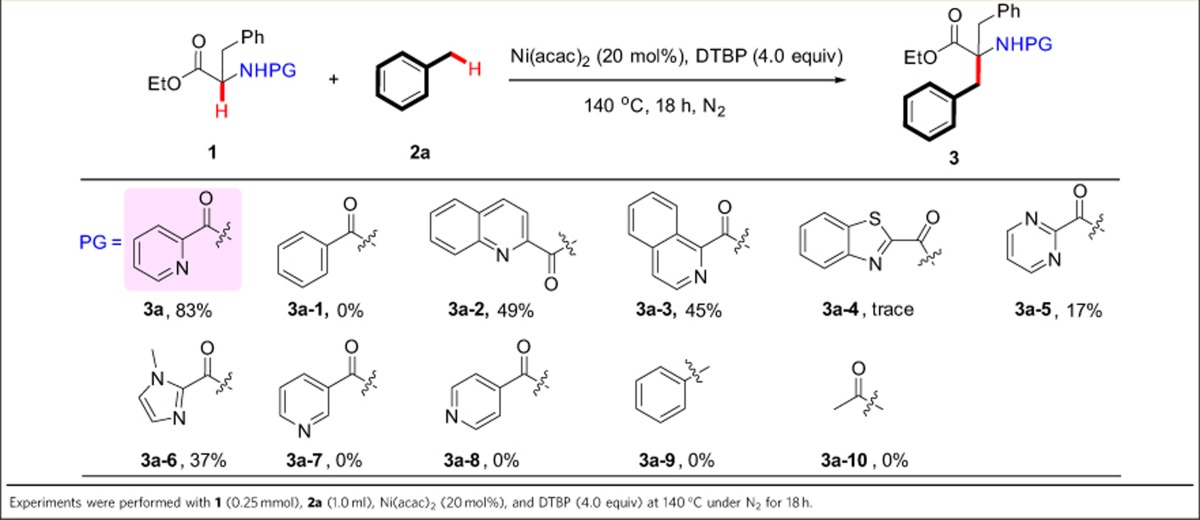
The effect of *N*-protecting groups on the oxidative cross-coupling between the N-substituted phenylalanine ethyl ester and toluene.

**Table 3:**
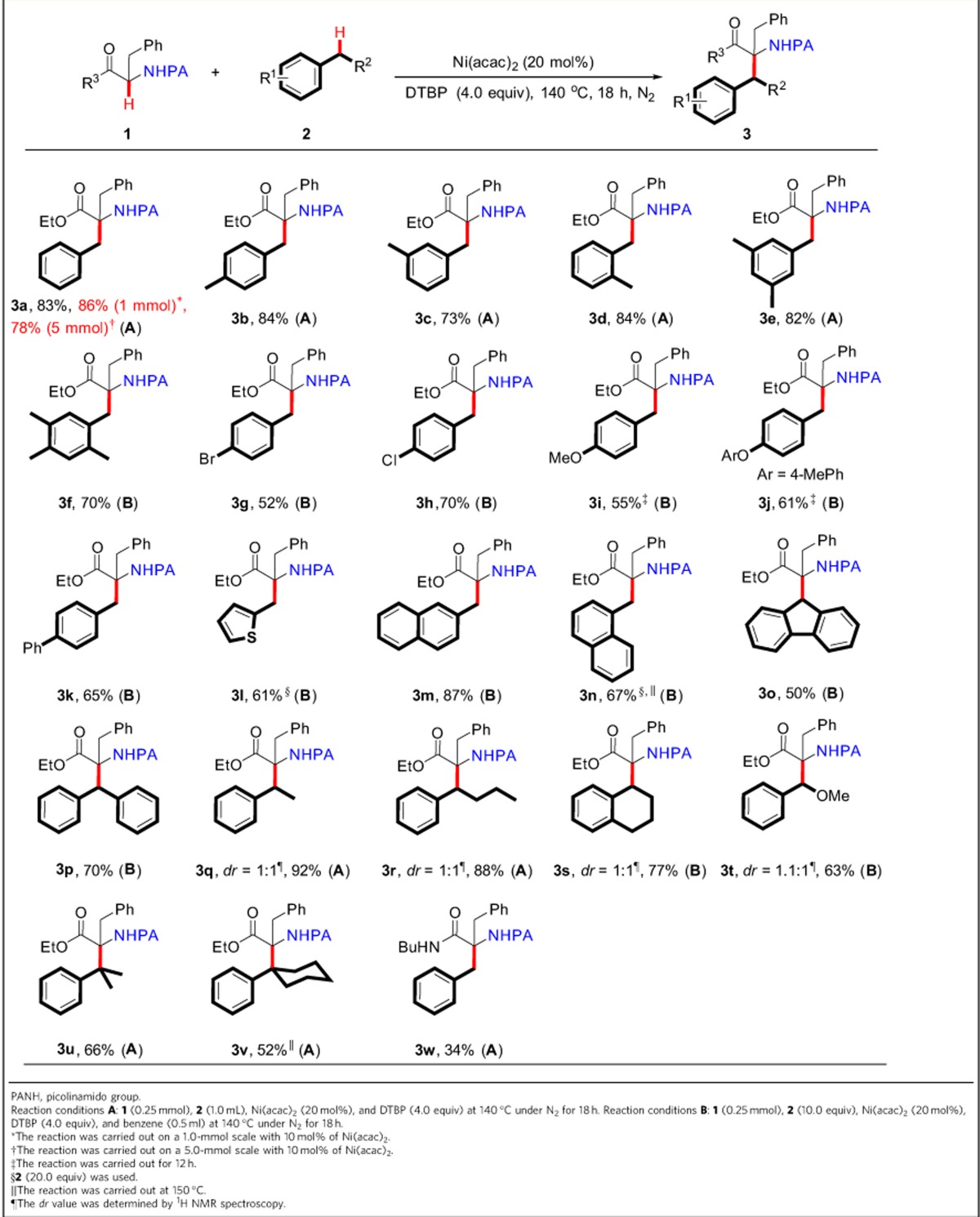
The scope of benzylic substrates.

**Table 4:**
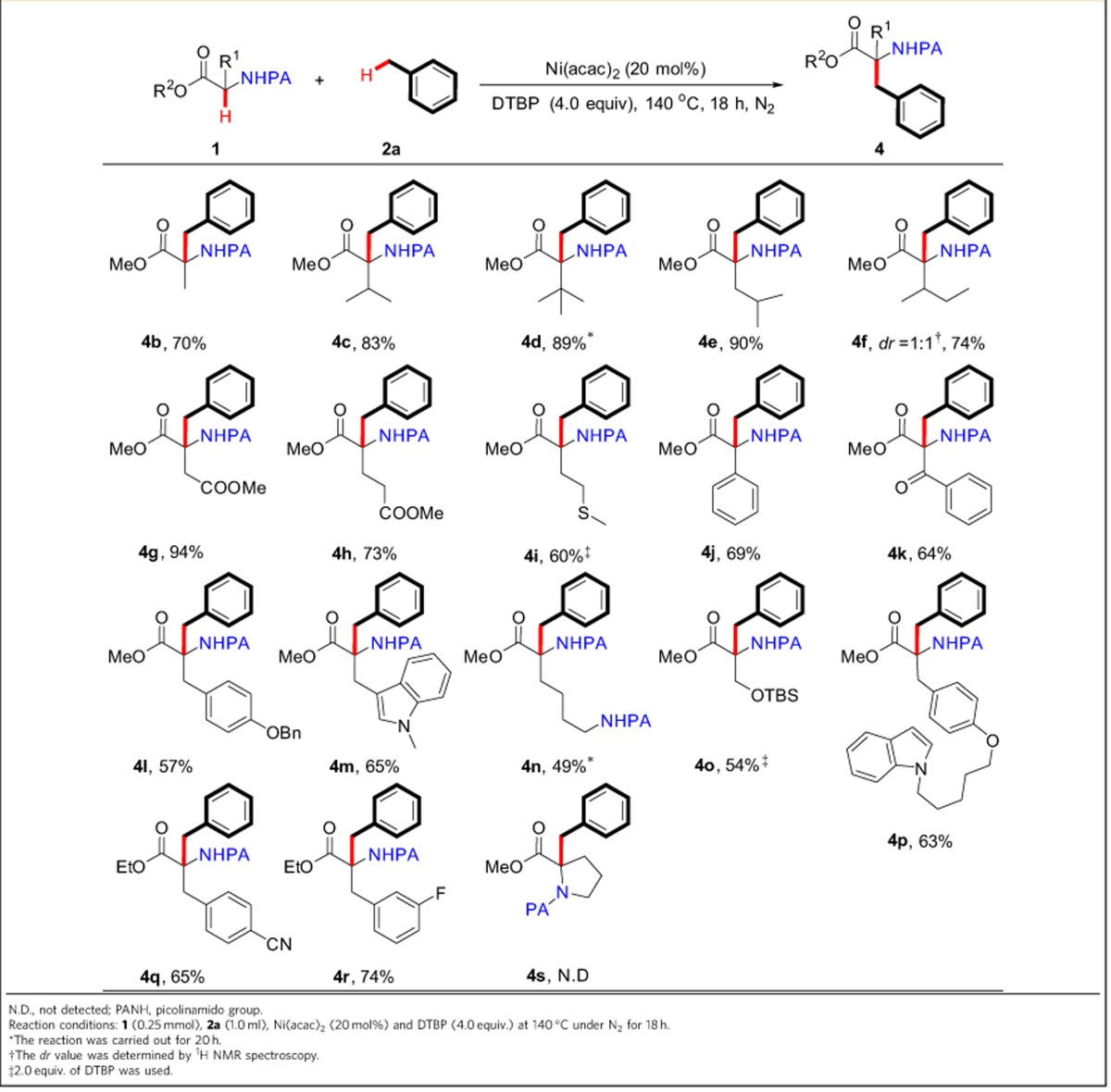
The scope of α-tertiary α-amino acid substrates.

**Table 5:**
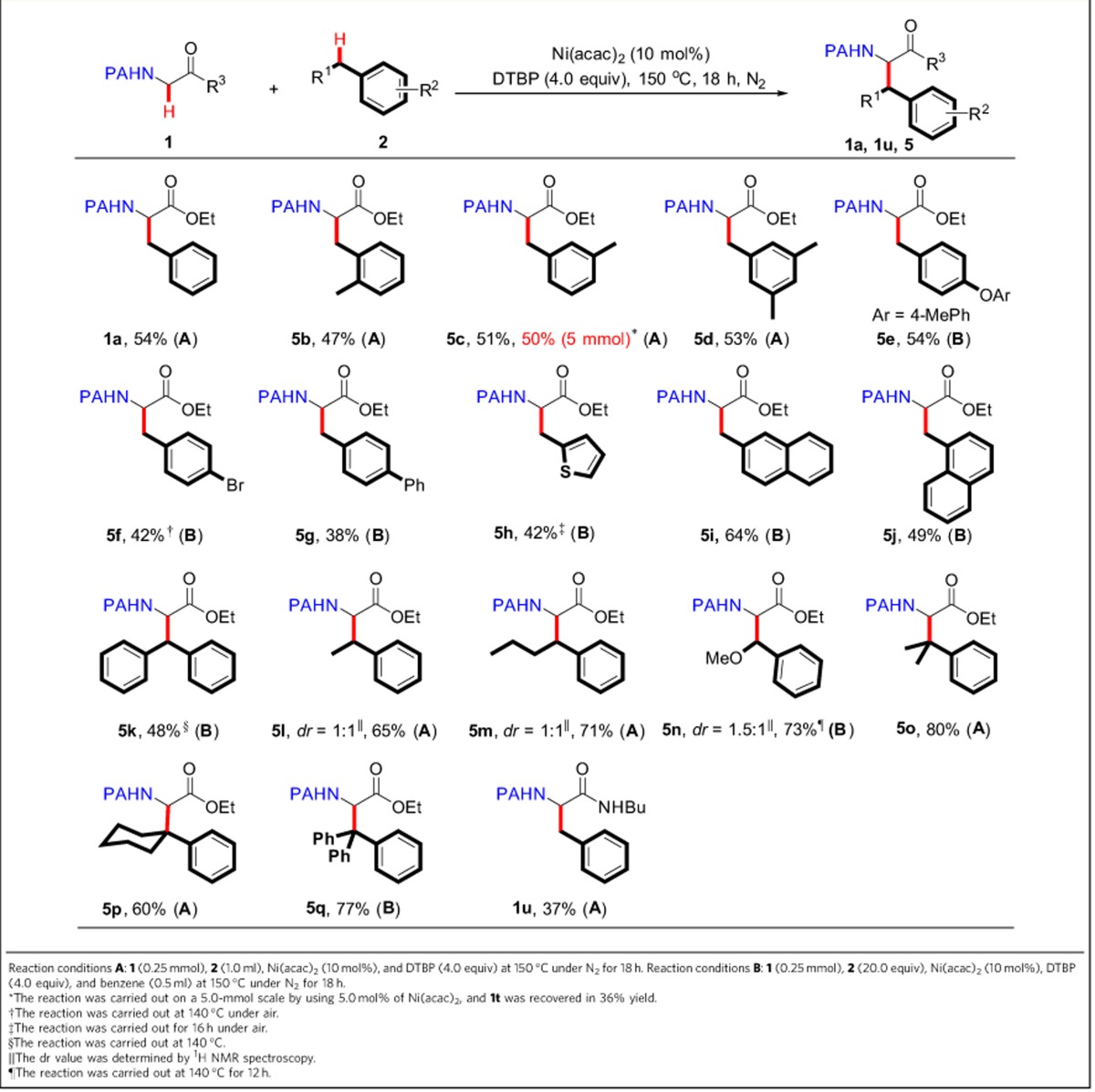
The reaction of glycine substrate with benzylic C−H bonds.
