# Supplementary material for: Coordinating activation strategy for C(sp3)–H/C(sp3)–H cross-coupling to access β-aromatic α-amino acids
Source: Nat Commun. 2015 Sep 29;6:8404. doi: 10.1038/ncomms9404 (PMC4598627; doi:10.1038/ncomms9404)
Supplement: Supplementary Information — Supplementary Figures 1-100, Supplementary Table 1, Supplementary Methods and Supplementary References. [file ncomms9404-s1.pdf]

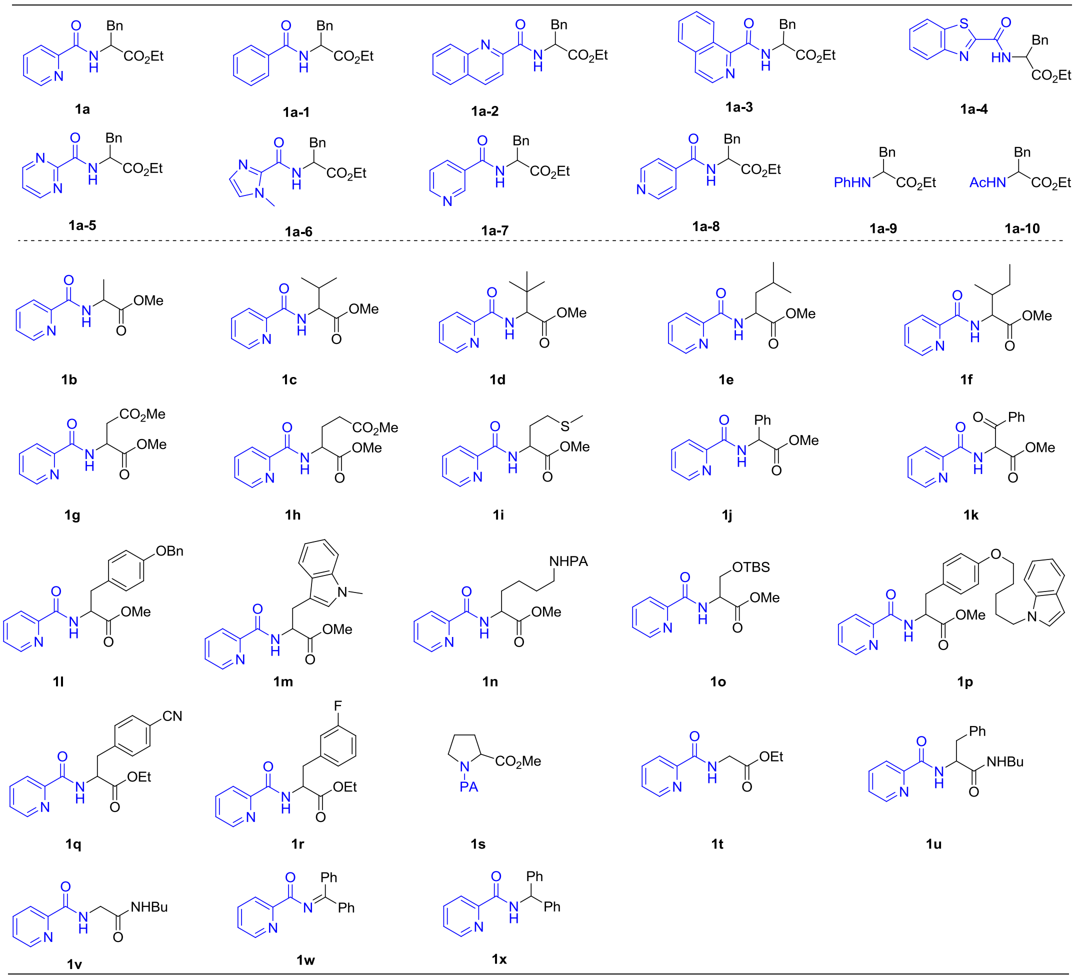

Supplementary Figure 1. Structures of starting materials

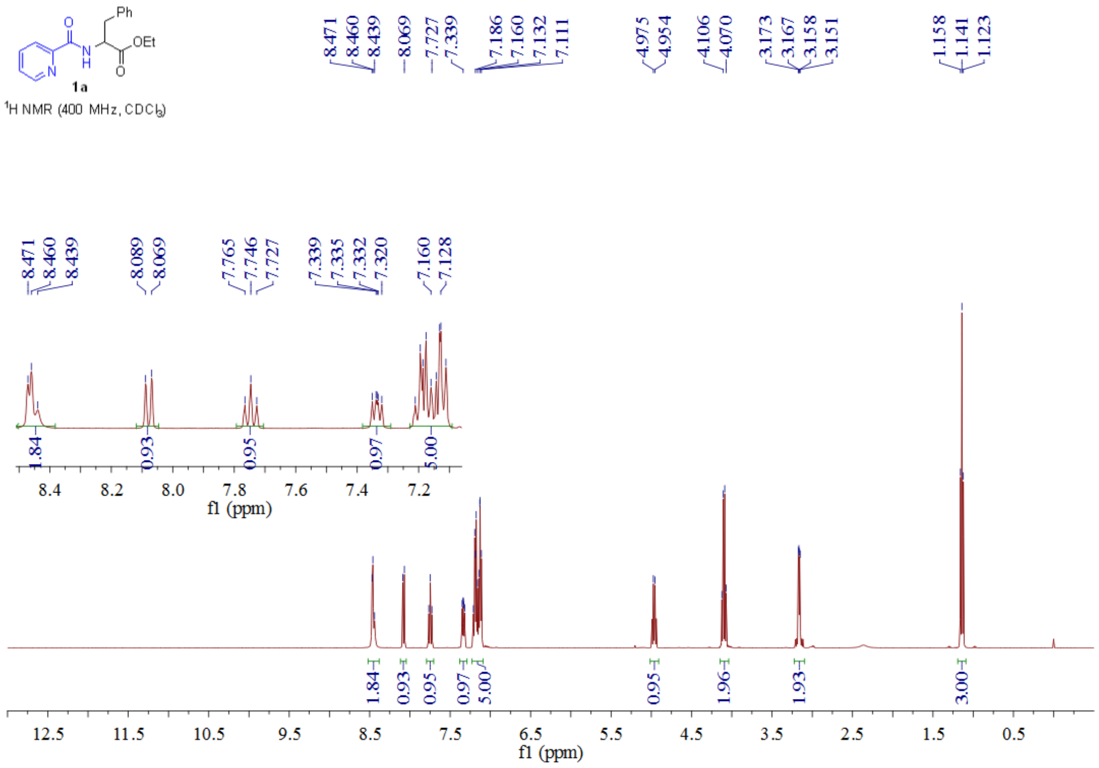

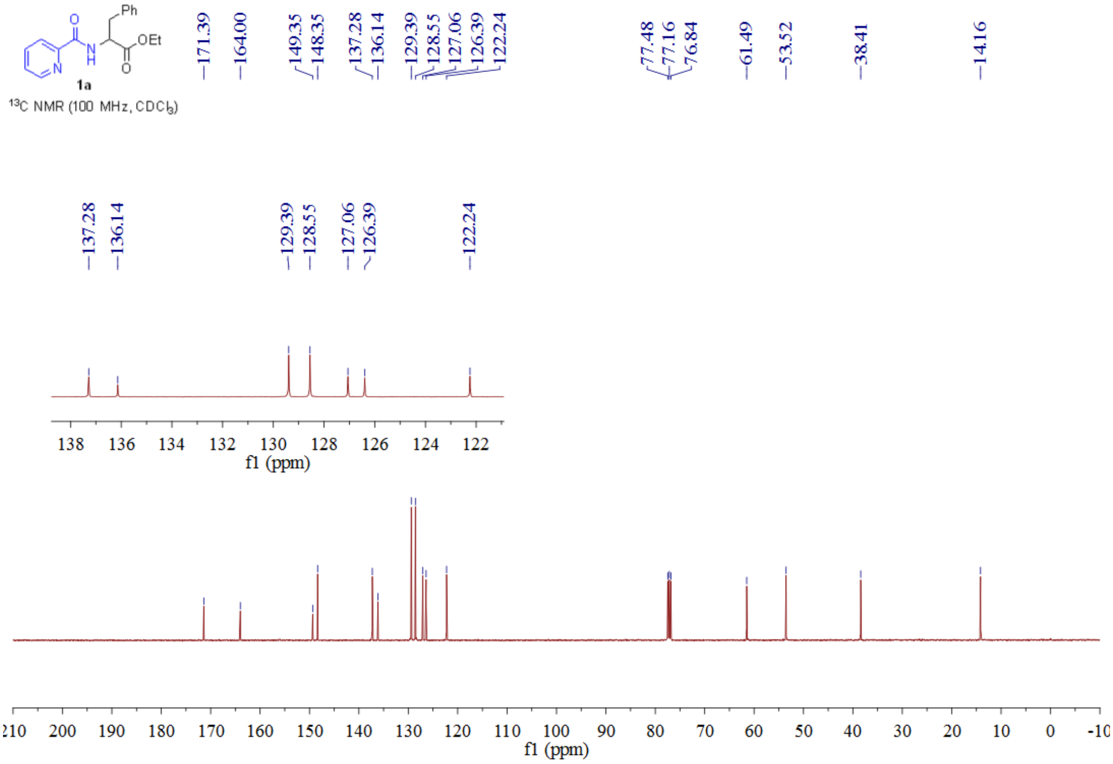

Supplementary Figure 2. ^{1}H NMR and ^{13}C NMR spectra for compound 1a

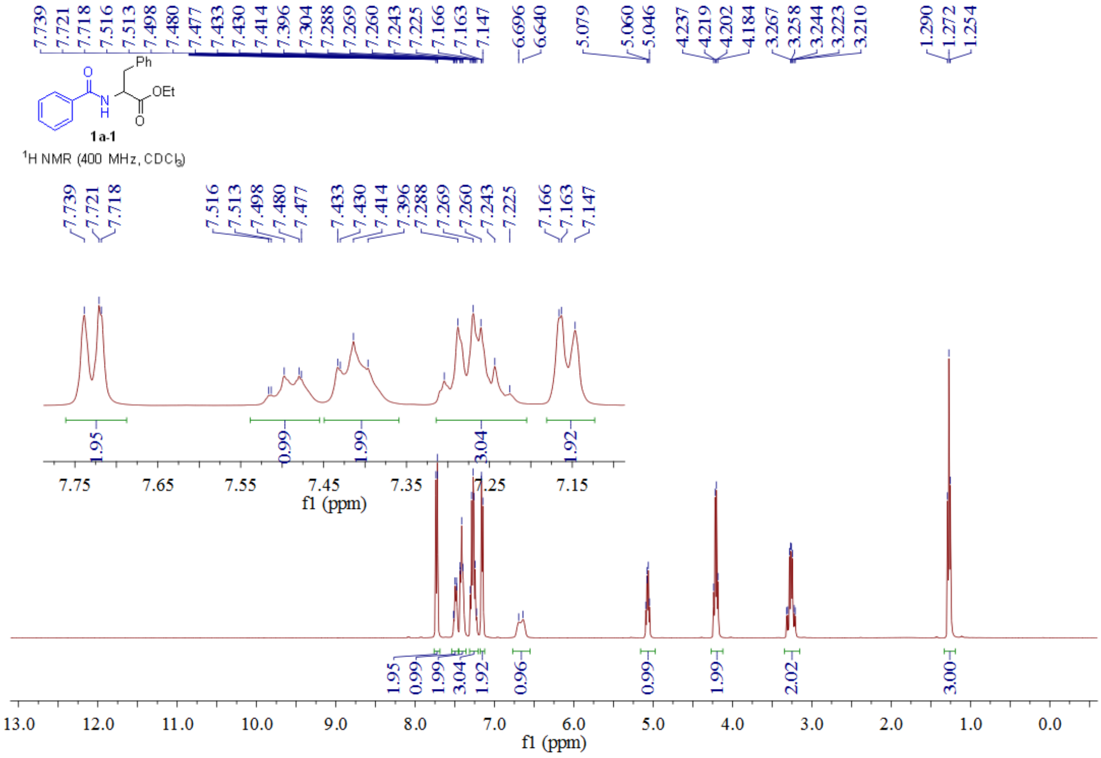

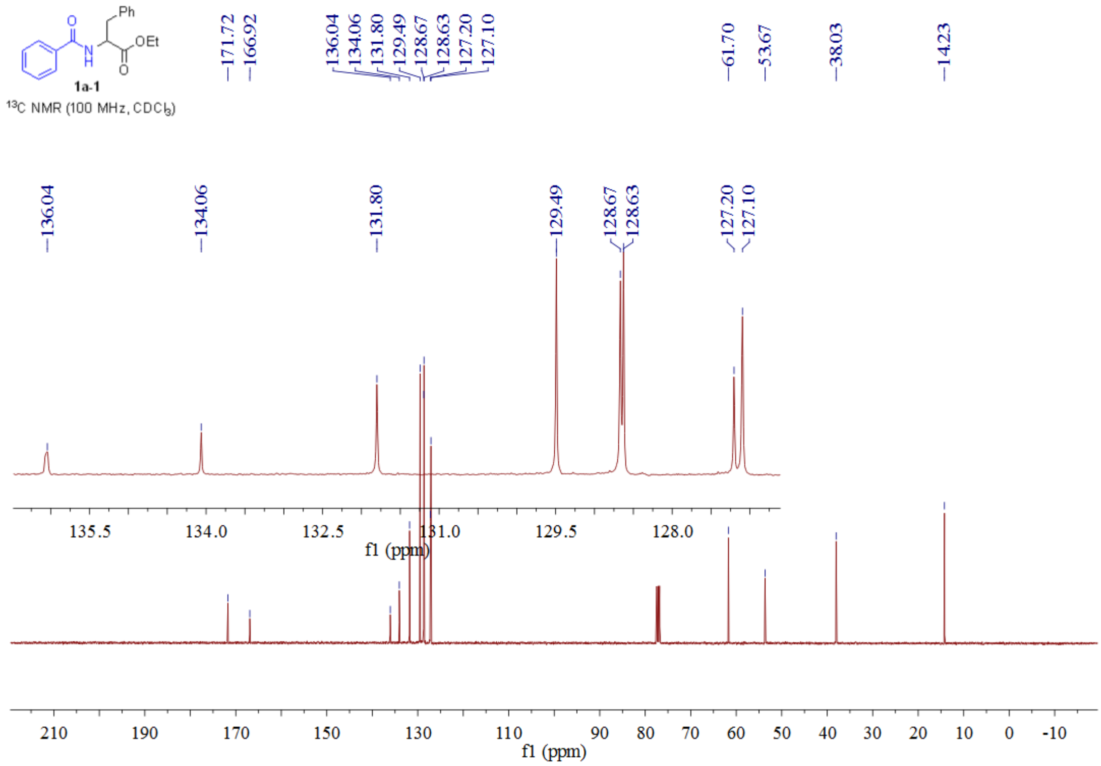

Supplementary Figure 3. ^{1}H NMR and ^{13}C NMR spectra for compound 1a-1

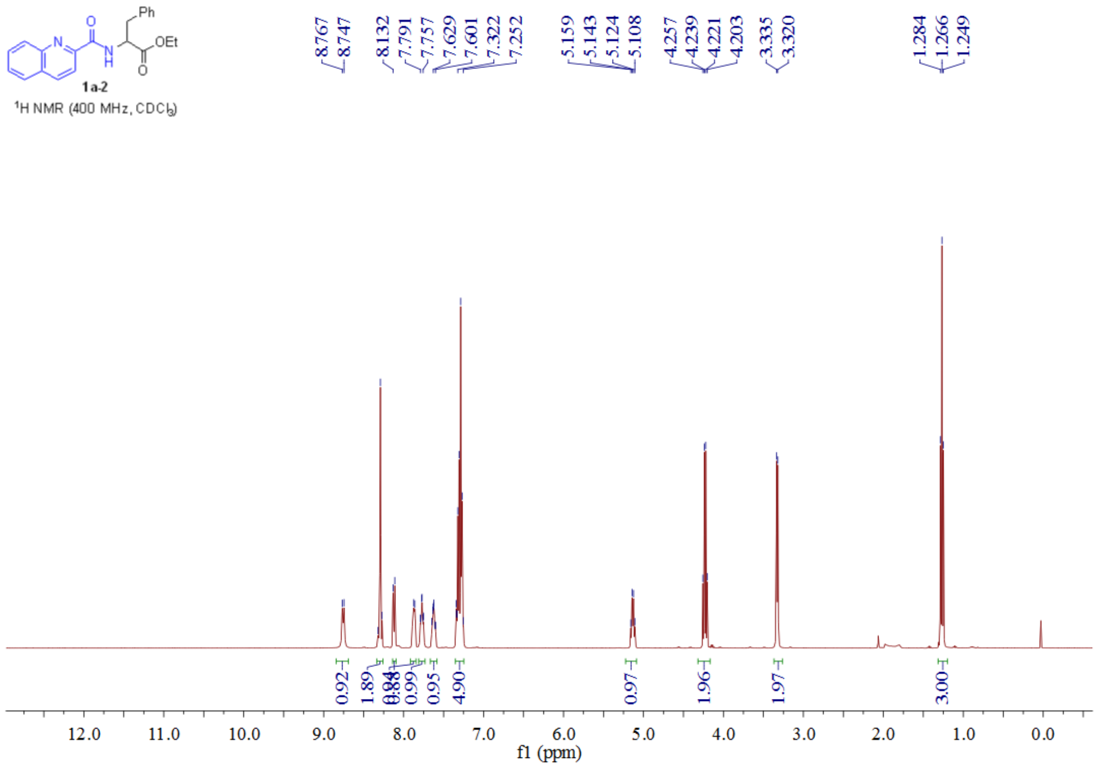

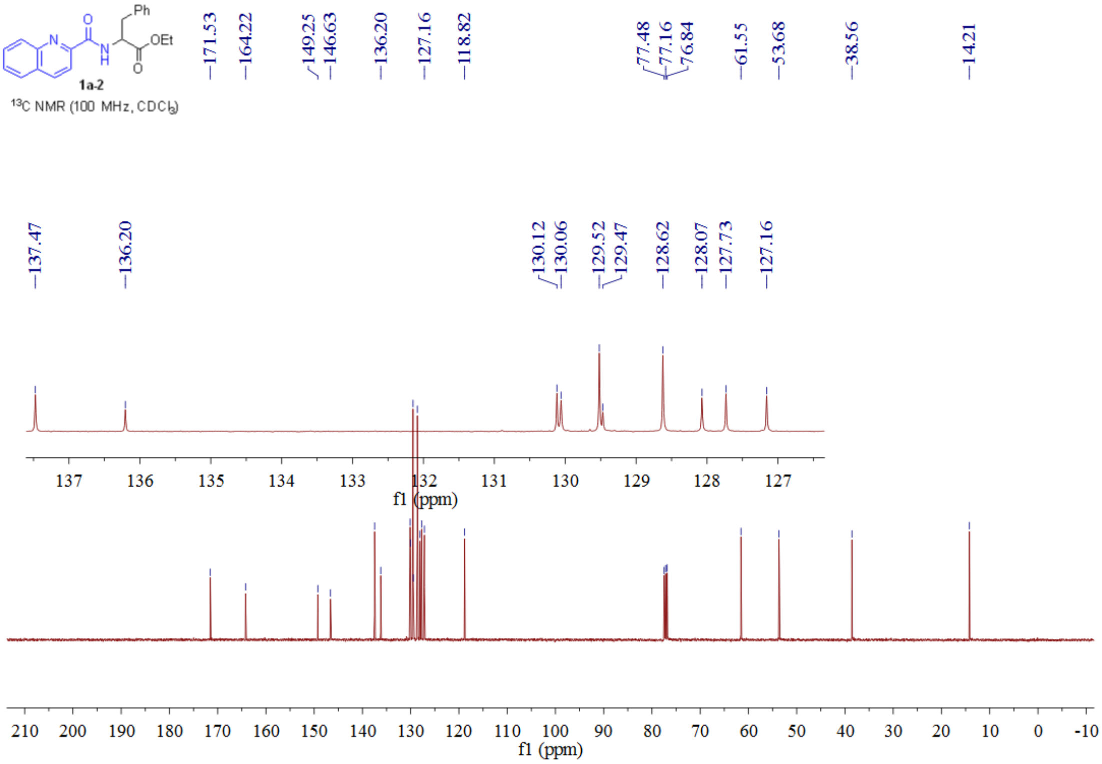

Supplementary Figure 4. ^{1}H NMR and ^{13}C NMR spectra for compound 1a-2

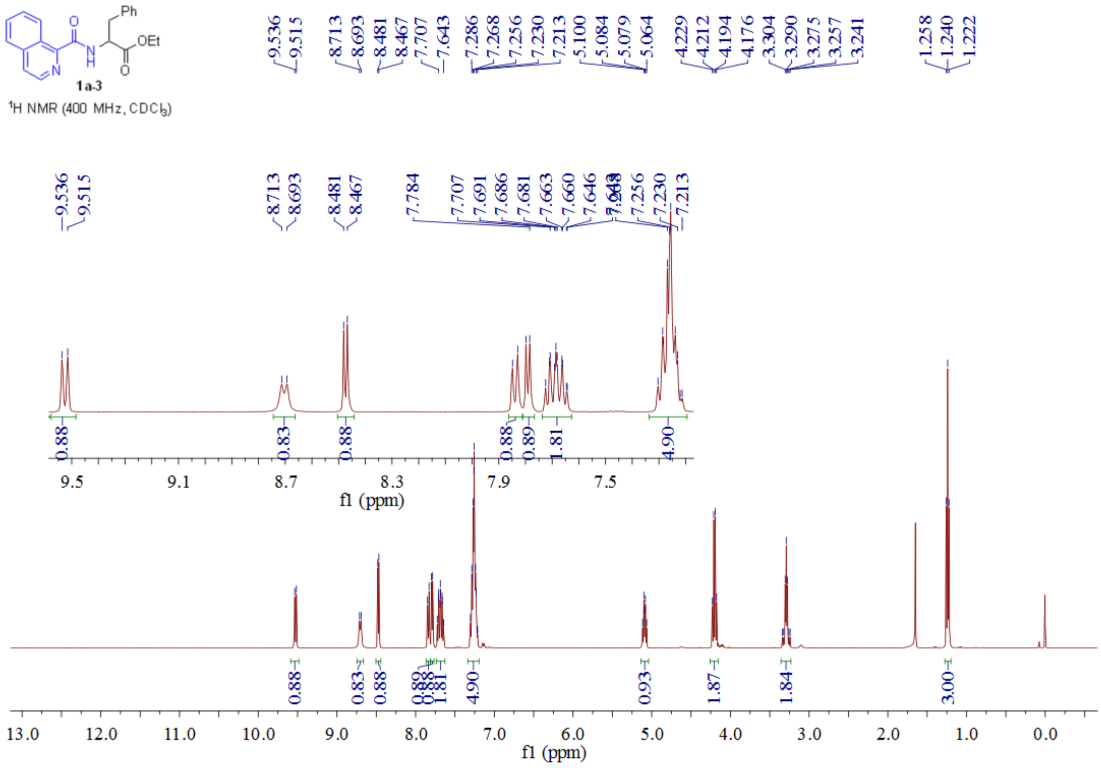

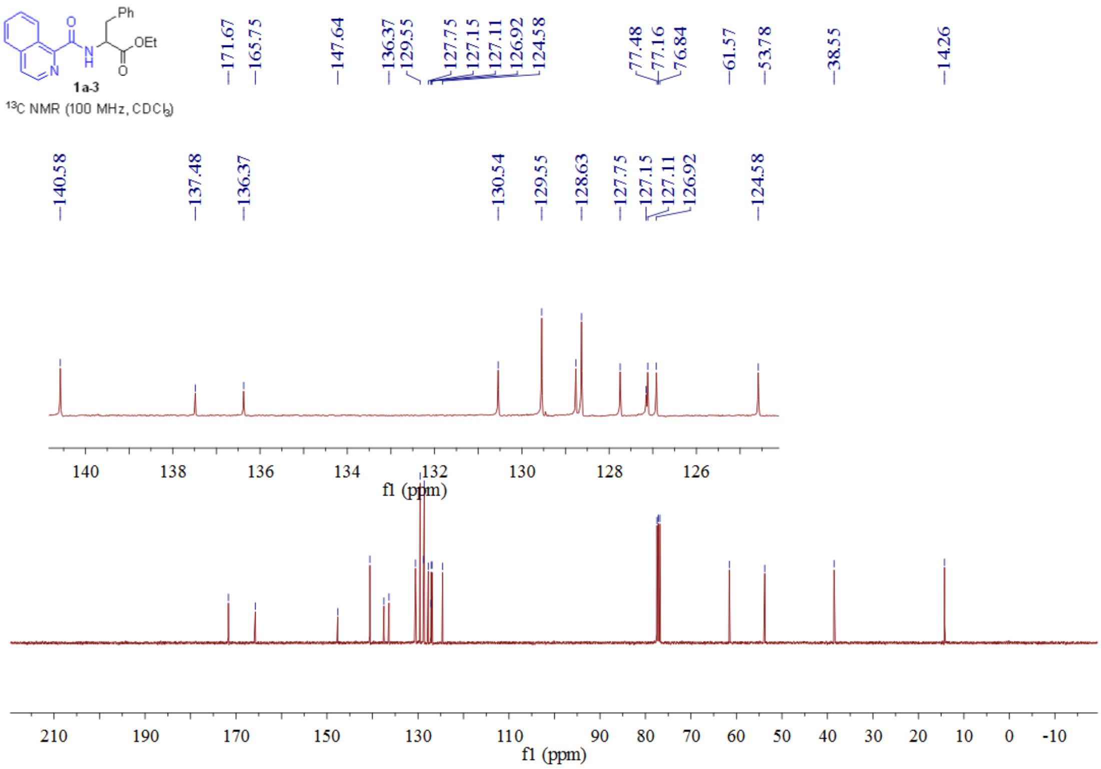

Supplementary Figure 5. ^{1}H **NMR and** ^{13}C **NMR spectra for compound 1a-3**

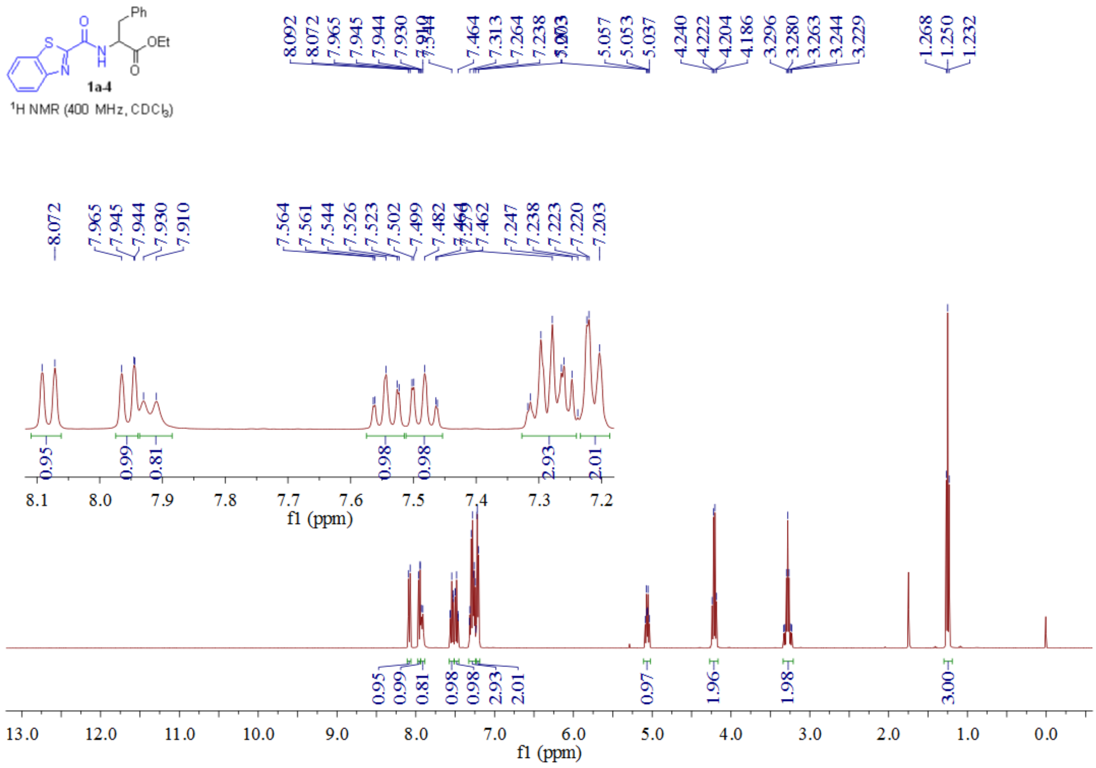

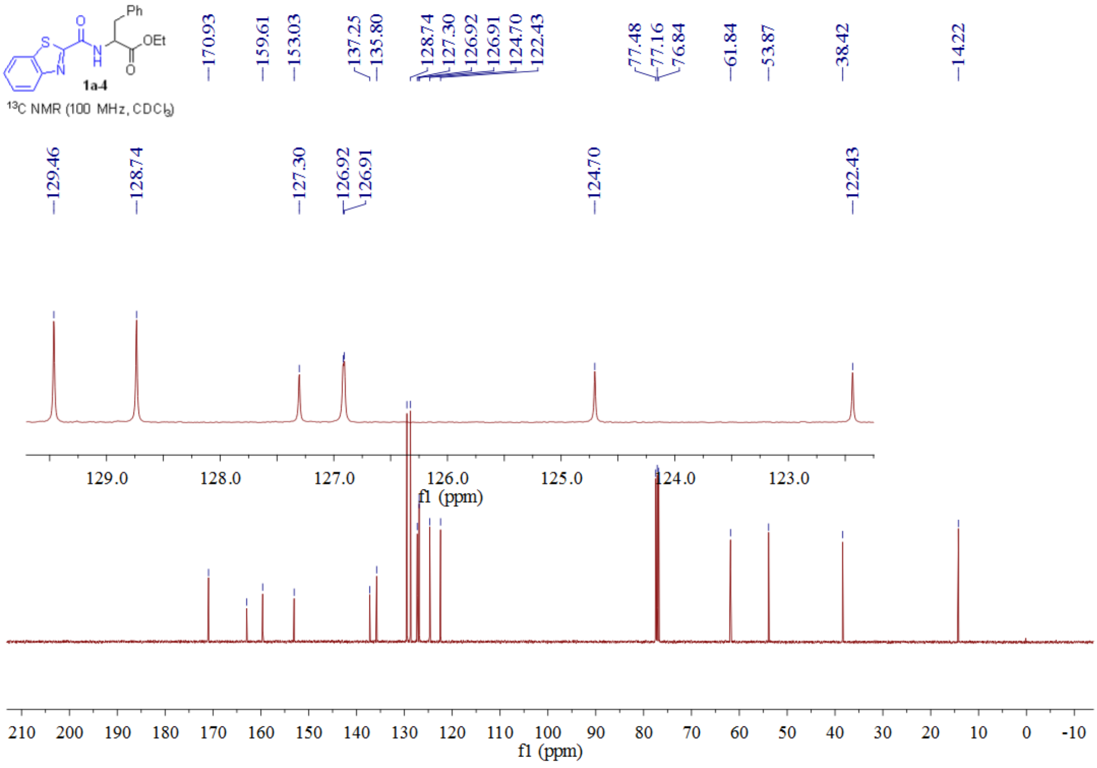

Supplementary Figure 6. ^{1}H NMR and ^{13}C NMR spectra for compound 1a-4

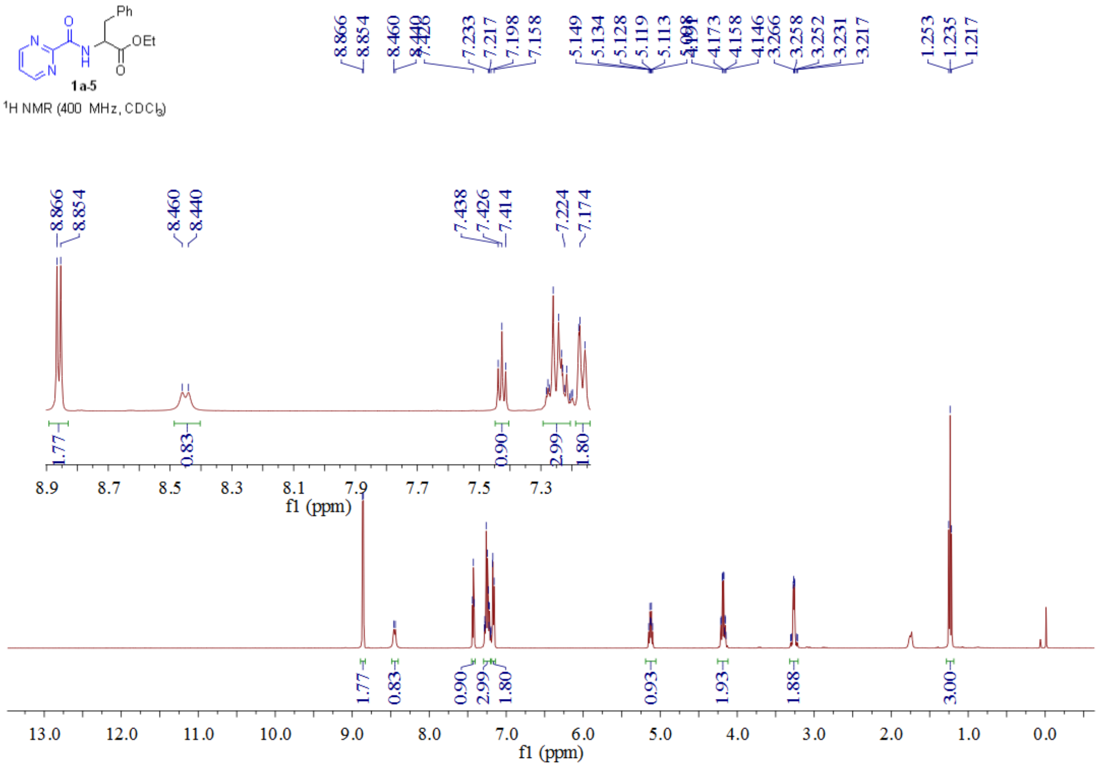

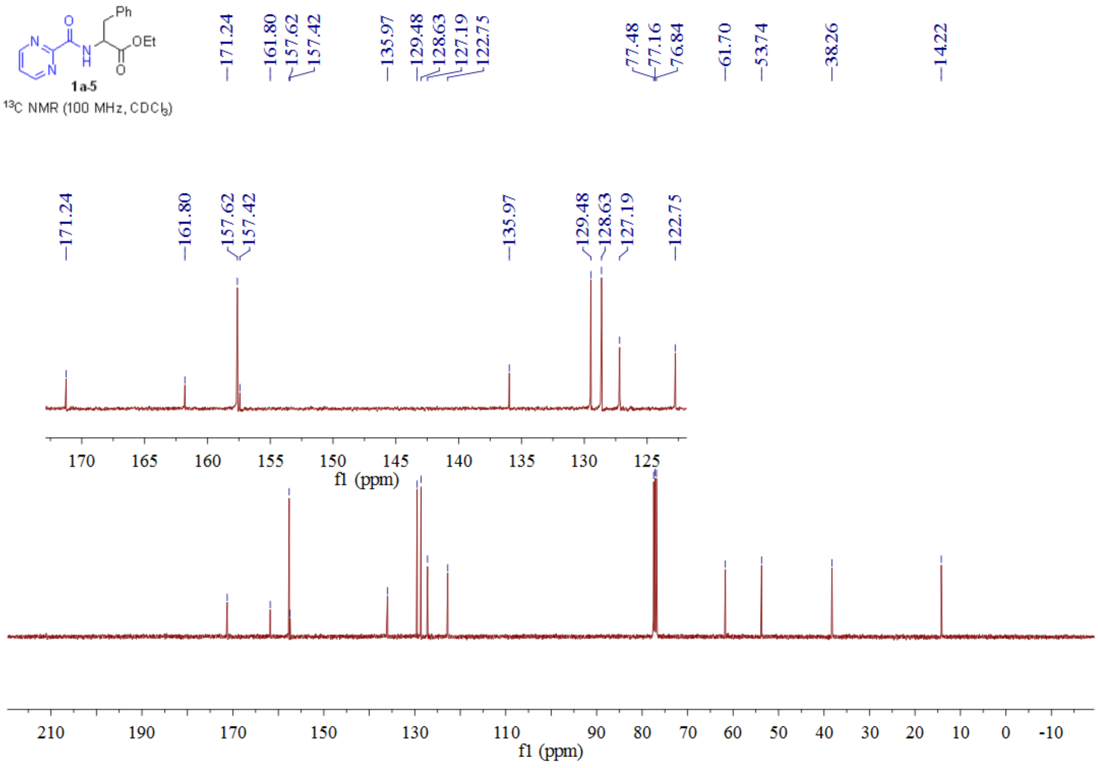

Supplementary Figure 7. ^{1}H NMR and ^{13}C NMR spectra for compound 1a-5

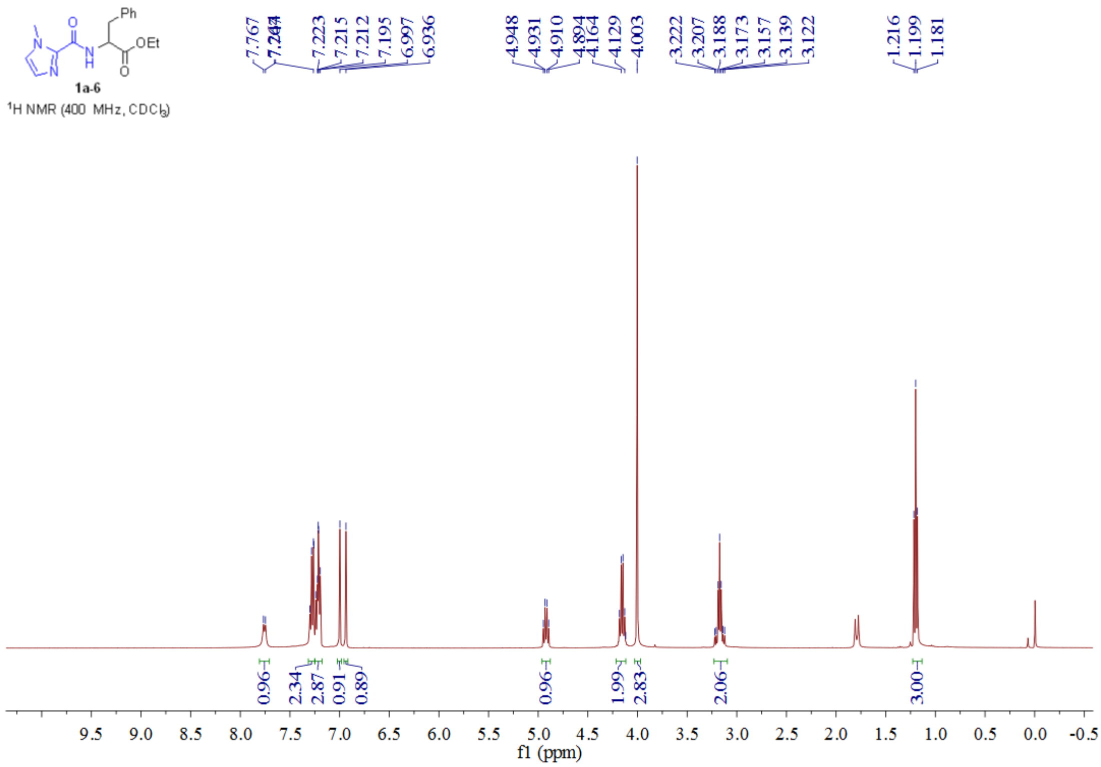

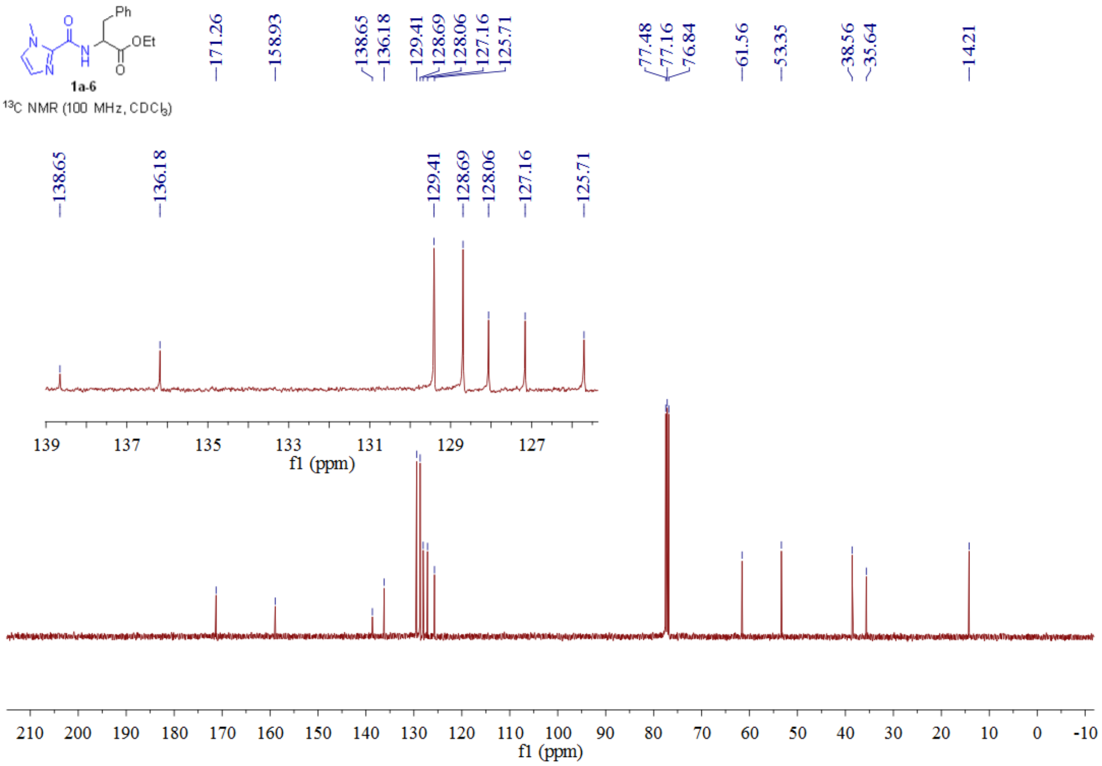

Supplementary Figure 8. ^{1}H NMR and ^{13}C NMR spectra for compound 1a-6

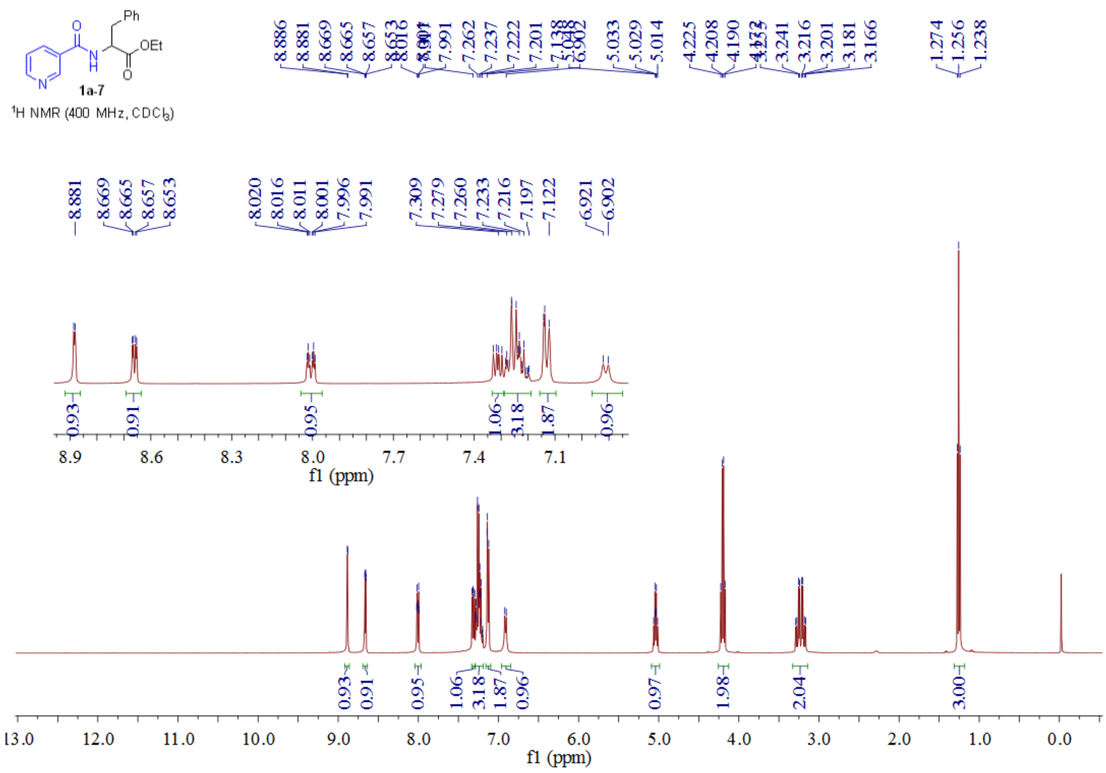

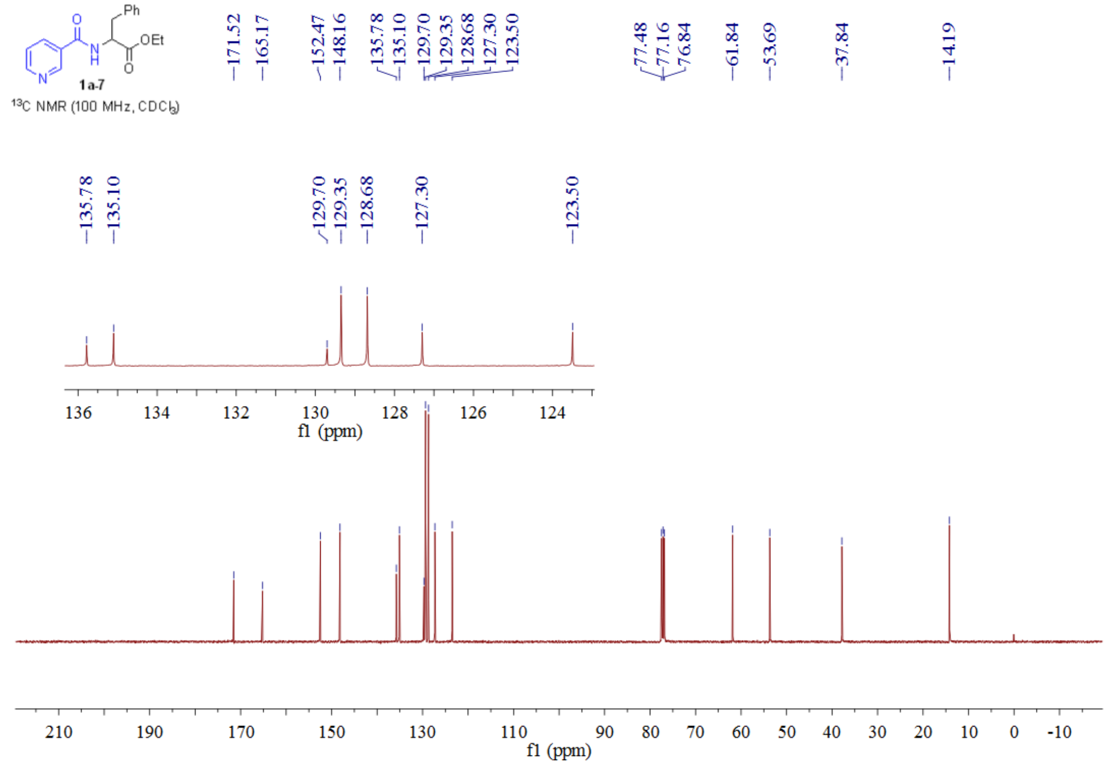

Supplementary Figure 9. ^{1}H NMR and ^{13}C NMR spectra for compound 1a-7

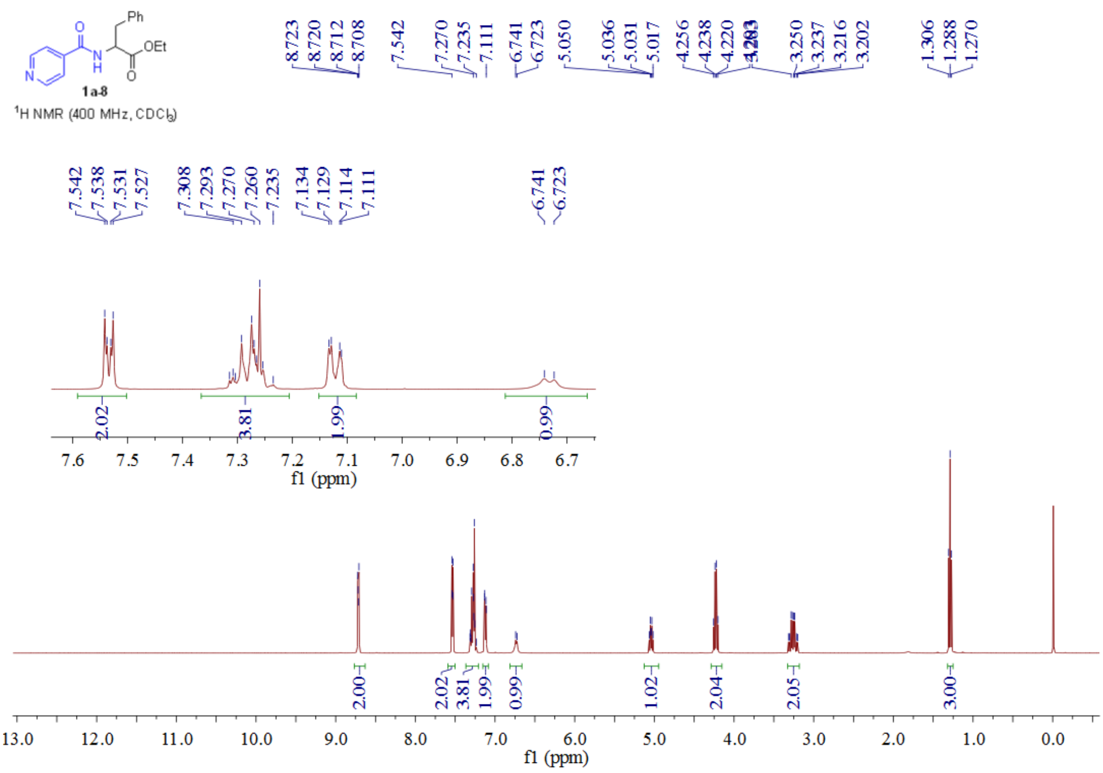

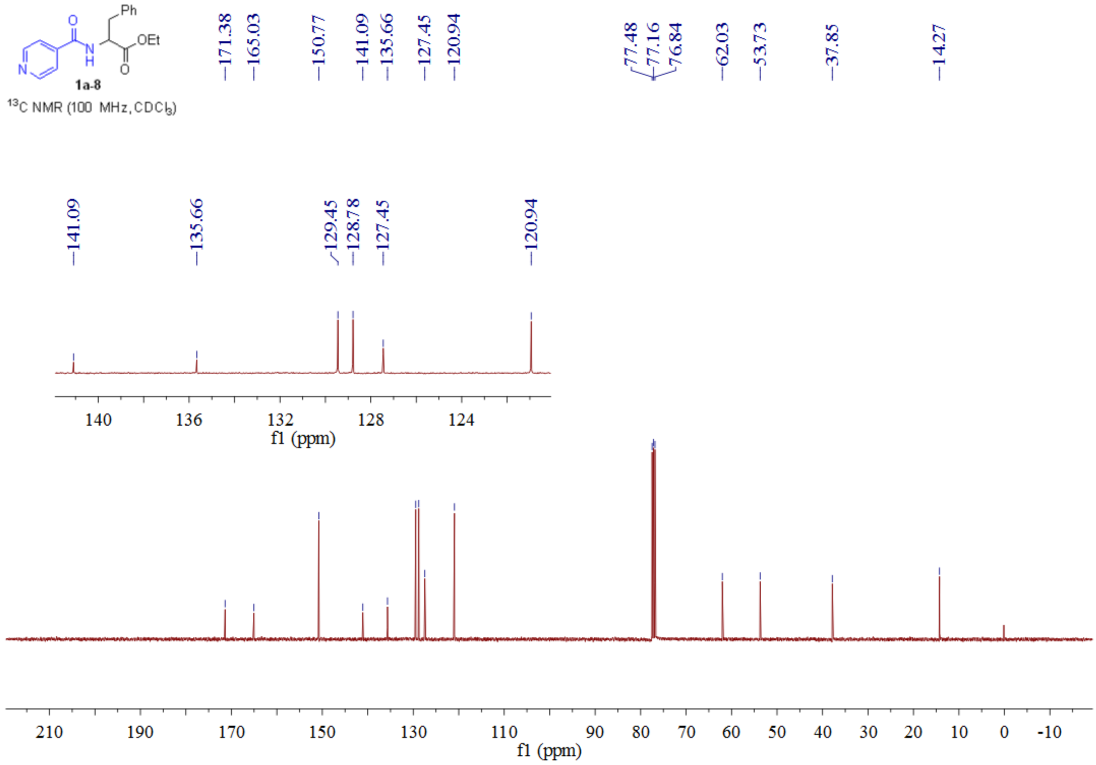

Supplementary Figure 10. ^{1}H NMR and ^{13}C NMR spectra for compound 1a-8

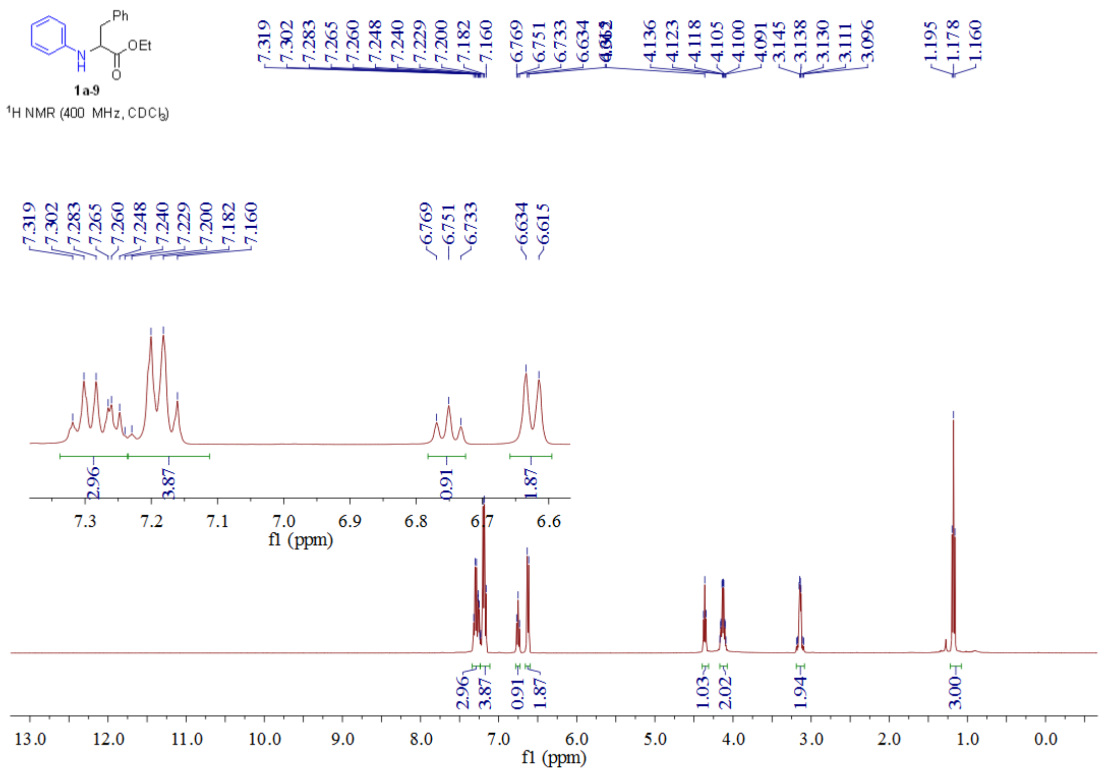

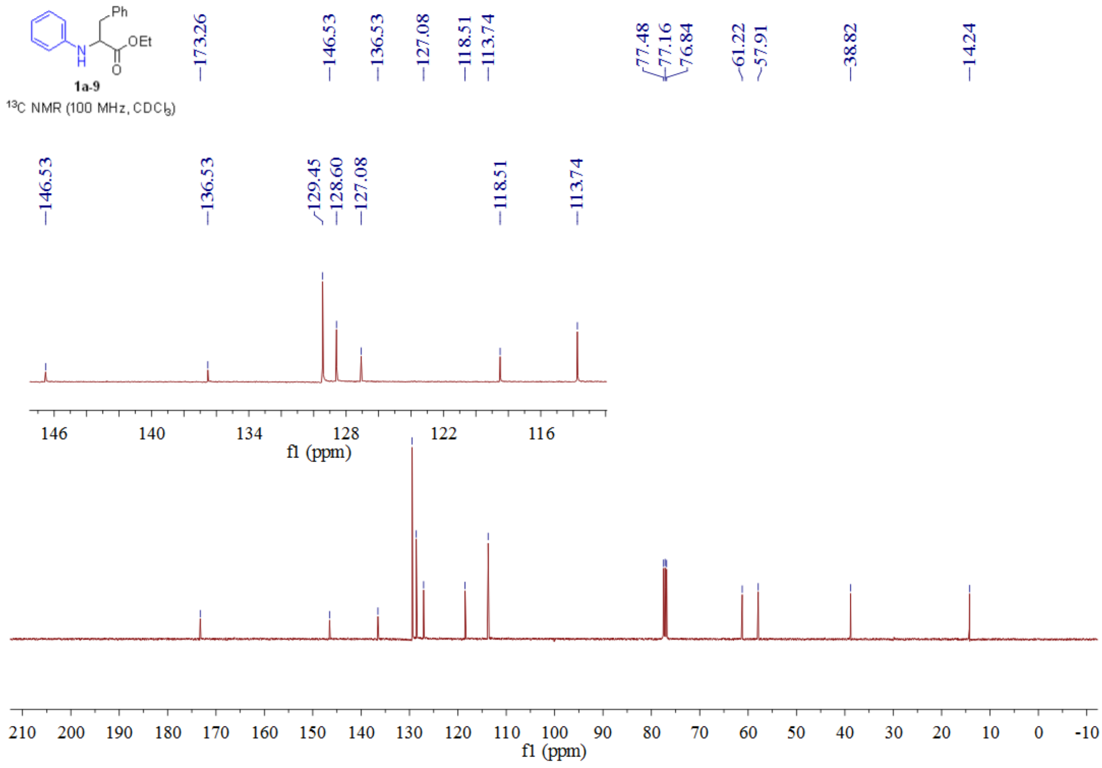

Supplementary Figure 11. ^{1}H NMR and ^{13}C NMR spectra for compound 1a-9

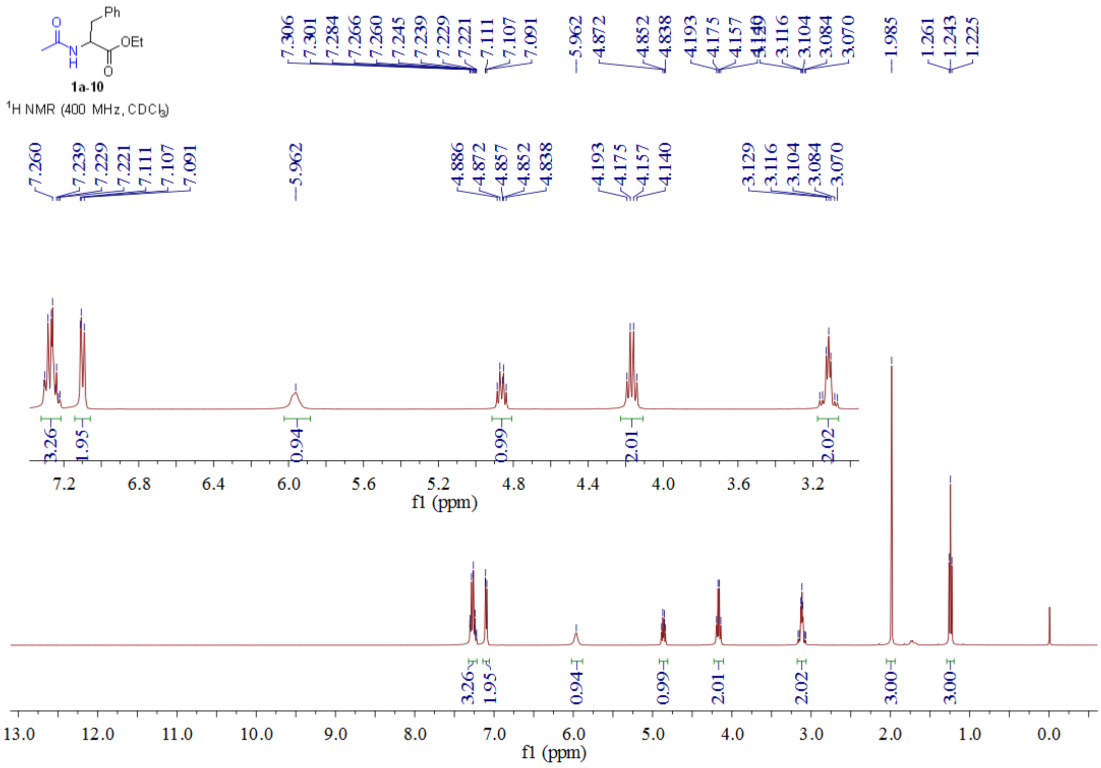

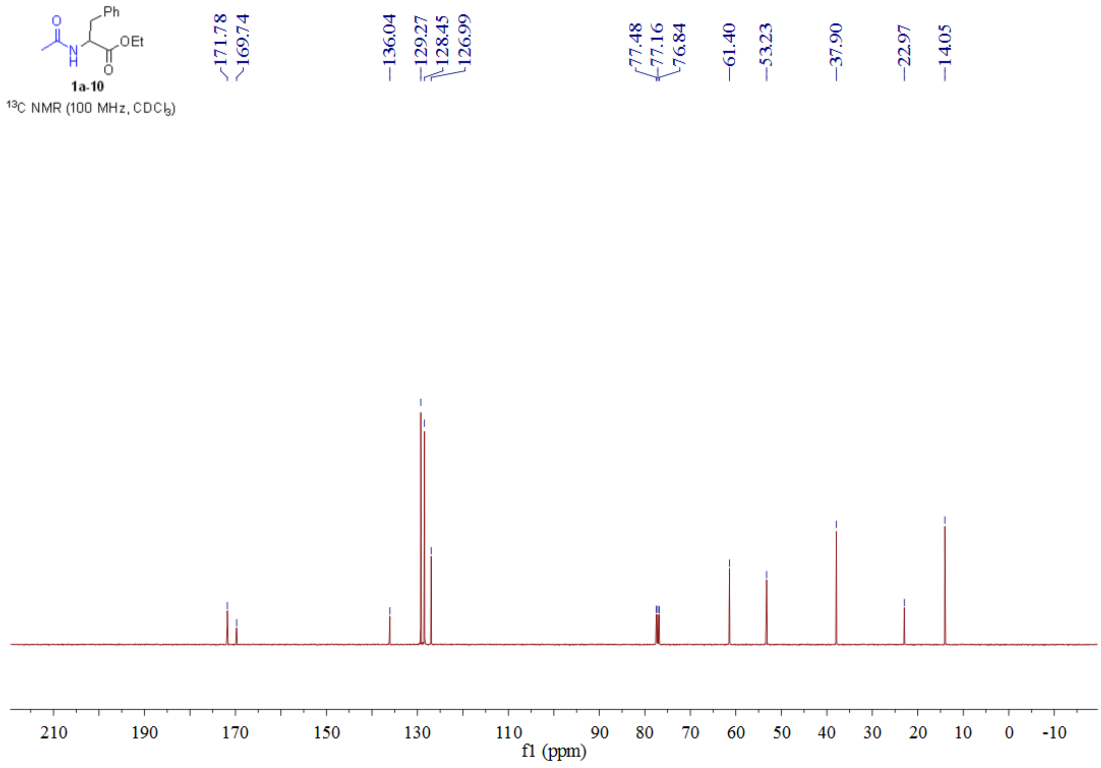

Supplementary Figure 12. ^{1}H NMR and ^{13}C NMR spectra for compound 1a-10

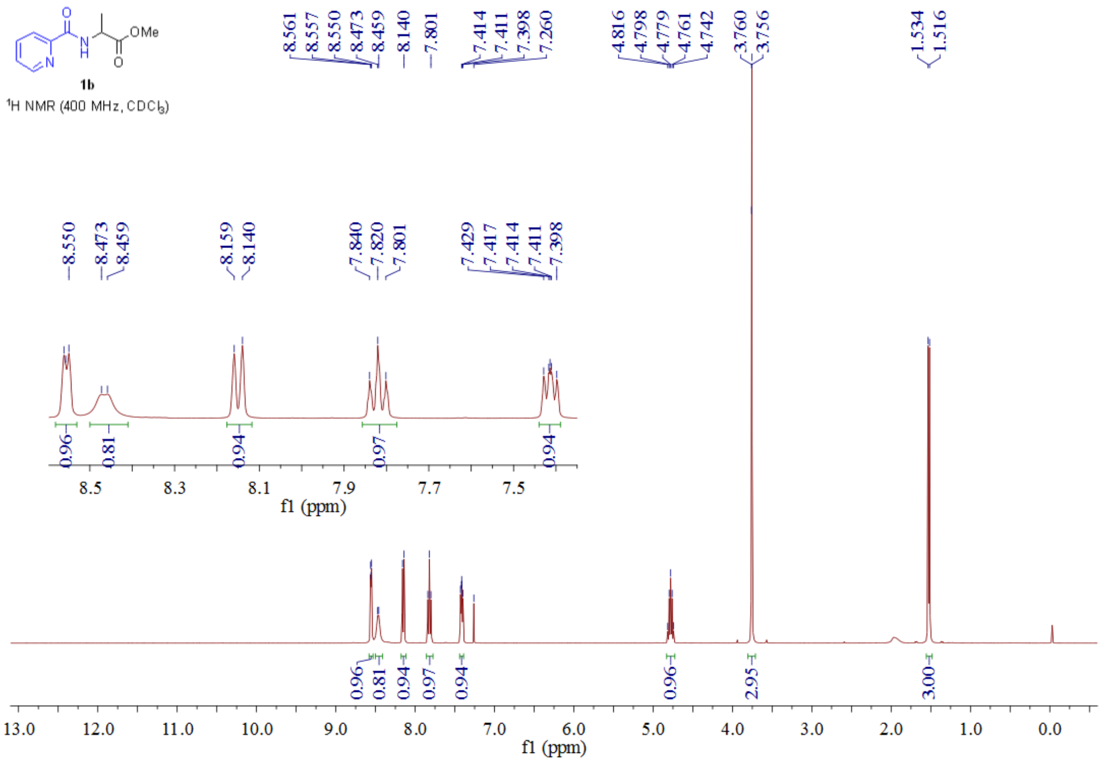

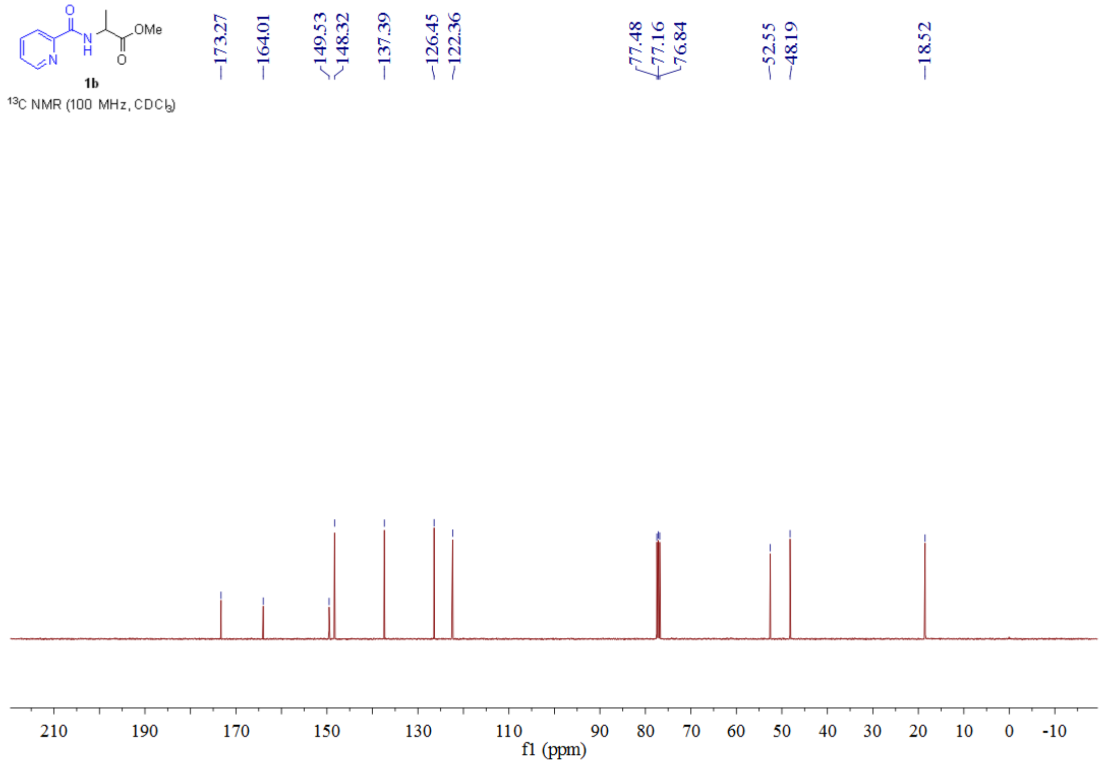

Supplementary Figure 13. ^{1}H NMR and ^{13}C NMR spectra for compound 1b

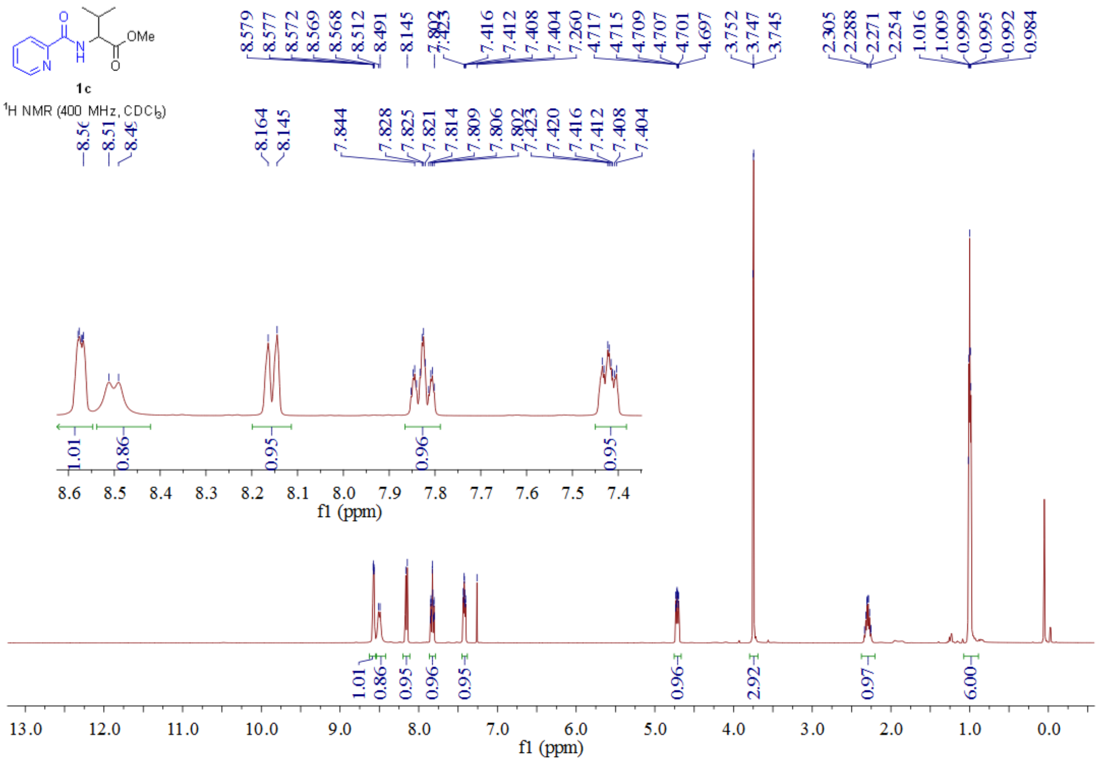

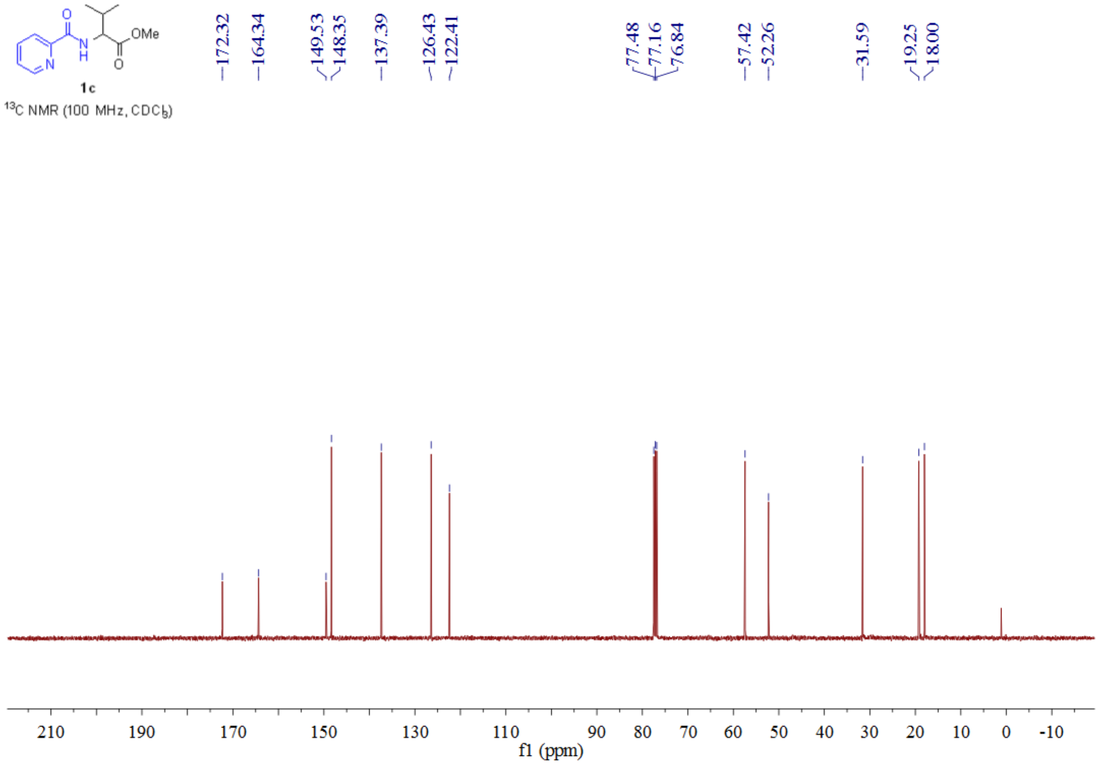

Supplementary Figure 14. ^{1}H NMR and ^{13}C NMR spectra for compound 1c

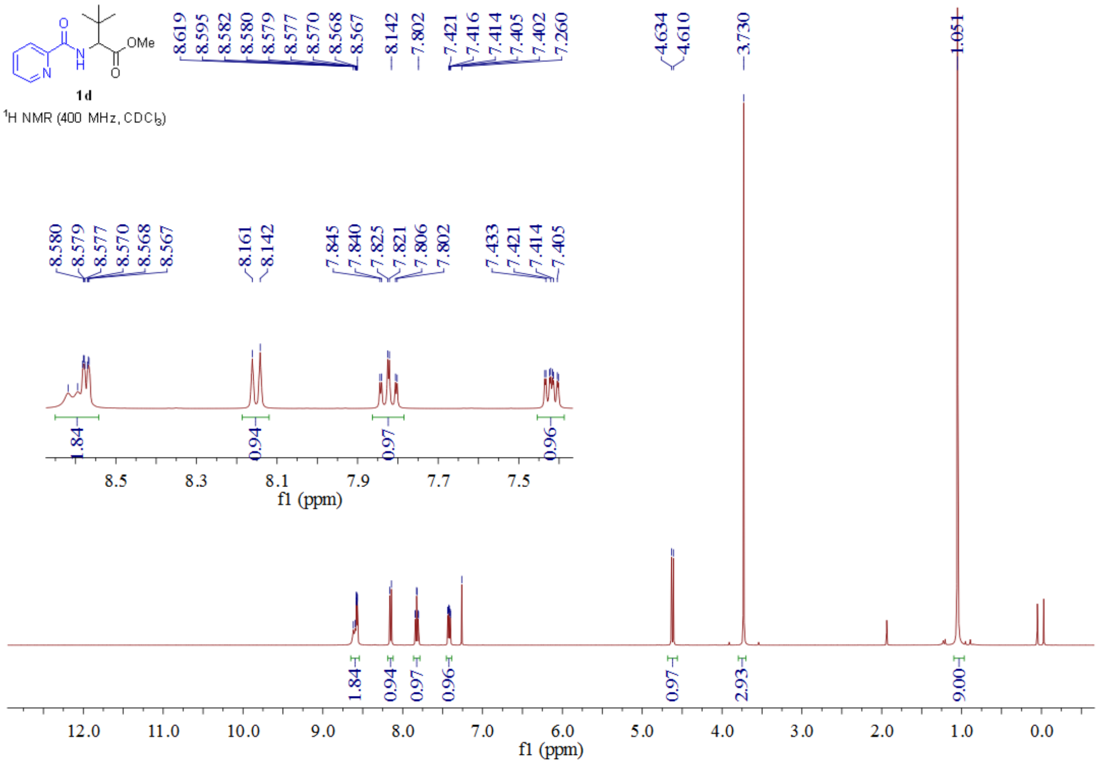

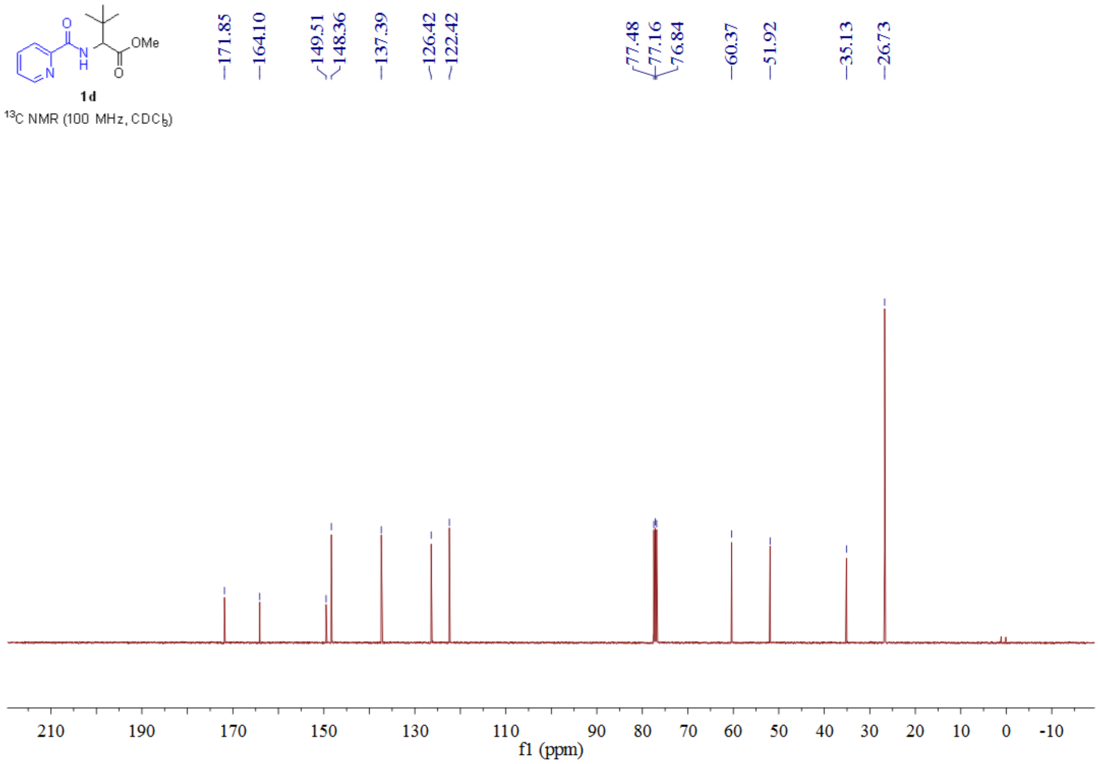

Supplementary Figure 15. ^{1}H NMR and ^{13}C NMR spectra for compound 1d

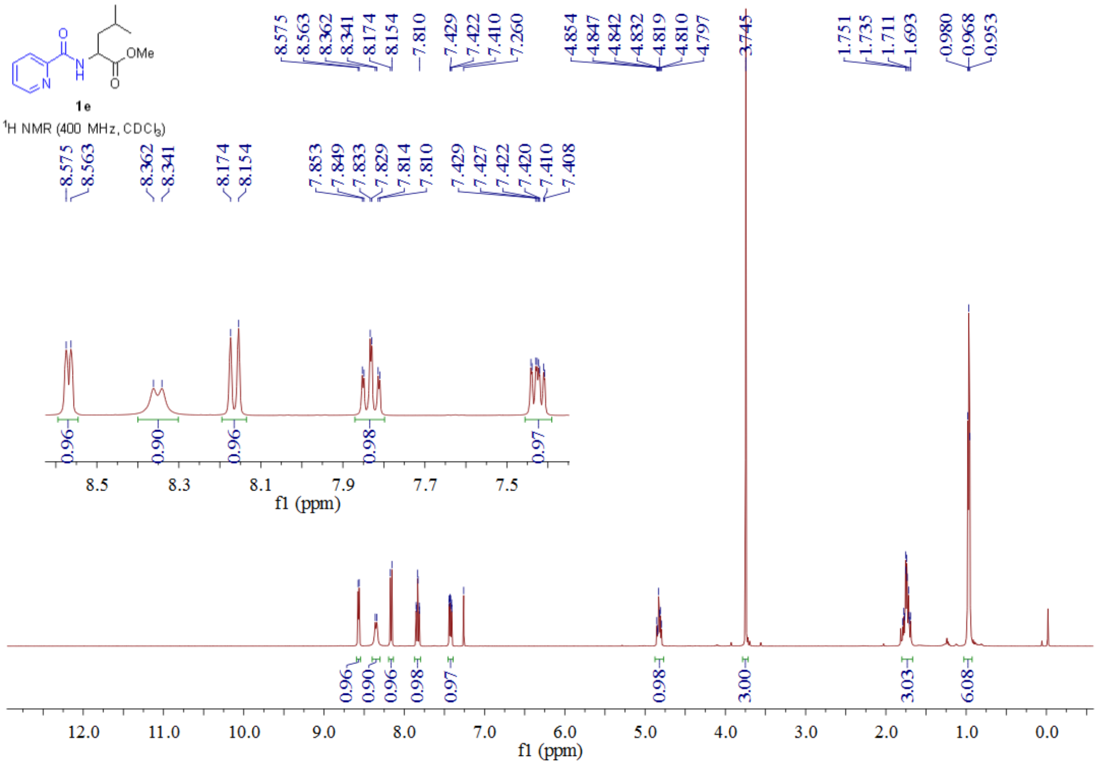

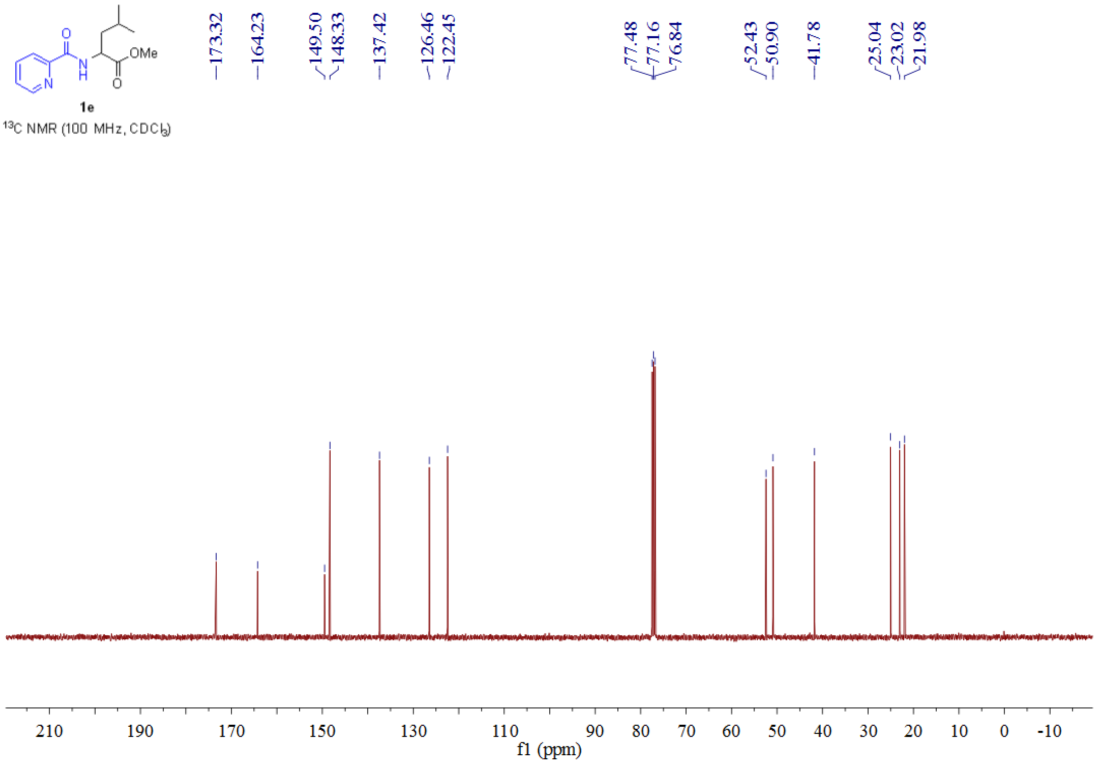

Supplementary Figure 16. ^{1}H NMR and ^{13}C NMR spectra for compound 1e

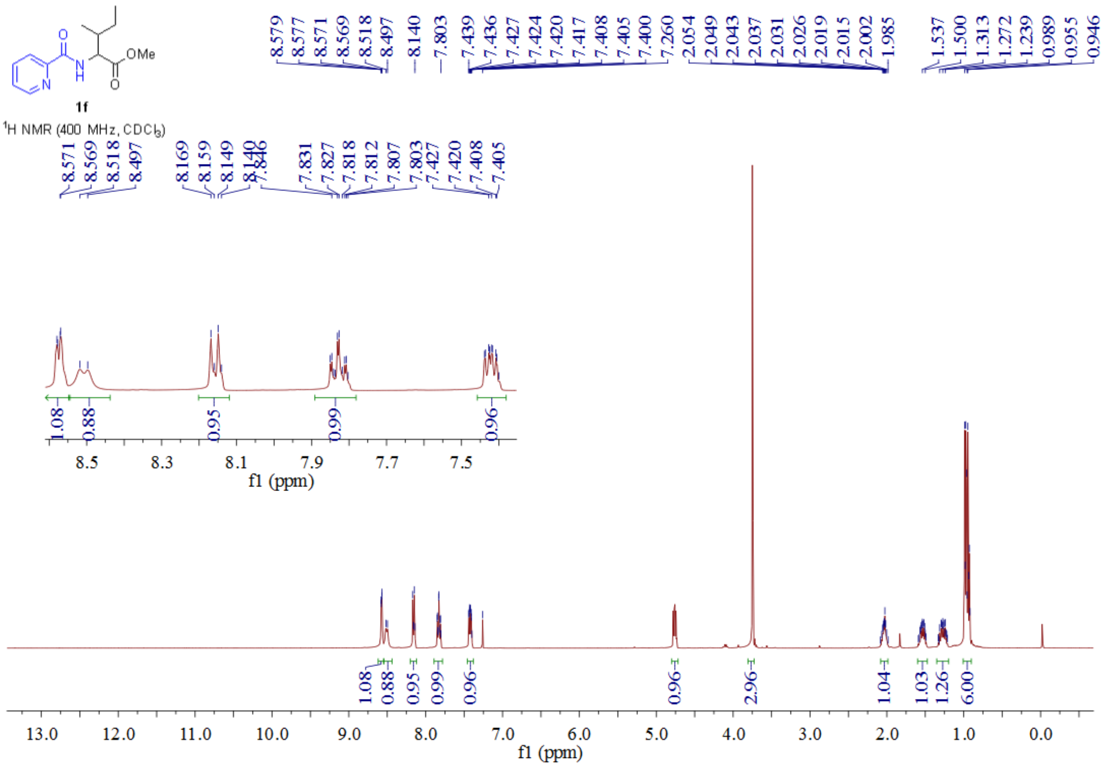

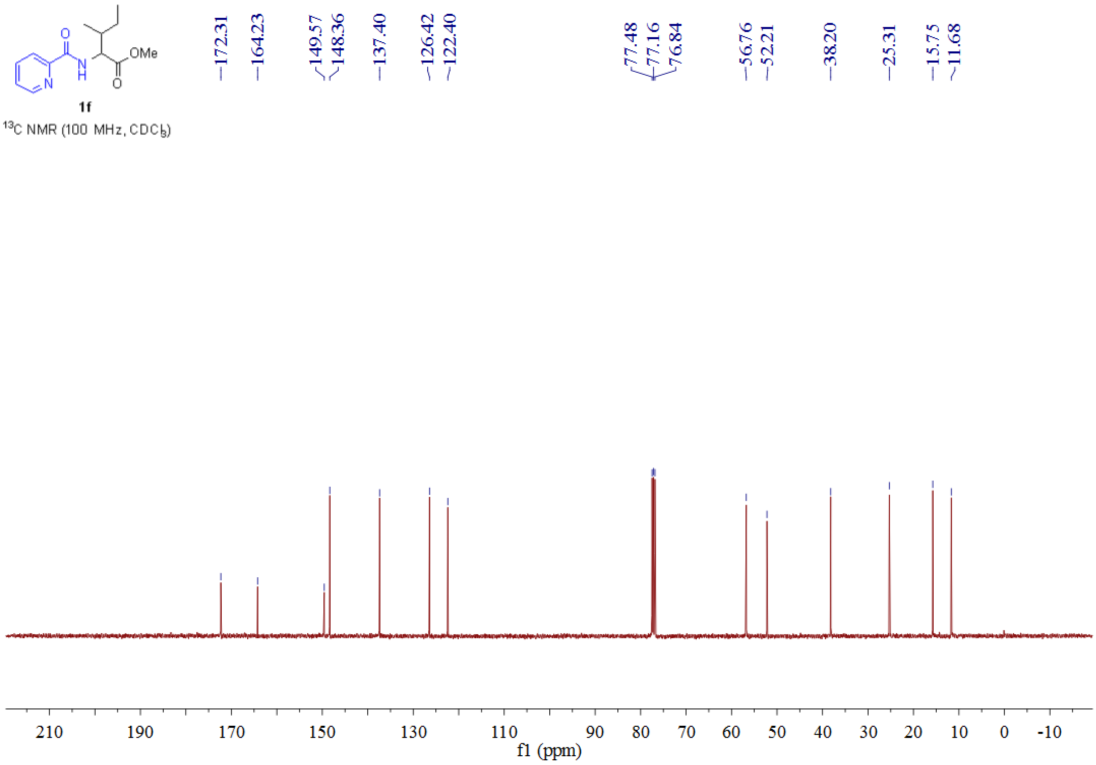

Supplementary Figure 17. ^{1}H NMR and ^{13}C NMR spectra for compound 1f

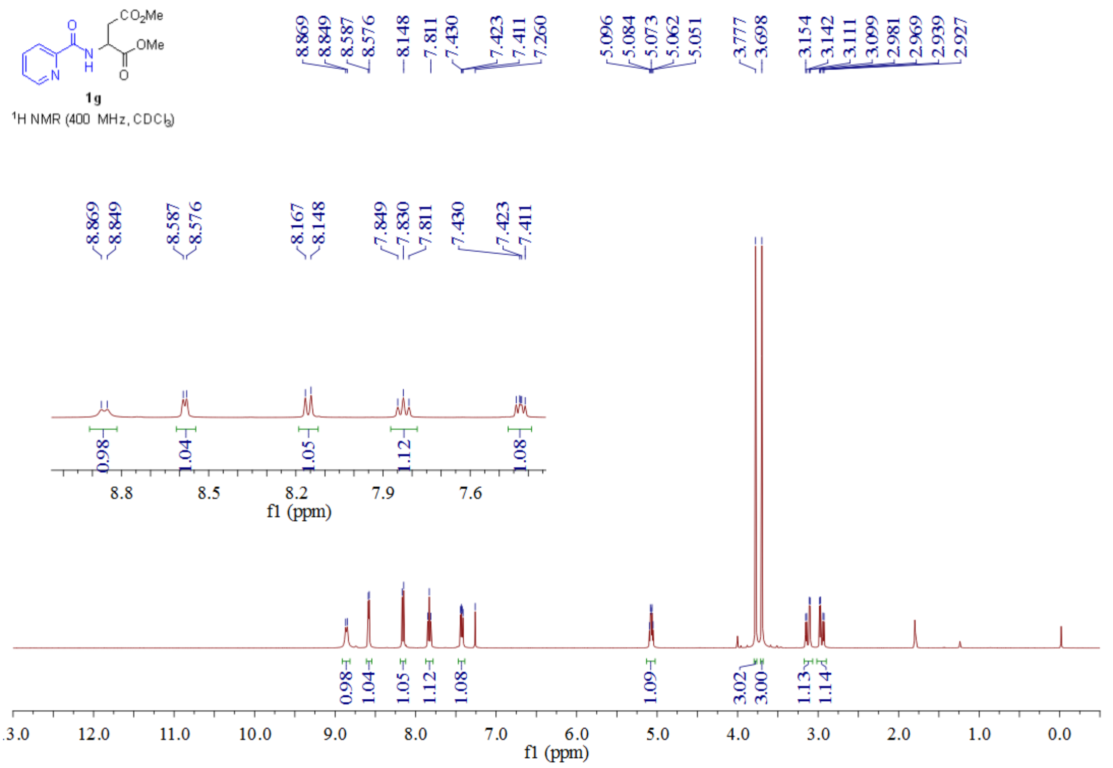

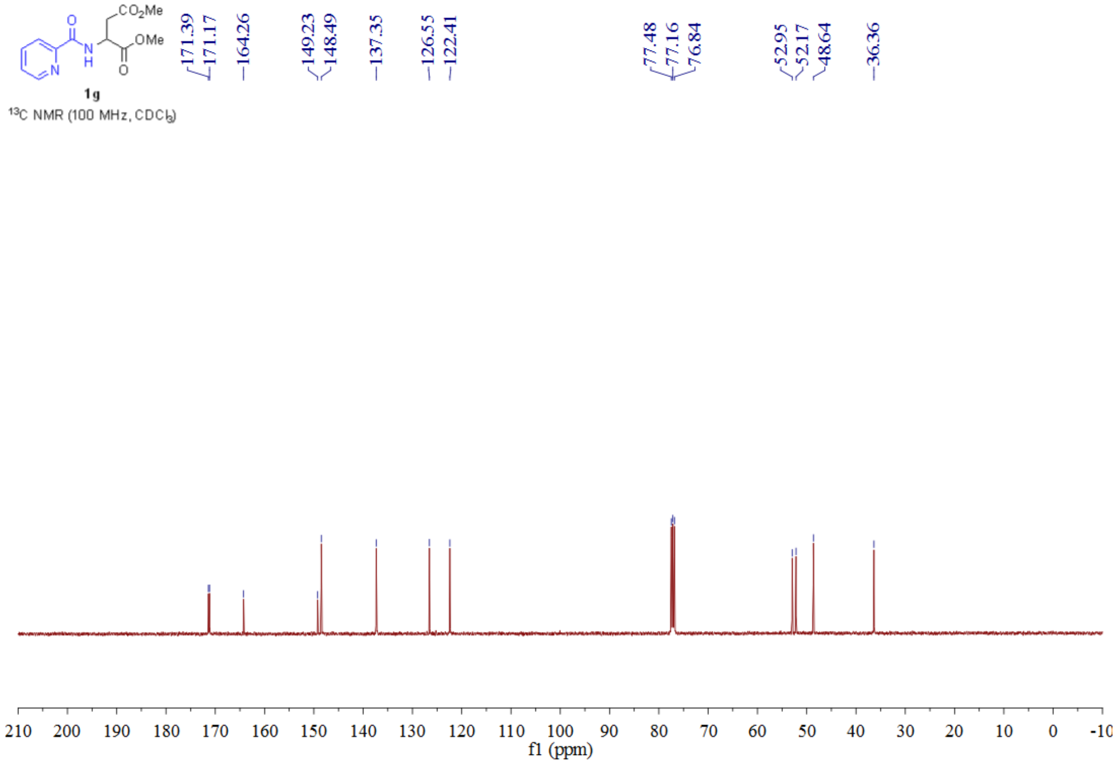

Supplementary Figure 18. ^{1}H NMR and ^{13}C NMR spectra for compound 1g

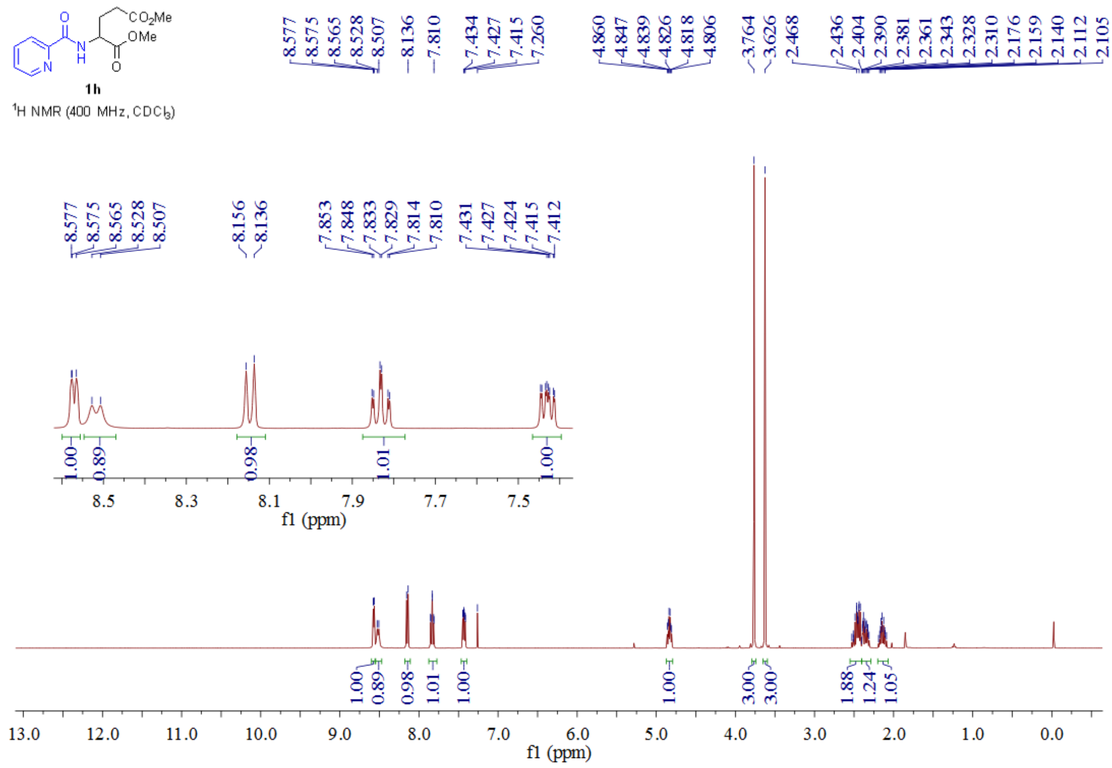

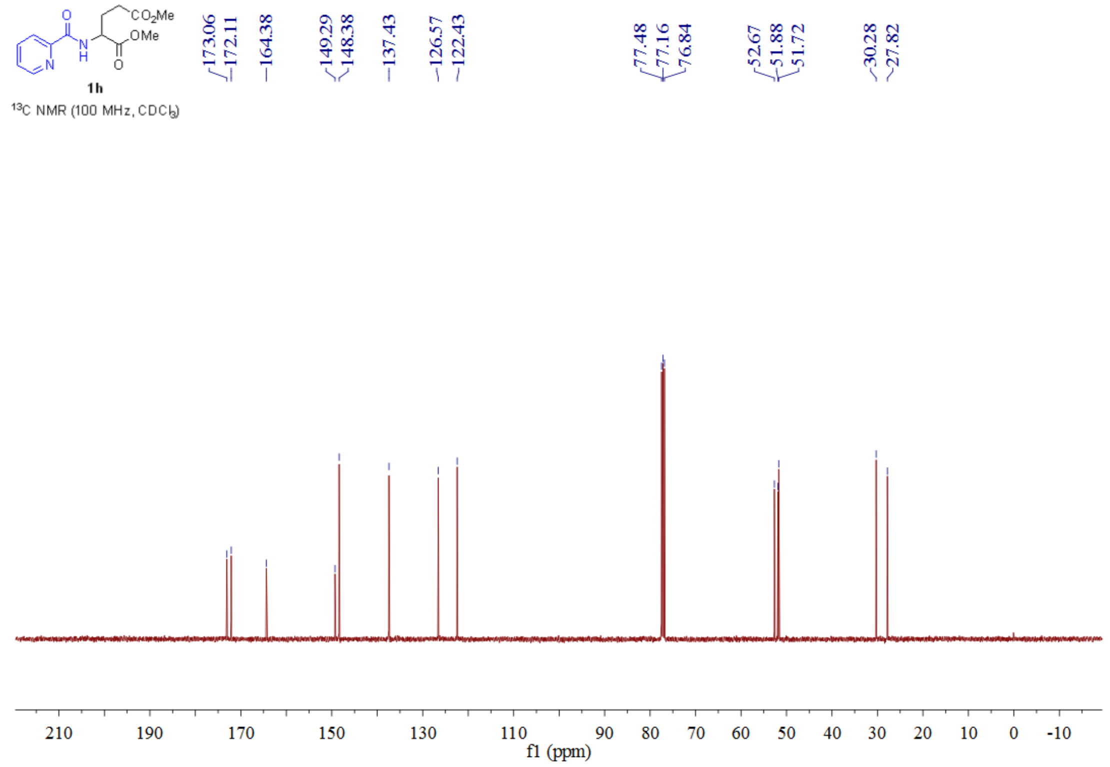

Supplementary Figure 19. ^{1}H NMR and ^{13}C NMR spectra for compound 1h

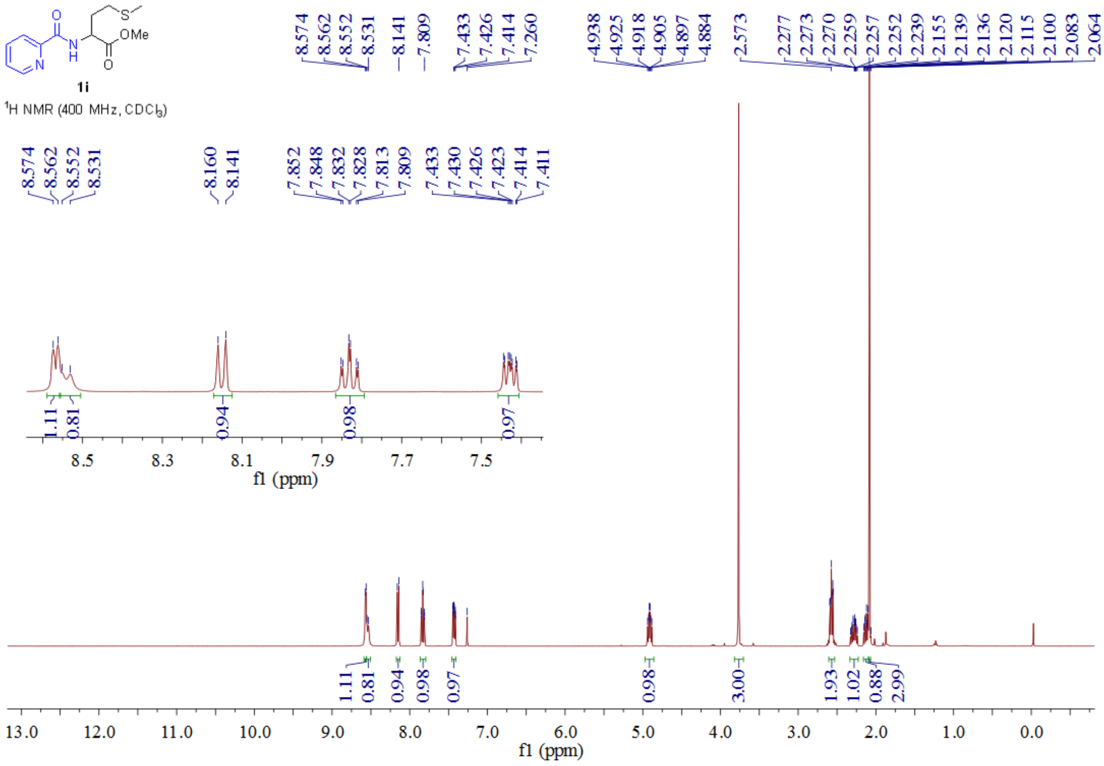

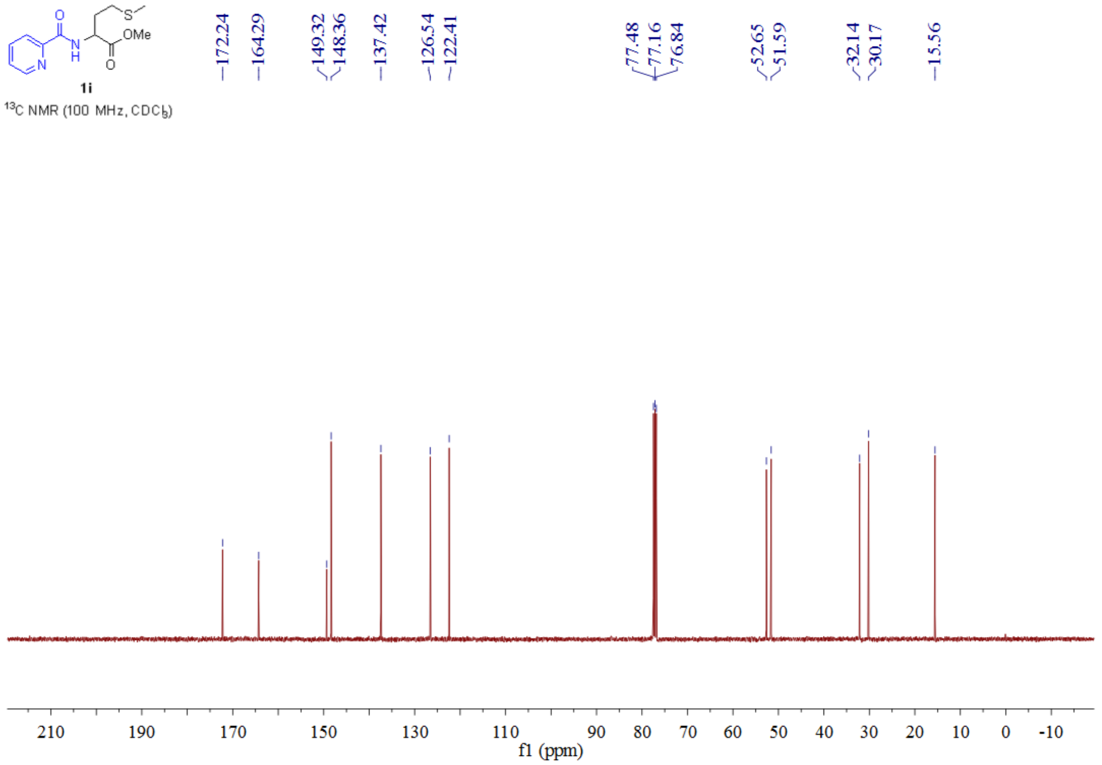

Supplementary Figure 20. ^{1}H NMR and ^{13}C NMR spectra for compound 1i

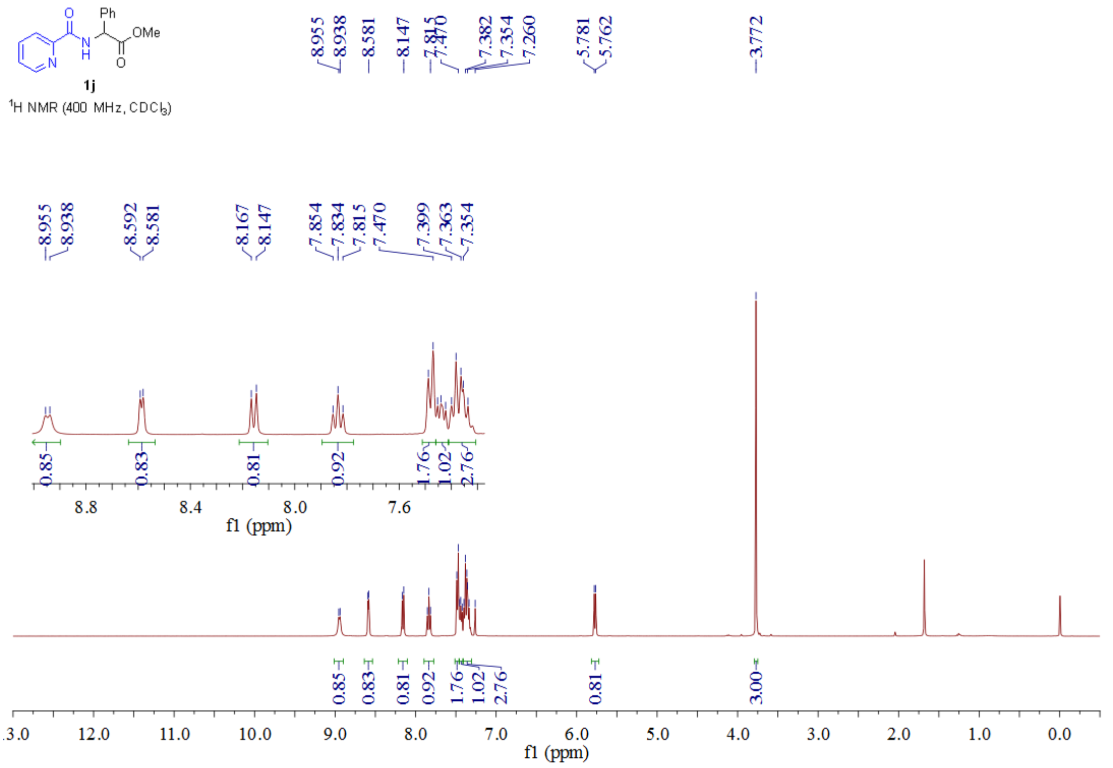

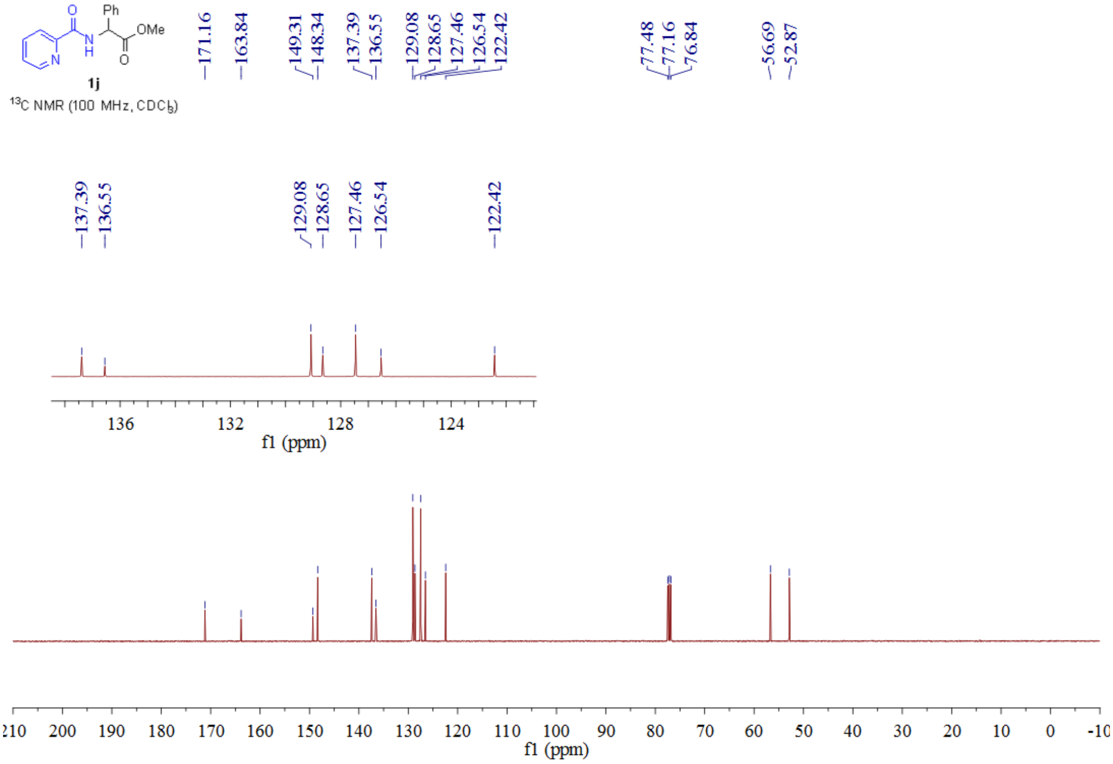

Supplementary Figure 21. ^{1}H NMR and ^{13}C NMR spectra for compound 1j

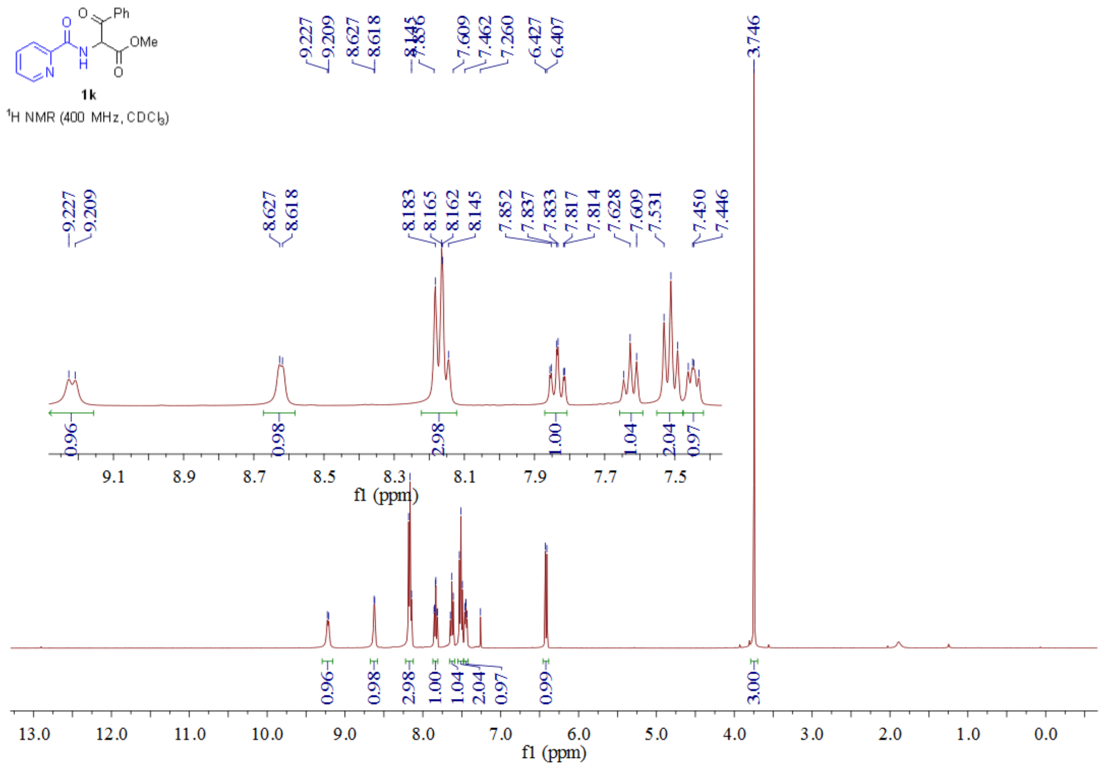

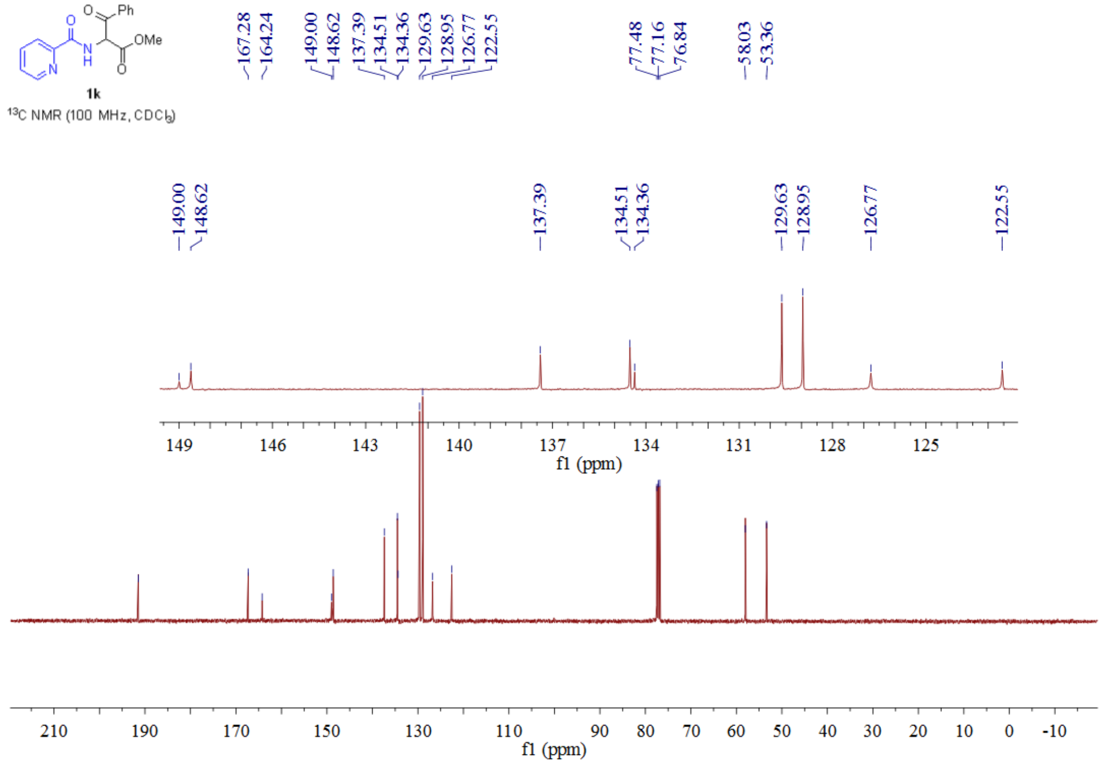

Supplementary Figure 22. ^{1}H NMR and ^{13}C NMR spectra for compound 1k

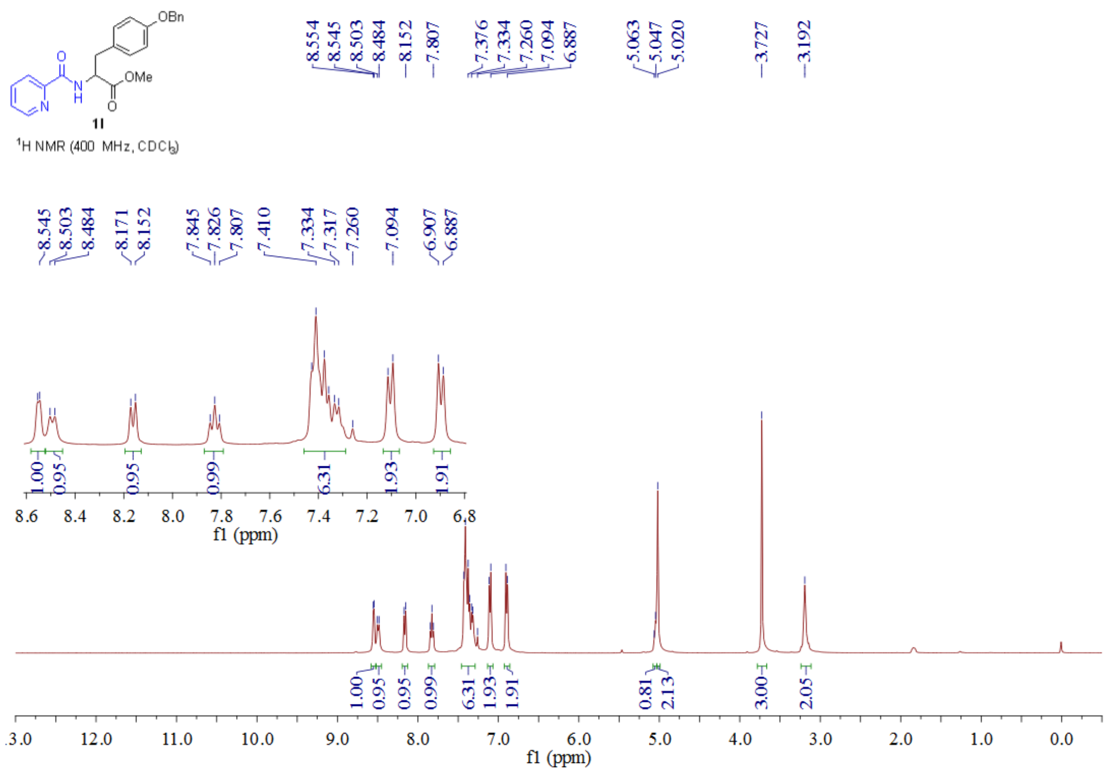

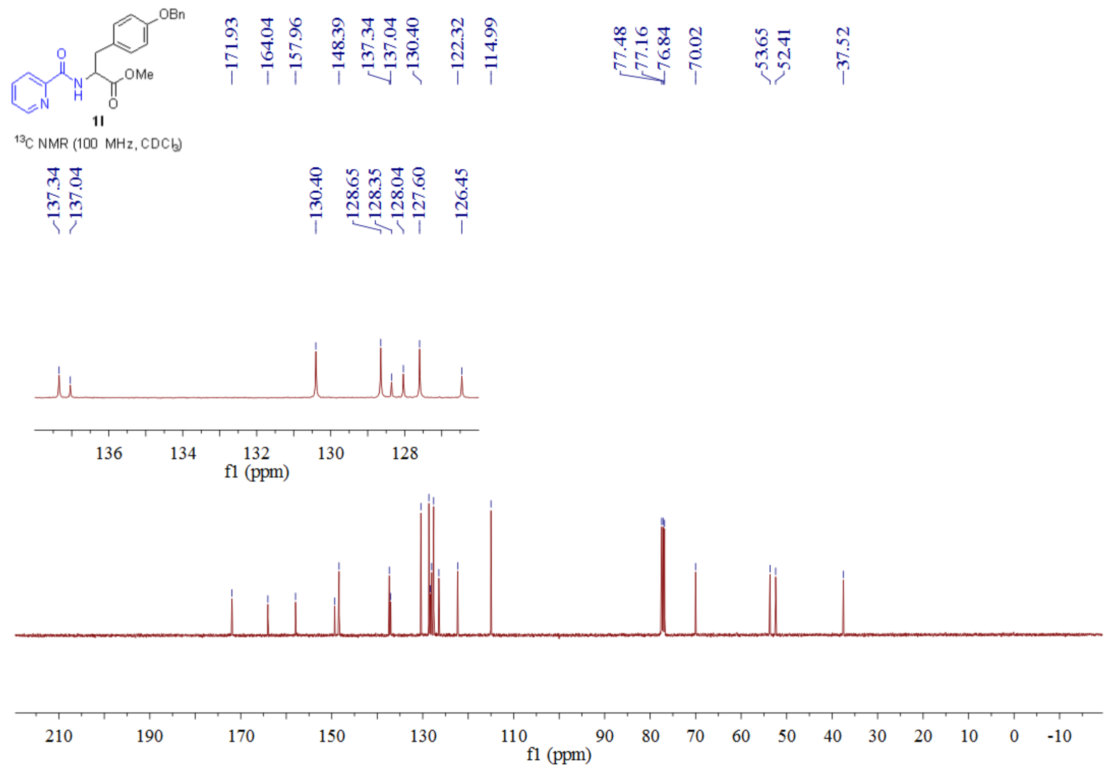

Supplementary Figure 23. ^{1}H NMR and ^{13}C NMR spectra for compound 1l

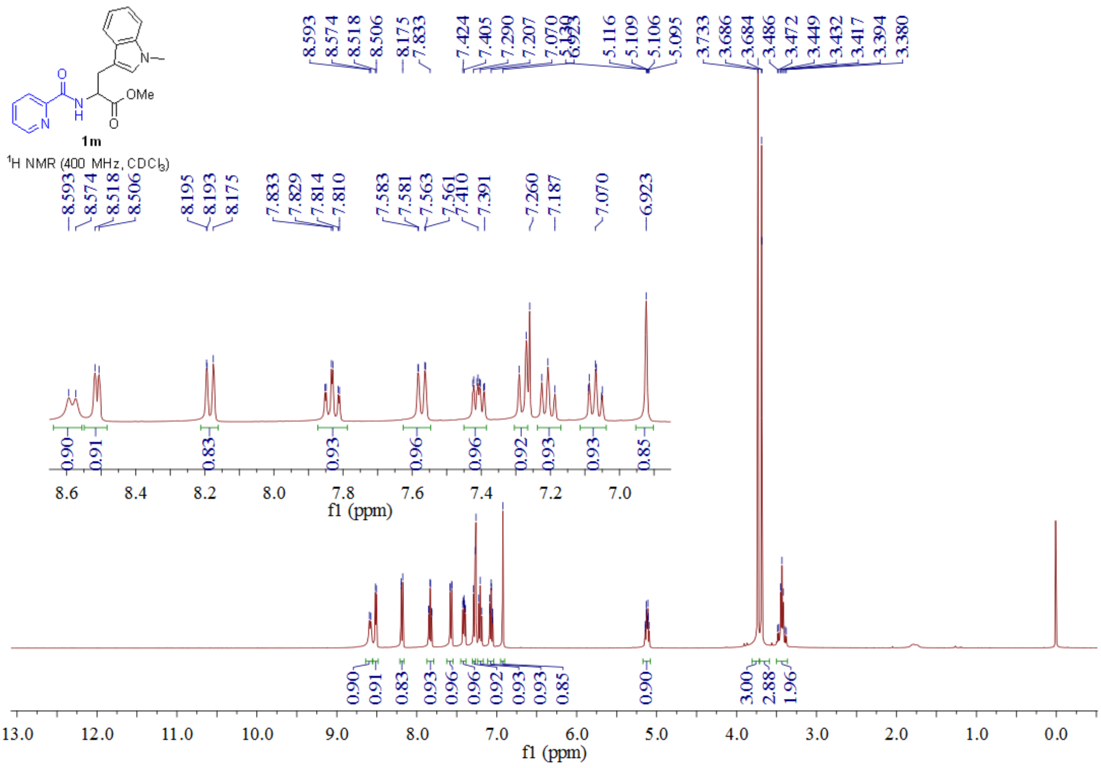

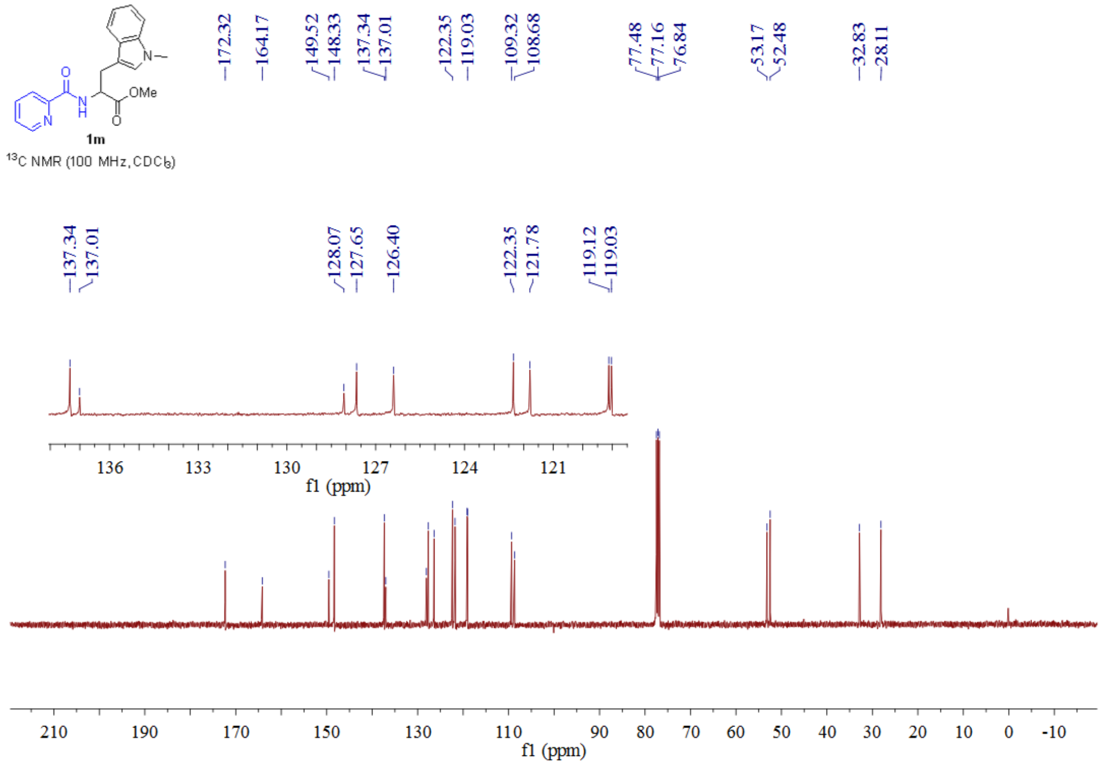

Supplementary Figure 24. ^{1}**H NMR and** ^{13}**C NMR spectra for compound 1m**

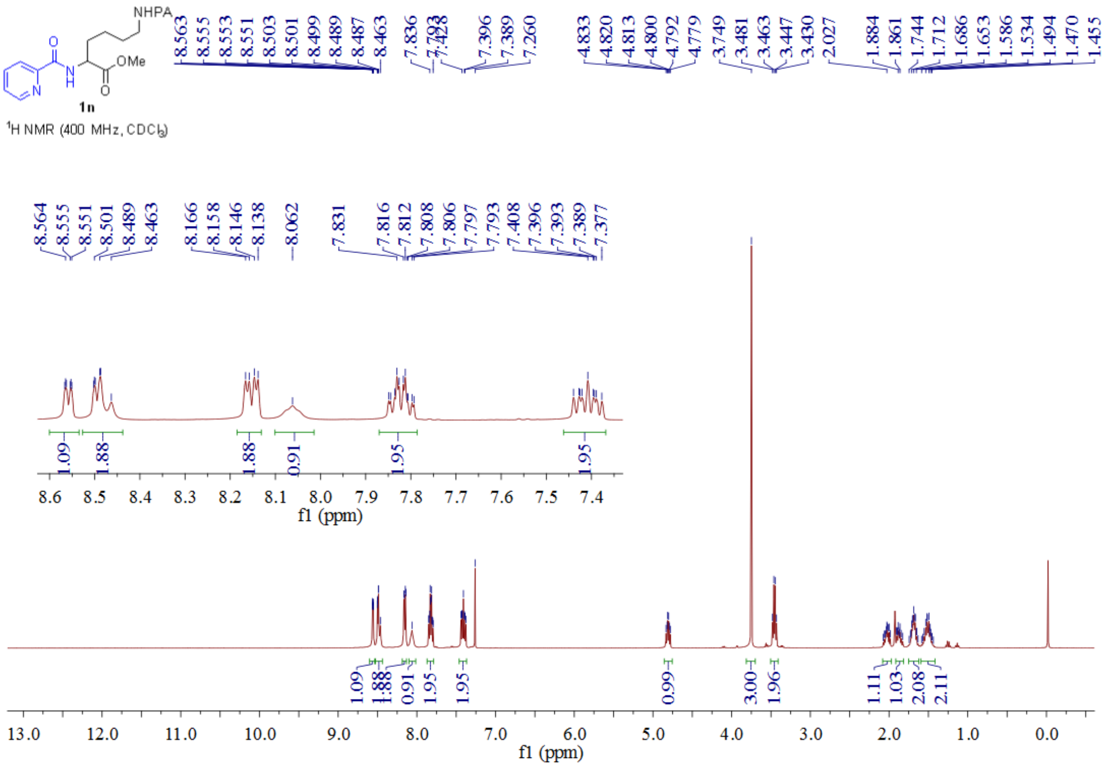

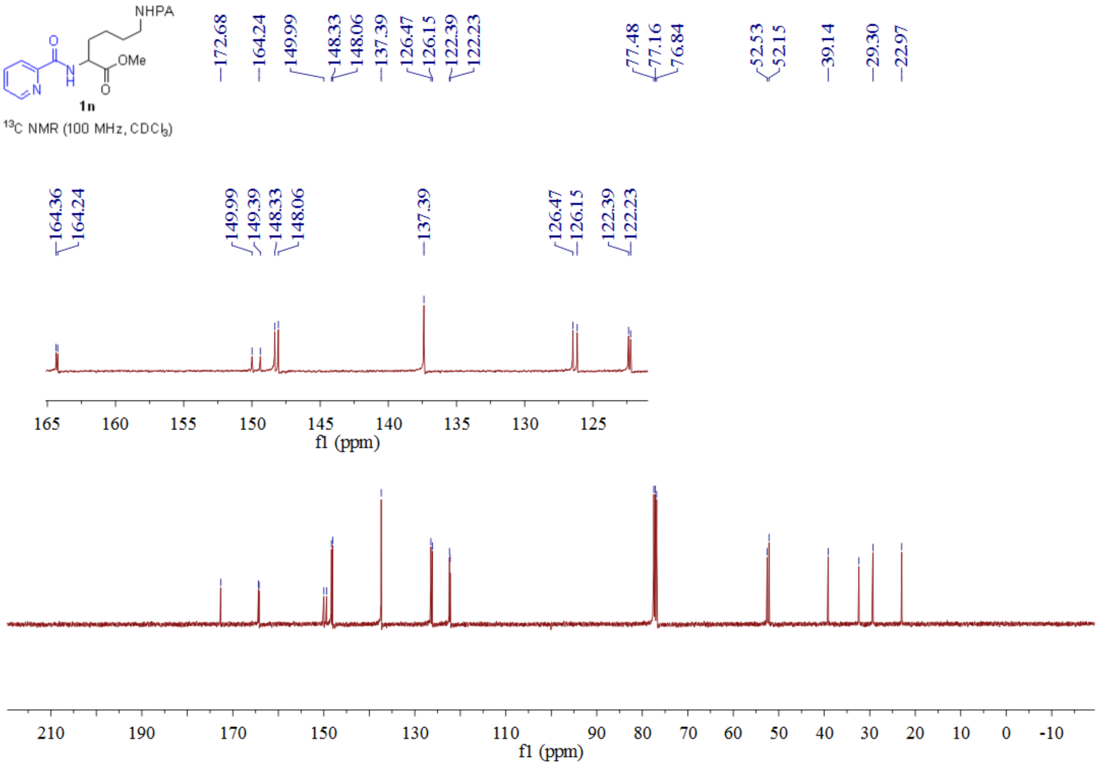

Supplementary Figure 25. ^{1}H NMR and ^{13}C NMR spectra for compound 1n

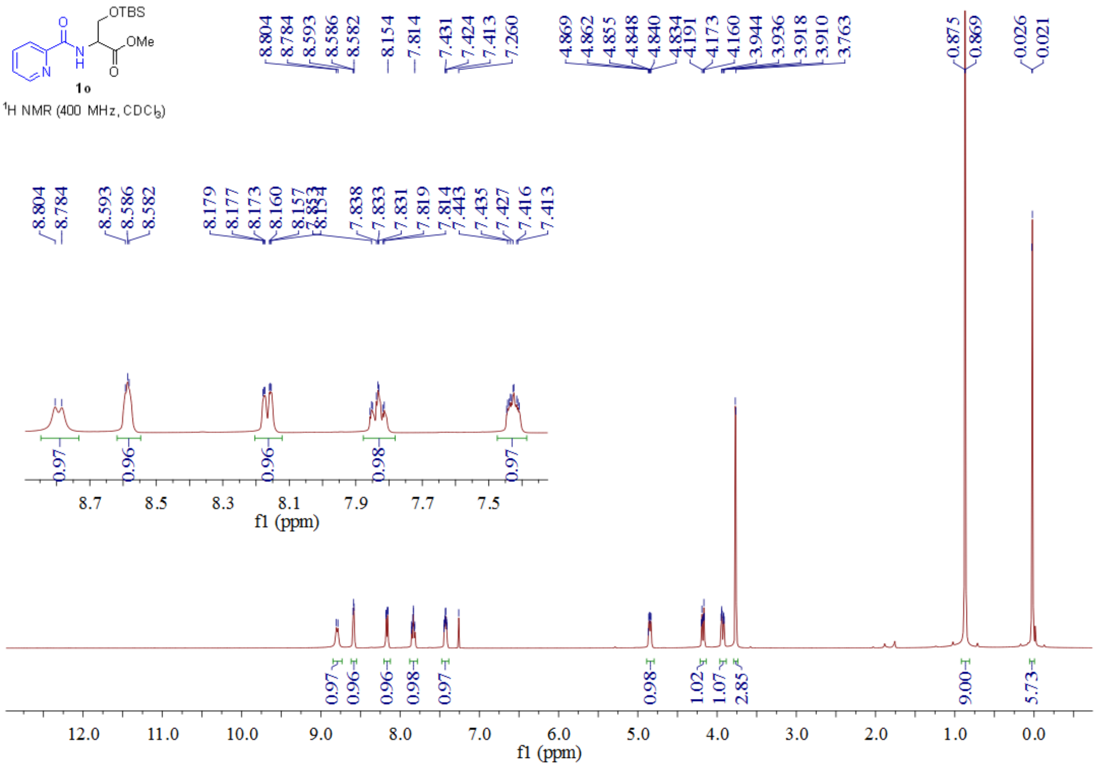

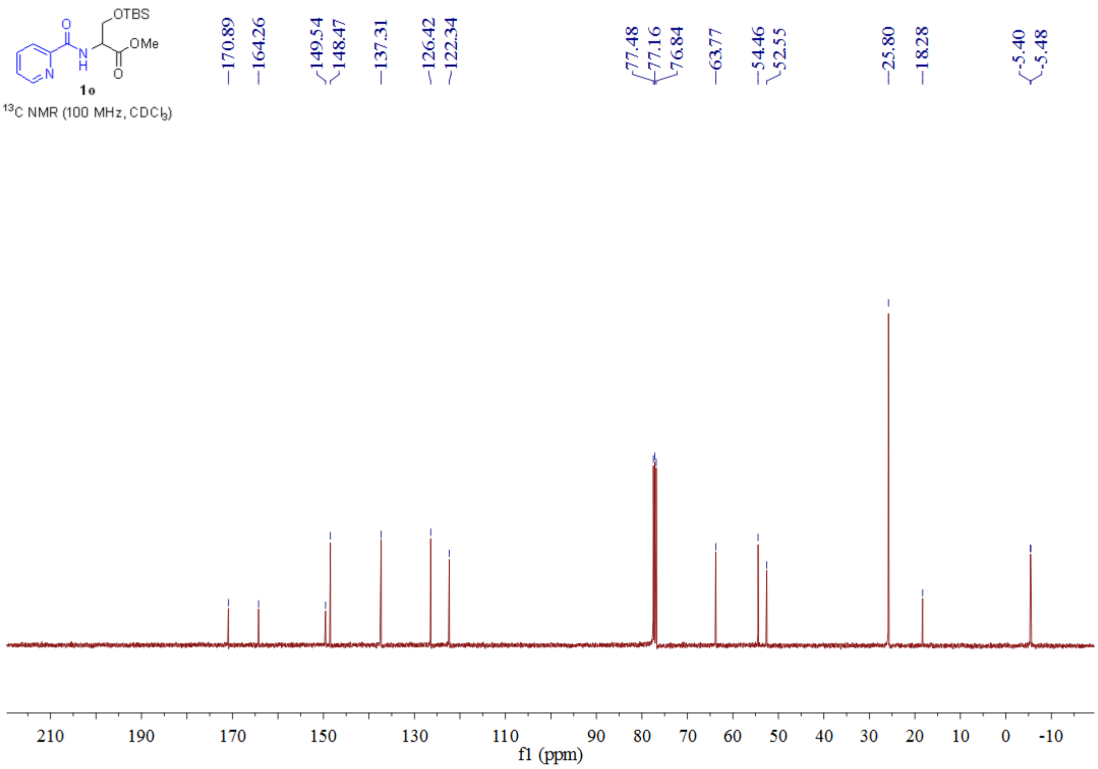

Supplementary Figure 26. ^{1}**H NMR and** ^{13}**C NMR spectra for compound 1o**

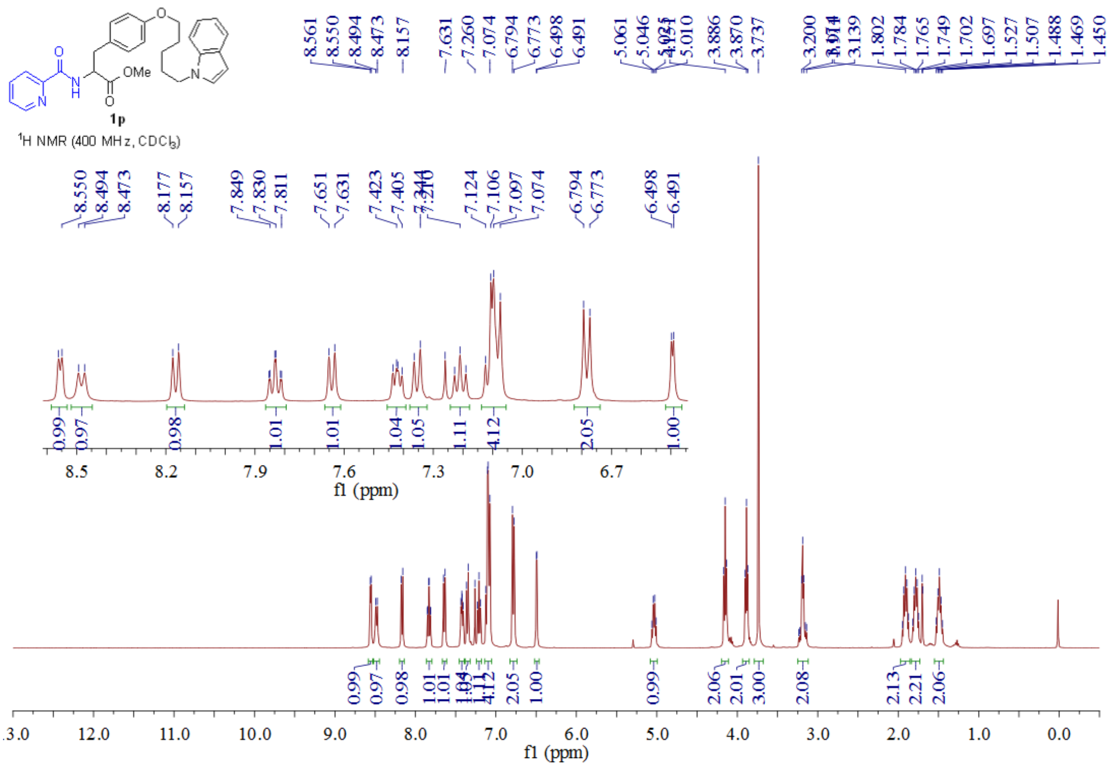

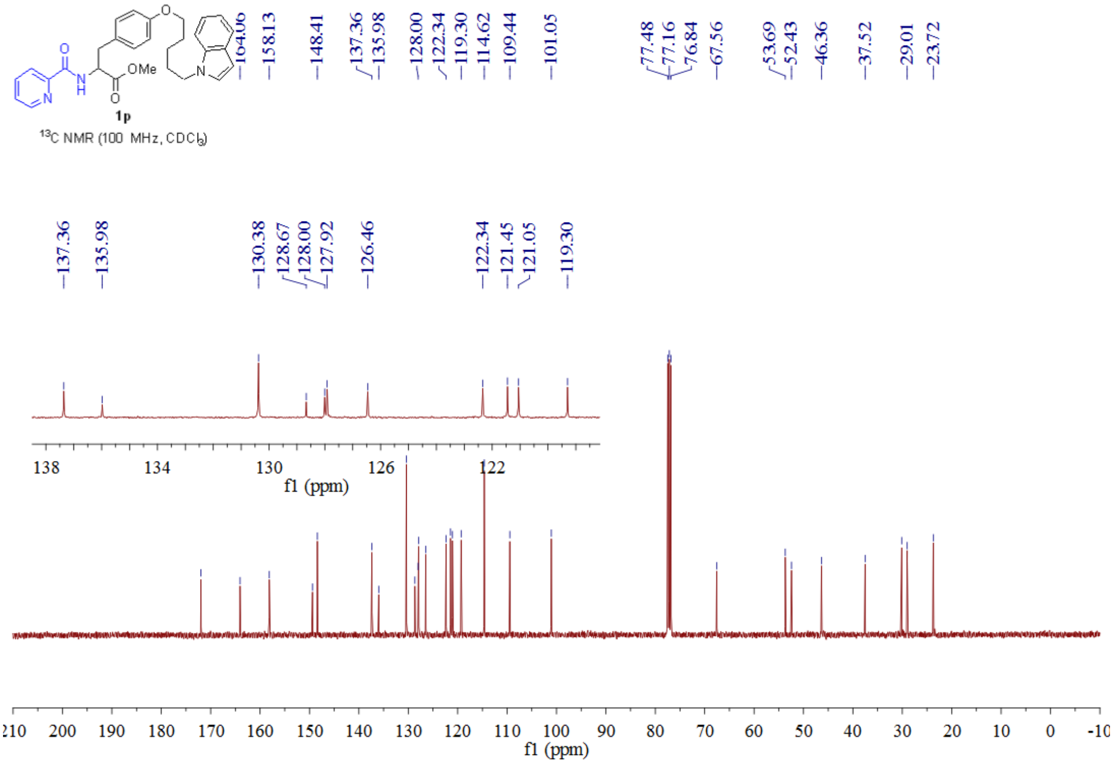

Supplementary Figure 27. ^{1}H NMR and ^{13}C NMR spectra for compound 1p

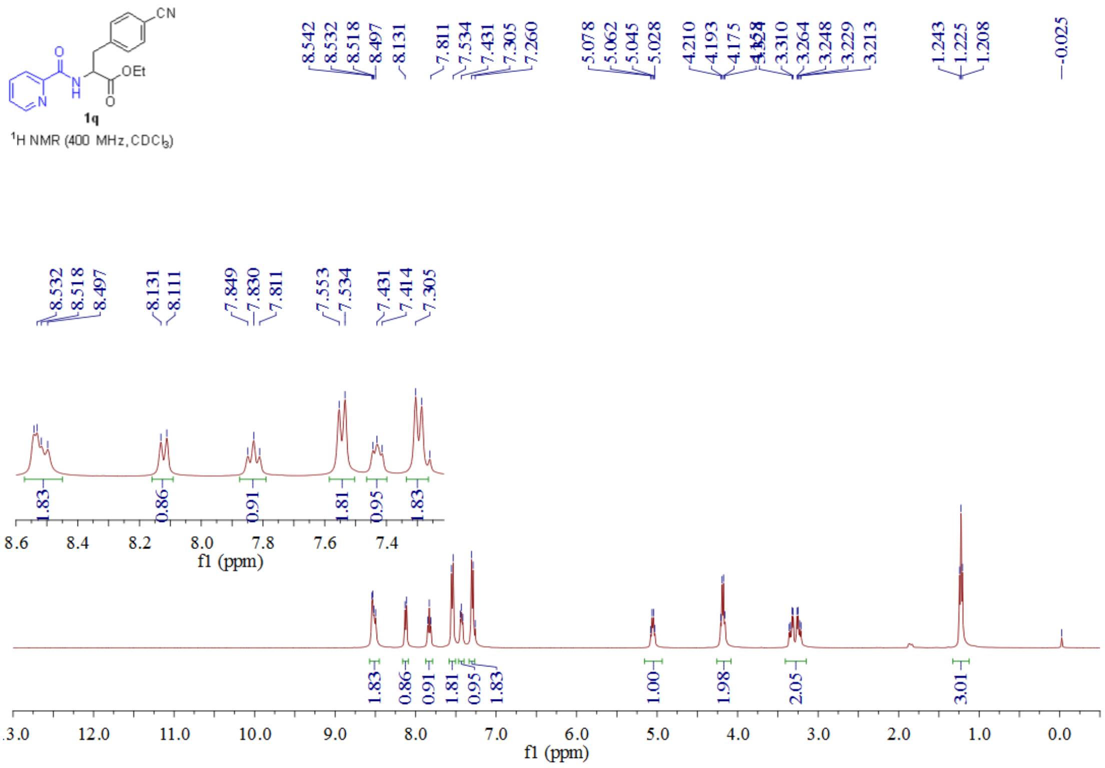

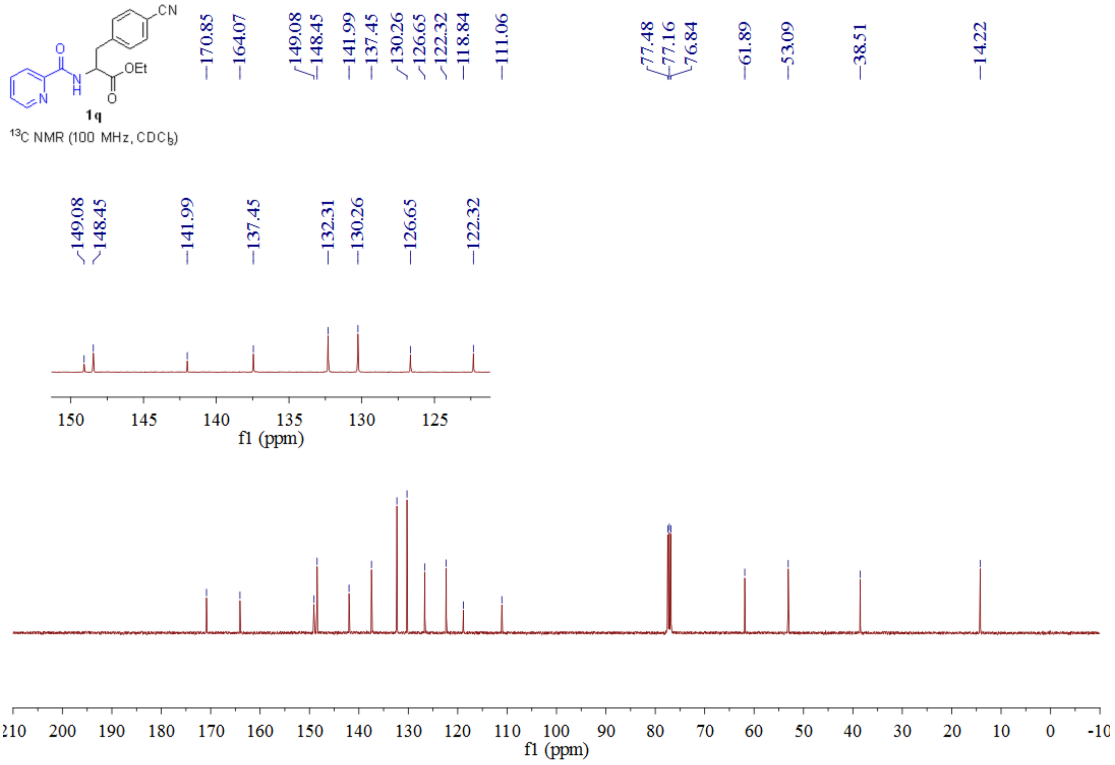

Supplementary Figure 28. ^{1}**H NMR and** ^{13}**C NMR spectra for compound 1q**

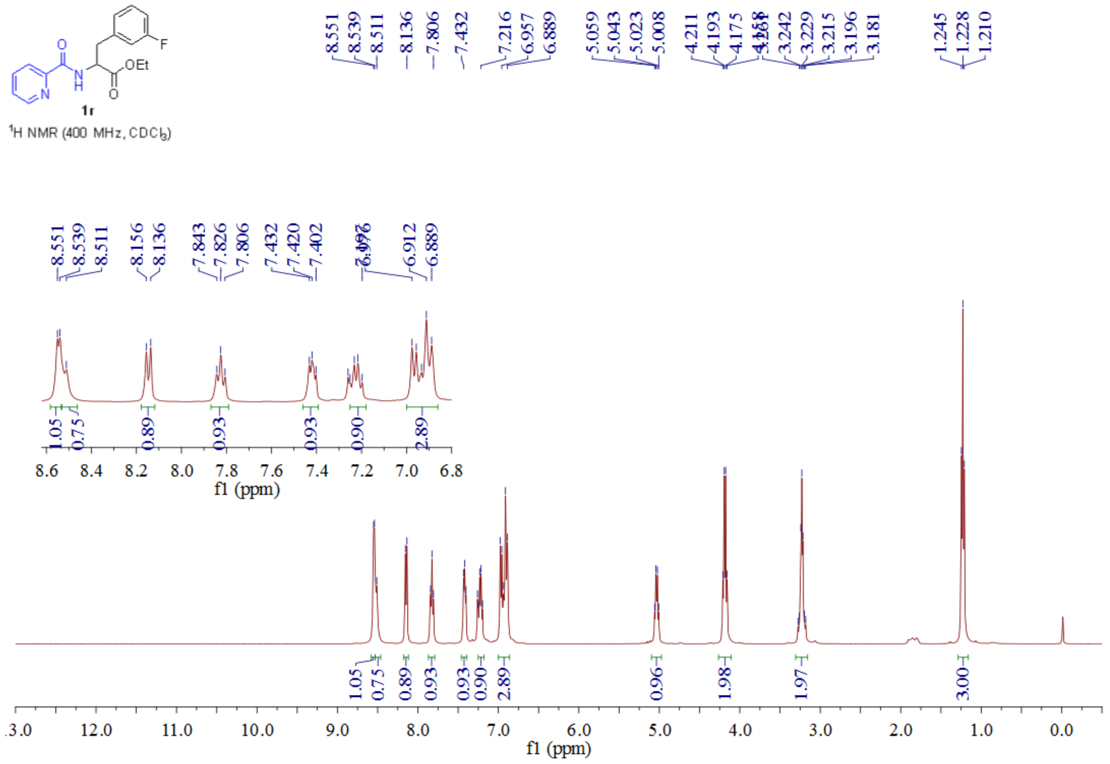

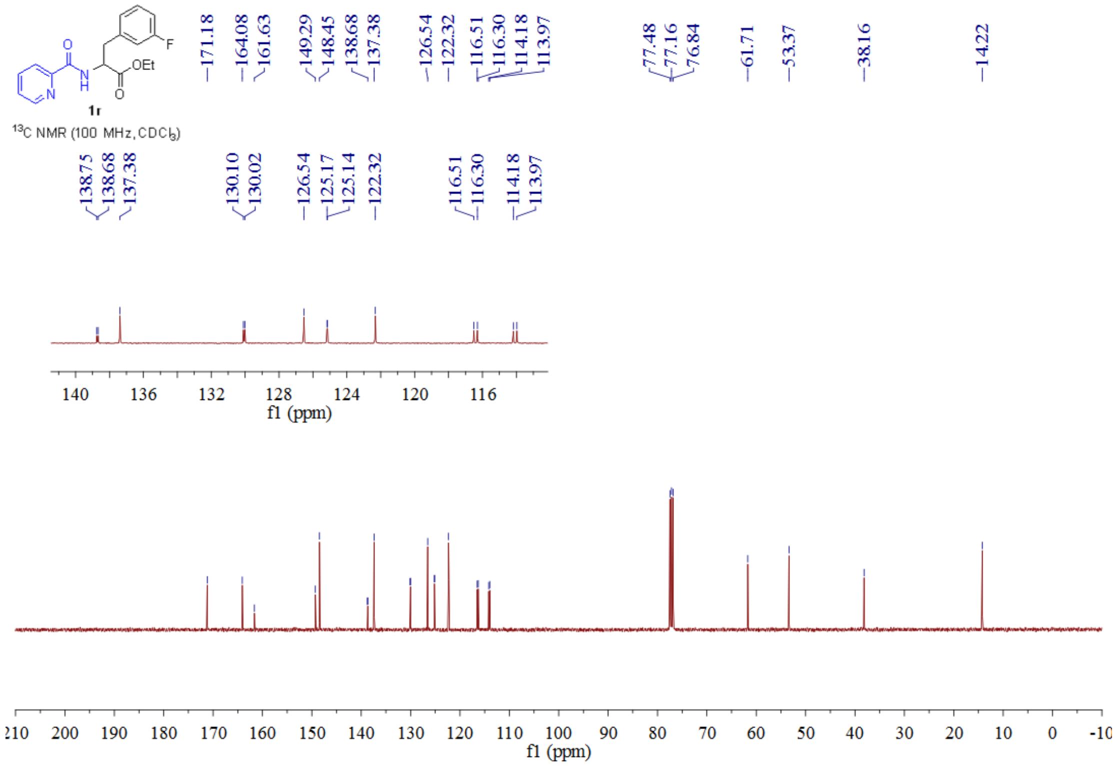

Supplementary Figure 29. ^{1}H NMR and ^{13}C NMR spectra for compound 1r

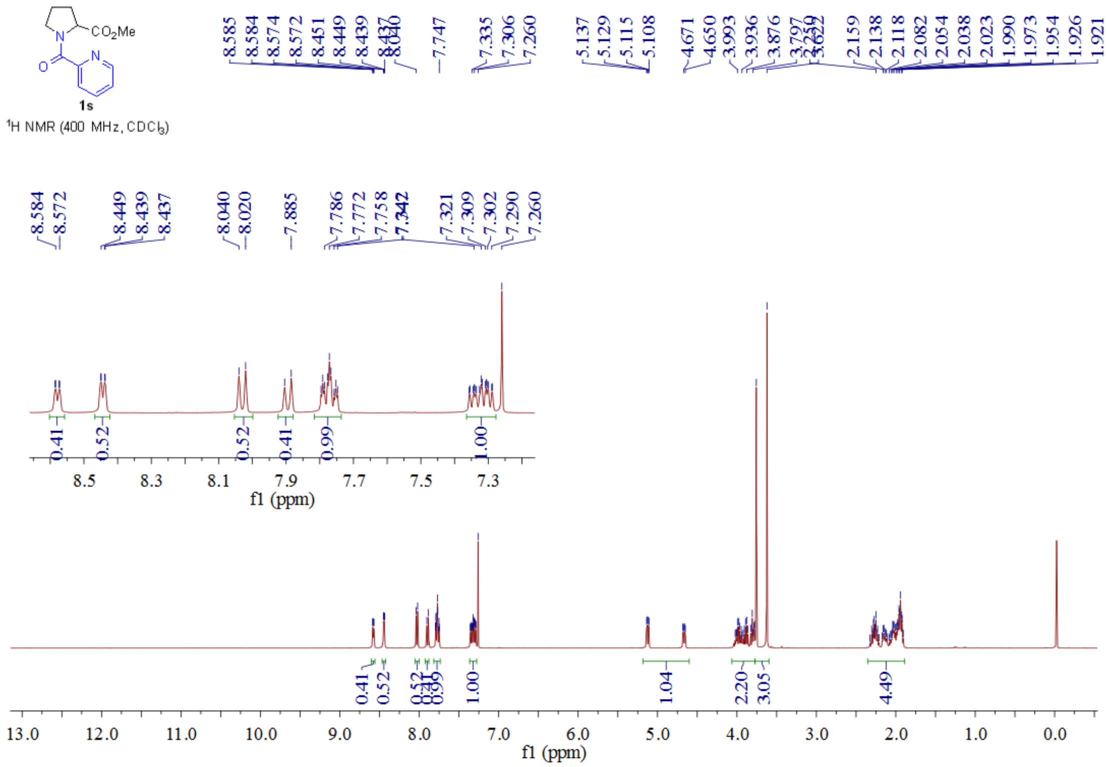

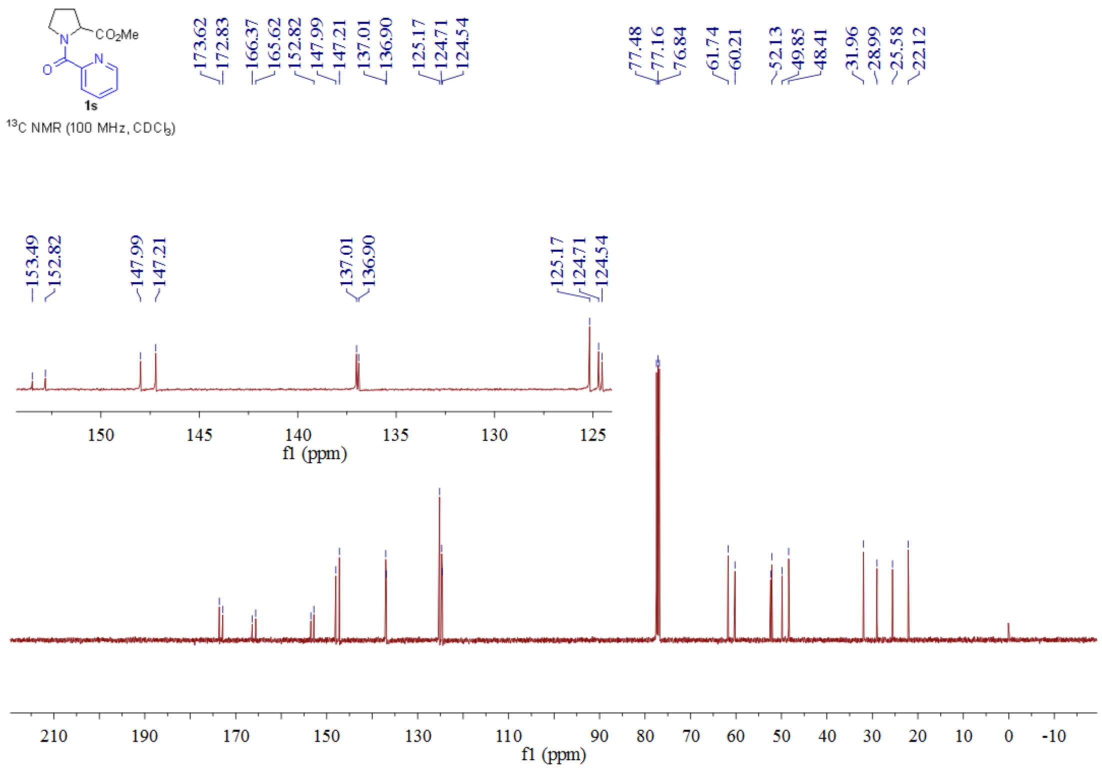

Supplementary Figure 30. **^{1}H NMR and ^{13}C NMR spectra for compound 1s**

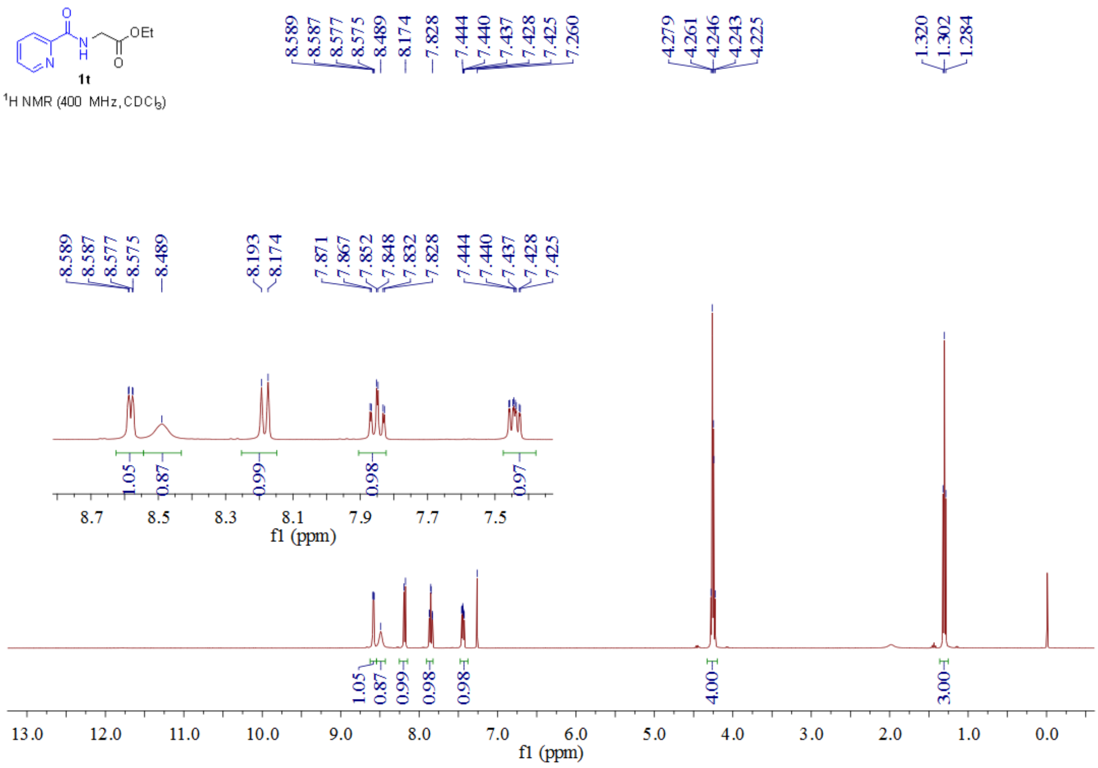

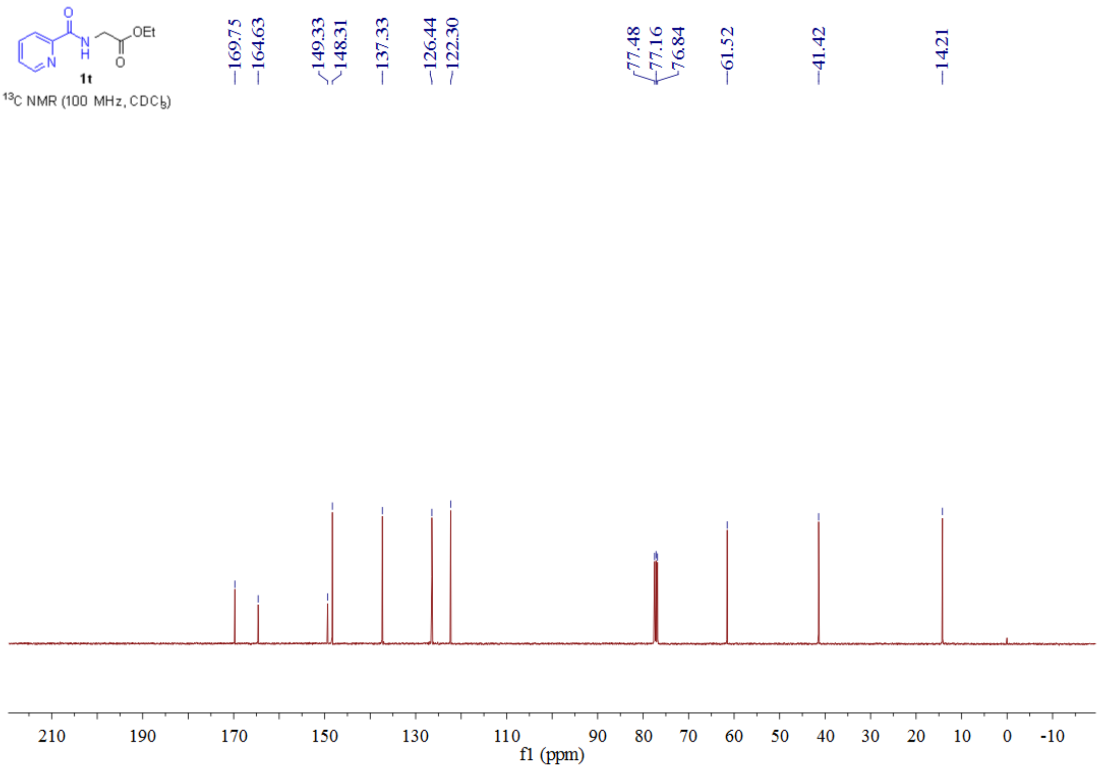

Supplementary Figure 31. ^{1}H NMR and ^{13}C NMR spectra for compound 1t

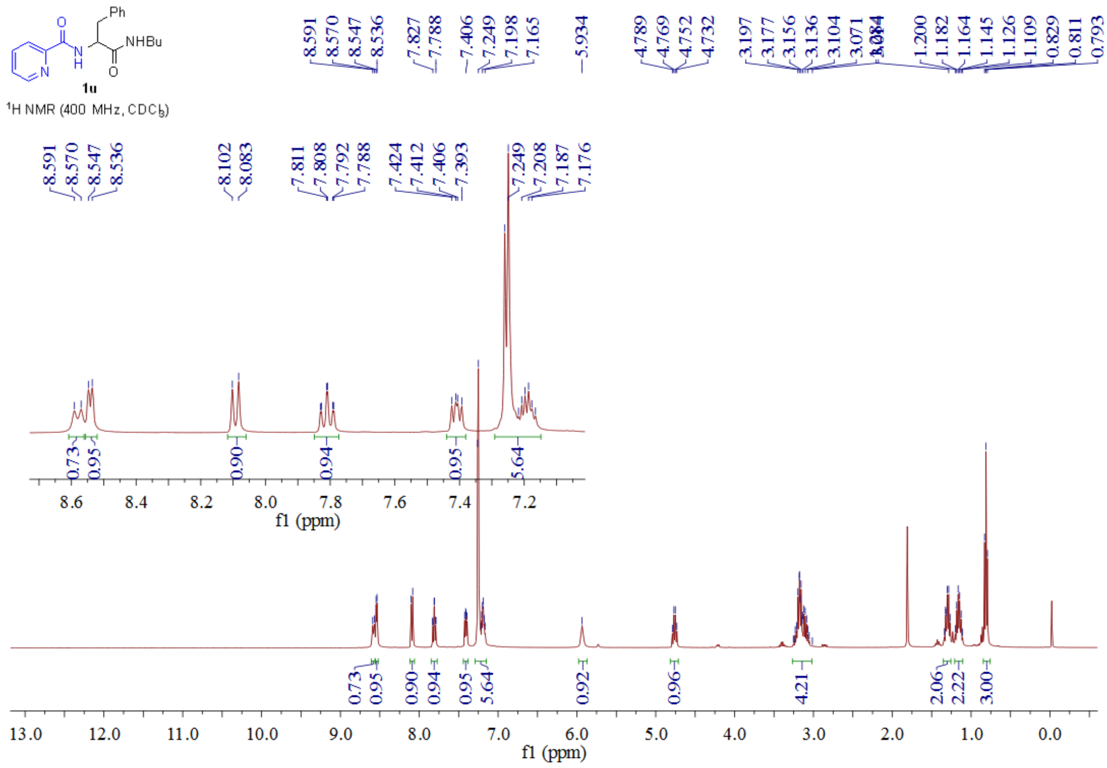

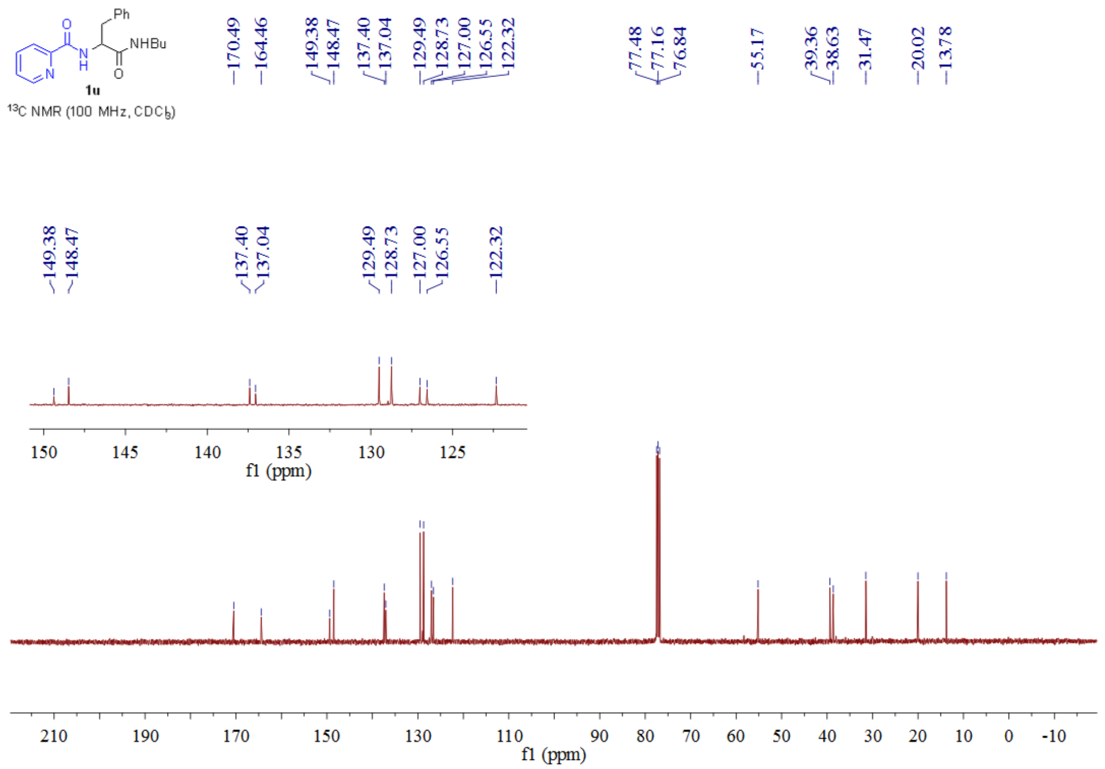

Supplementary Figure 32. ^{1}H NMR and ^{13}C NMR spectra for compound 1u

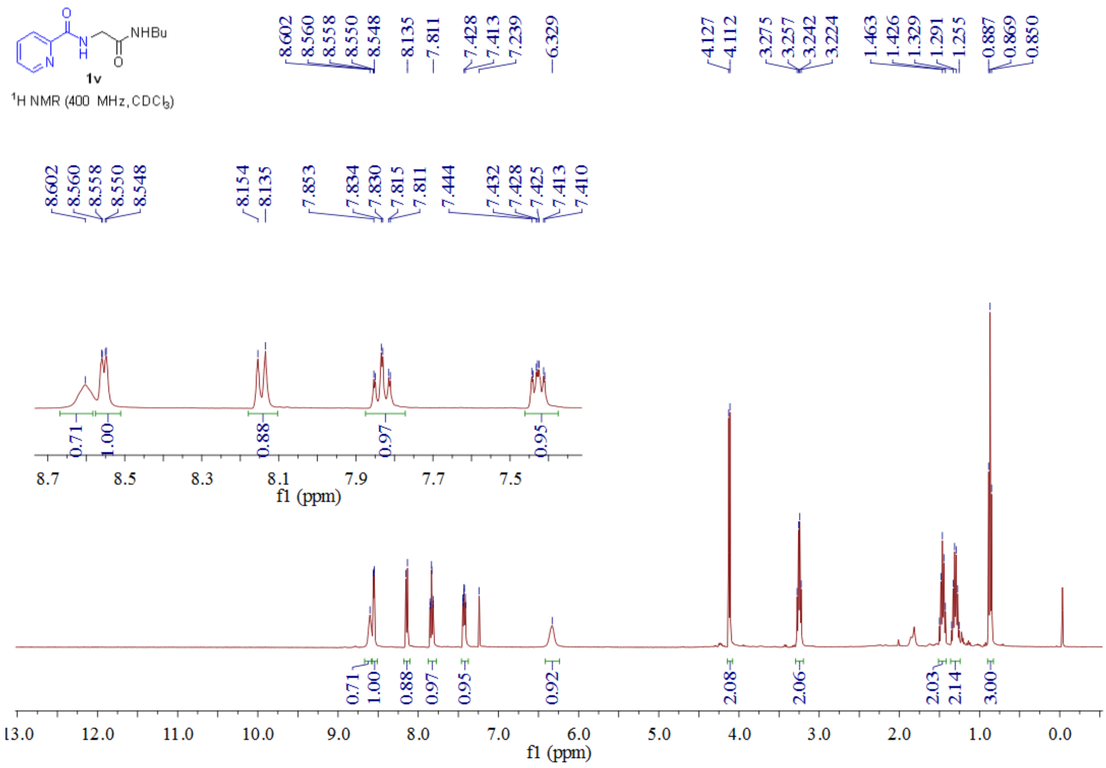

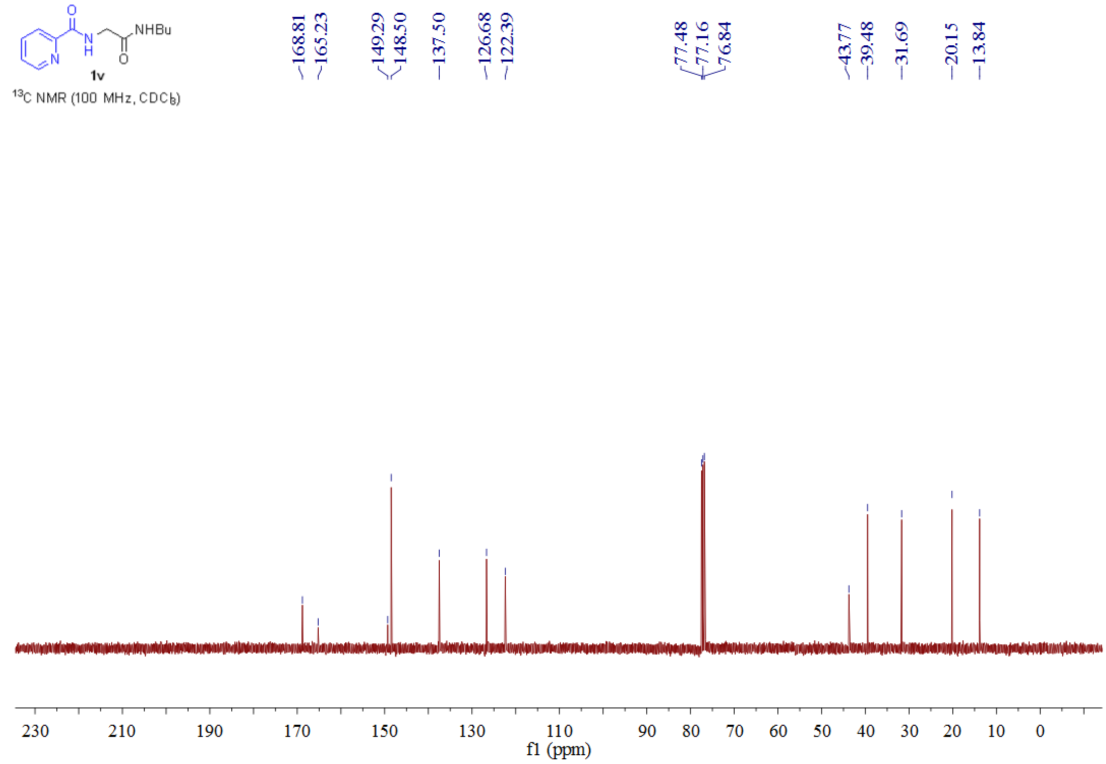

Supplementary Figure 33. ^{1}H NMR and ^{13}C NMR spectra for compound 1v

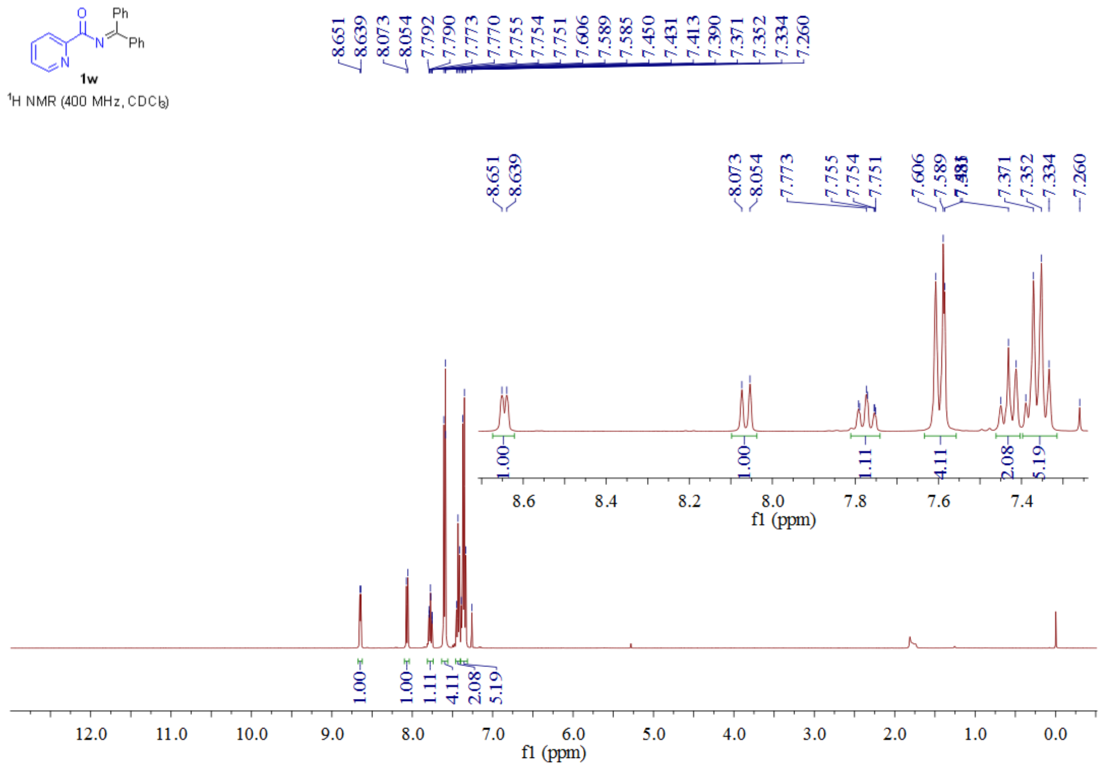

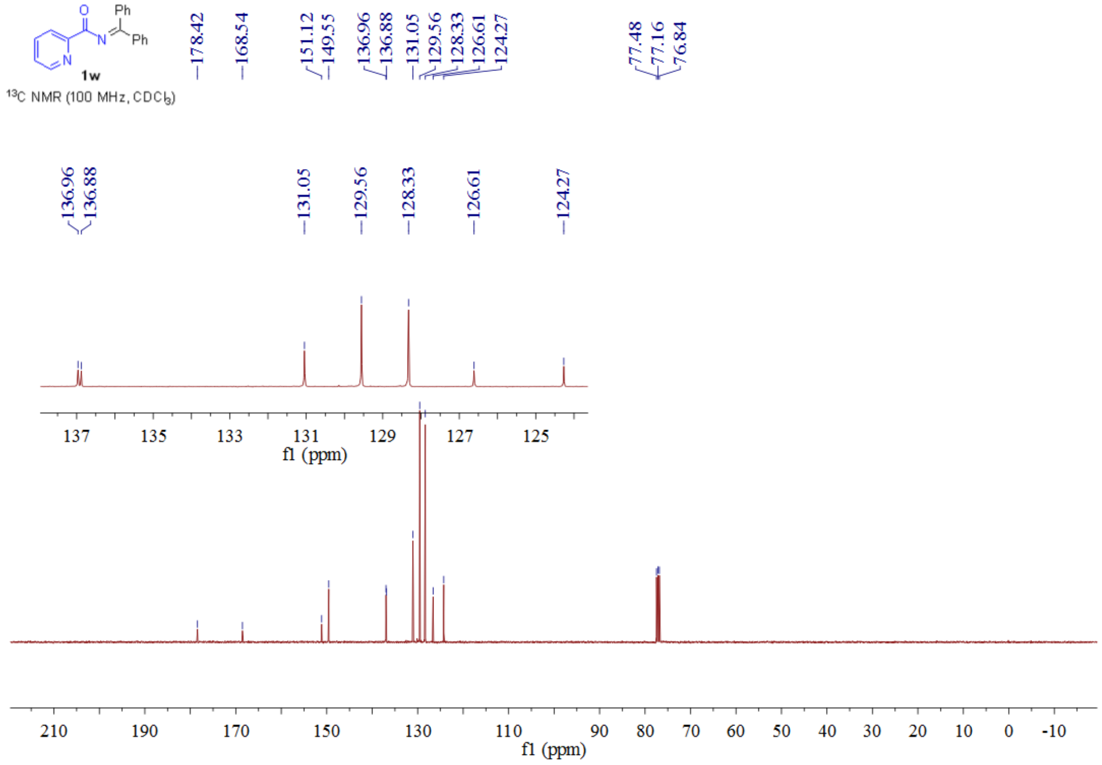

Supplementary Figure 34. ^{1}H NMR and ^{13}C NMR spectra for compound 1w

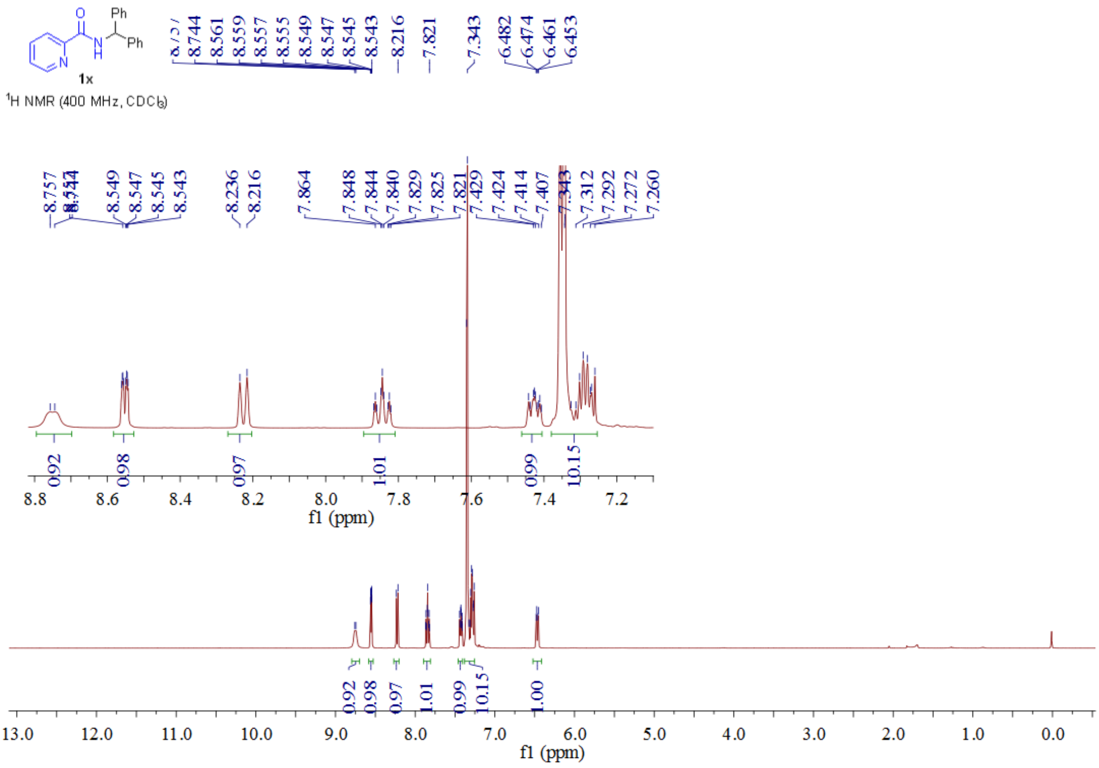

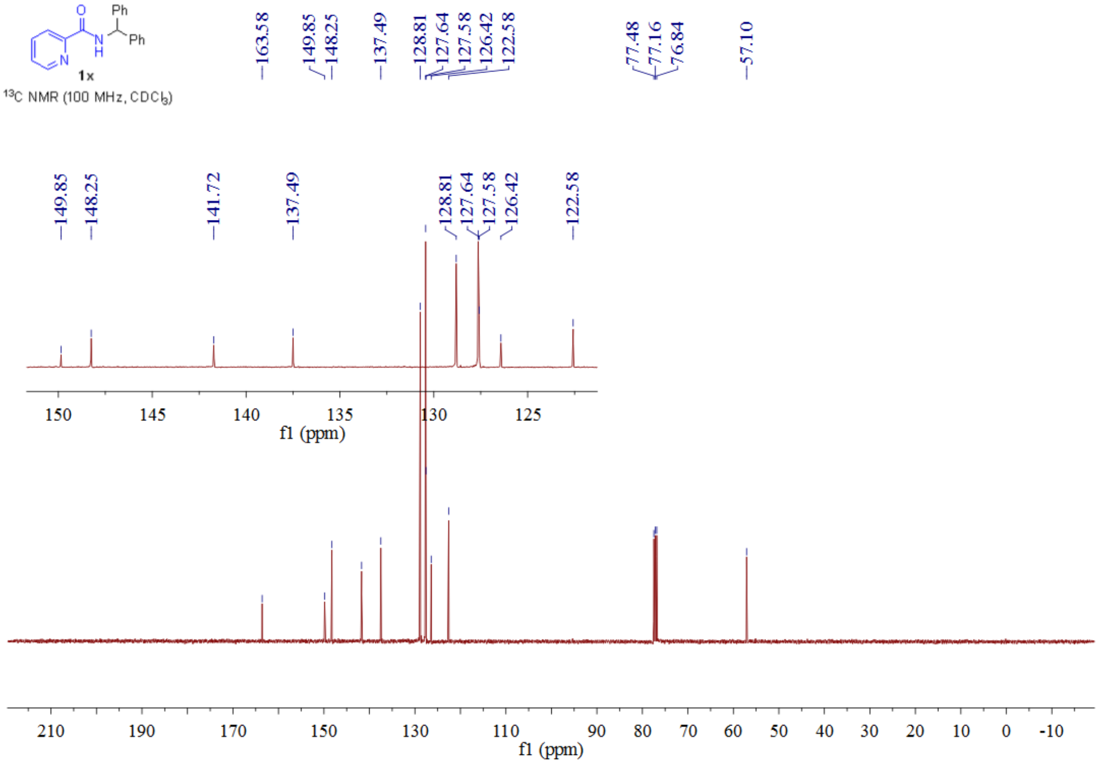

Supplementary Figure 35. ^{1}**H NMR and** ^{13}**C NMR spectra for compound 1x**

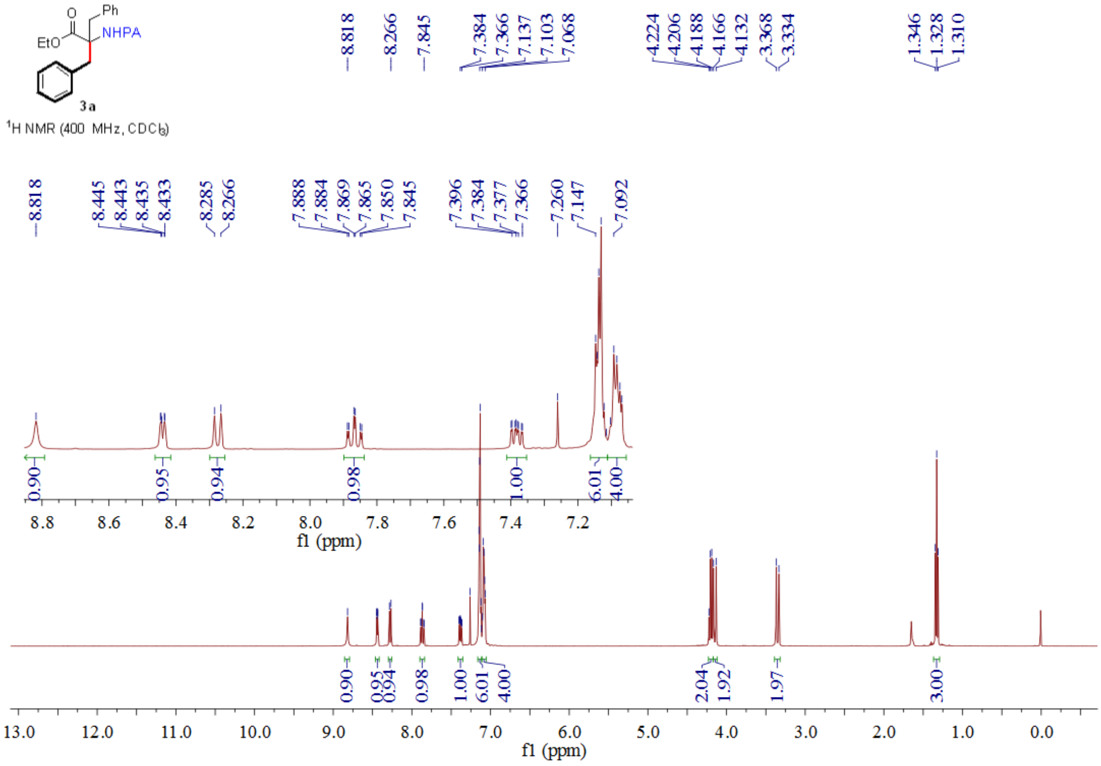

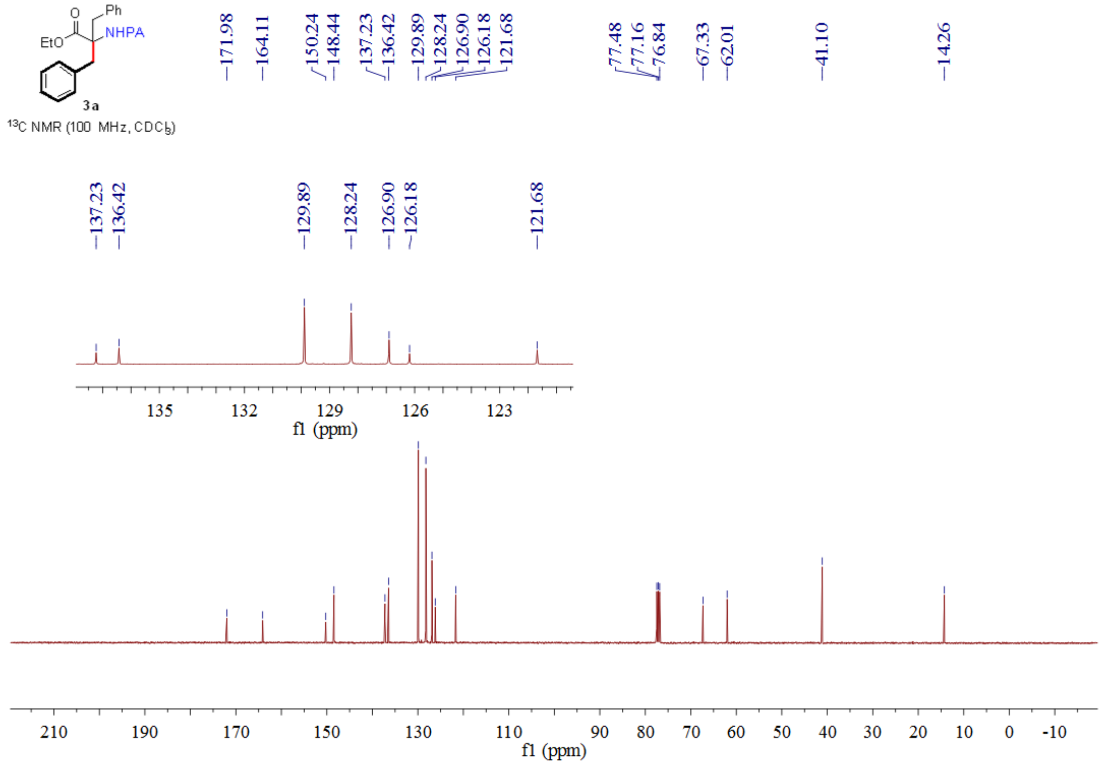

Supplementary Figure 36. ^{1}H NMR and ^{13}C NMR spectra for compound 3a

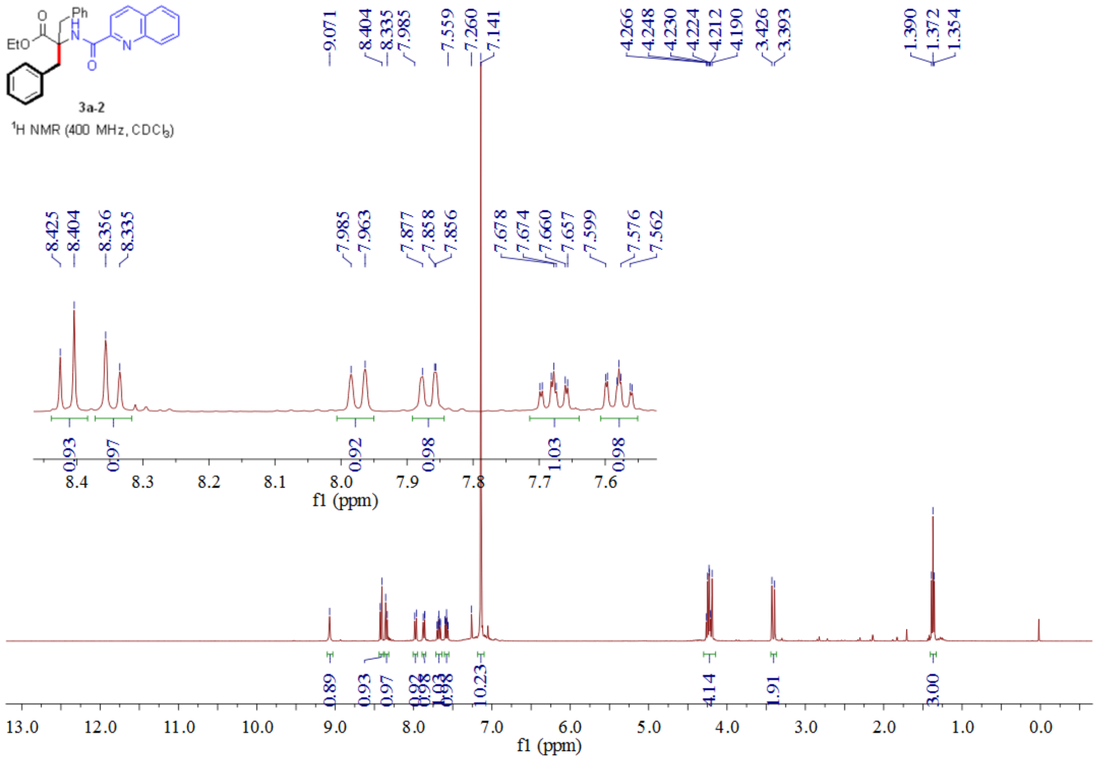

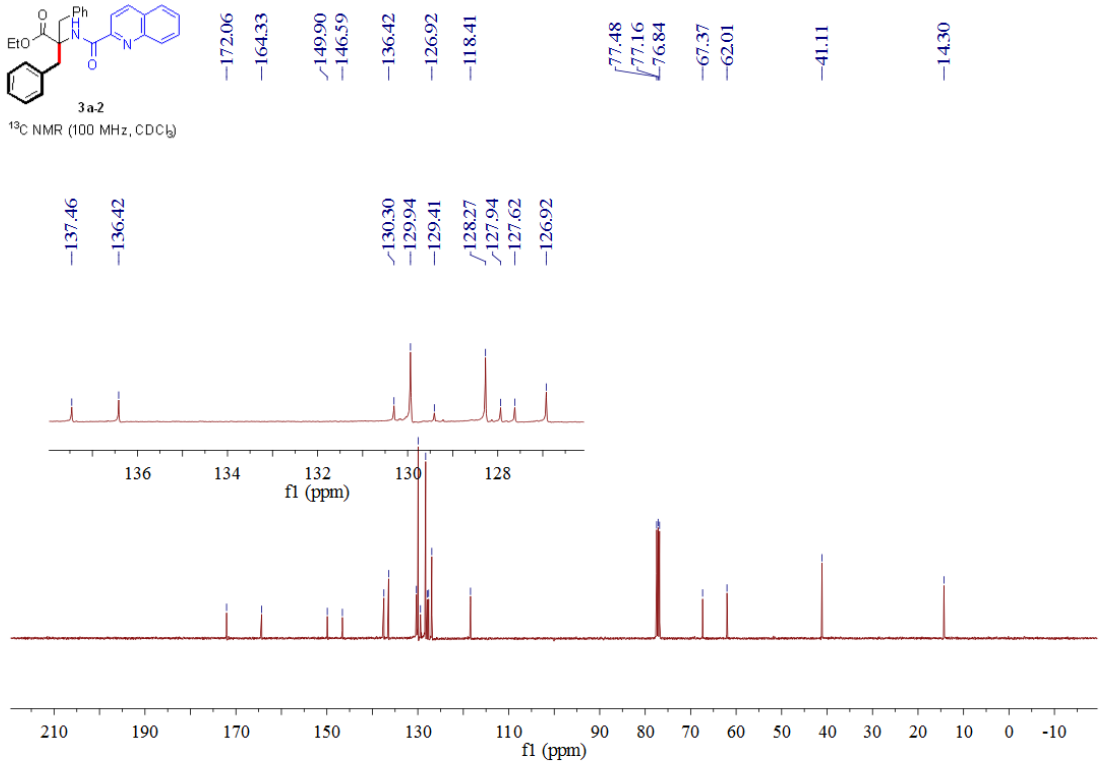

Supplementary Figure 37. ^{1}**H NMR and** ^{13}**C NMR spectra for compound 3a-2**

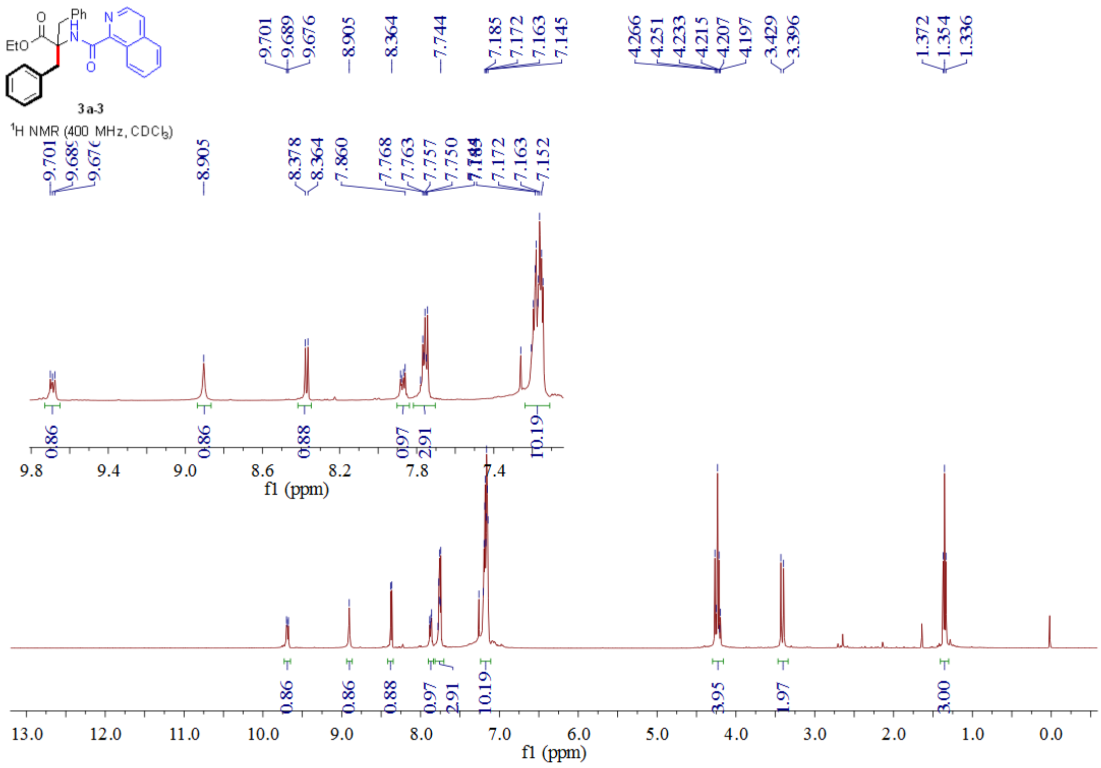

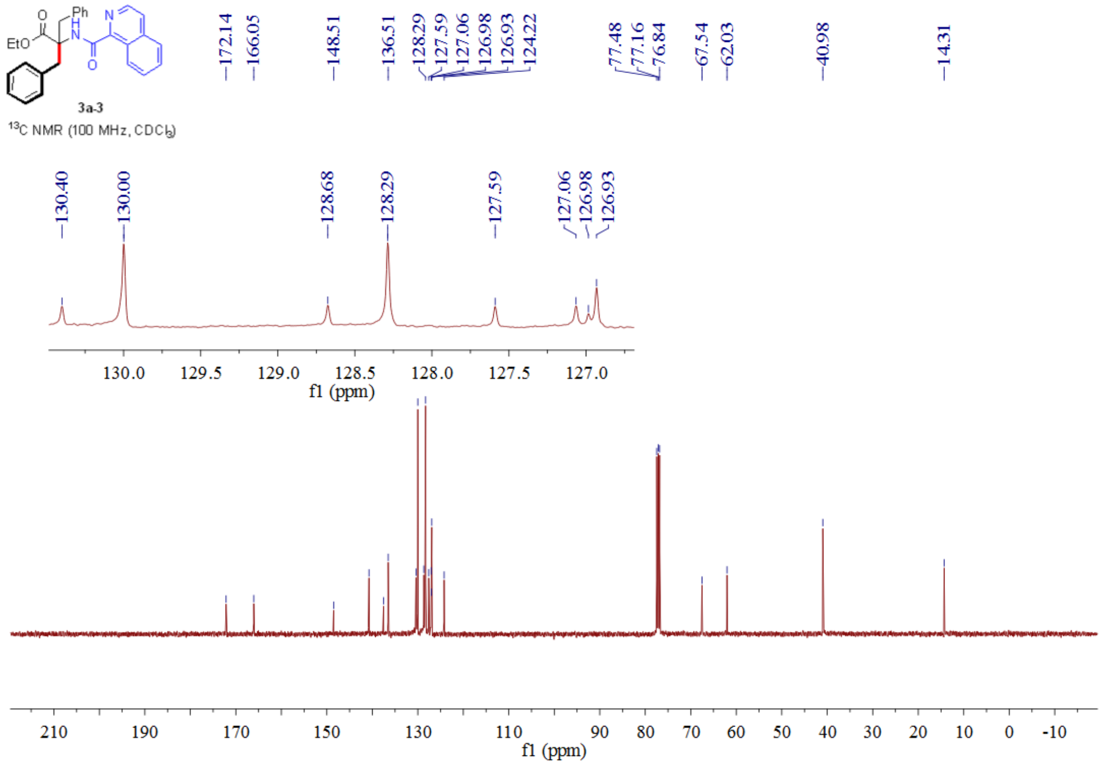

Supplementary Figure 38. ^{1}H NMR and ^{13}C NMR spectra for compound 3a-3

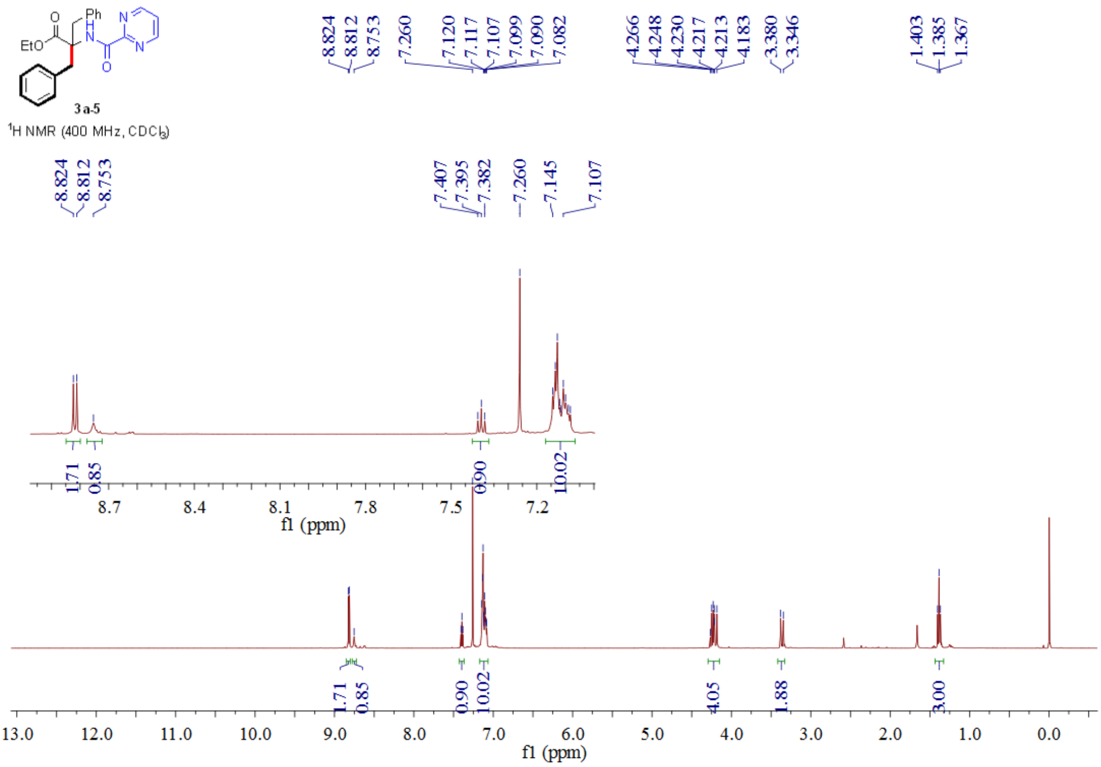

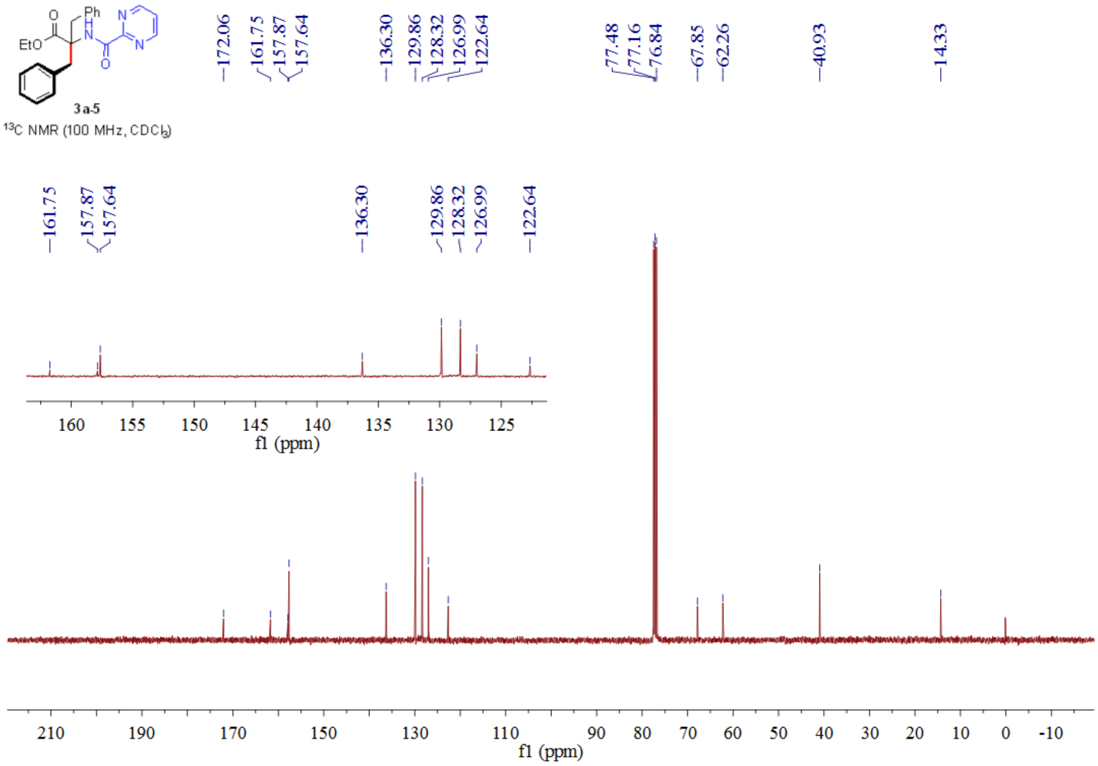

Supplementary Figure 39. ^{1}H NMR and ^{13}C NMR spectra for compound 3a-5

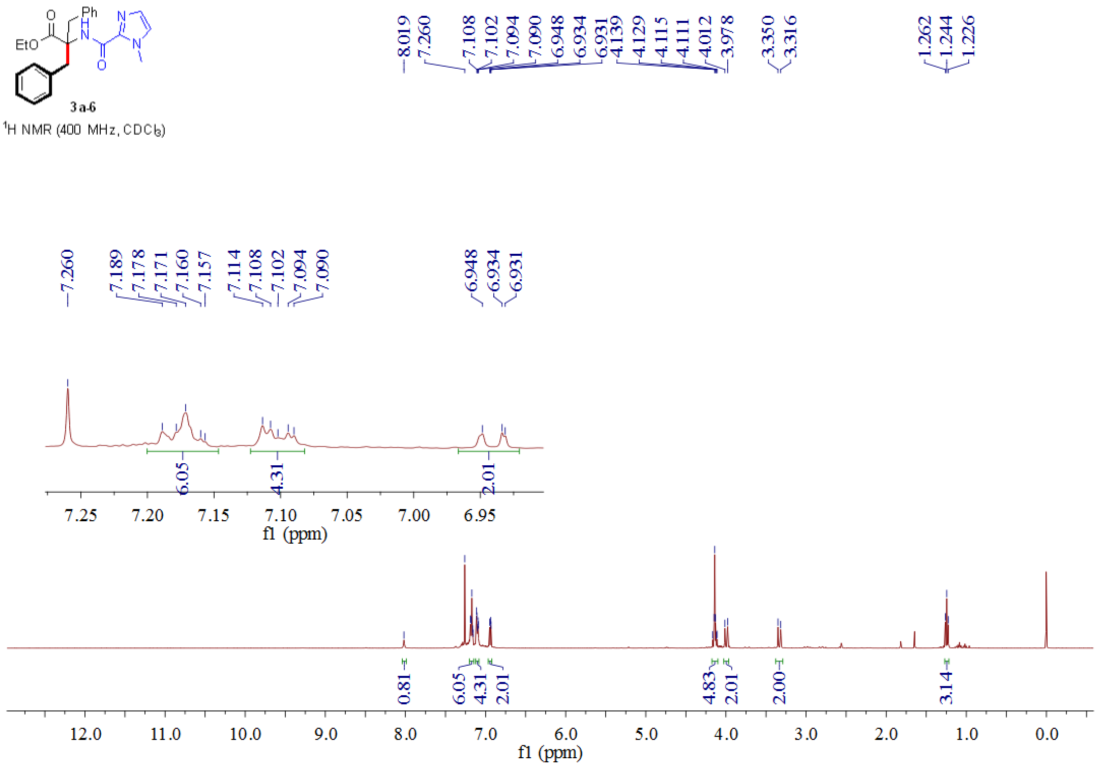

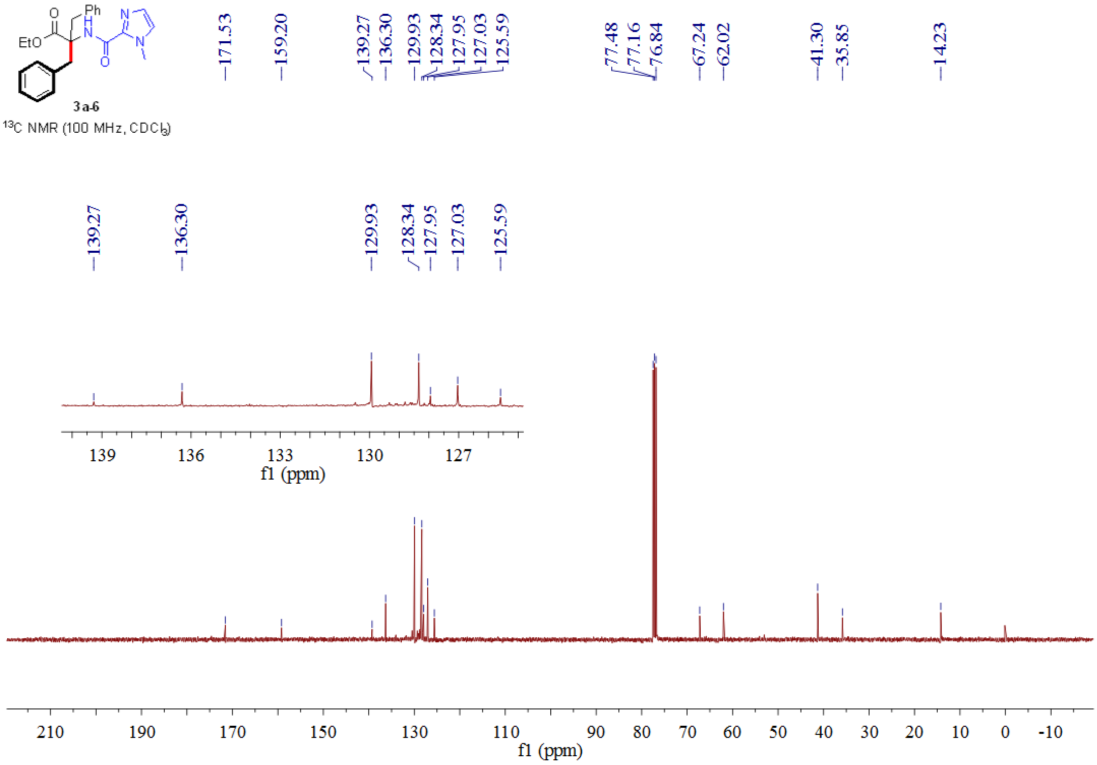

Supplementary Figure 40. ^{1}H NMR and ^{13}C NMR spectra for compound 3a-6

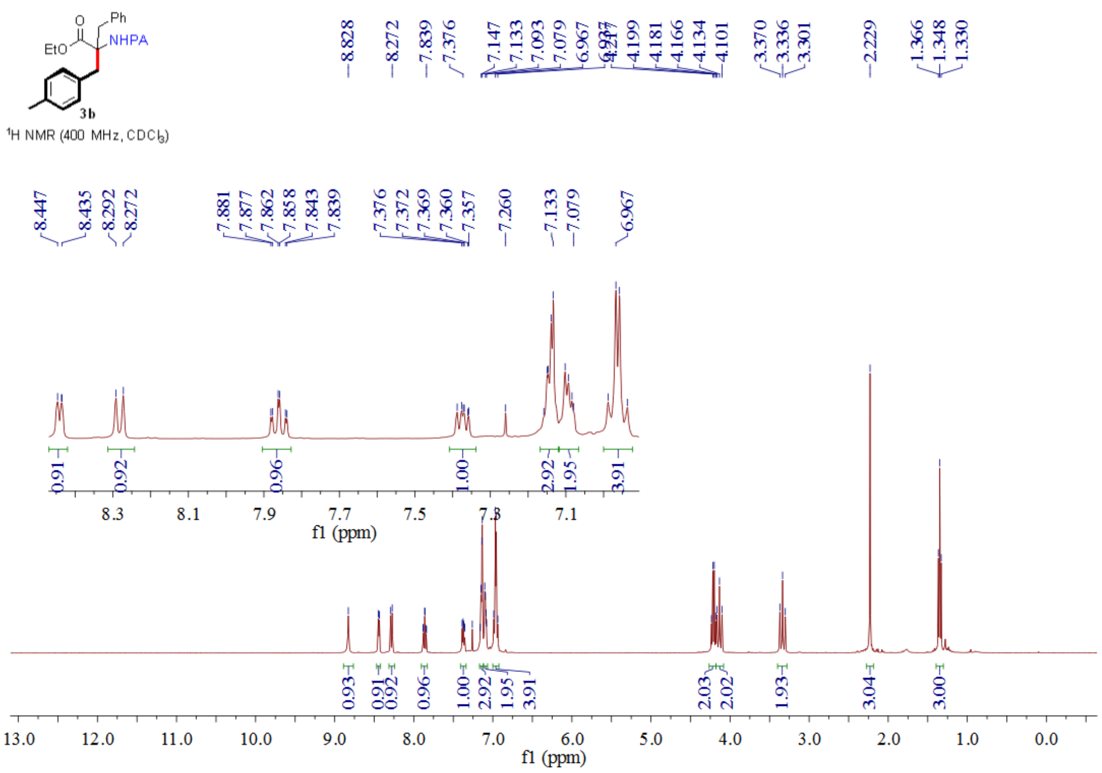

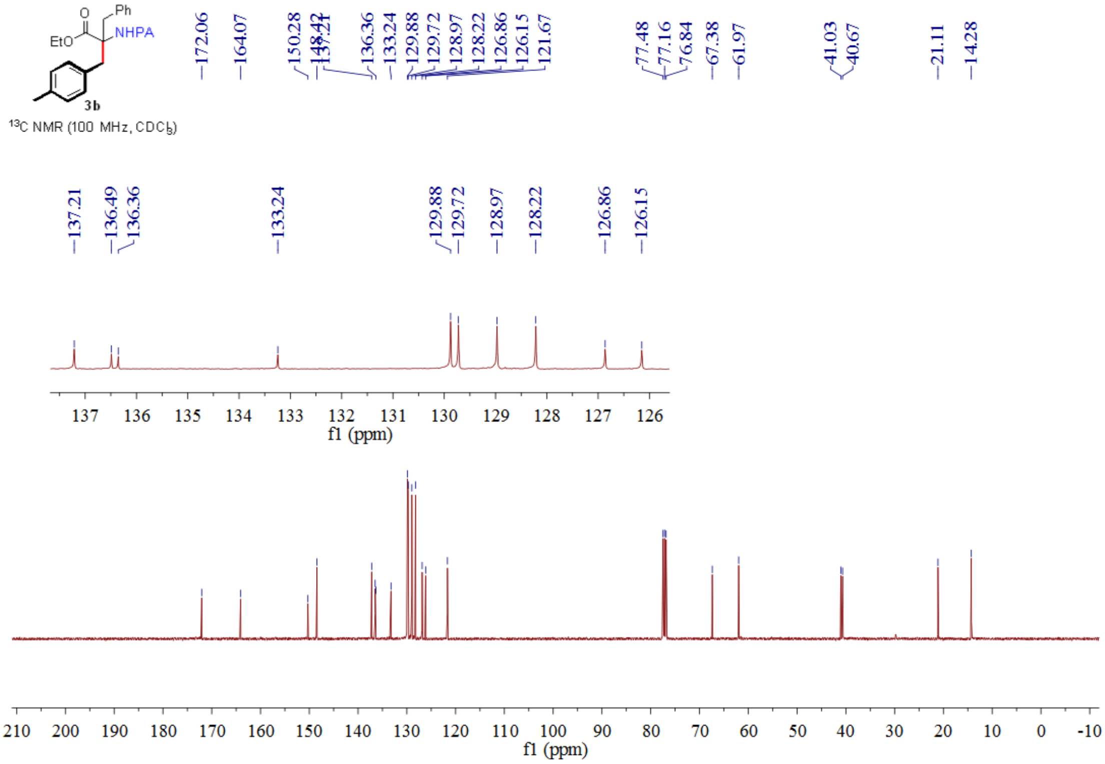

Supplementary Figure 41. ^{1}H NMR and ^{13}C NMR spectra for compound 3b

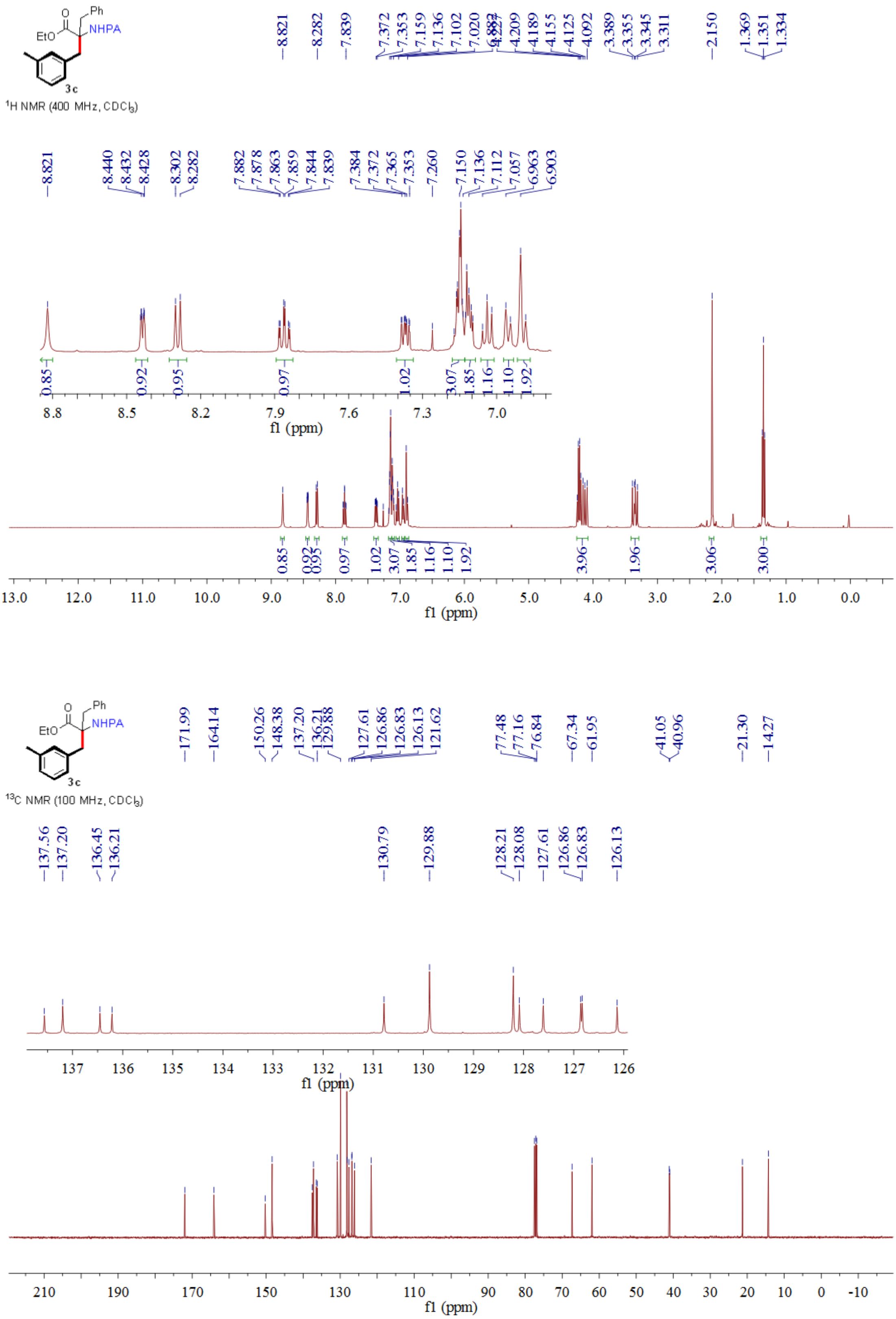

Supplementary Figure 42. ^{1}H NMR and ^{13}C NMR spectra for compound 3c

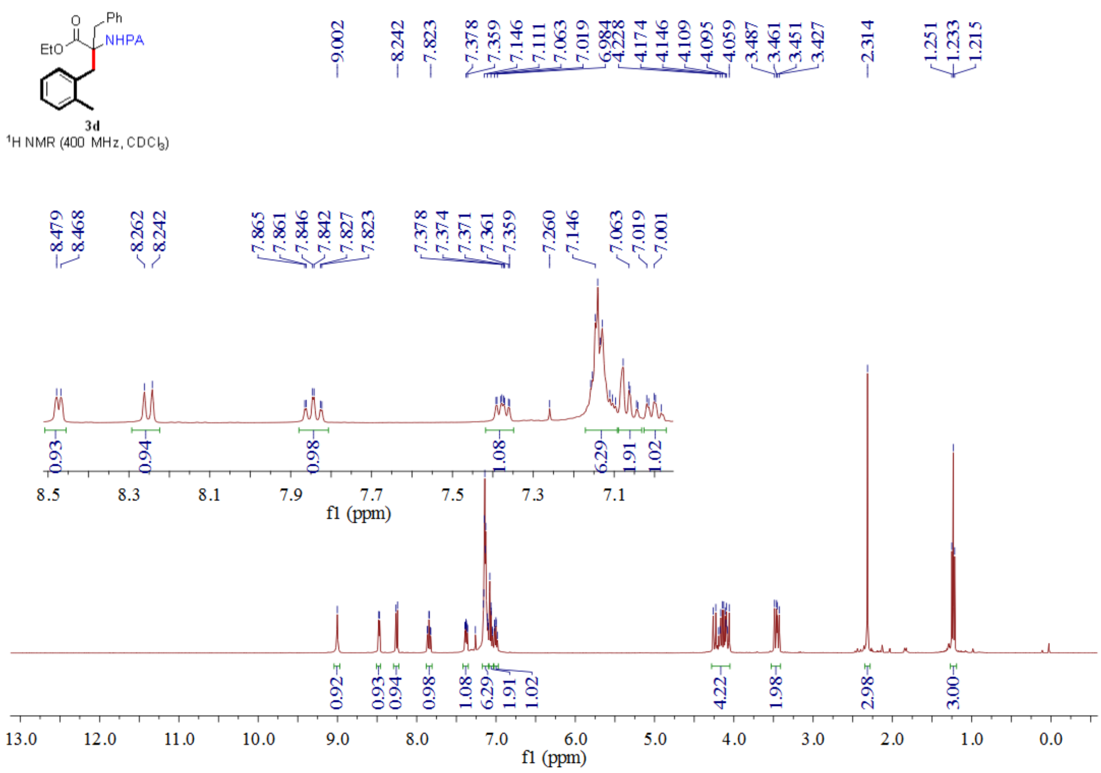

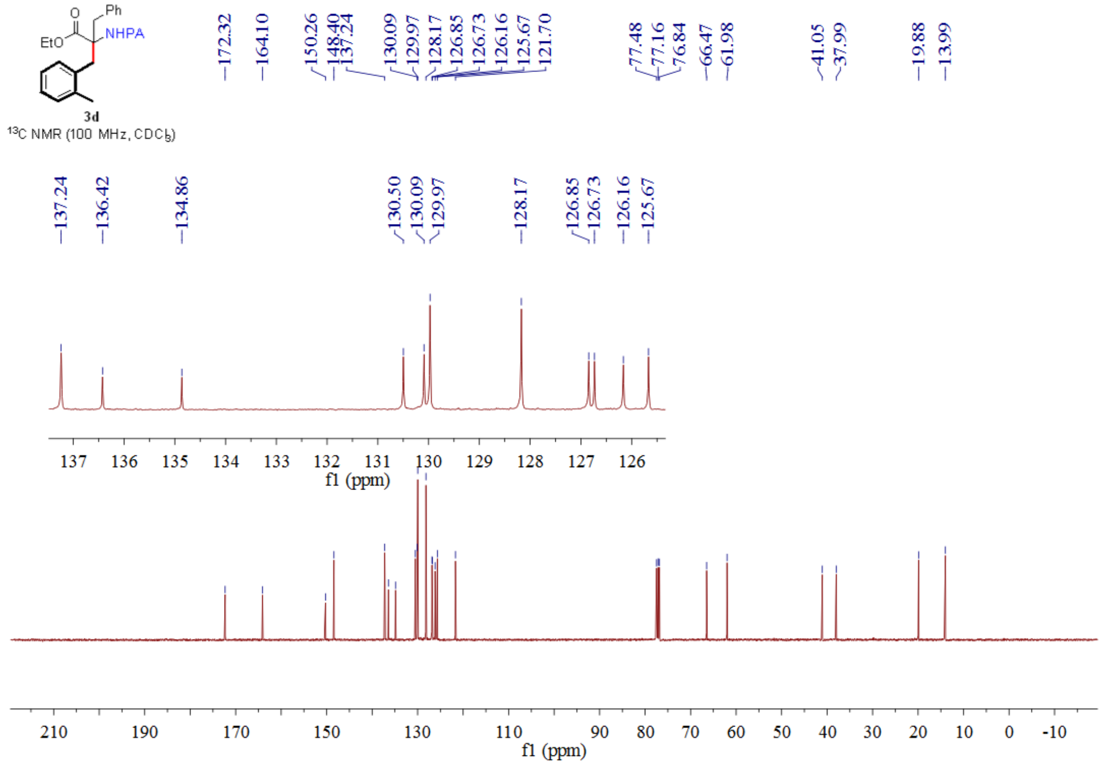

Supplementary Figure 43. ^{1}H NMR and ^{13}C NMR spectra for compound 3d

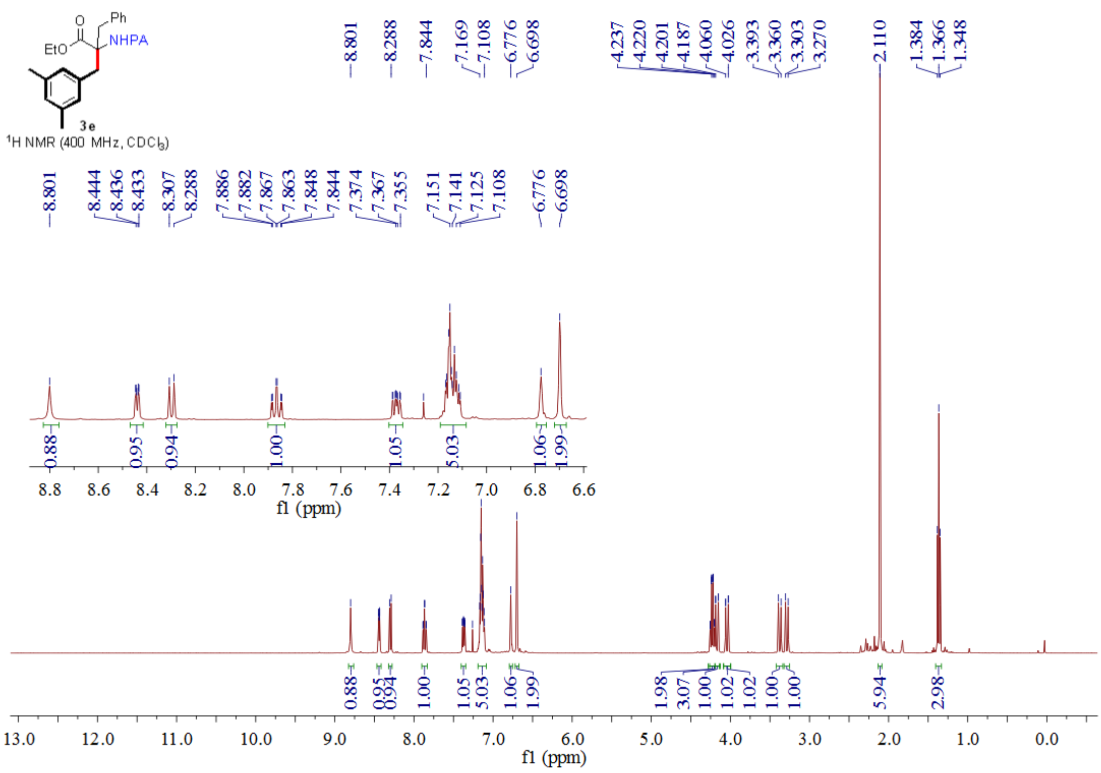

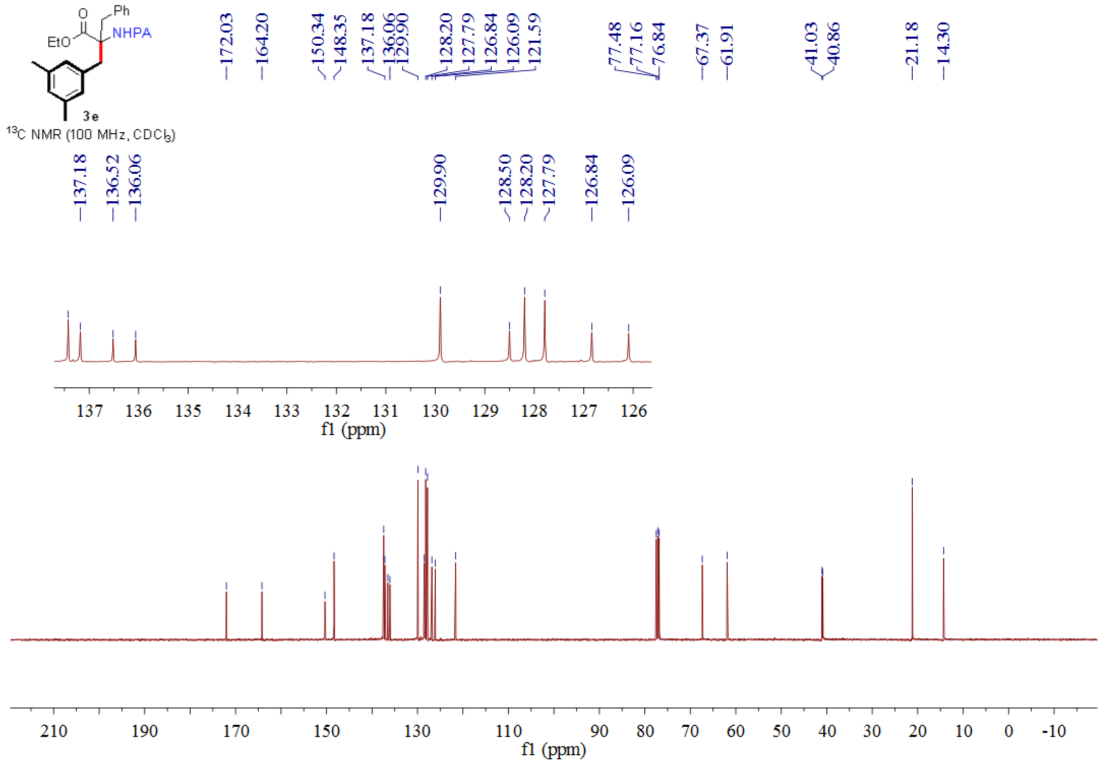

Supplementary Figure 44. ^{1}H NMR and ^{13}C NMR spectra for compound 3e

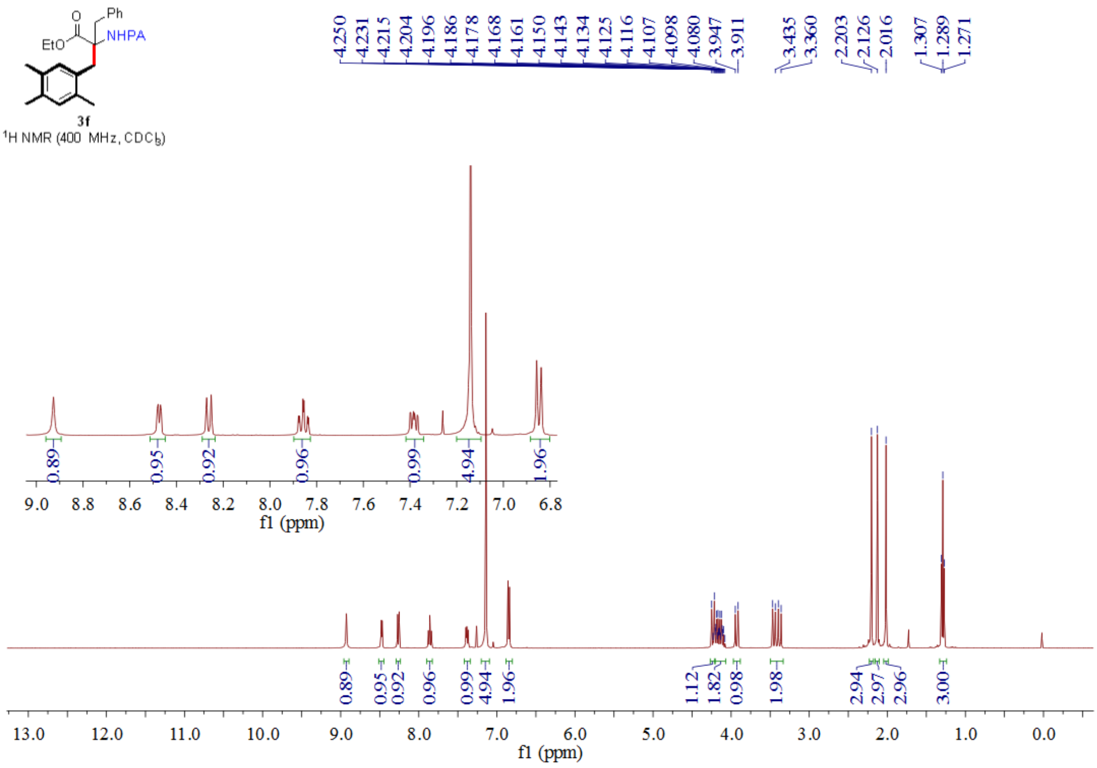

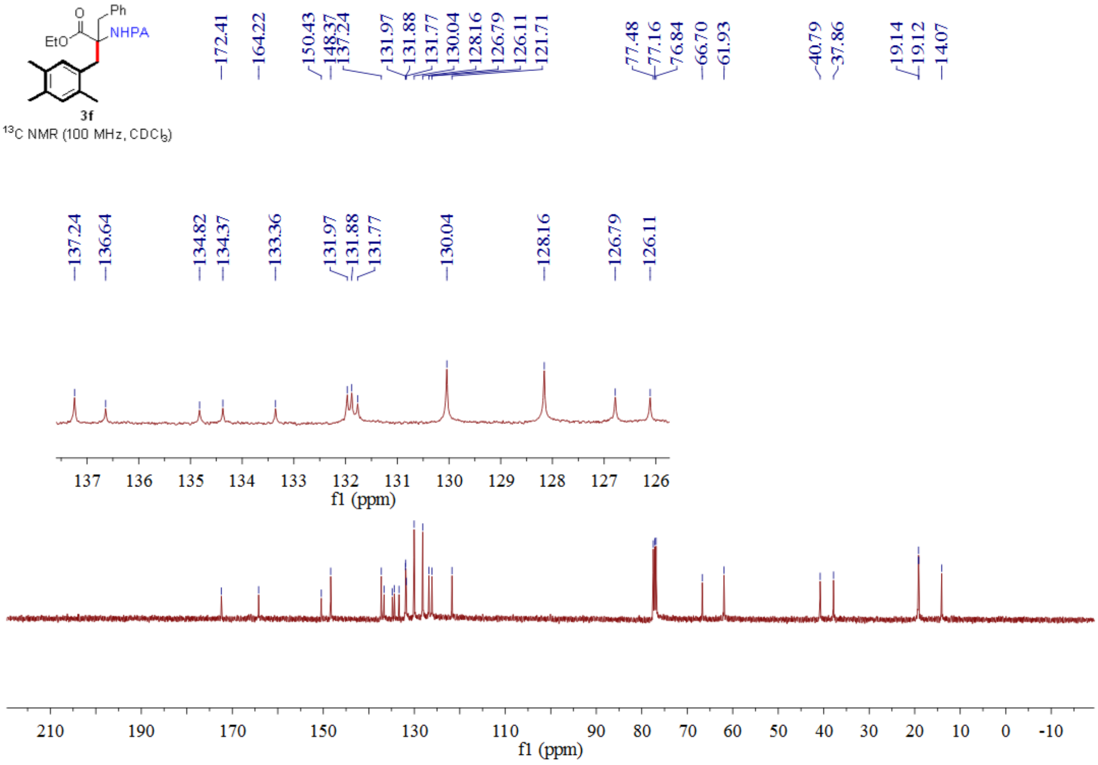

Supplementary Figure 45. ^{1}H NMR and ^{13}C NMR spectra for compound 3f

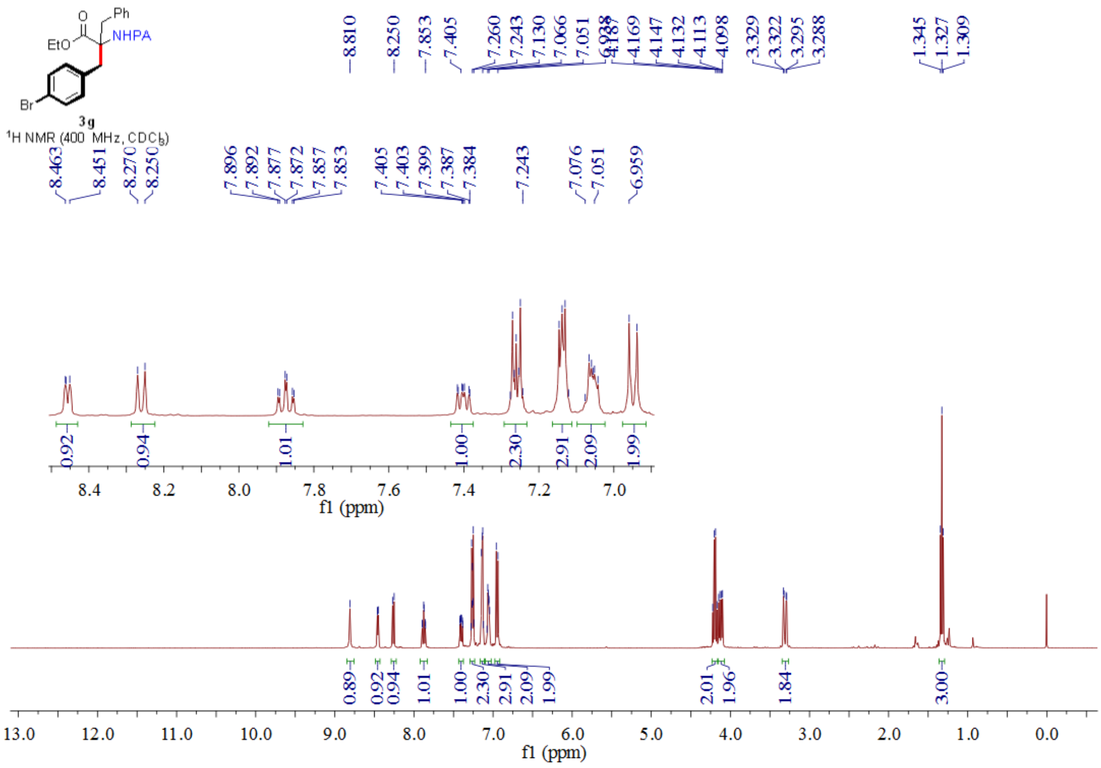

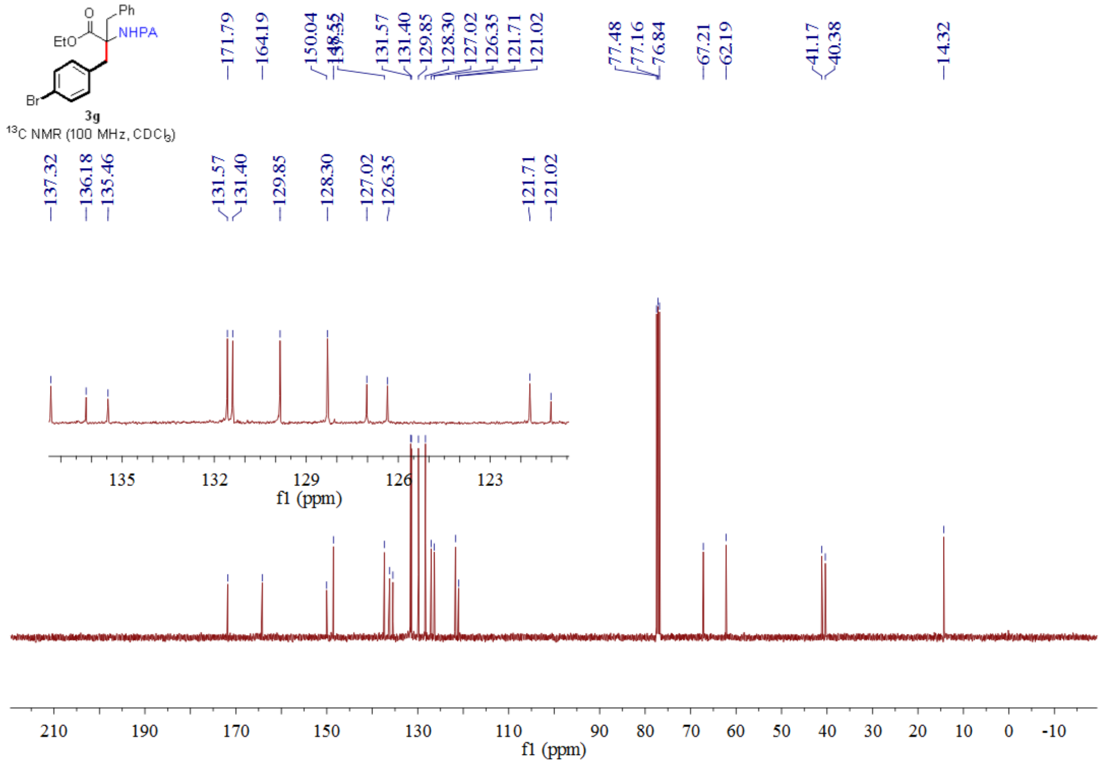

Supplementary Figure 46. ^{1}**H NMR and** ^{13}**C NMR spectra for compound 3g**

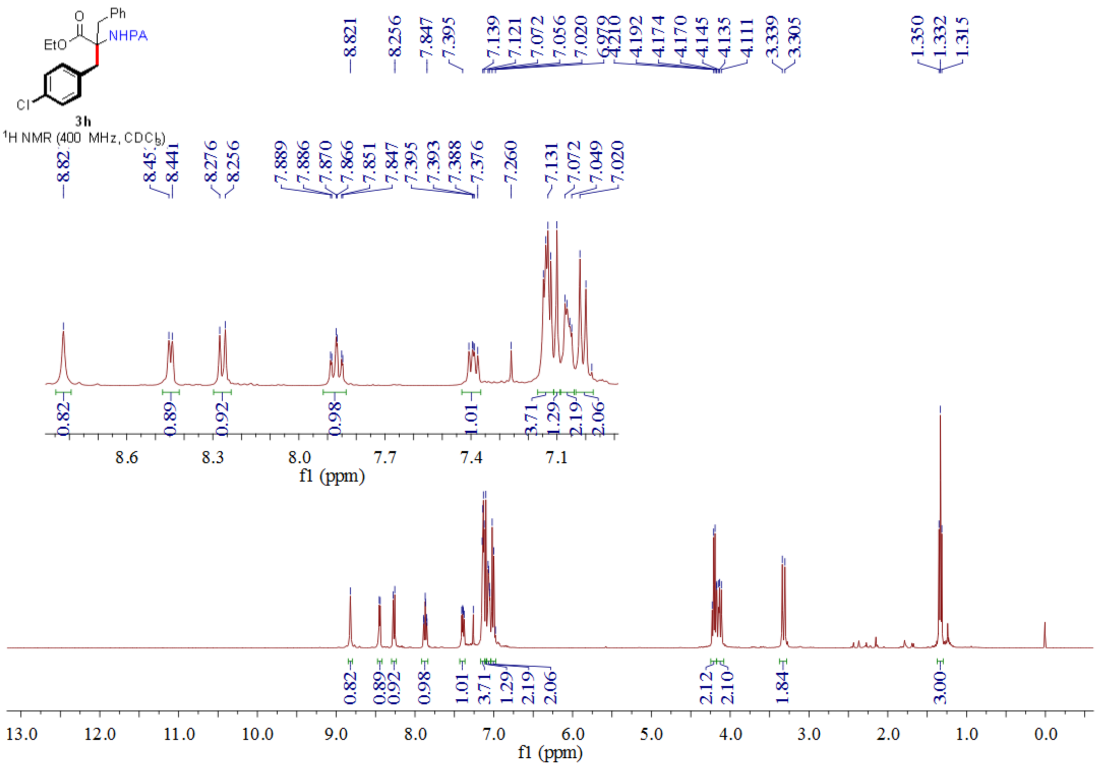

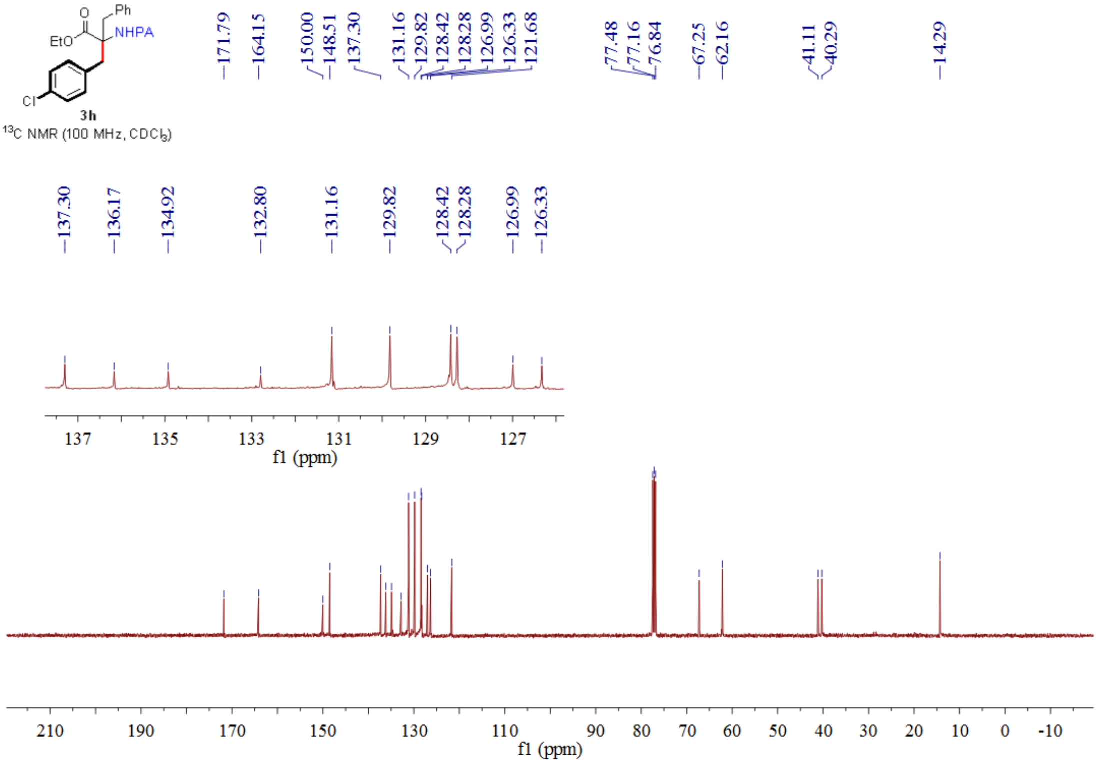

Supplementary Figure 47. ^{1}H NMR and ^{13}C NMR spectra for compound 3h

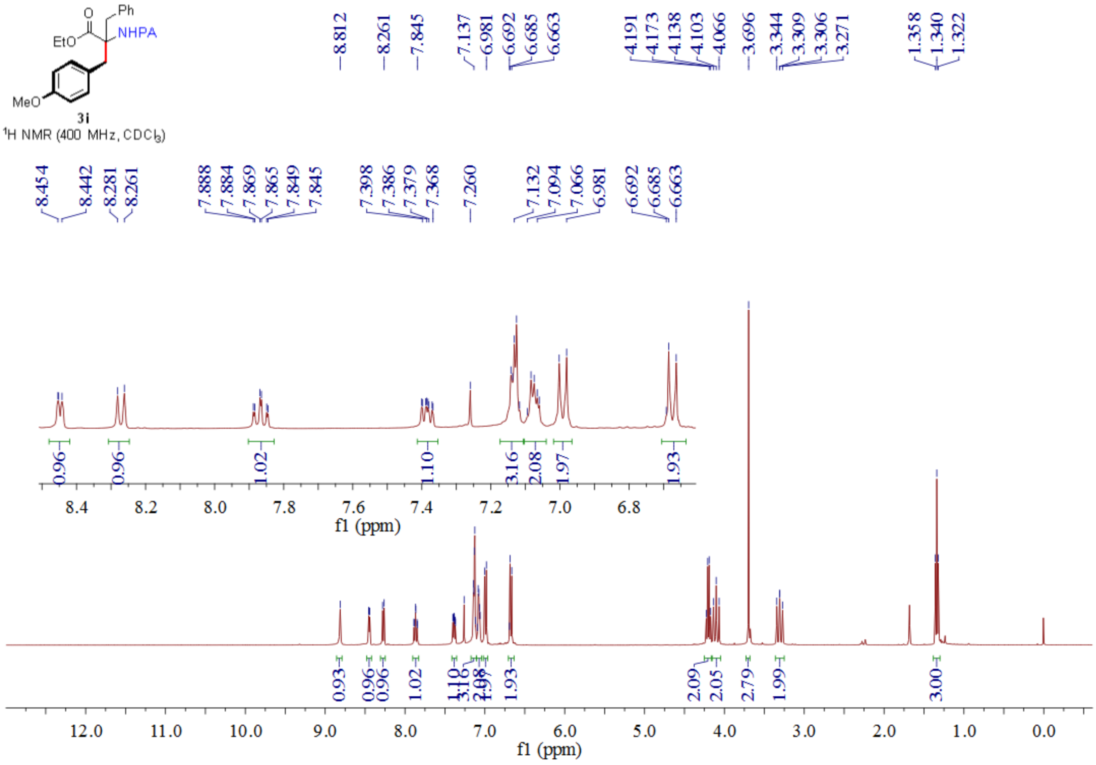

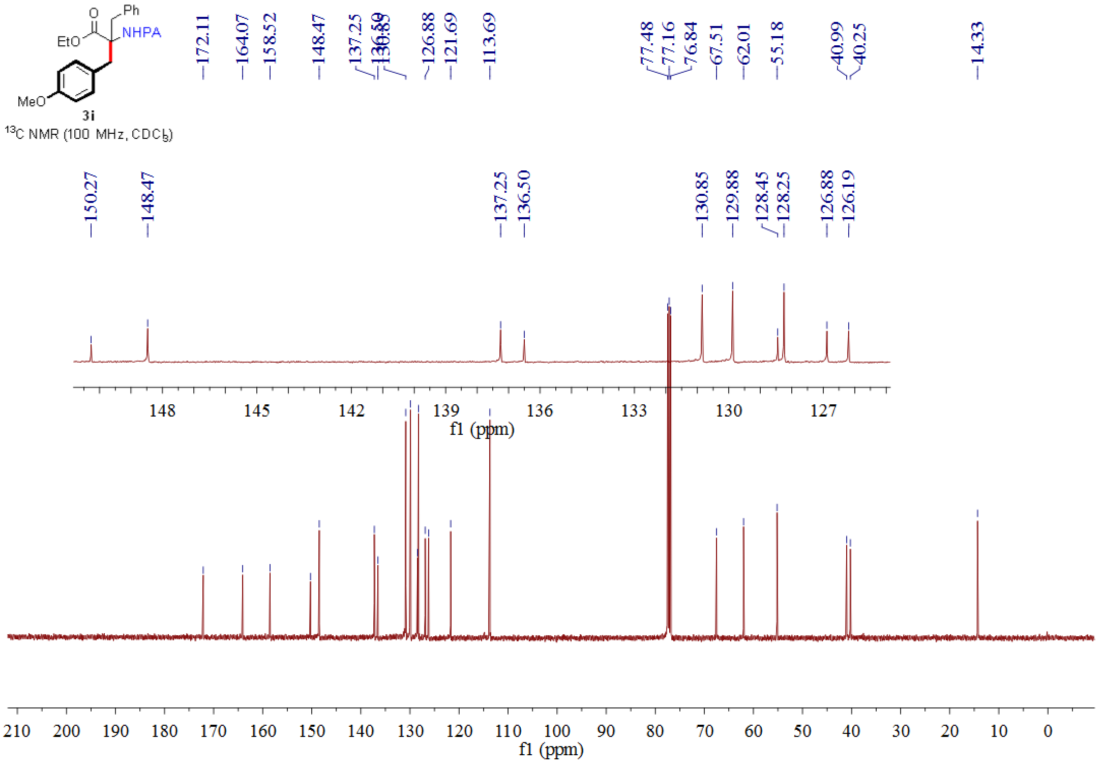

Supplementary Figure 48. ^{1}**H NMR and** ^{13}**C NMR spectra for compound 3i**

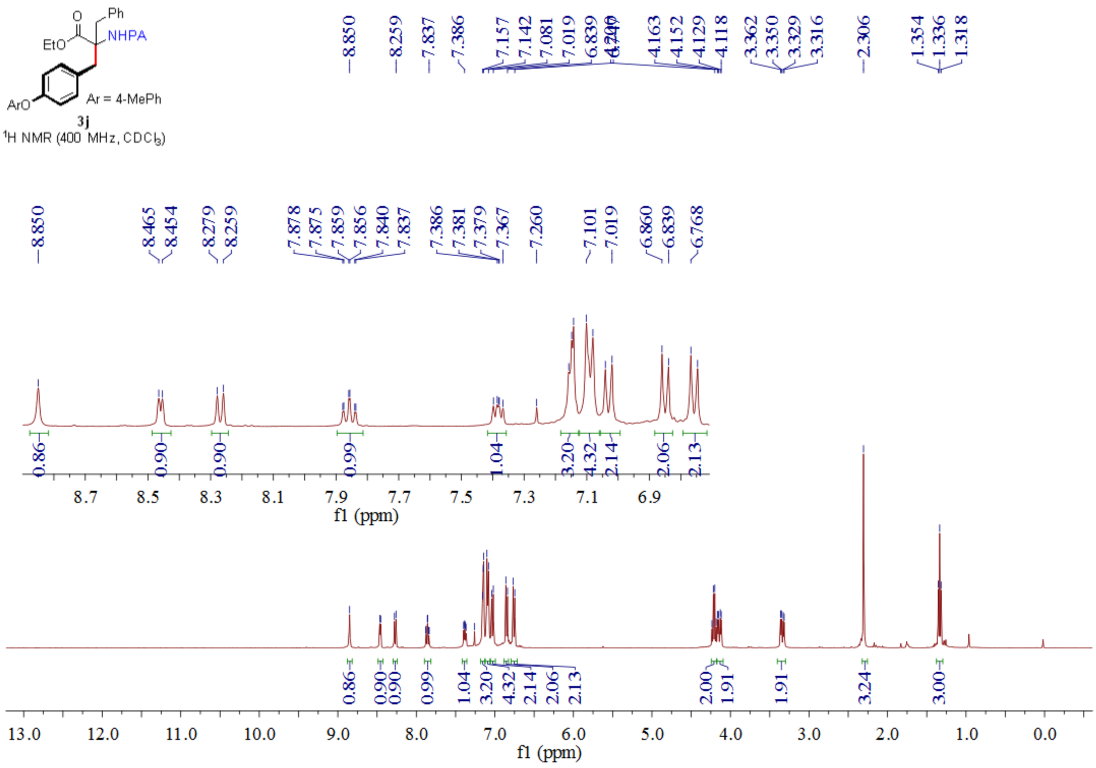

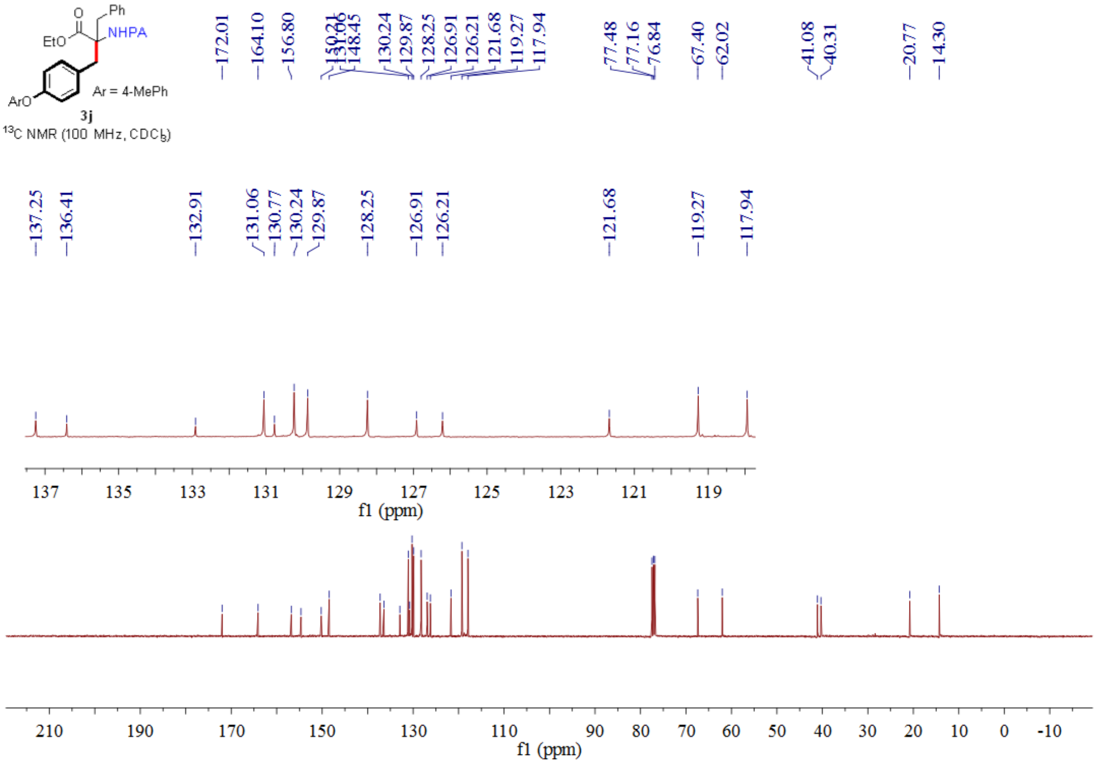

Supplementary Figure 49. ^{1}H NMR and ^{13}C NMR spectra for compound 3j

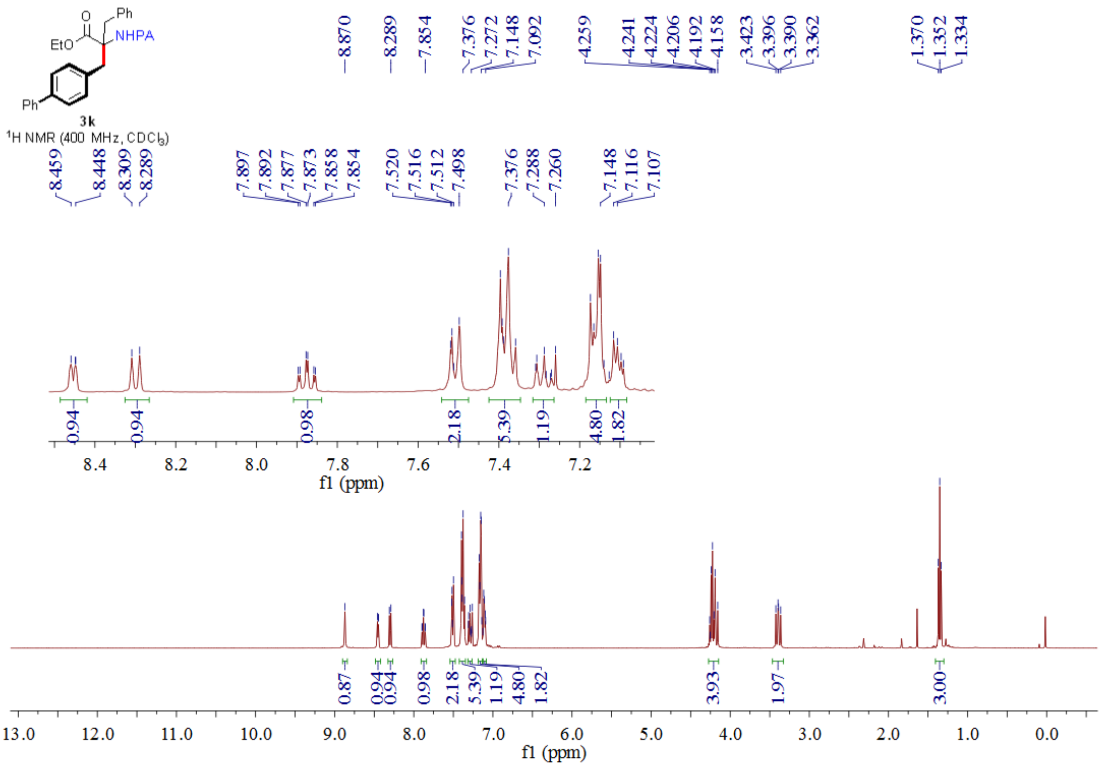

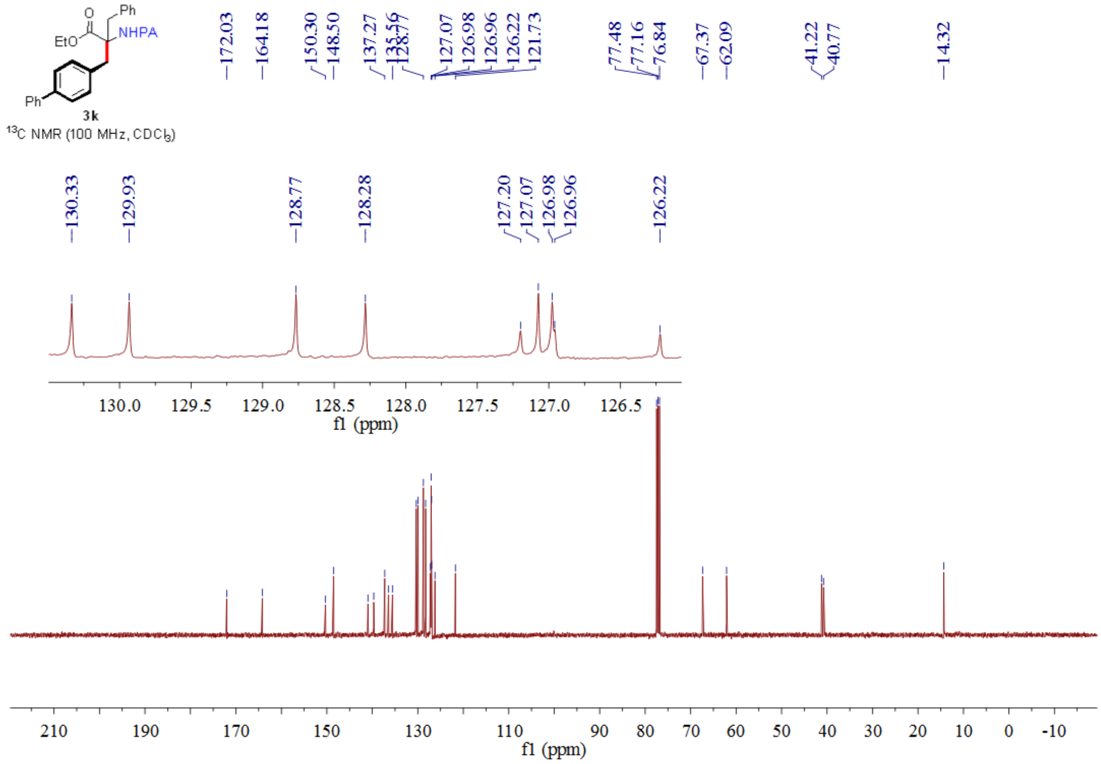

Supplementary Figure 50. ^{1}**H NMR and** ^{13}**C NMR spectra for compound 3k**

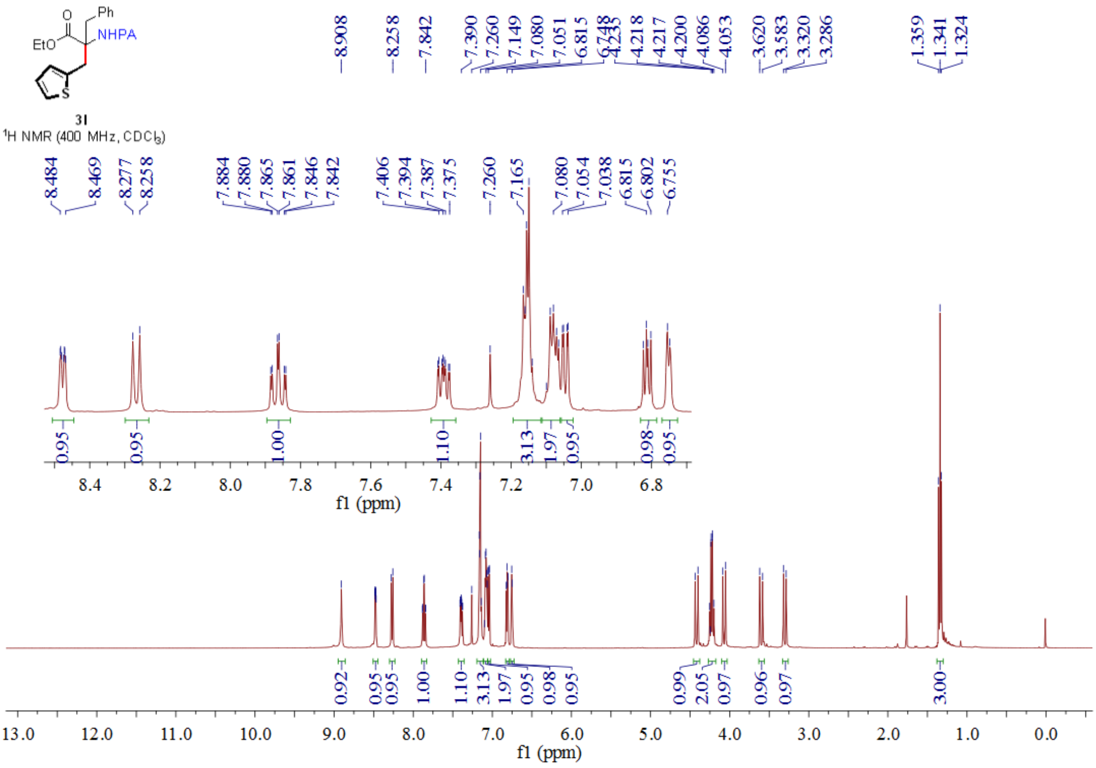

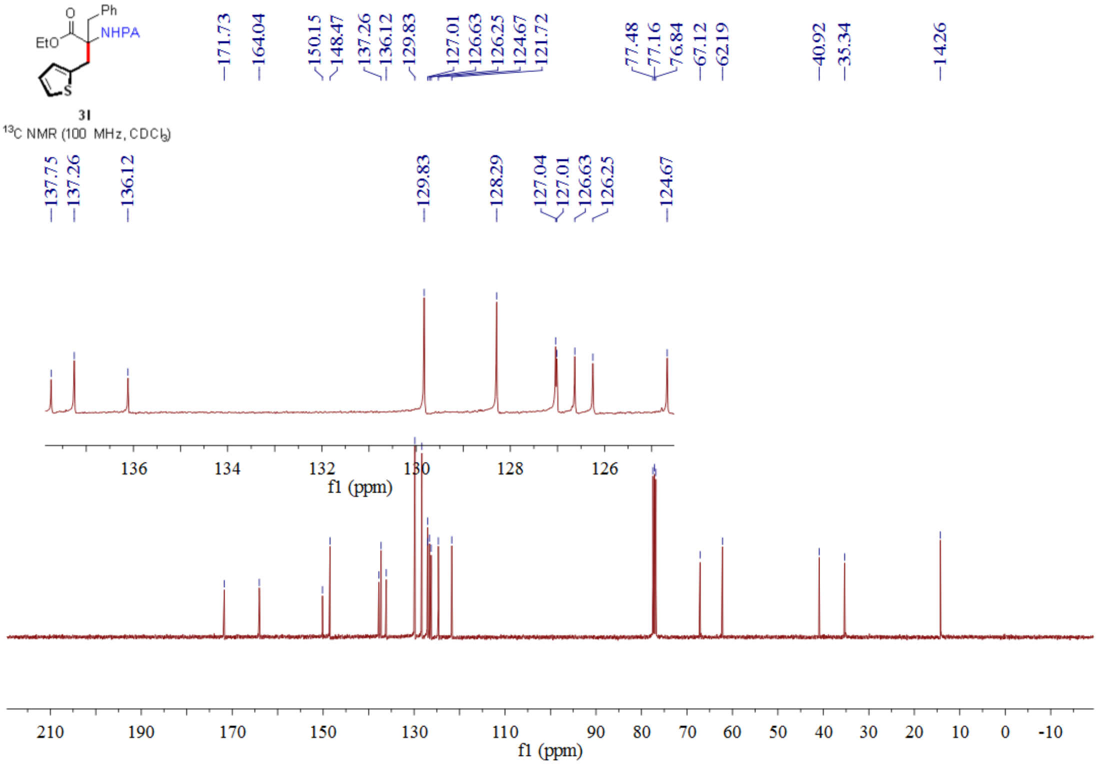

Supplementary Figure 51. ^{1}H NMR and ^{13}C NMR spectra for compound 3l

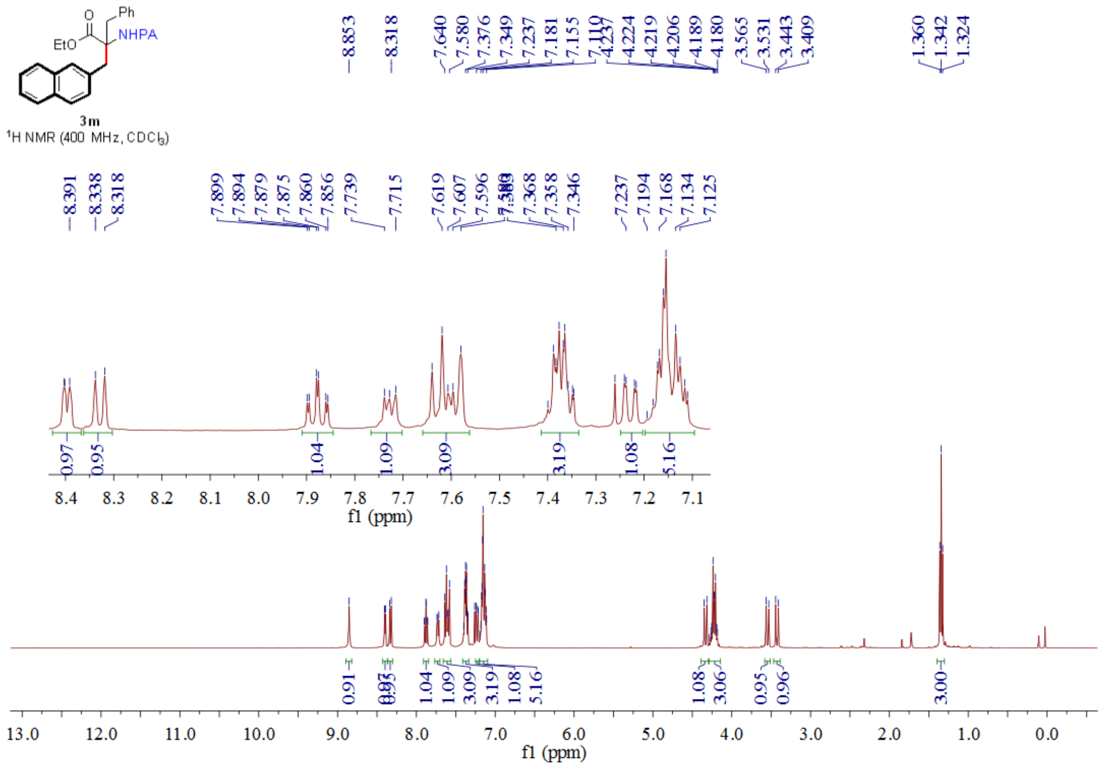

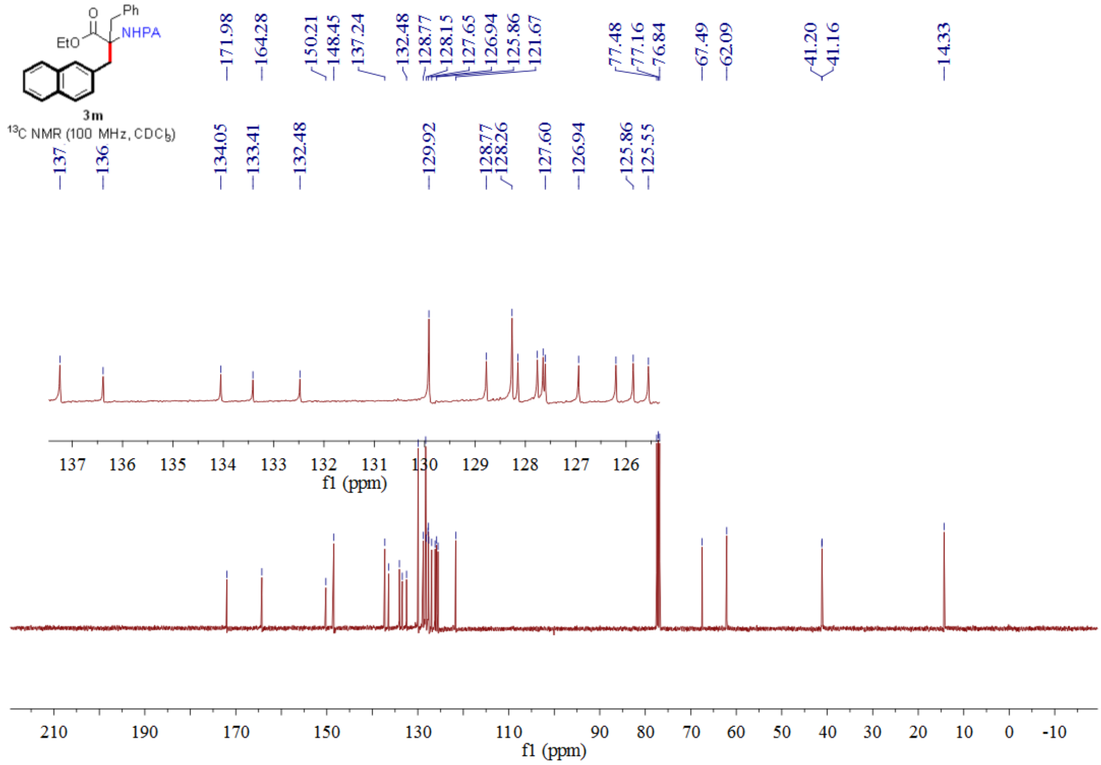

Supplementary Figure 52. ^{1}H NMR and ^{13}C NMR spectra for compound 3m

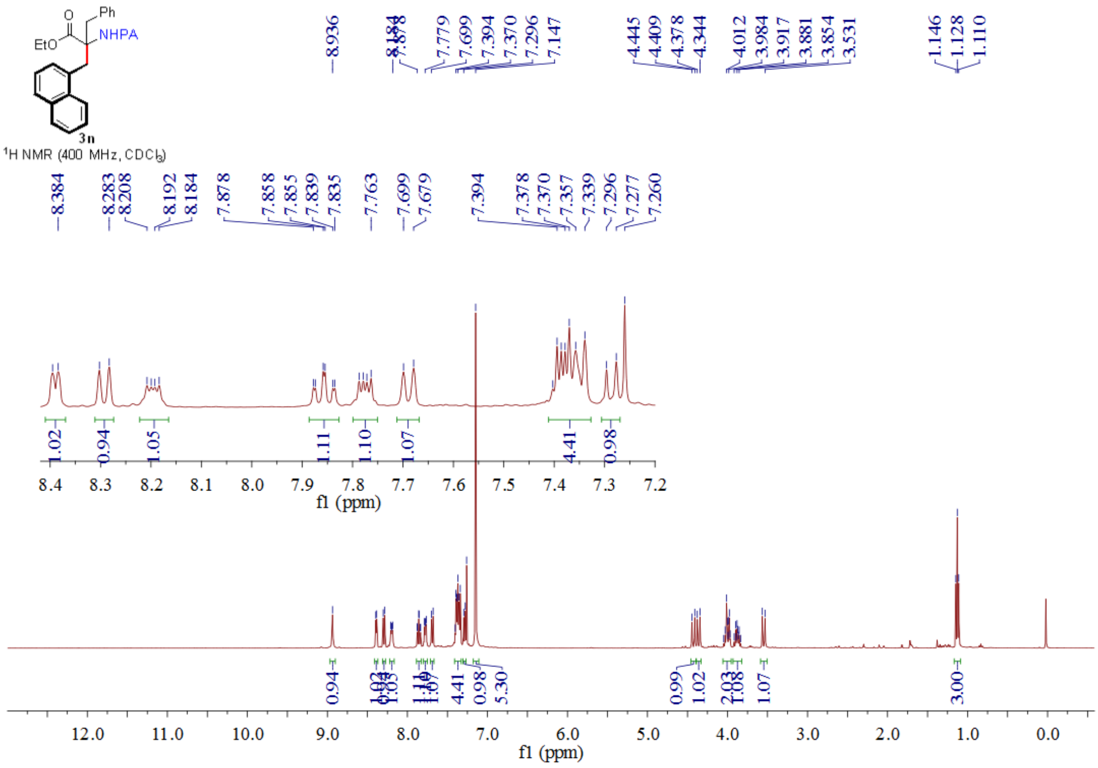

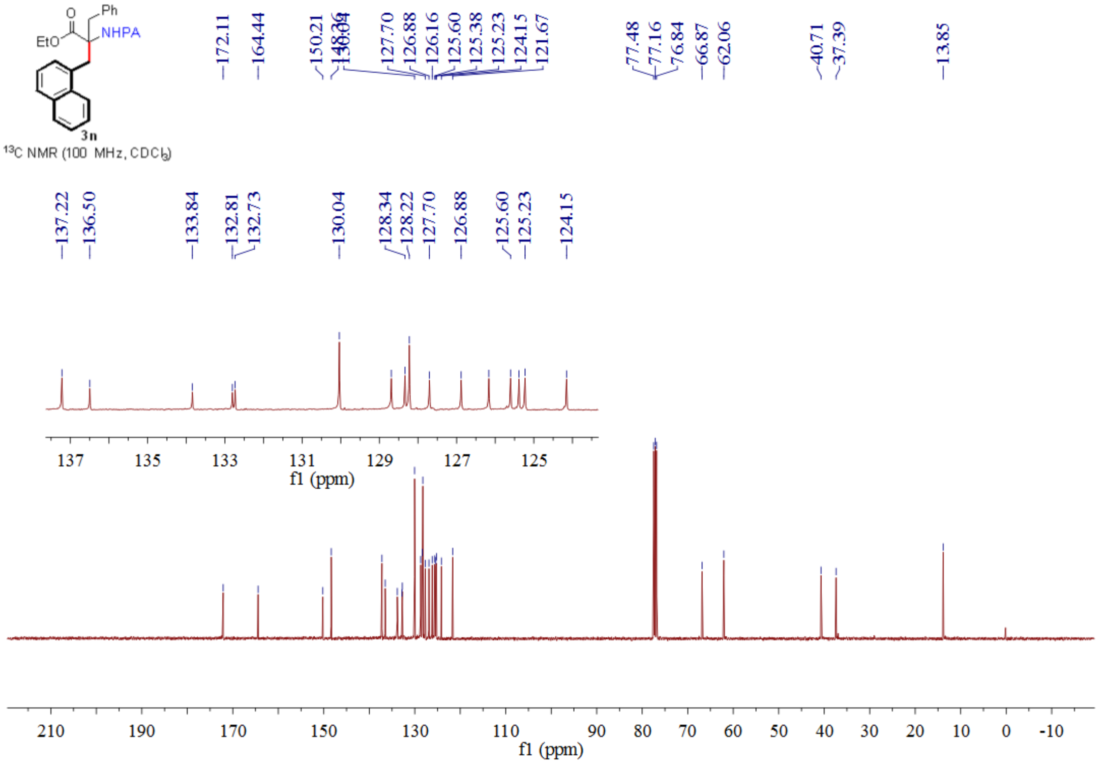

Supplementary Figure 53. ^{1}H NMR and ^{13}C NMR spectra for compound 3n

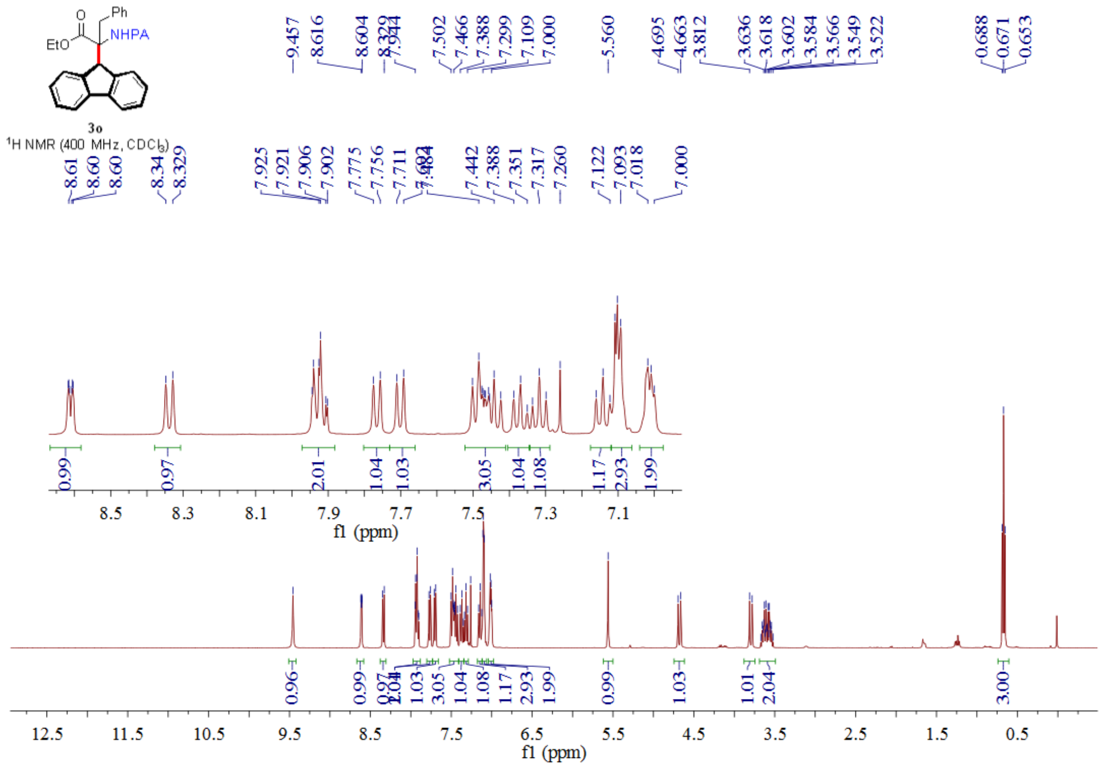

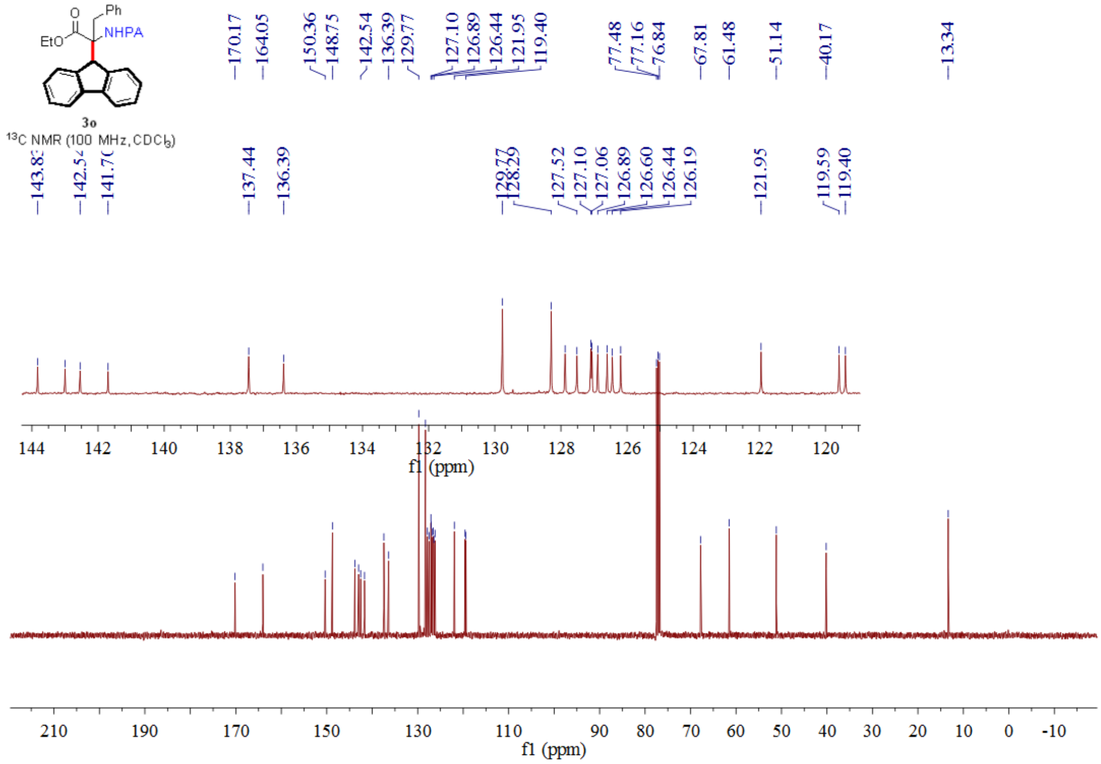

Supplementary Figure 54. ^{1}H NMR and ^{13}C NMR spectra for compound 3o

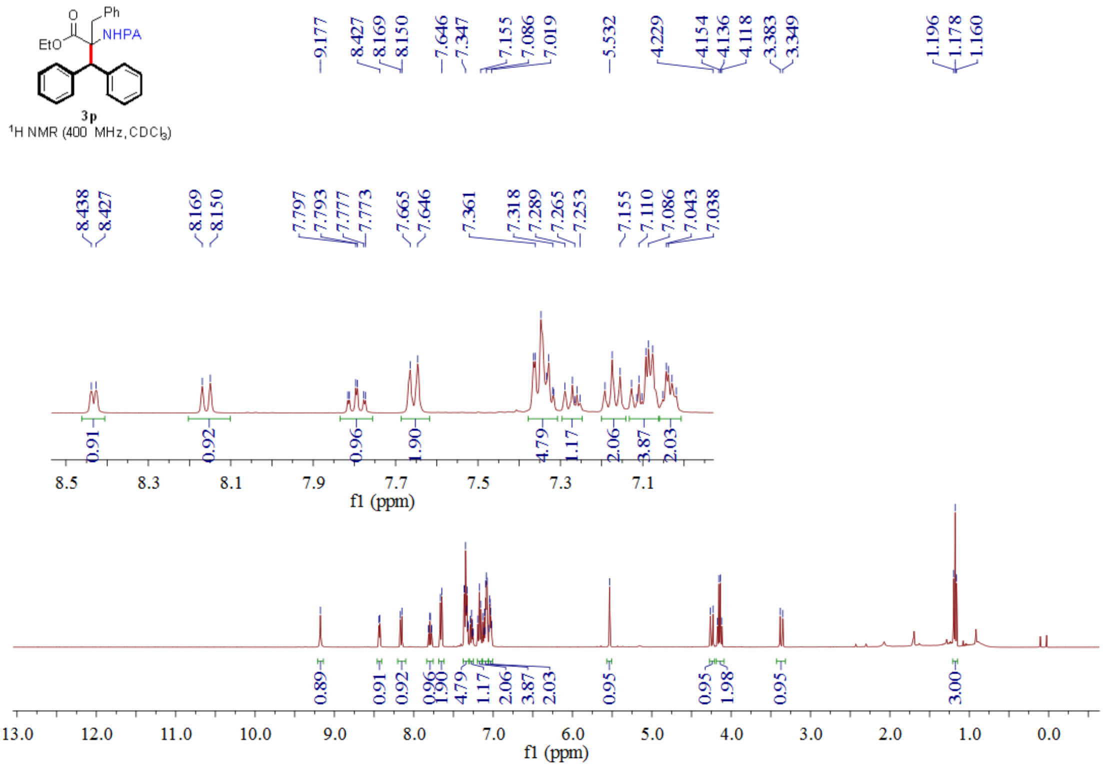

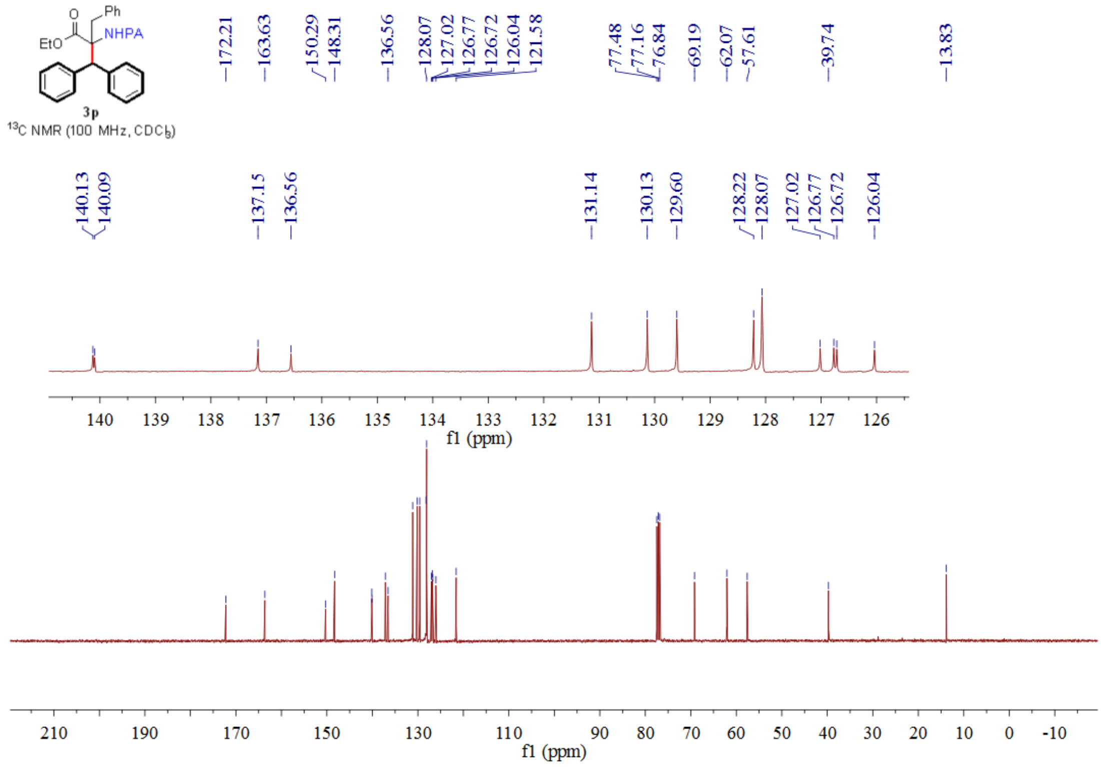

Supplementary Figure 55. ^{1}H NMR and ^{13}C NMR spectra for compound 3p

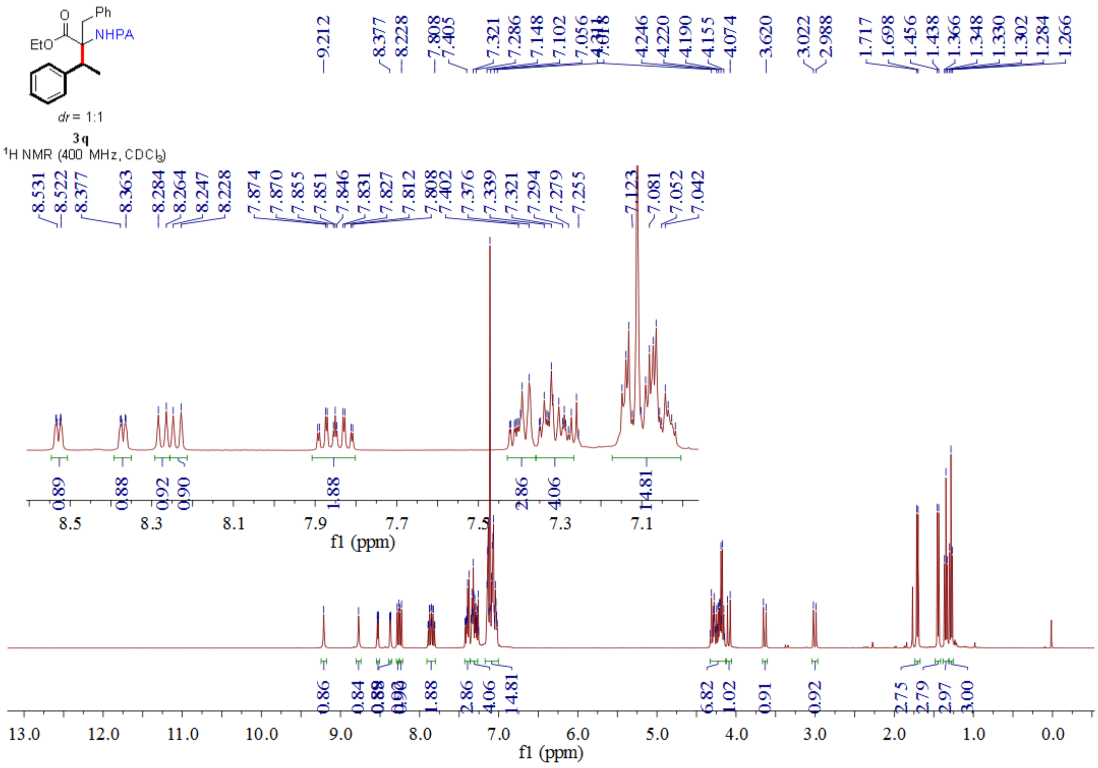

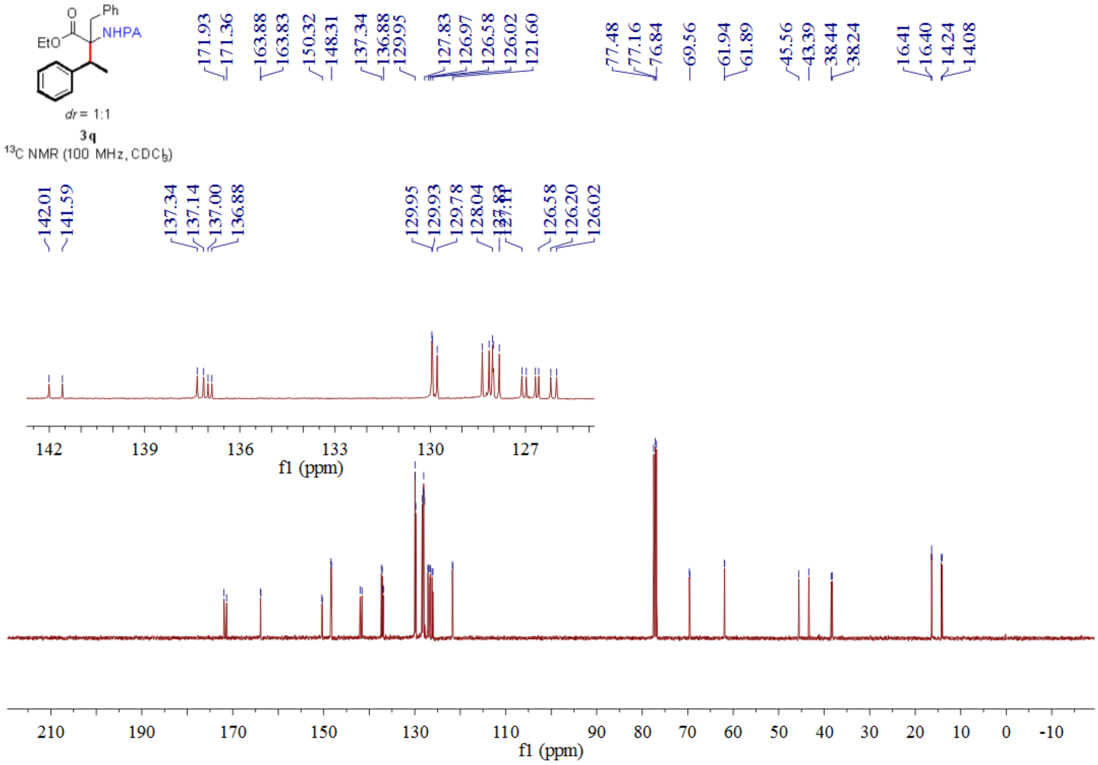

Supplementary Figure 56. ^{1}H NMR and ^{13}C NMR spectra for compound 3q

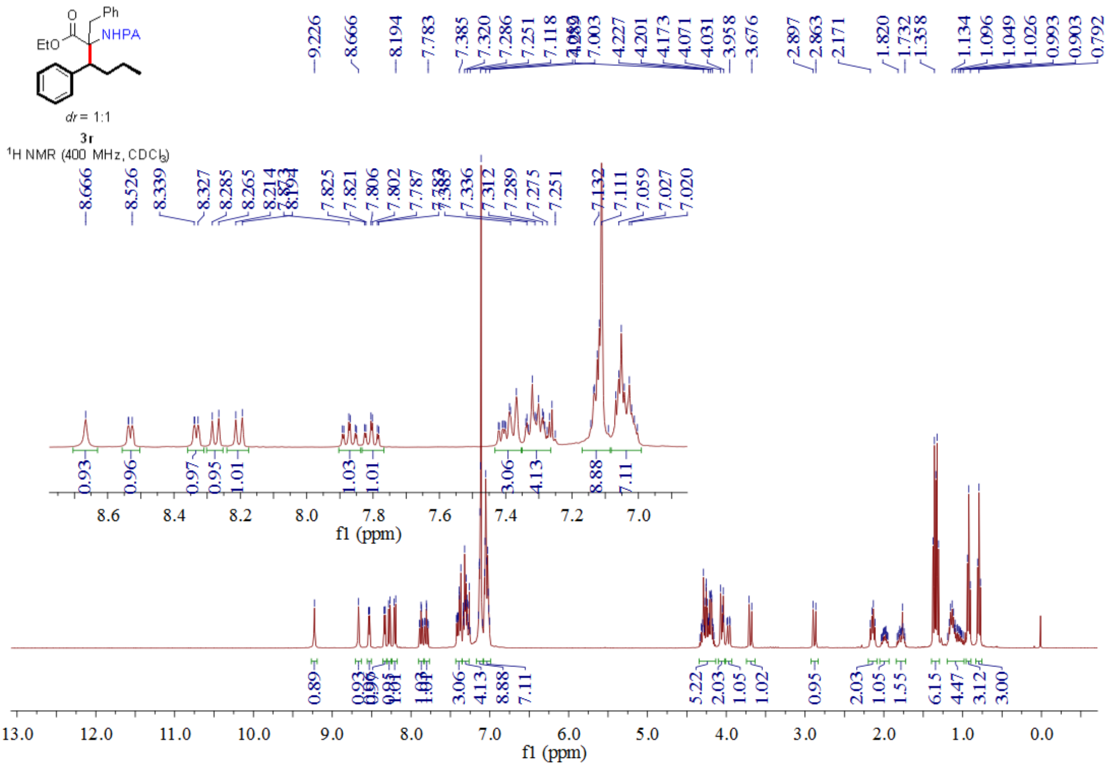

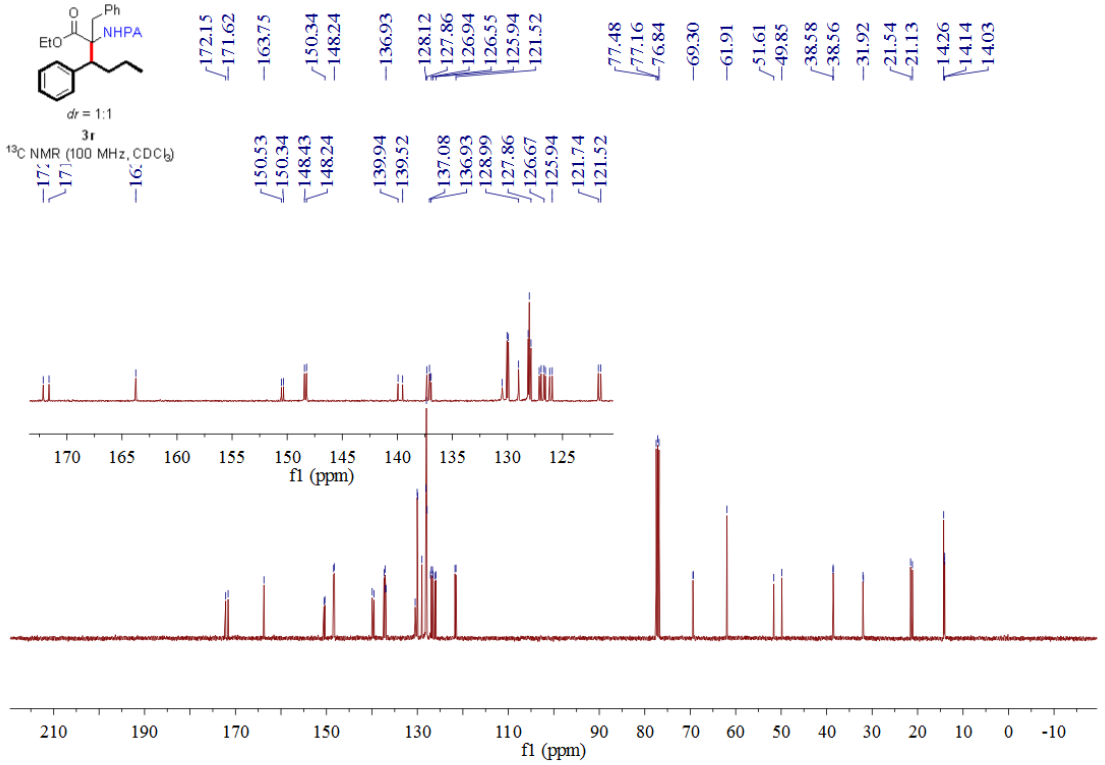

Supplementary Figure 57. ^{1}H NMR and ^{13}C NMR spectra for compound 3r

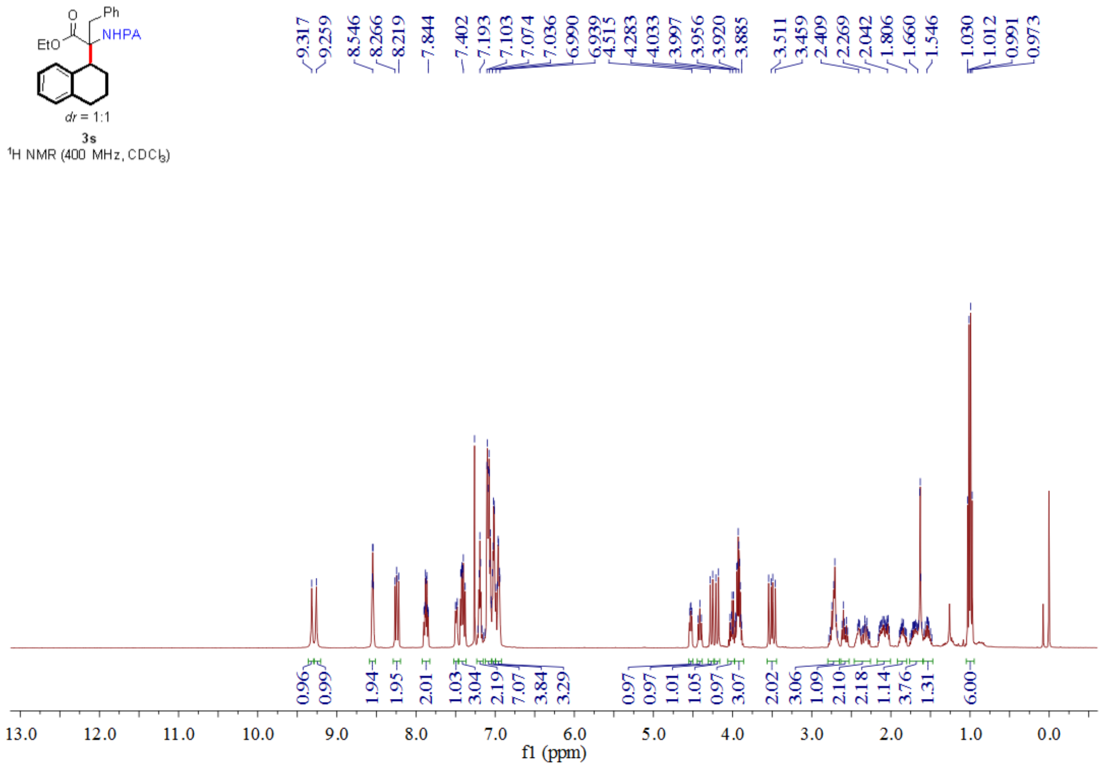

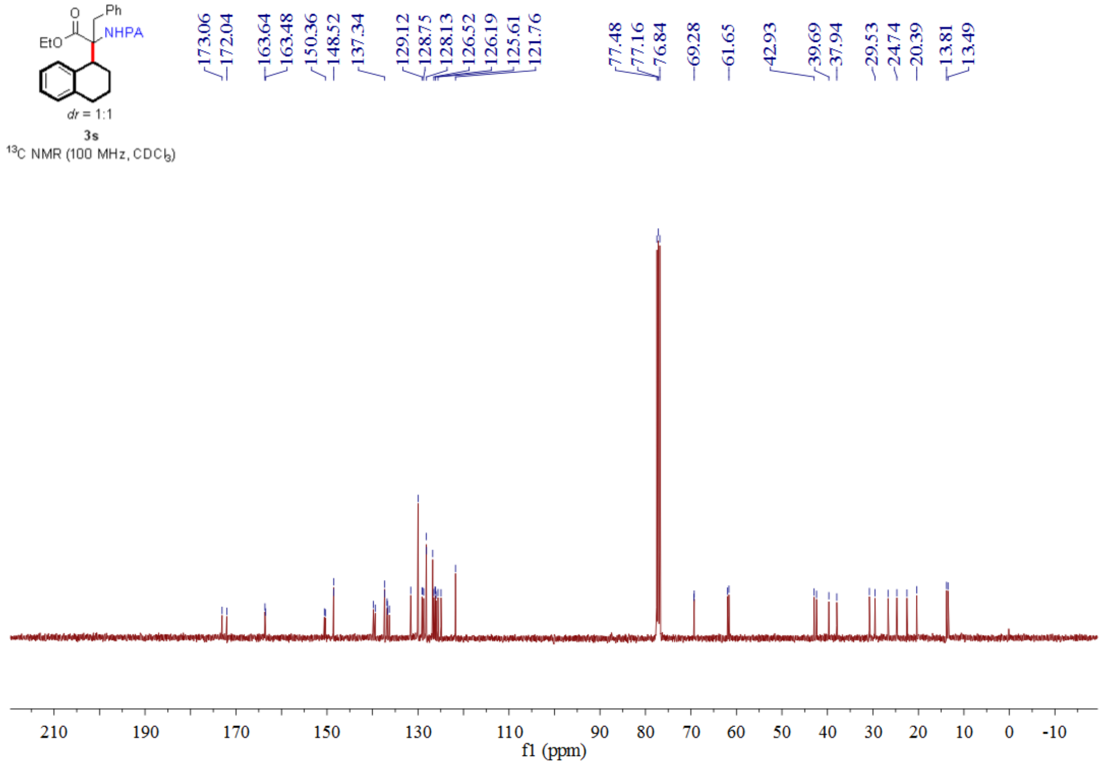

Supplementary Figure 58. ^{1}H NMR and ^{13}C NMR spectra for compound 3s

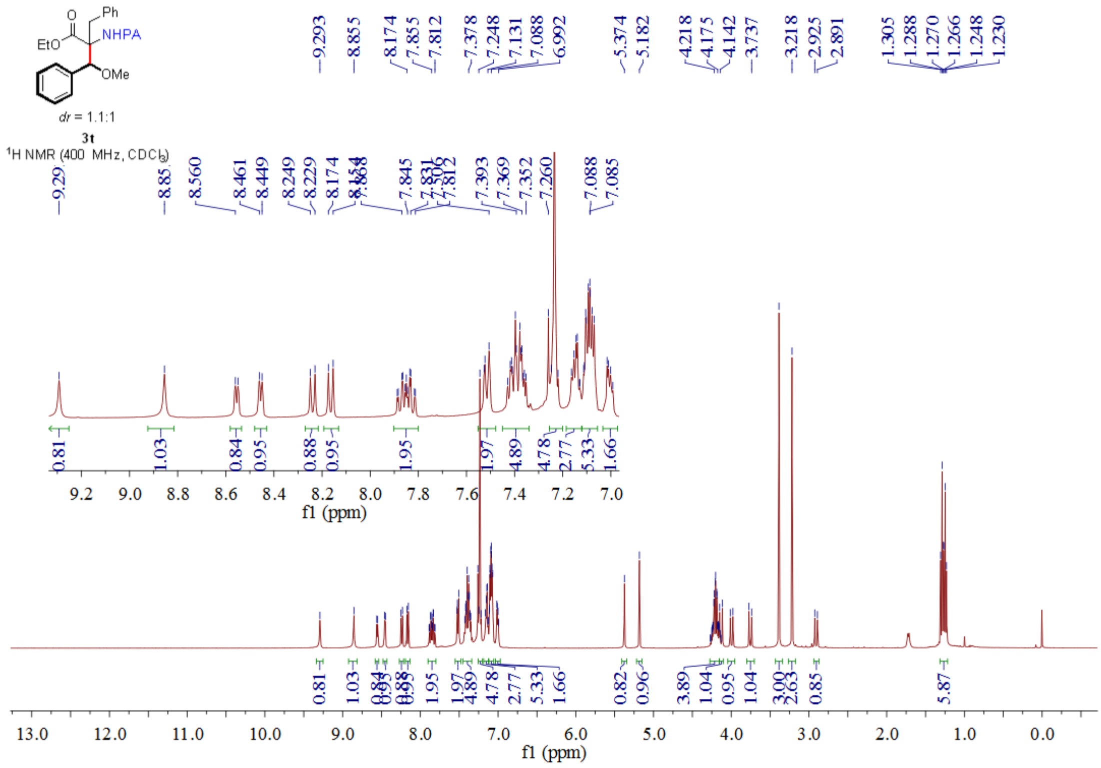

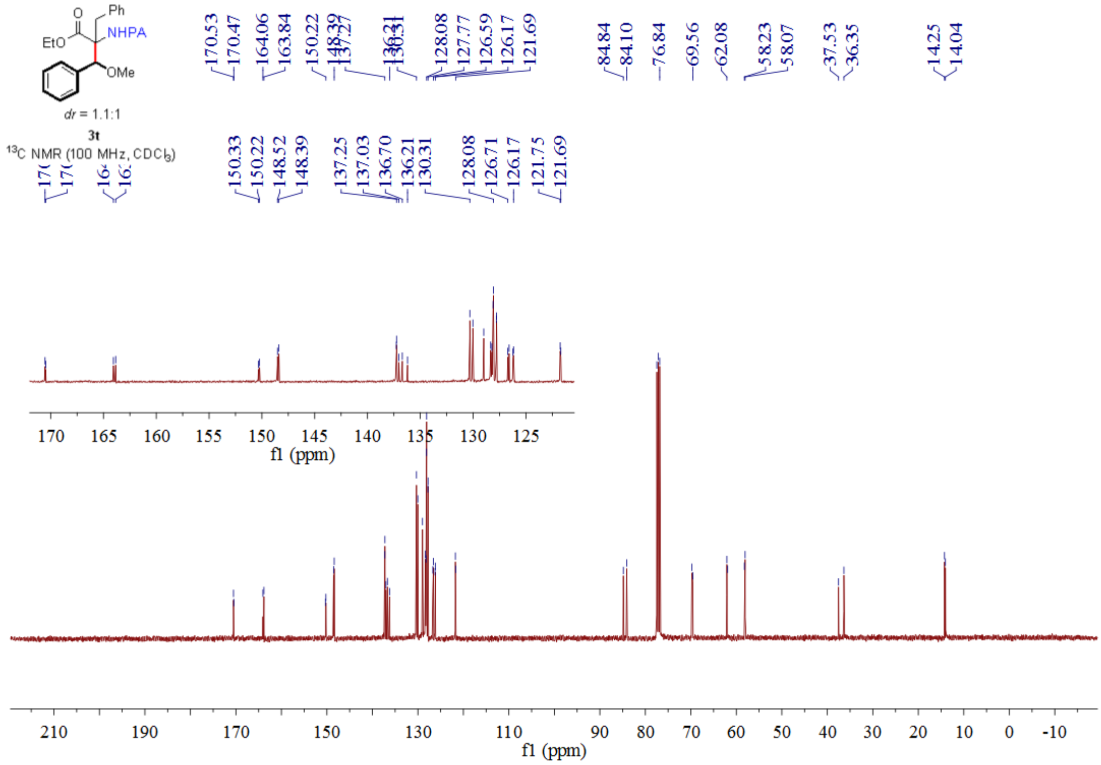

Supplementary Figure 59. ^{1}H NMR and ^{13}C NMR spectra for compound 3t

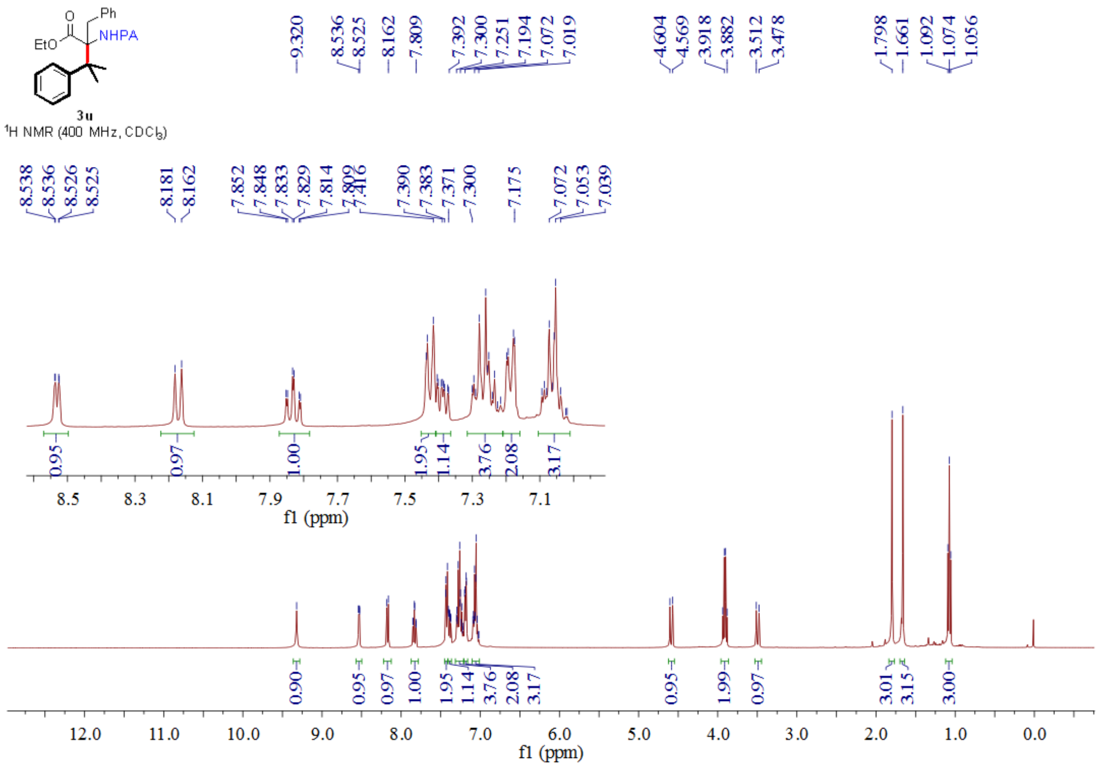

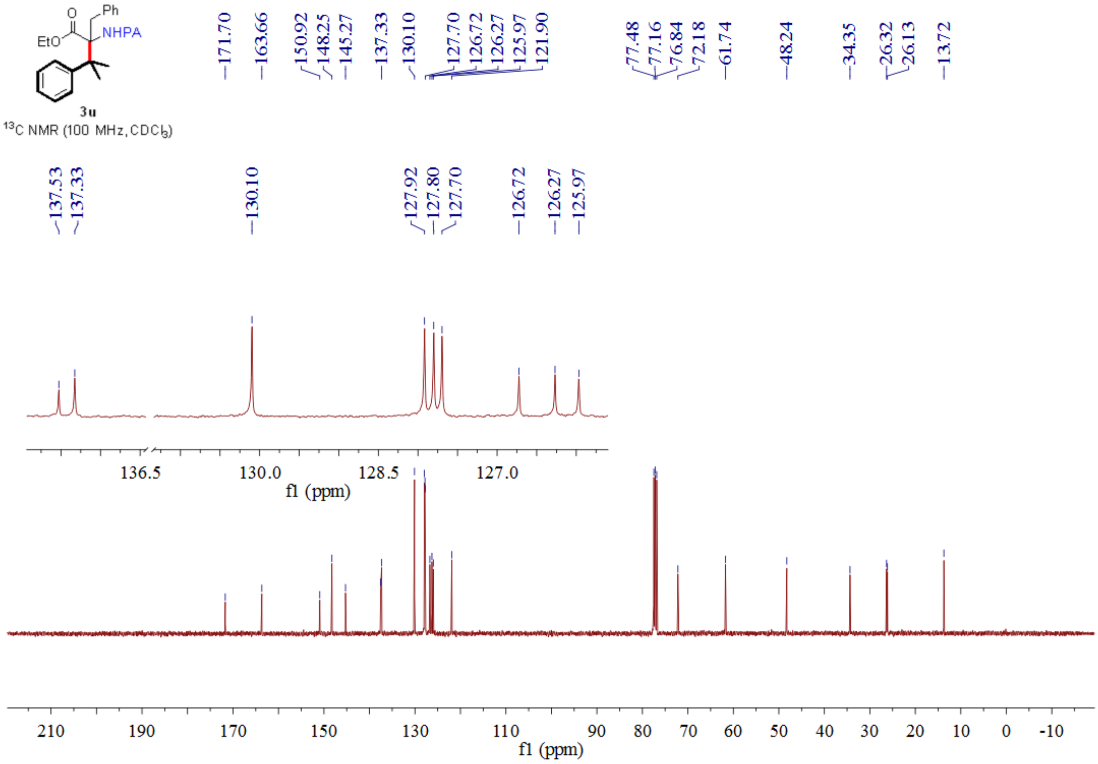

Supplementary Figure 60. ^{1}H NMR and ^{13}C NMR spectra for compound 3u

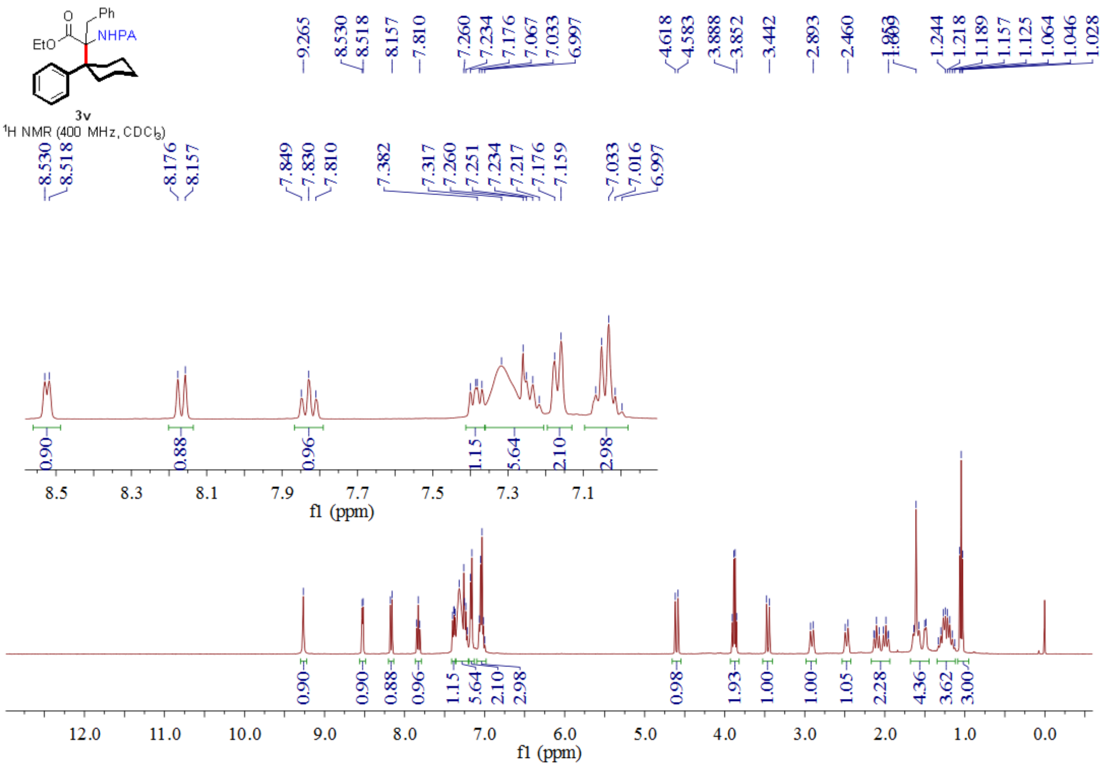

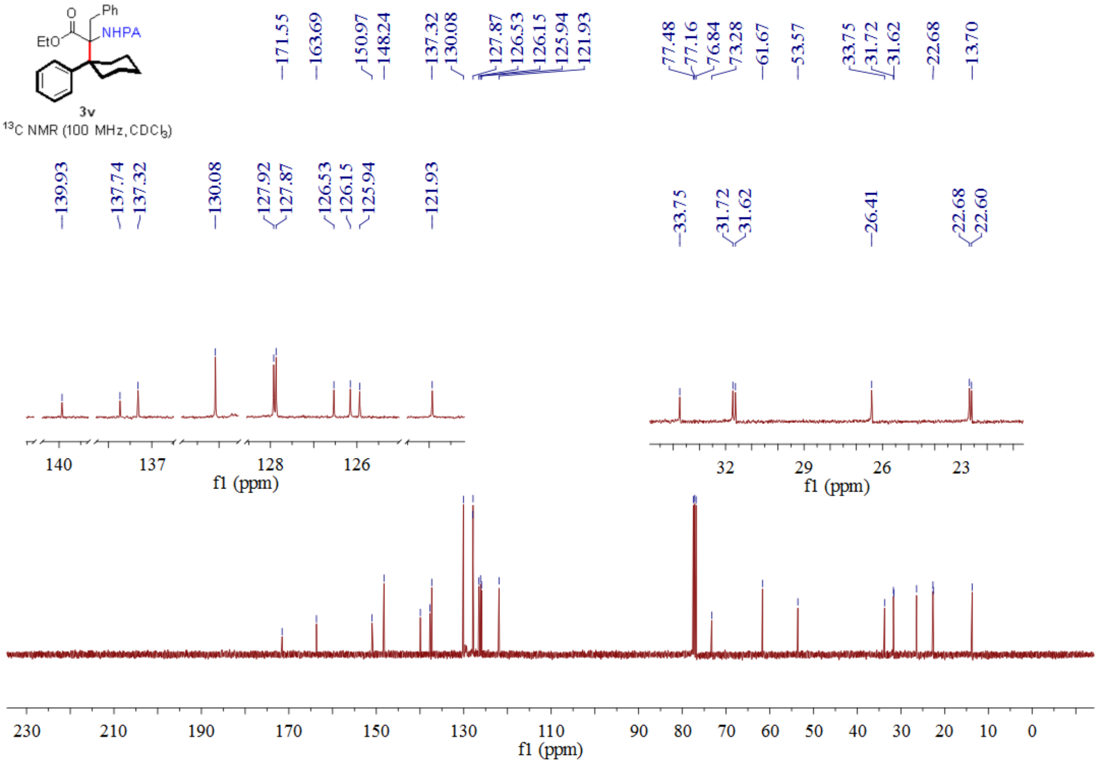

Supplementary Figure 61. ^{1}H NMR and ^{13}C NMR spectra for compound 3v

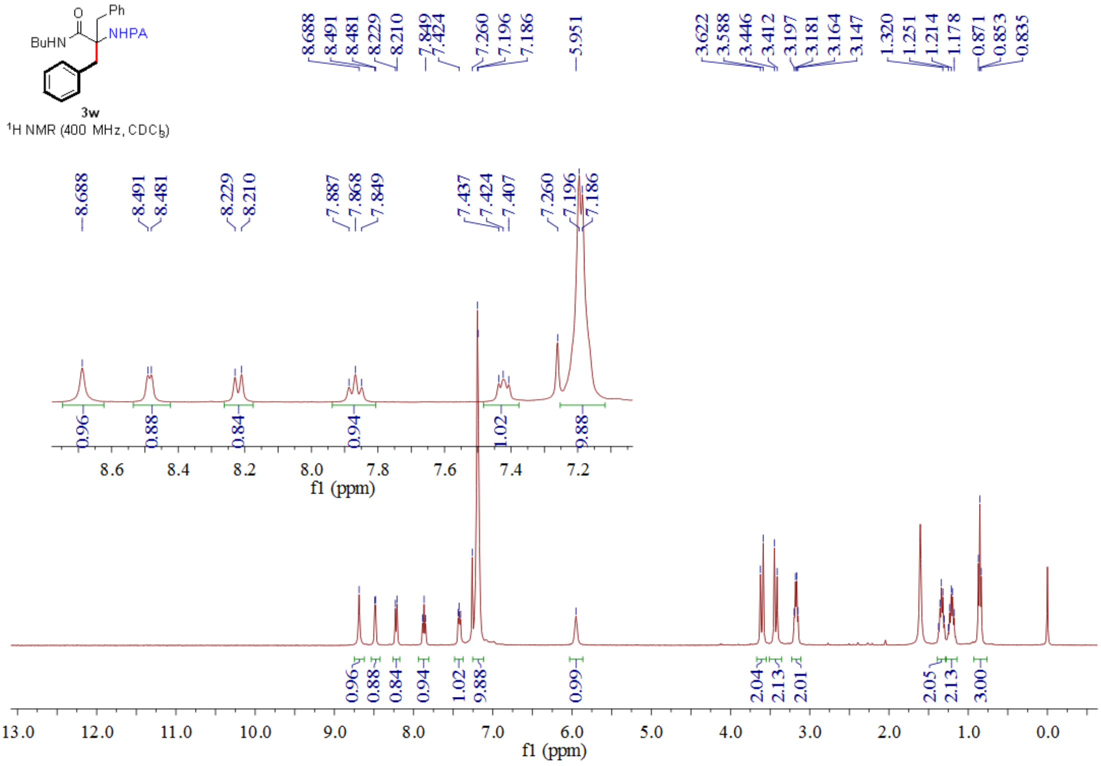

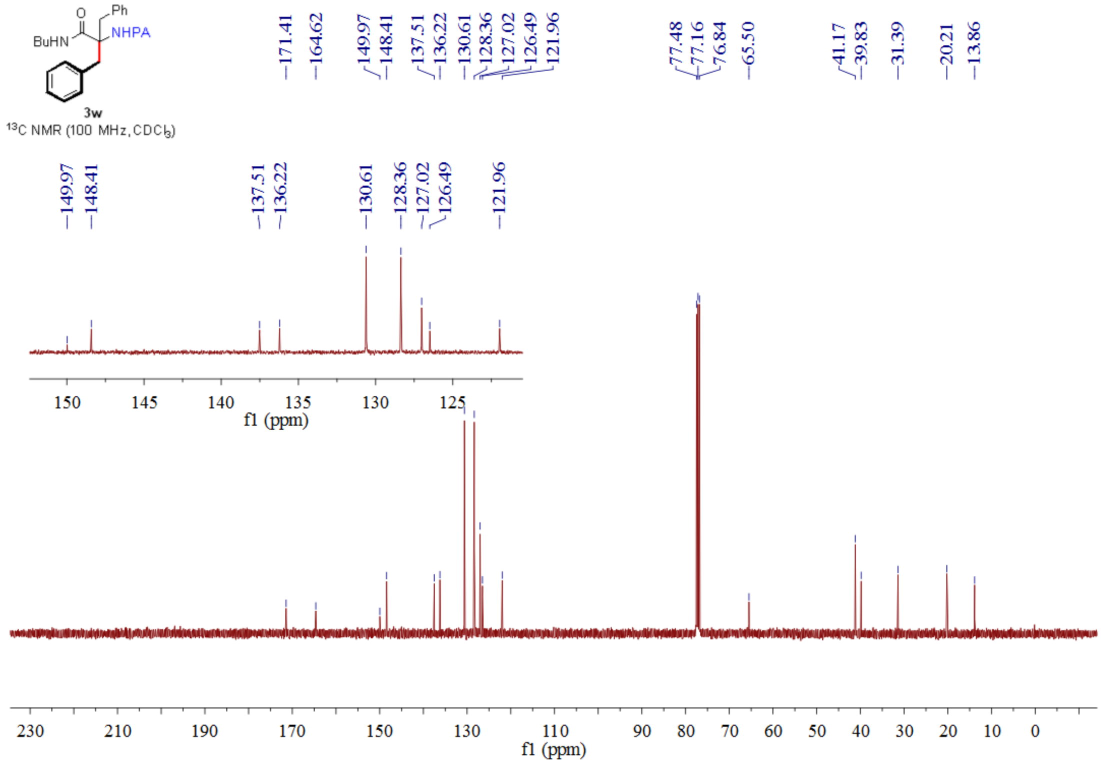

Supplementary Figure 62. ^{1}H NMR and ^{13}C NMR spectra for compound 3w

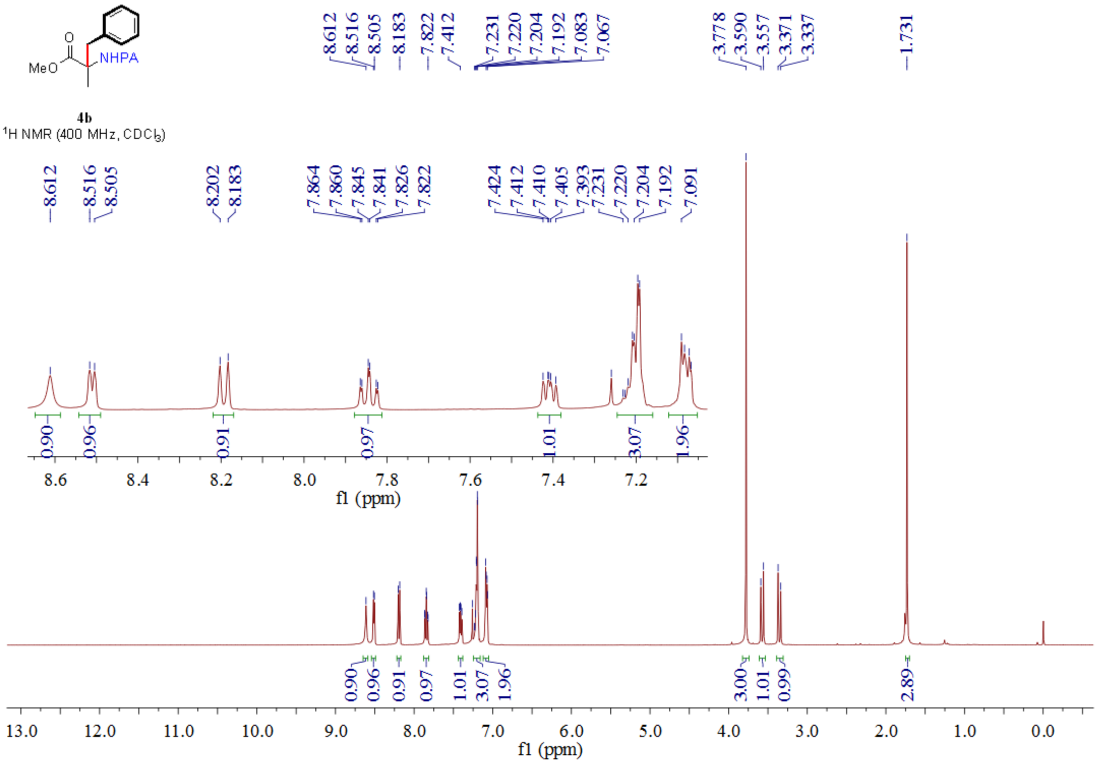

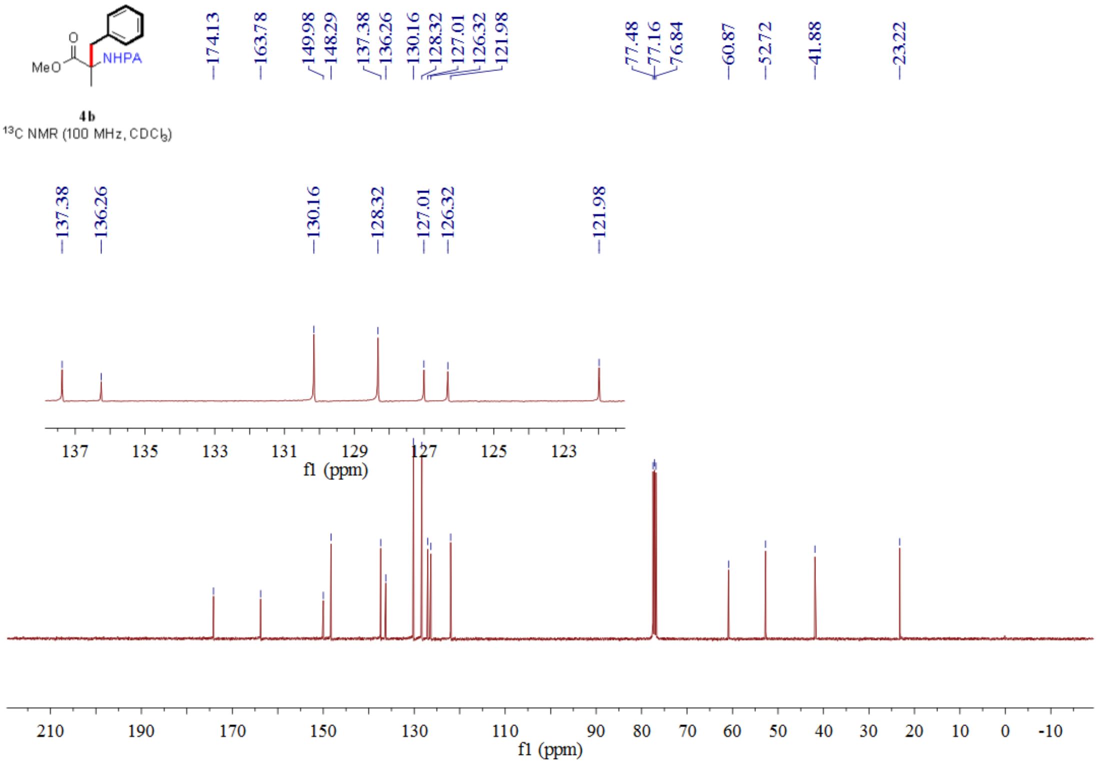

Supplementary Figure 63. ^{1}**H NMR and** ^{13}**C NMR spectra for compound 4b**

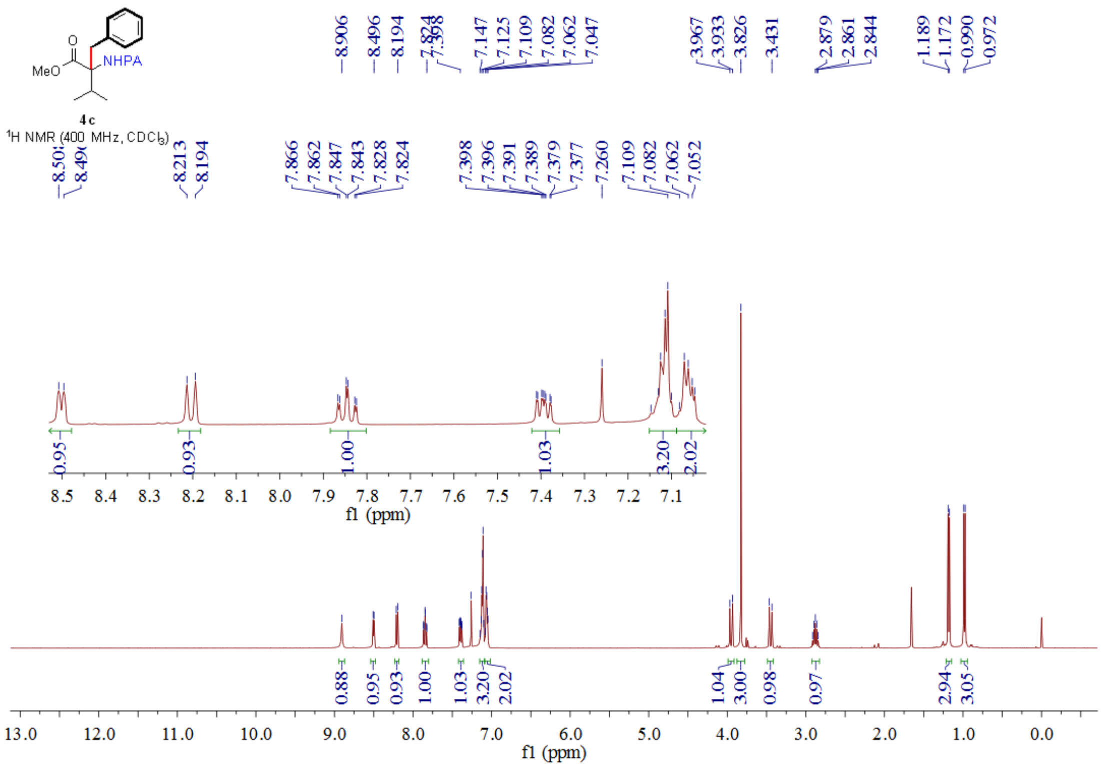

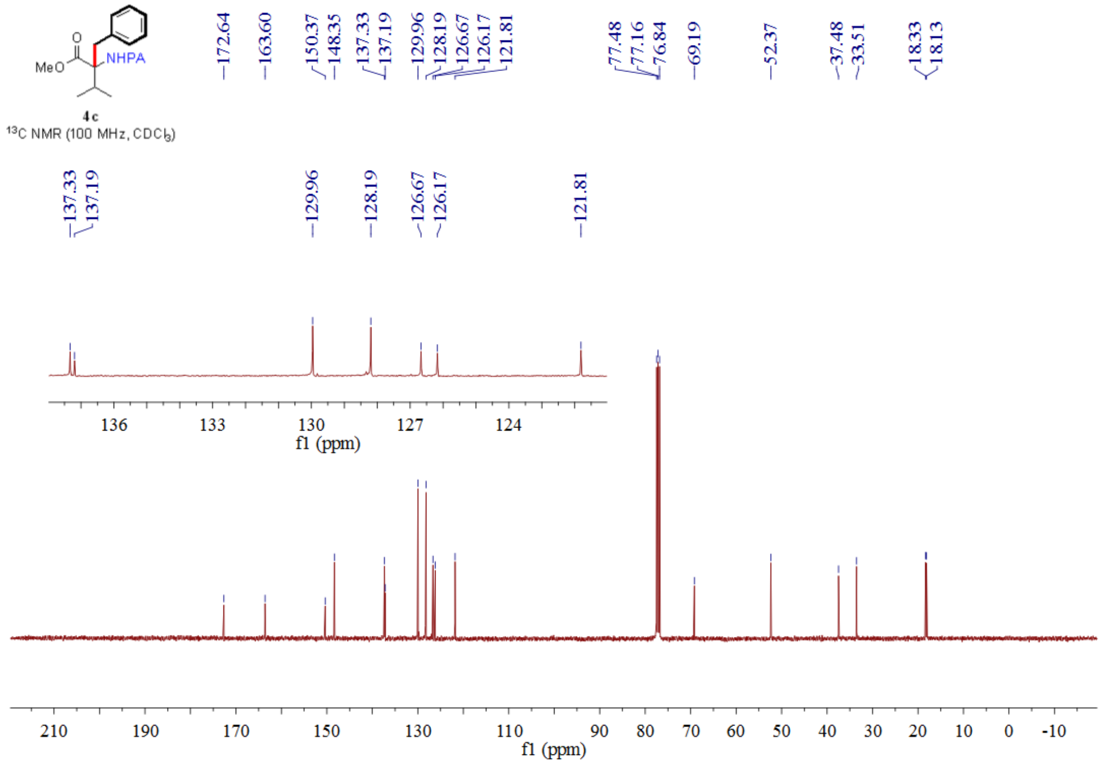

Supplementary Figure 64. ^{1}H NMR and ^{13}C NMR spectra for compound 4c

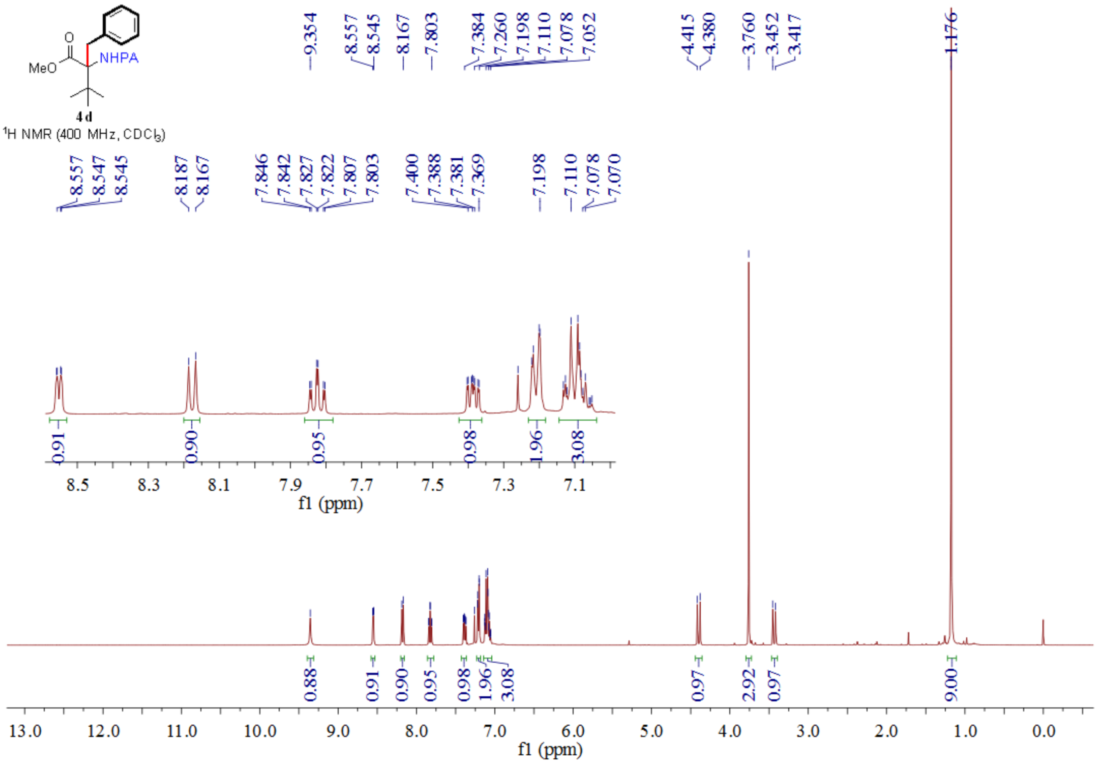

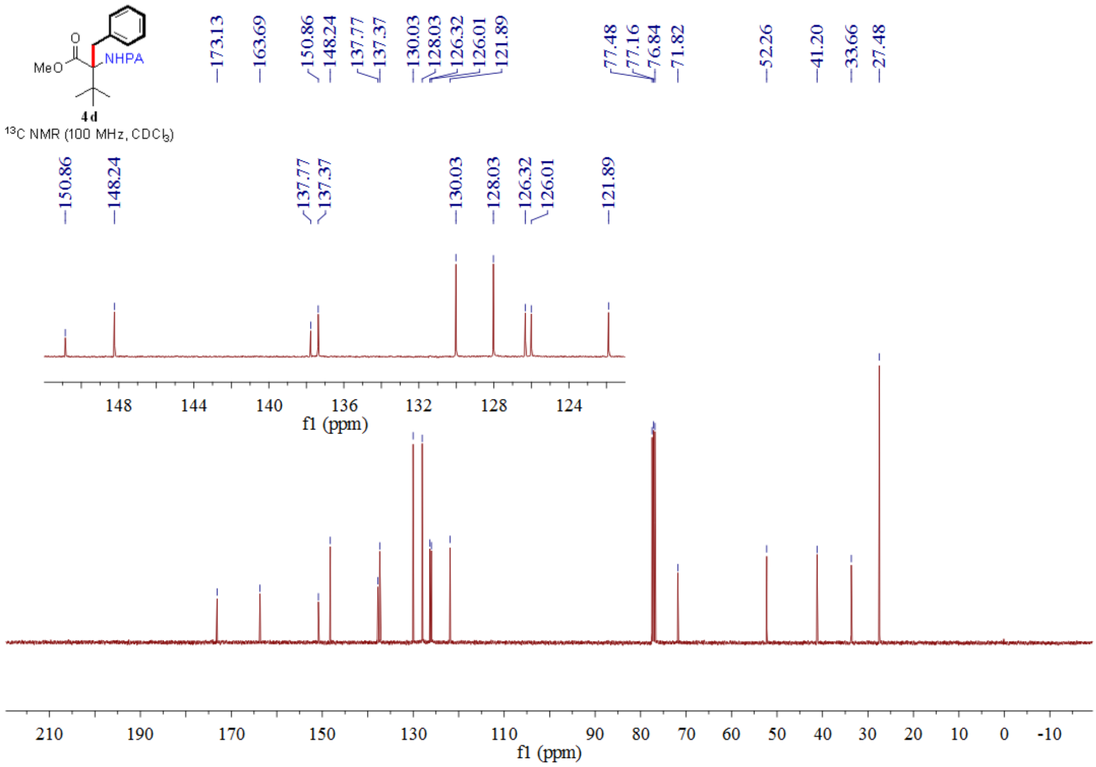

Supplementary Figure 65. ^{1}H NMR and ^{13}C NMR spectra for compound 4d

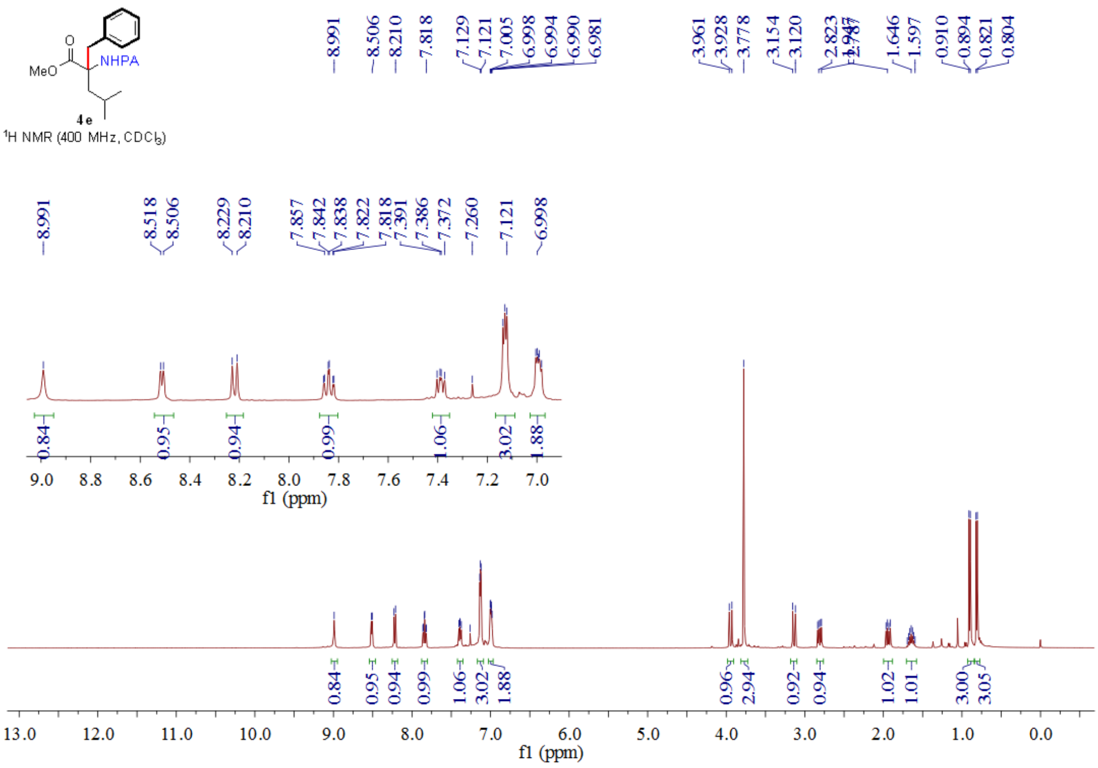

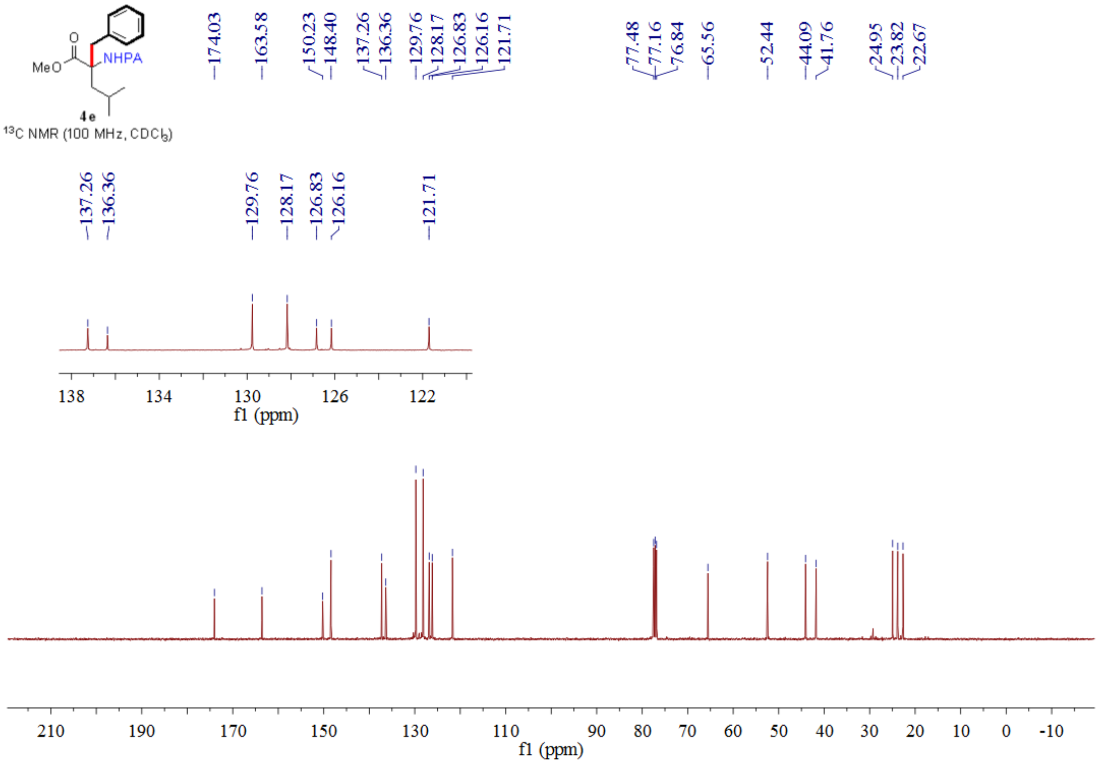

Supplementary Figure 66. ^{1}H NMR and ^{13}C NMR spectra for compound 4e

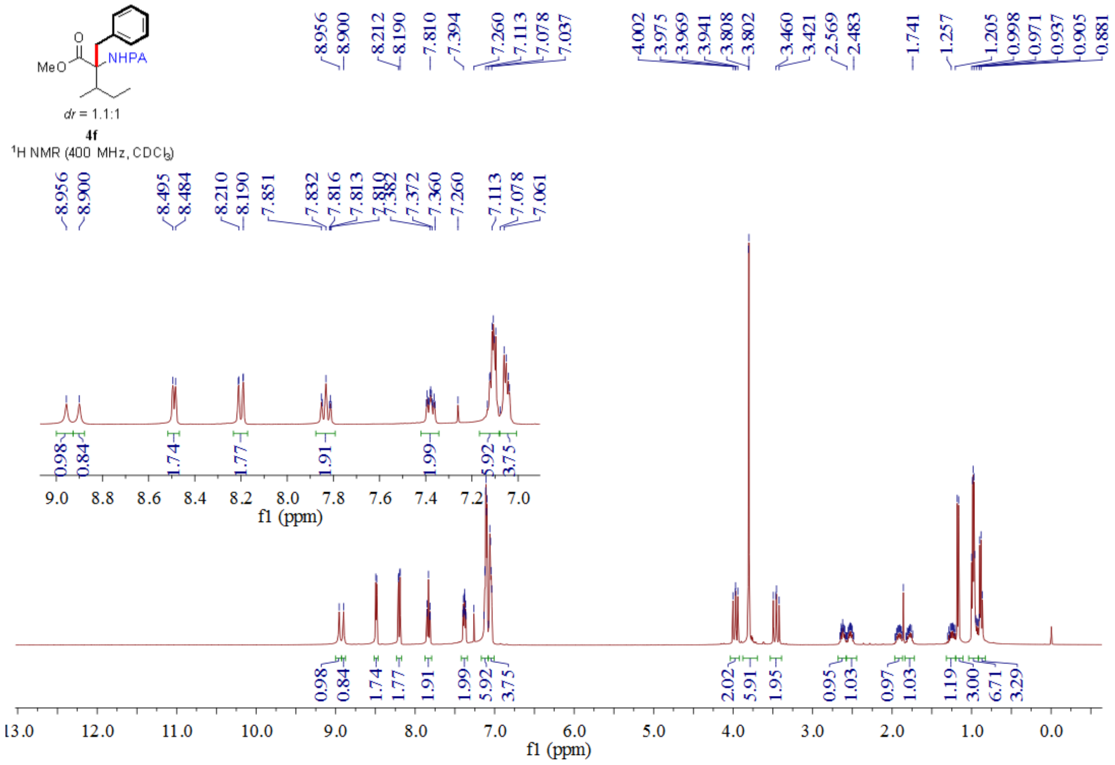

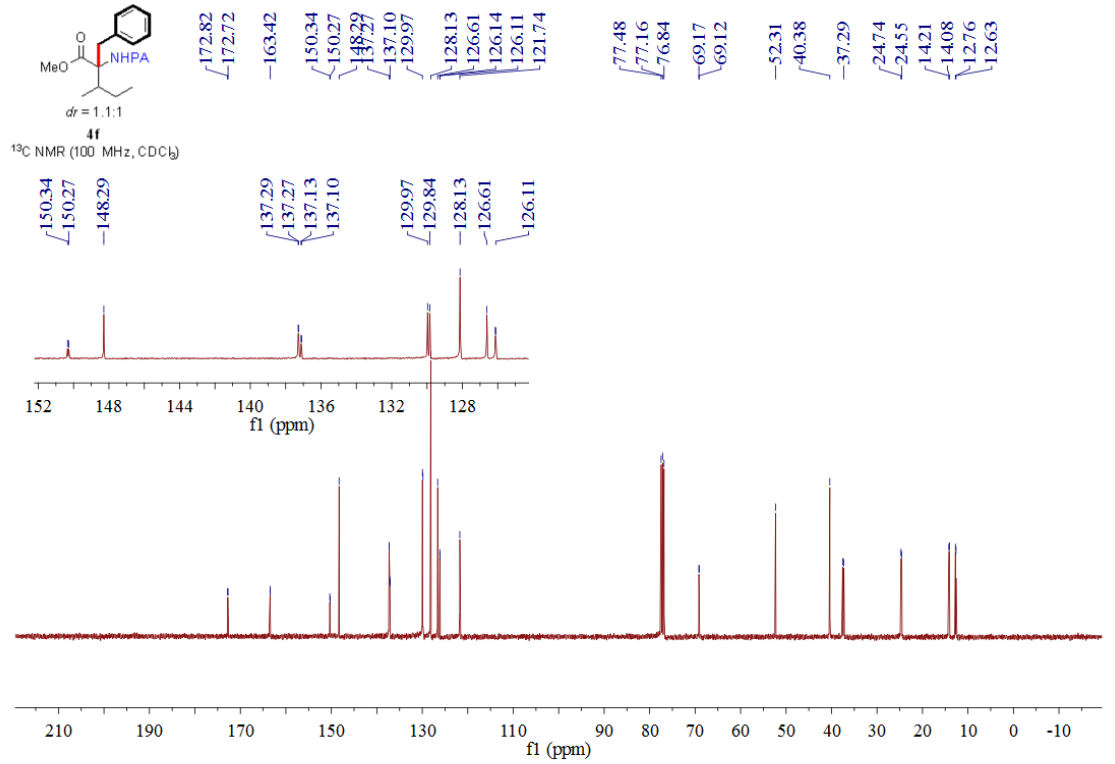

Supplementary Figure 67. ^{1}**H NMR and** ^{13}**C NMR spectra for compound 4f**

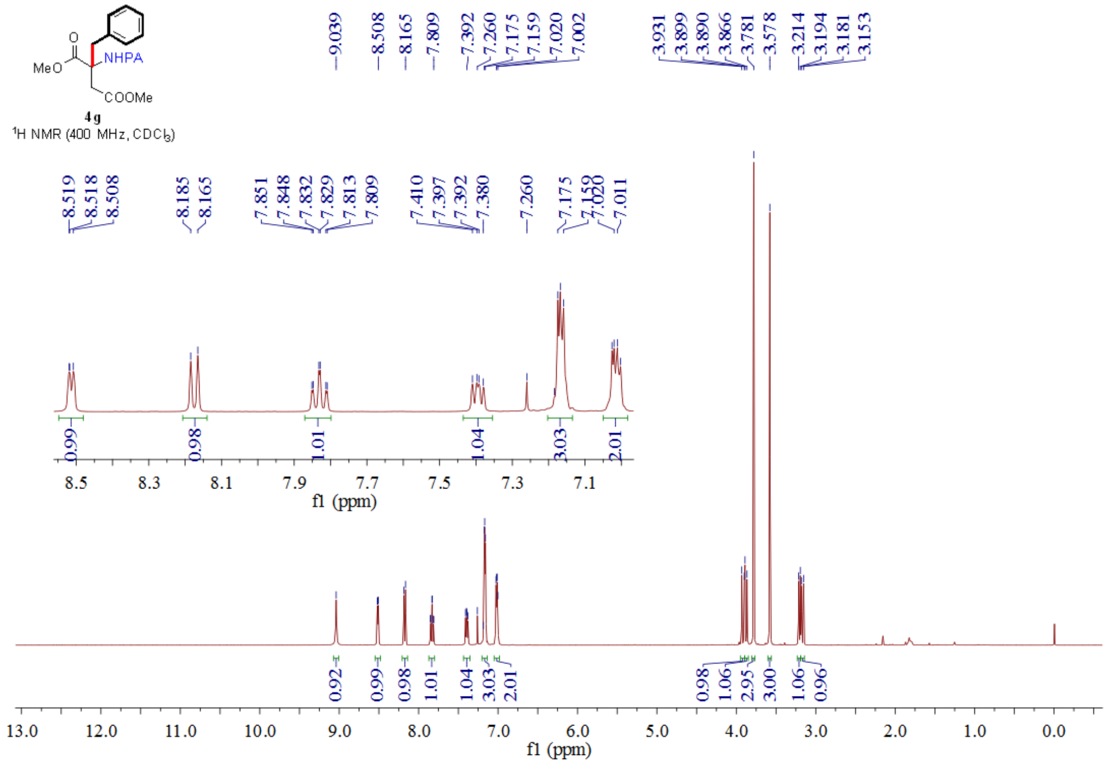

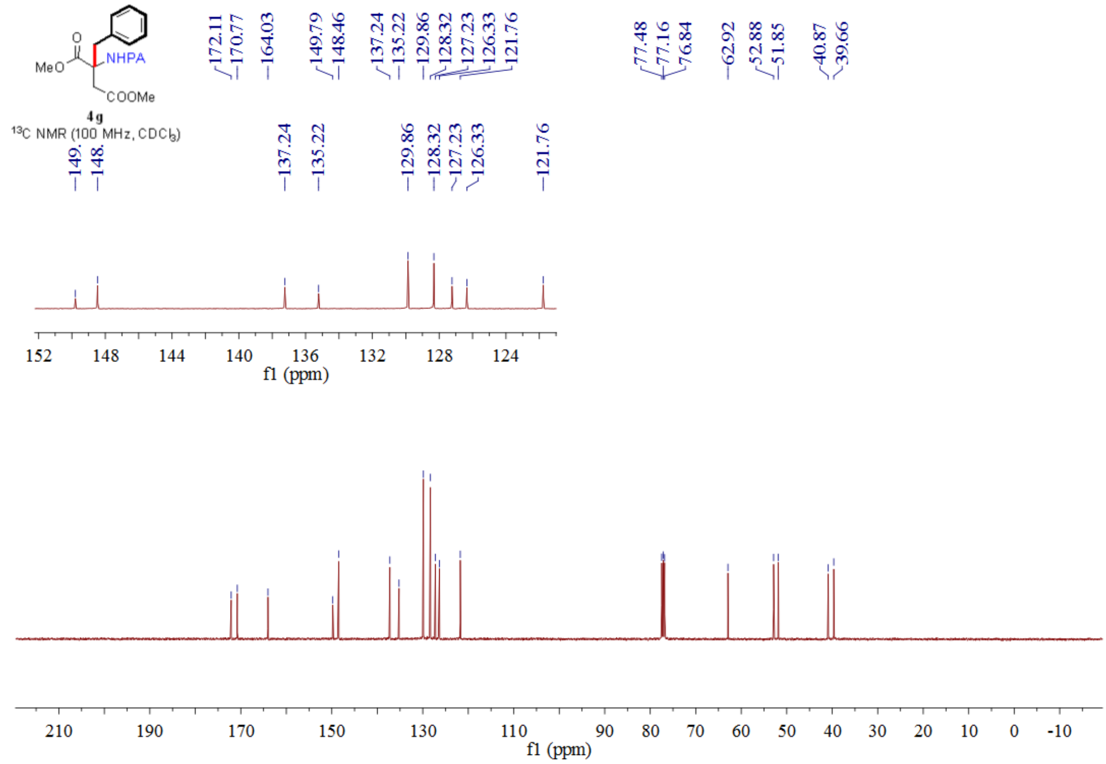

Supplementary Figure 68. ^{1}H NMR and ^{13}C NMR spectra for compound 4g

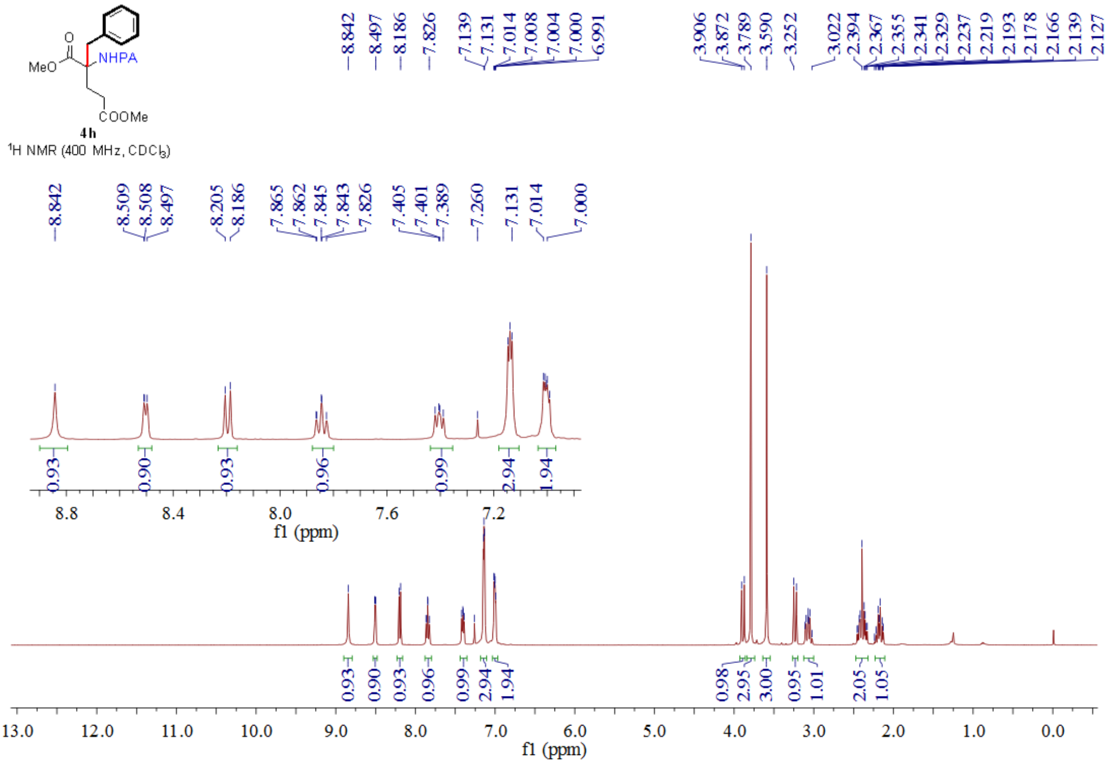

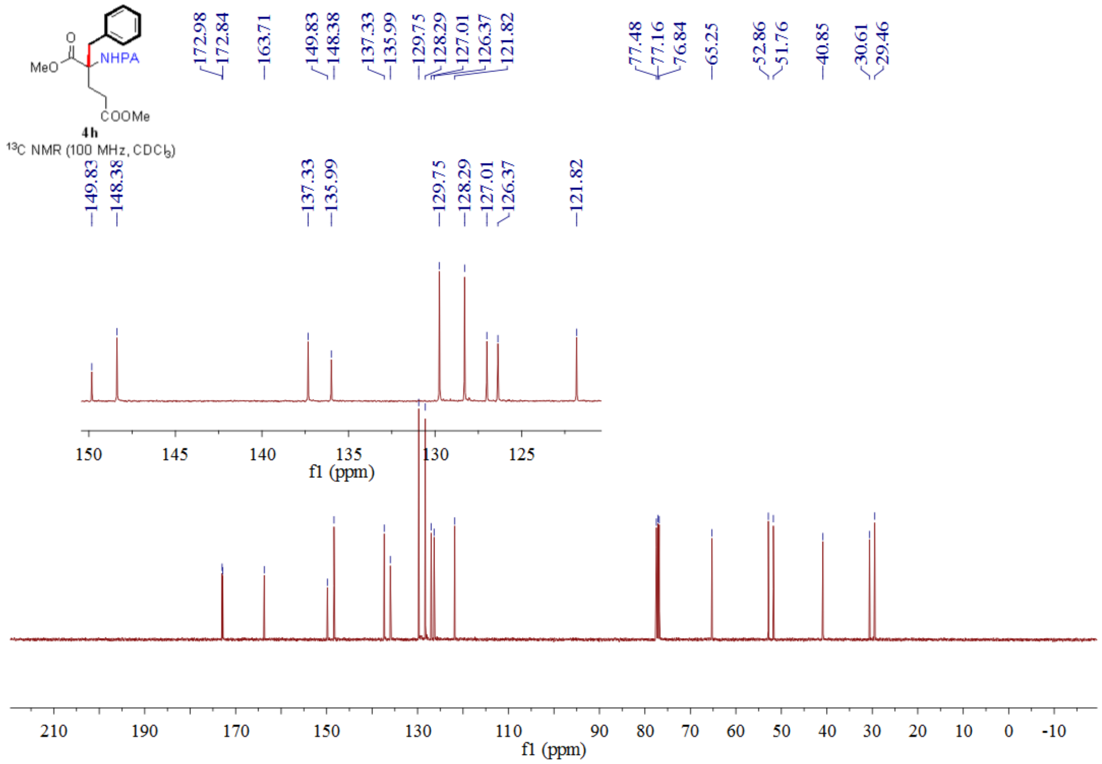

Supplementary Figure 69. ^{1}H NMR and ^{13}C NMR spectra for compound 4h

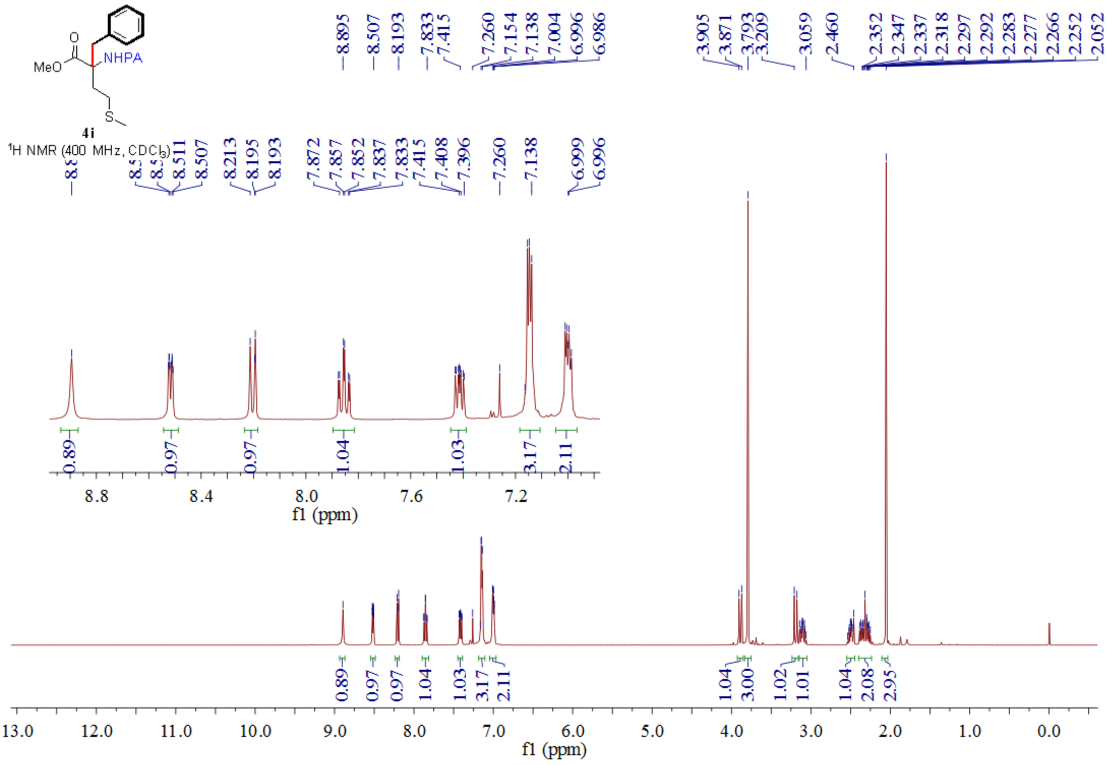

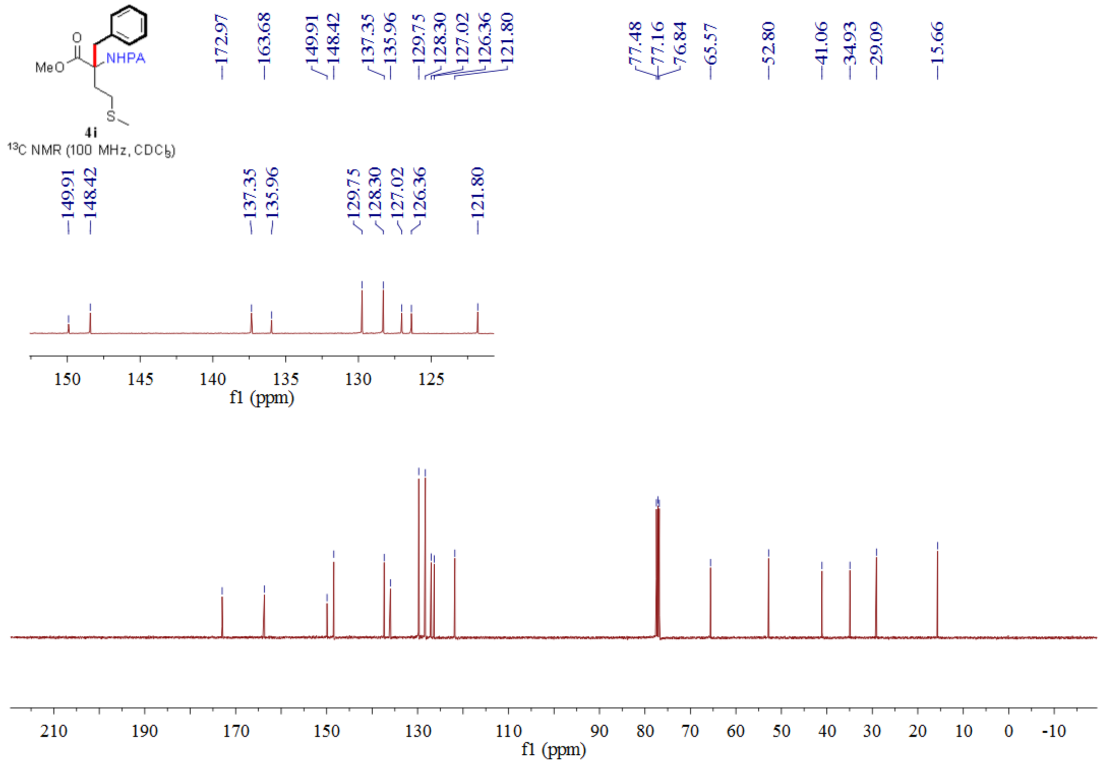

Supplementary Figure 70. ^{1}H NMR and ^{13}C NMR spectra for compound 4i

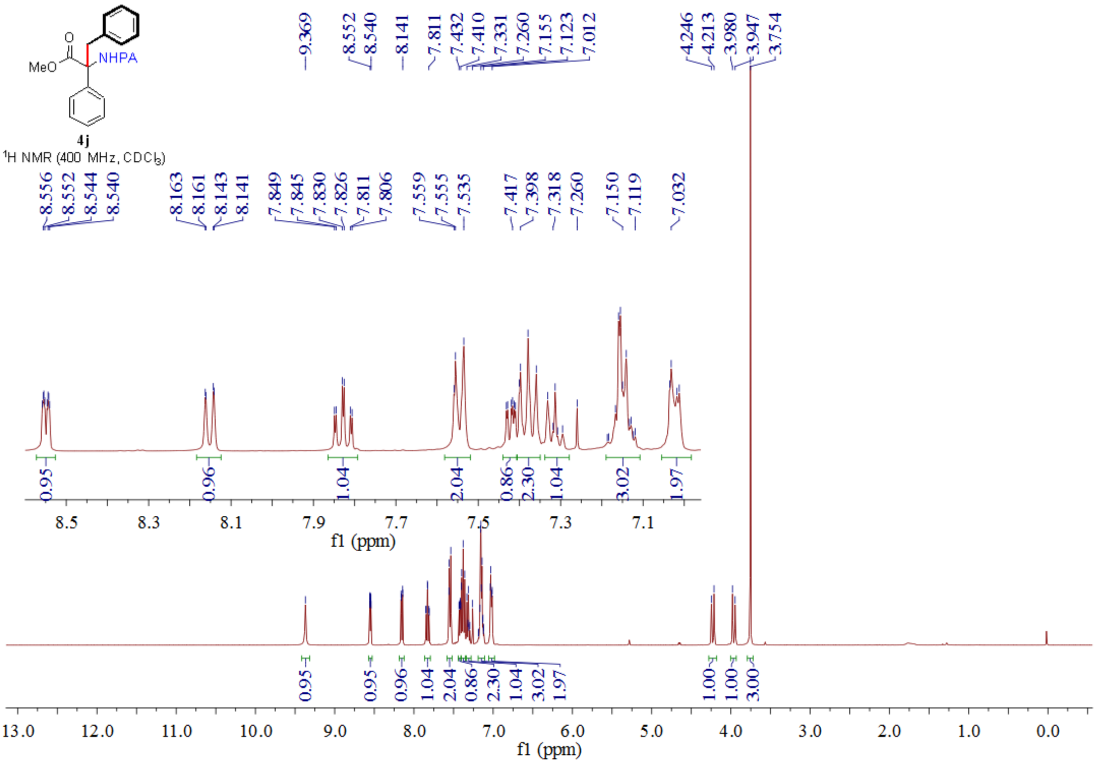

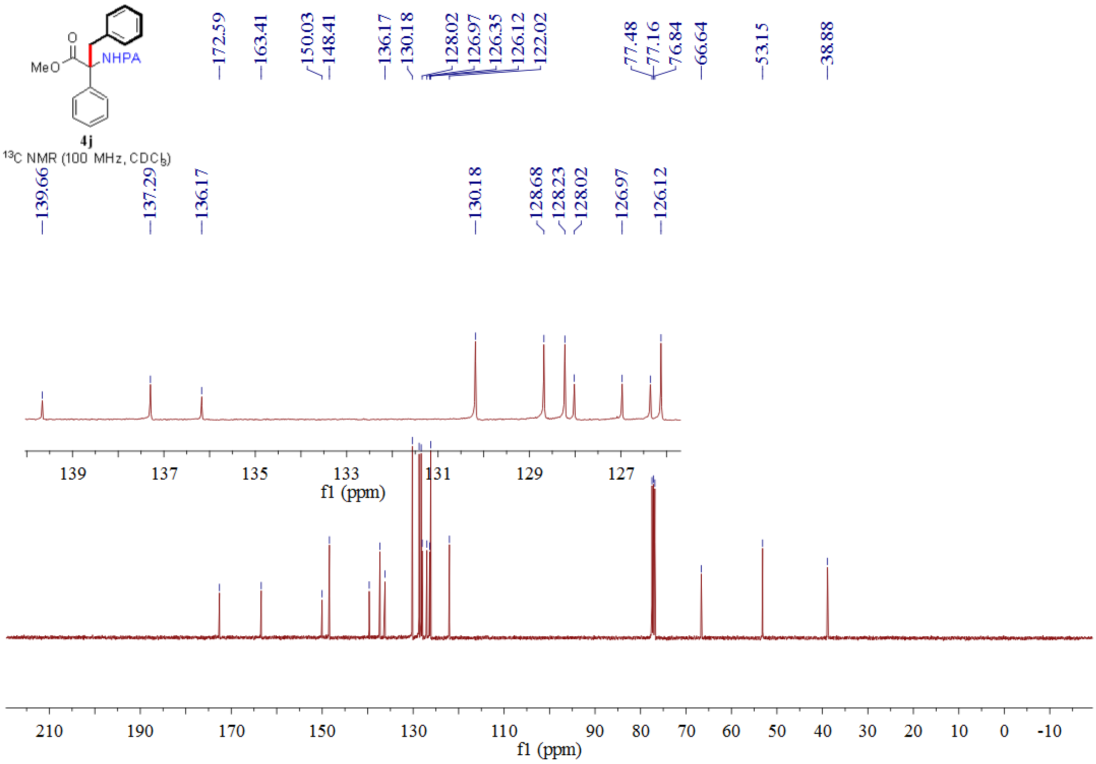

Supplementary Figure 71. ^{1}**H NMR and** ^{13}**C NMR spectra for compound 4j**

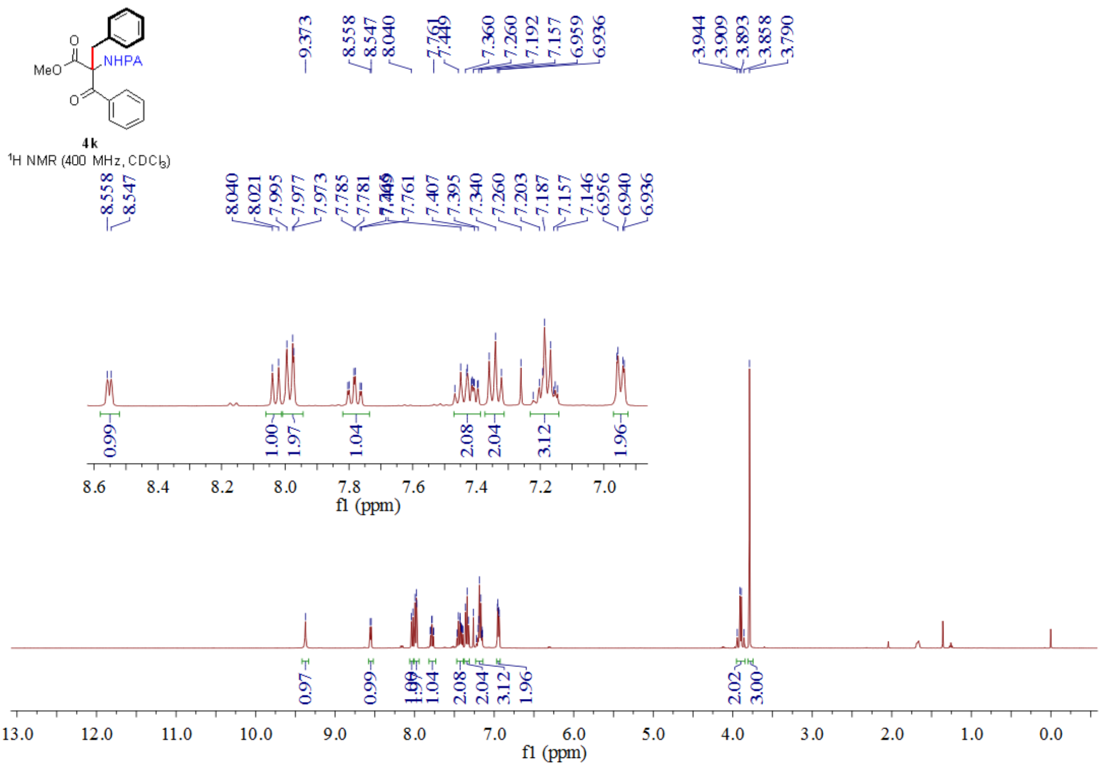

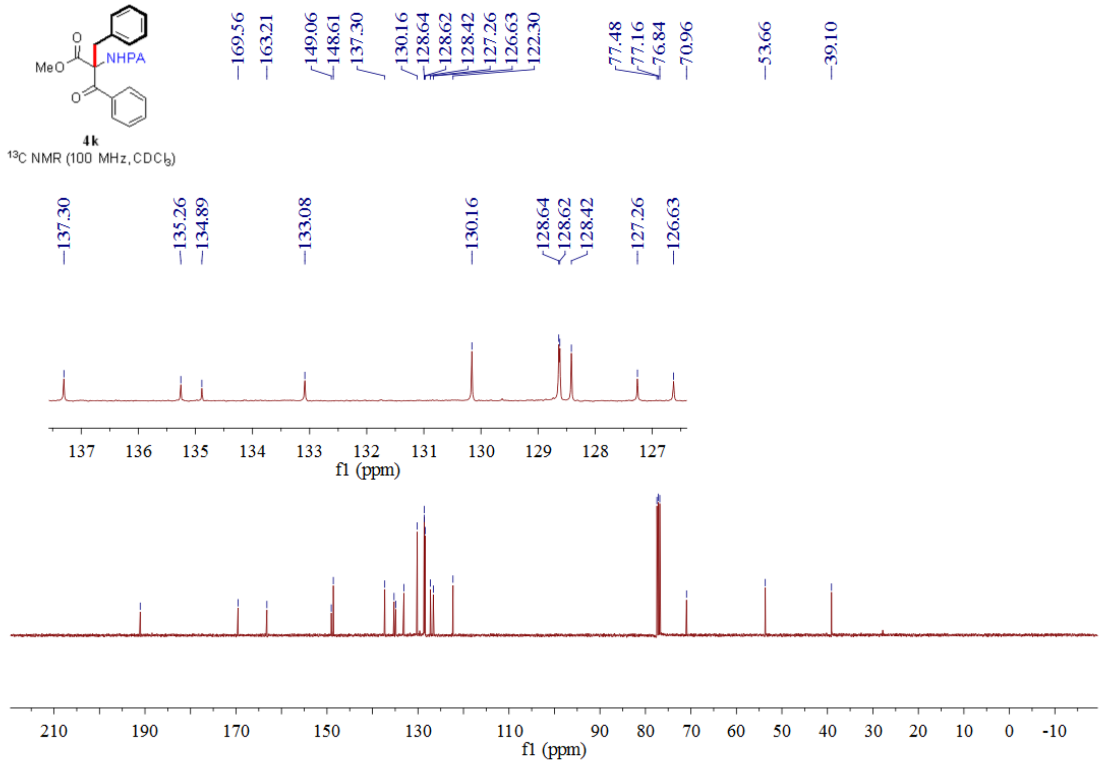

Supplementary Figure 72. ^{1}H NMR and ^{13}C NMR spectra for compound 4k

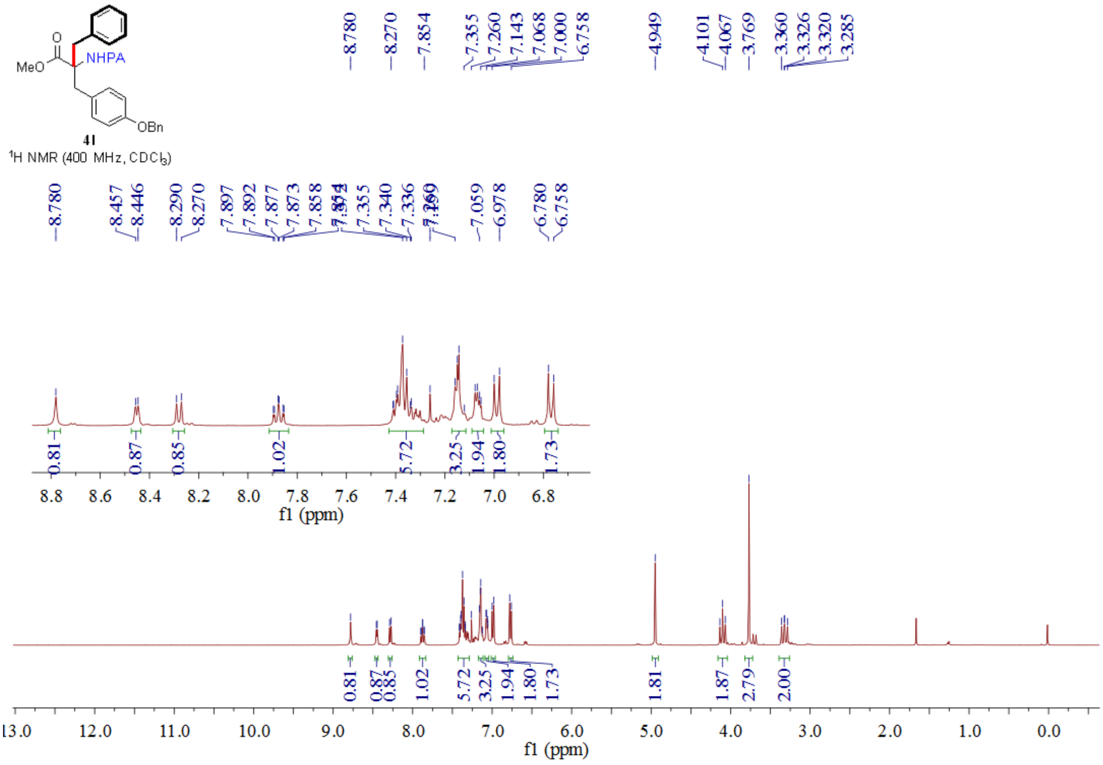

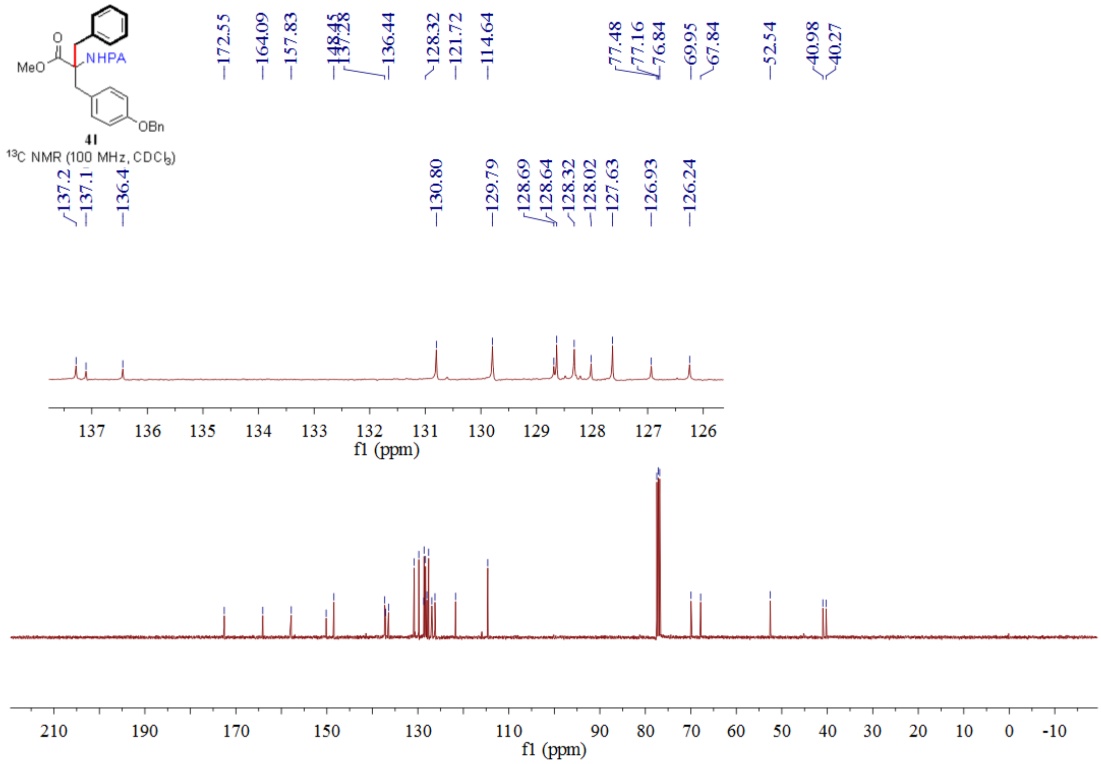

Supplementary Figure 73. ^{1}H NMR and ^{13}C NMR spectra for compound 4l

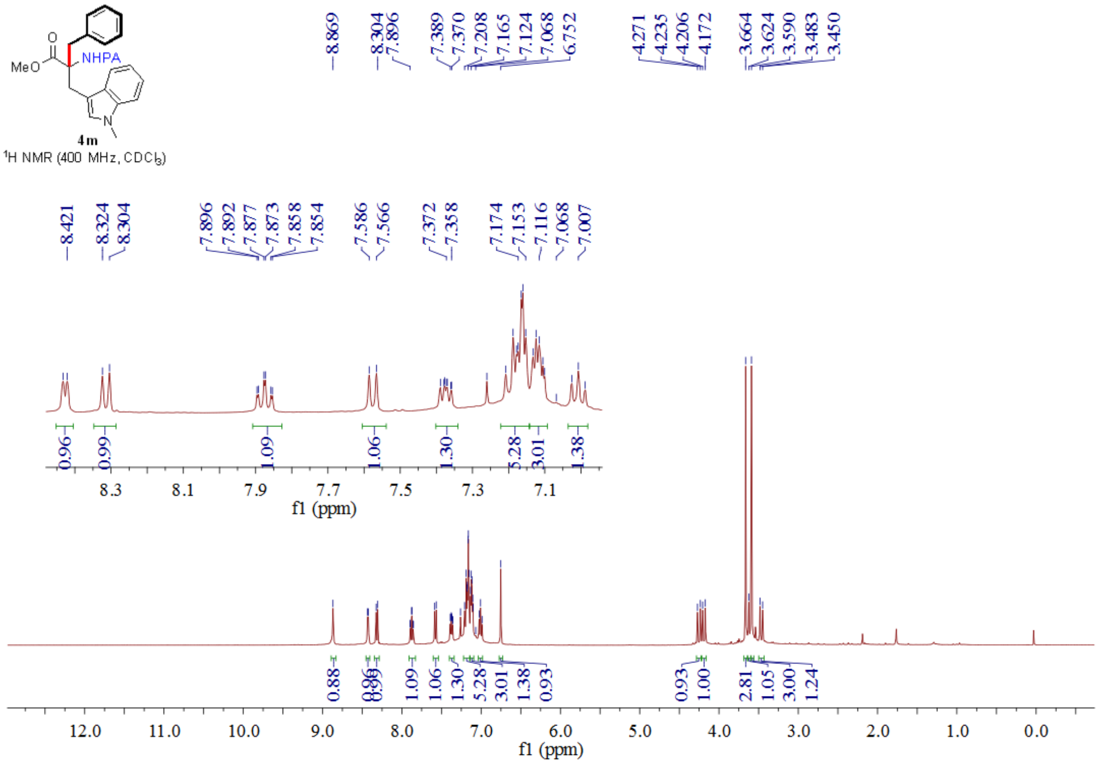

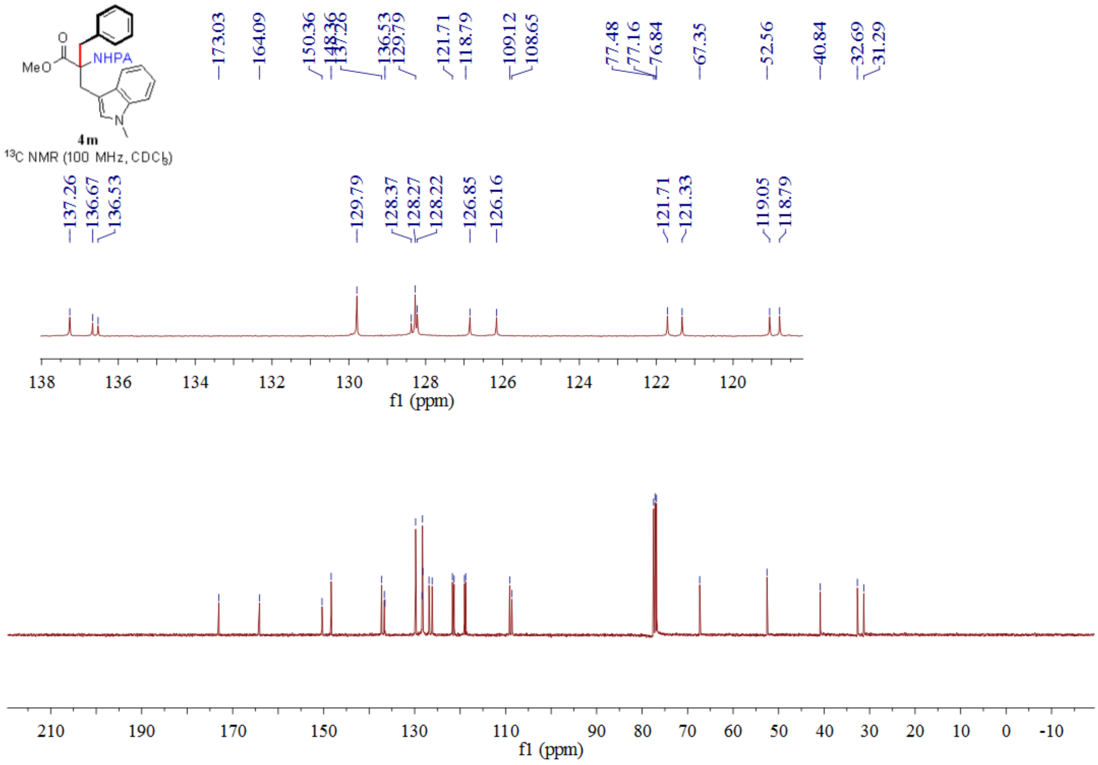

Supplementary Figure 74. ^{1}H NMR and ^{13}C NMR spectra for compound 4m

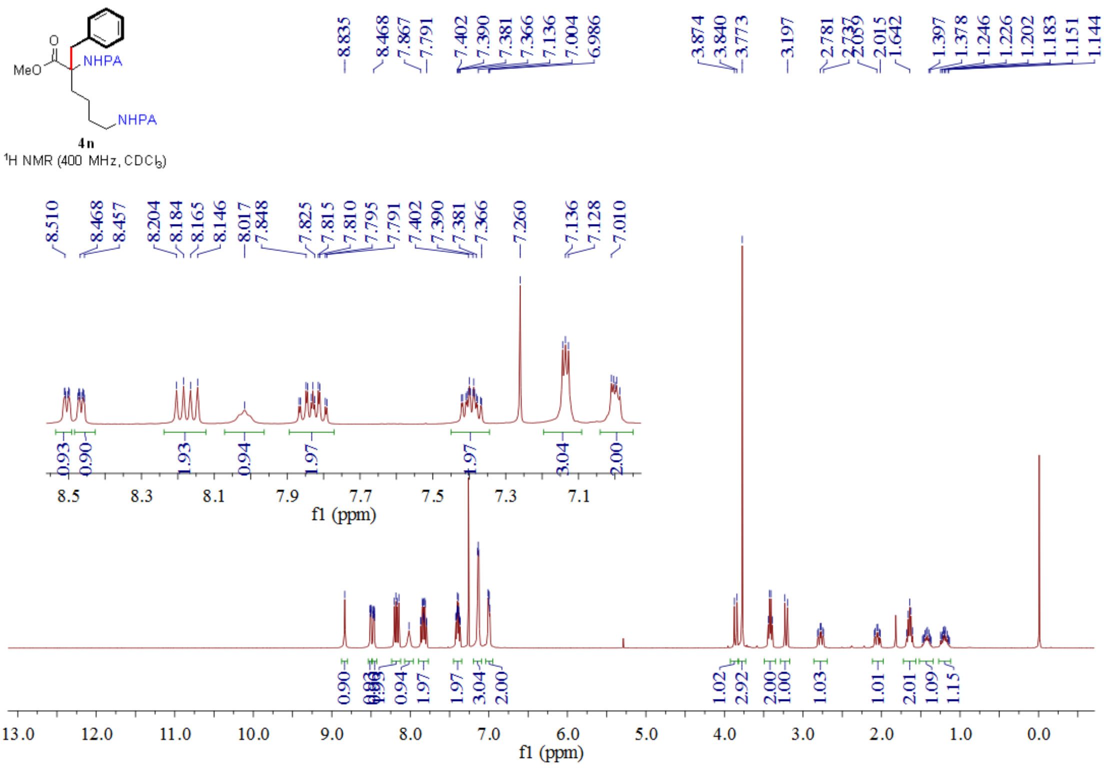

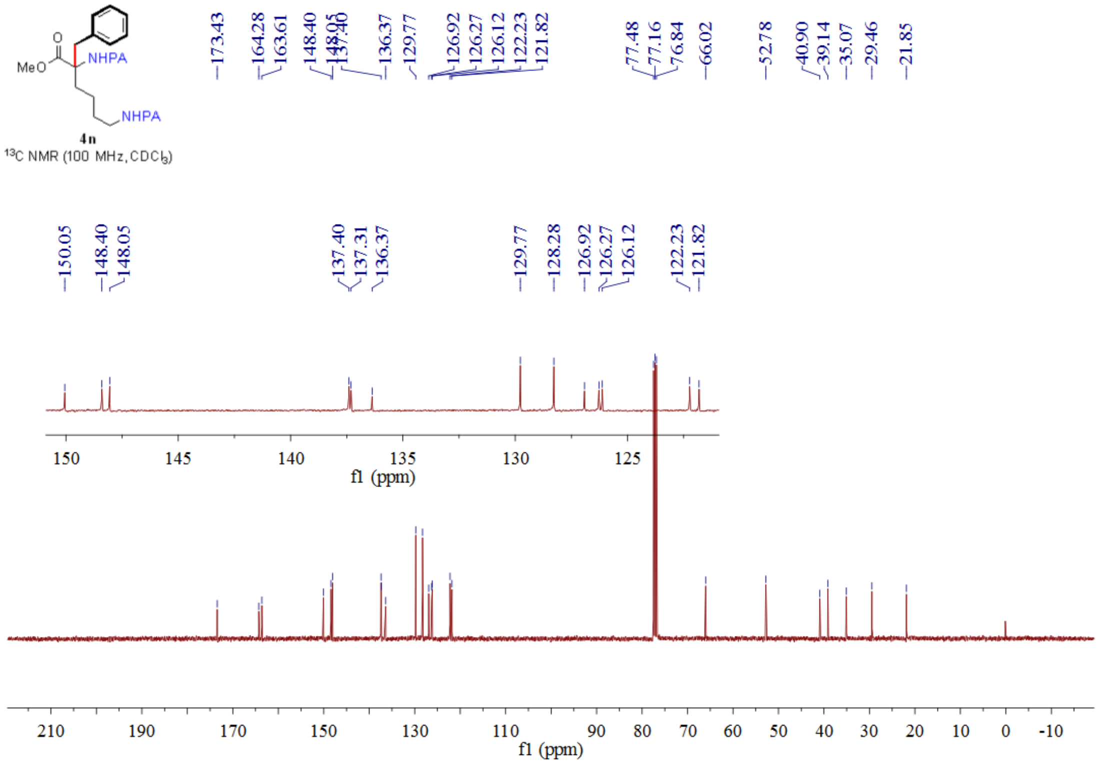

Supplementary Figure 75. ^{1}H NMR and ^{13}C NMR spectra for compound 4n

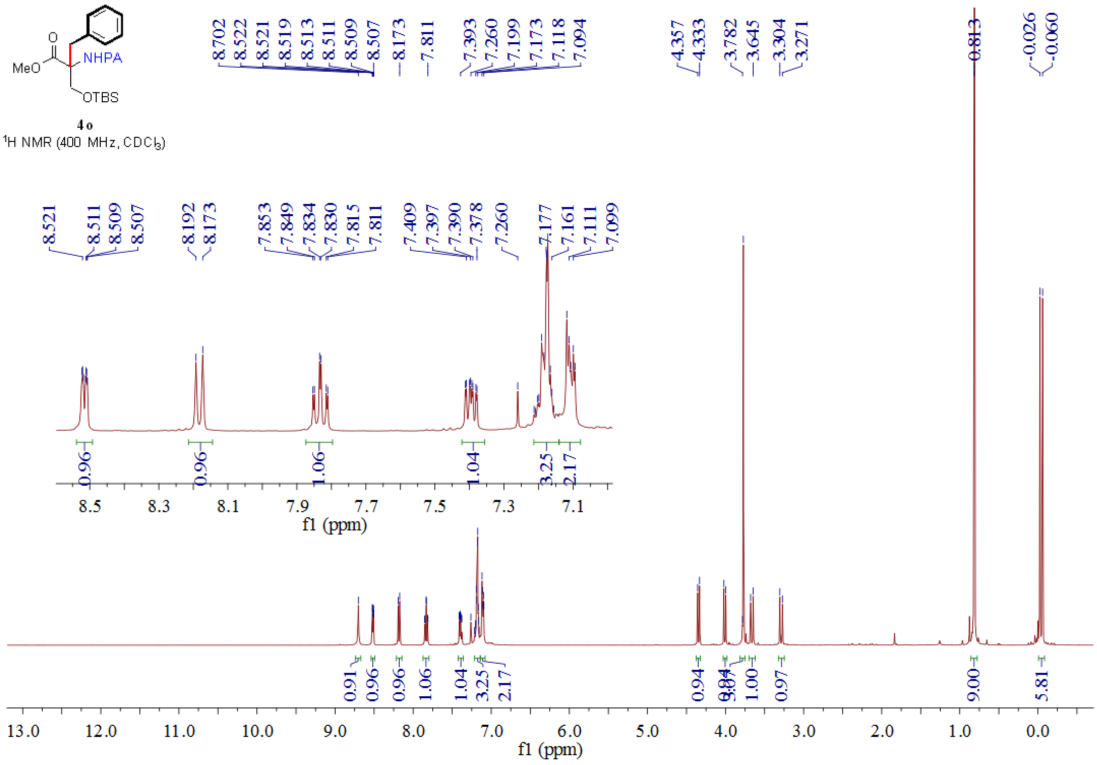

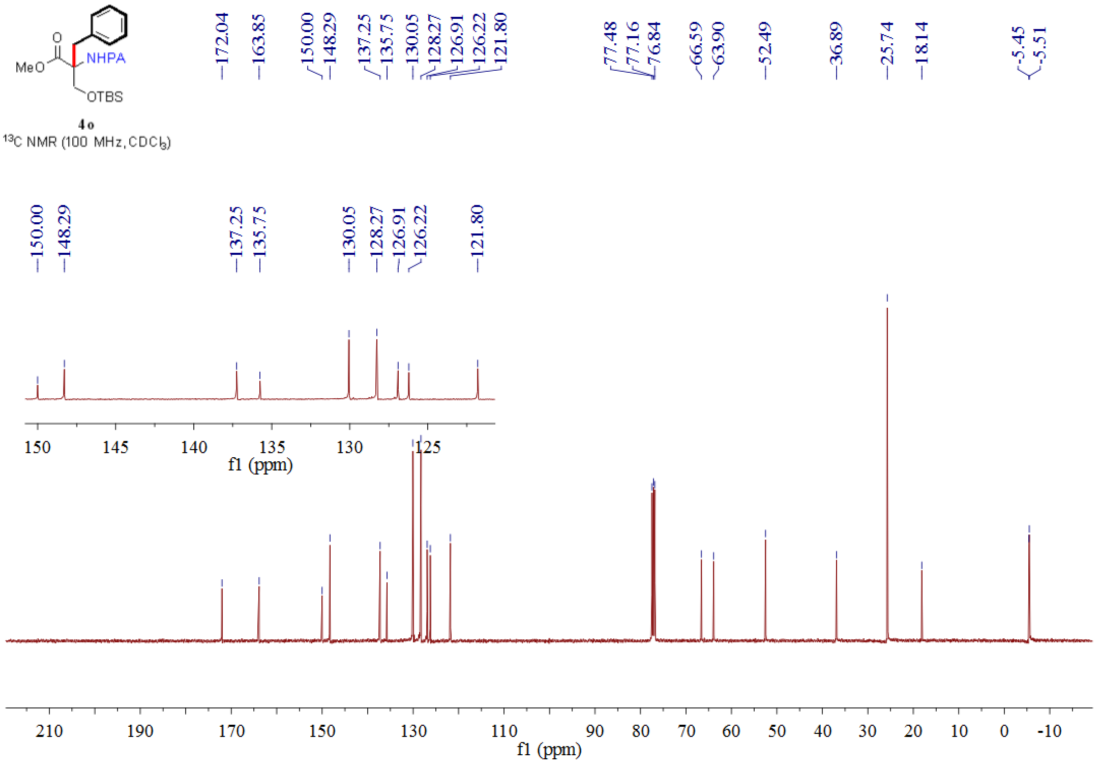

Supplementary Figure 76. ^{1}H NMR and ^{13}C NMR spectra for compound 4o

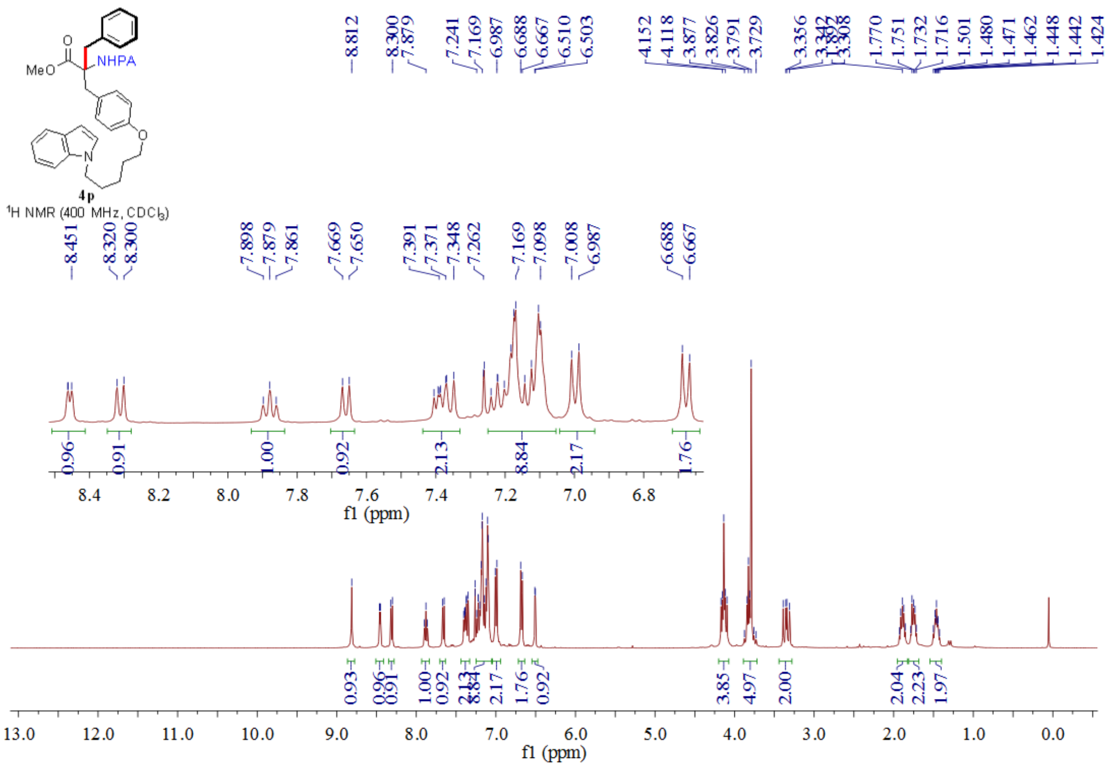

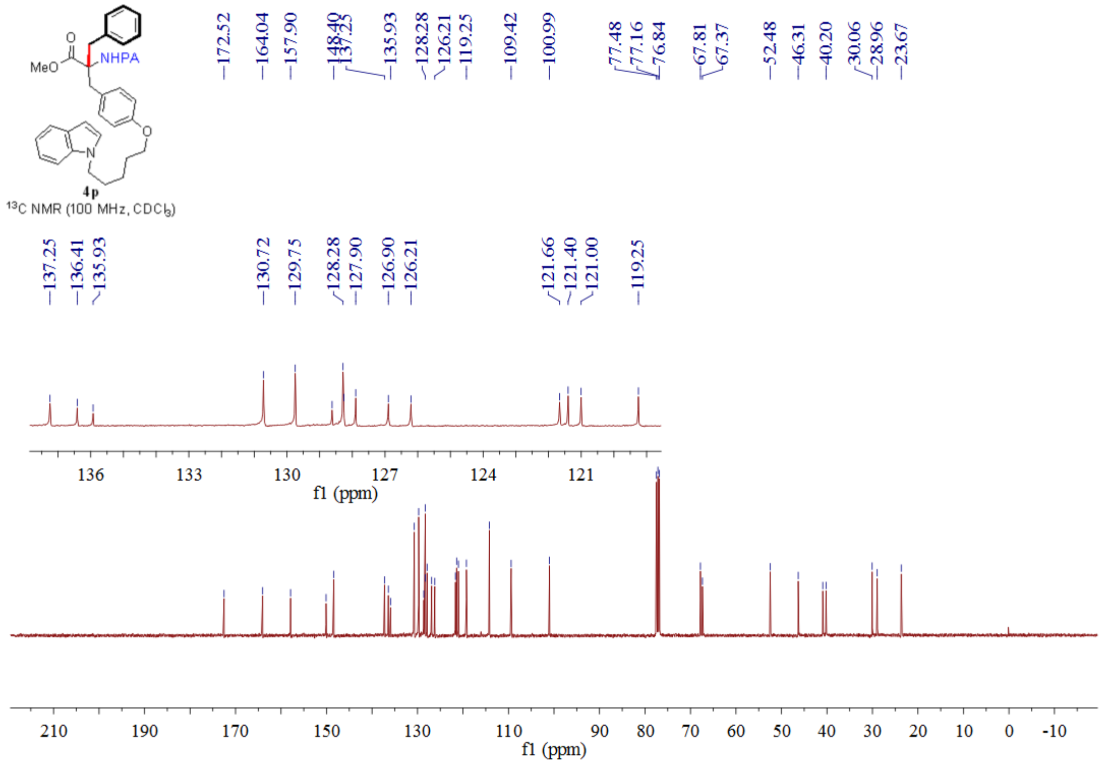

Supplementary Figure 77. ^{1}H NMR and ^{13}C NMR spectra for compound 4p

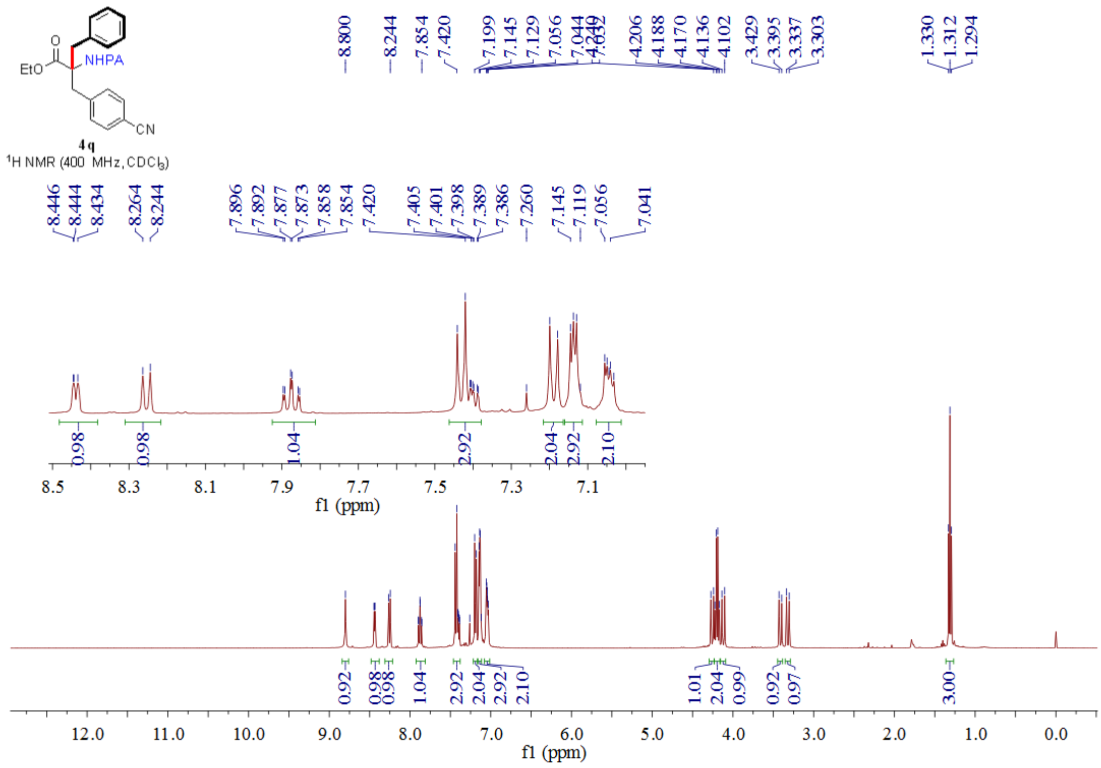

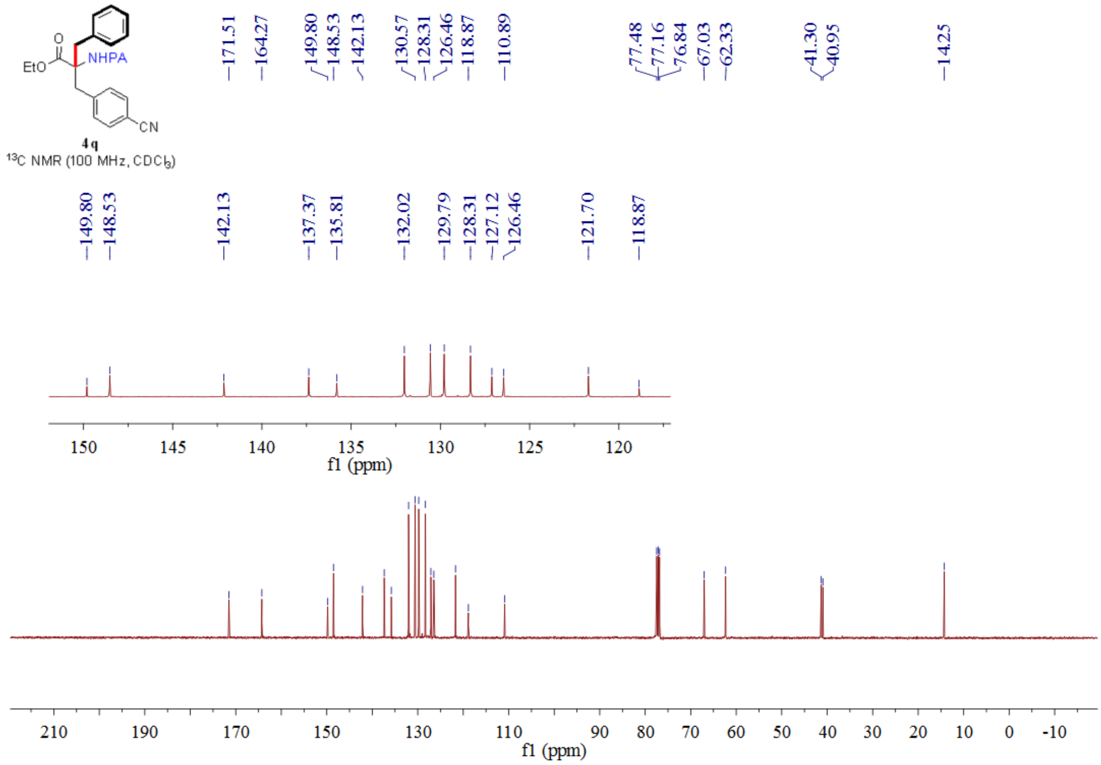

Supplementary Figure 78. ^{1}H NMR and ^{13}C NMR spectra for compound 4q

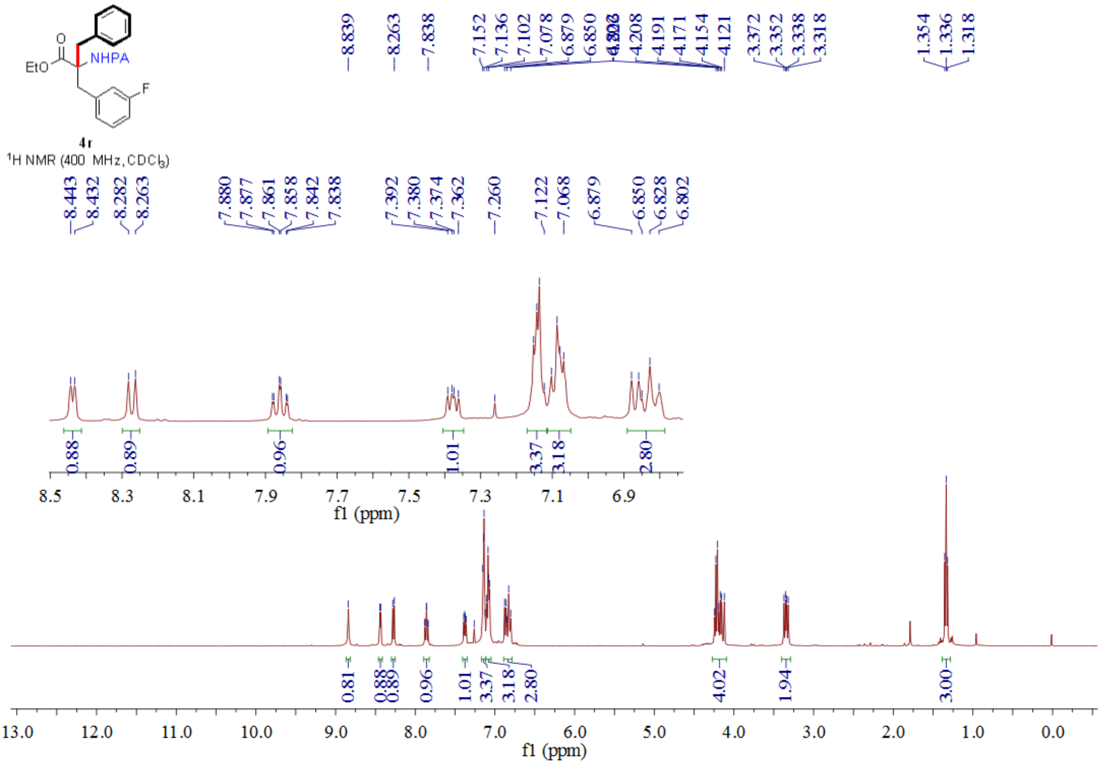

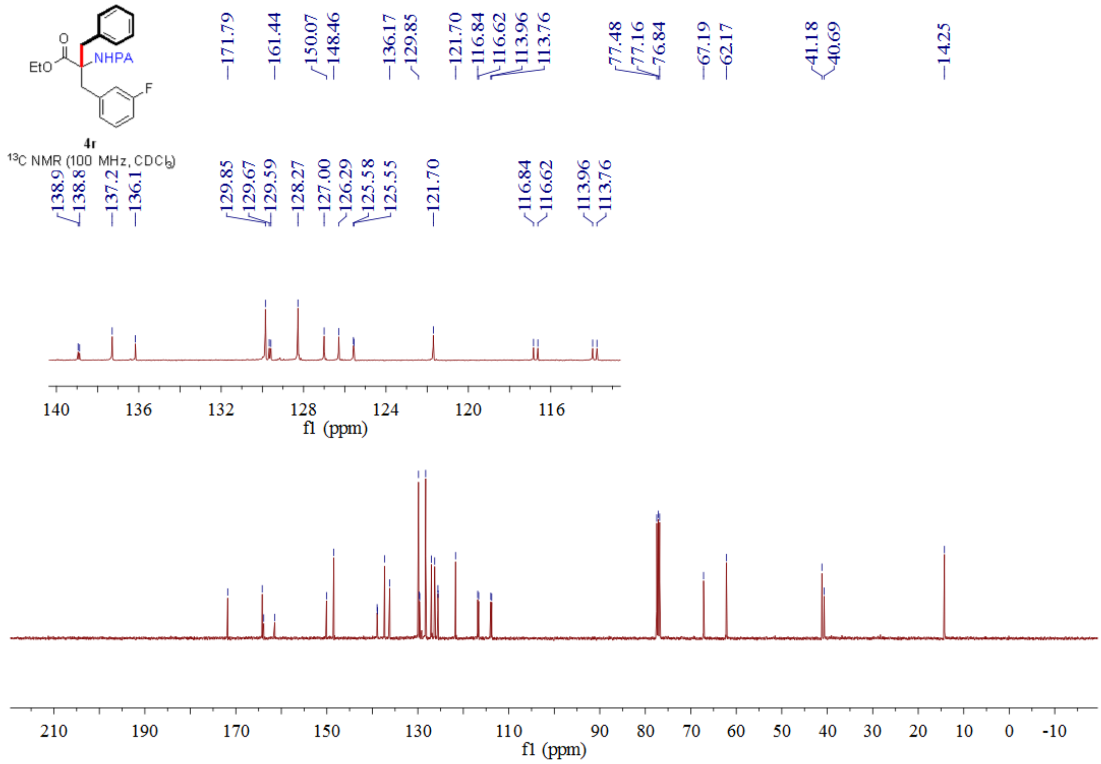

Supplementary Figure 79. ^{1}H NMR and ^{13}C NMR spectra for compound 4r

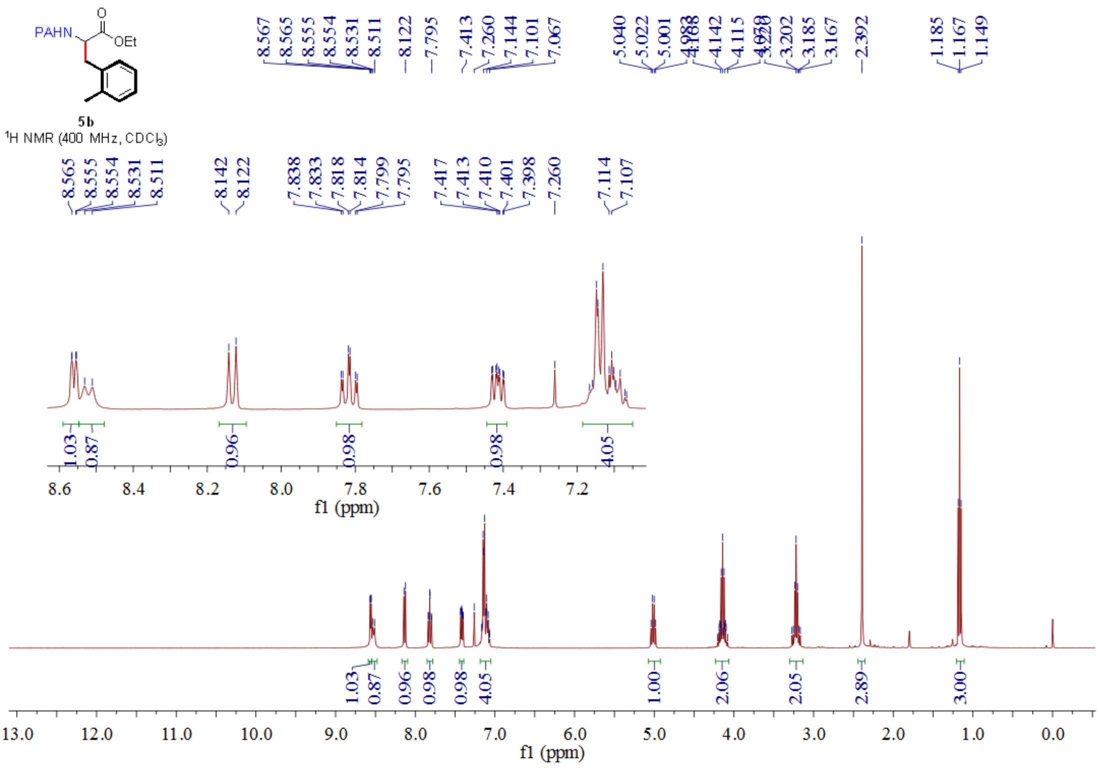

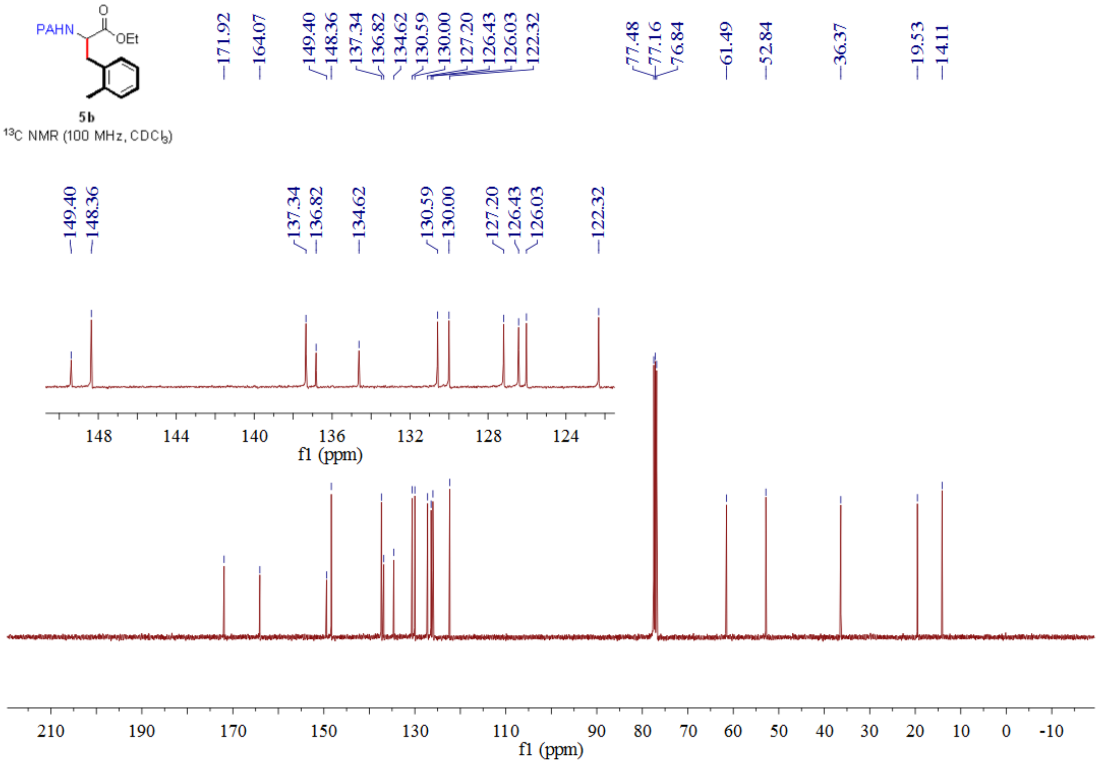

Supplementary Figure 80. ^{1}**H NMR and** ^{13}**C NMR spectra for compound 5b**

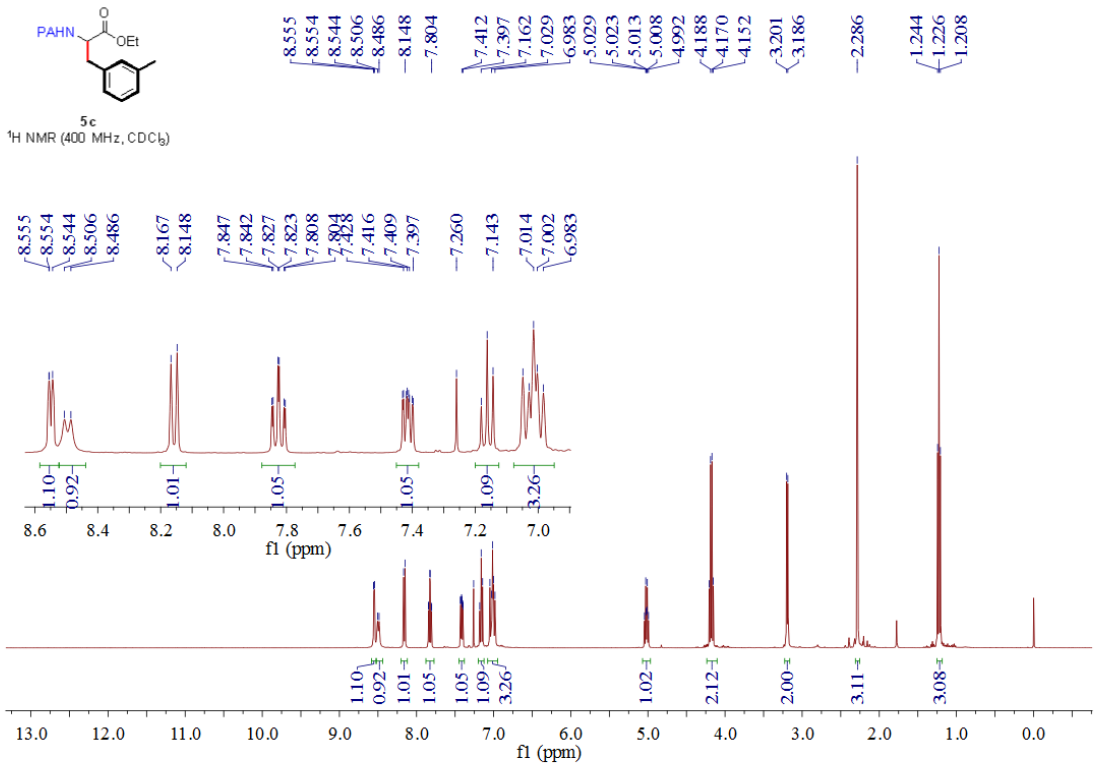

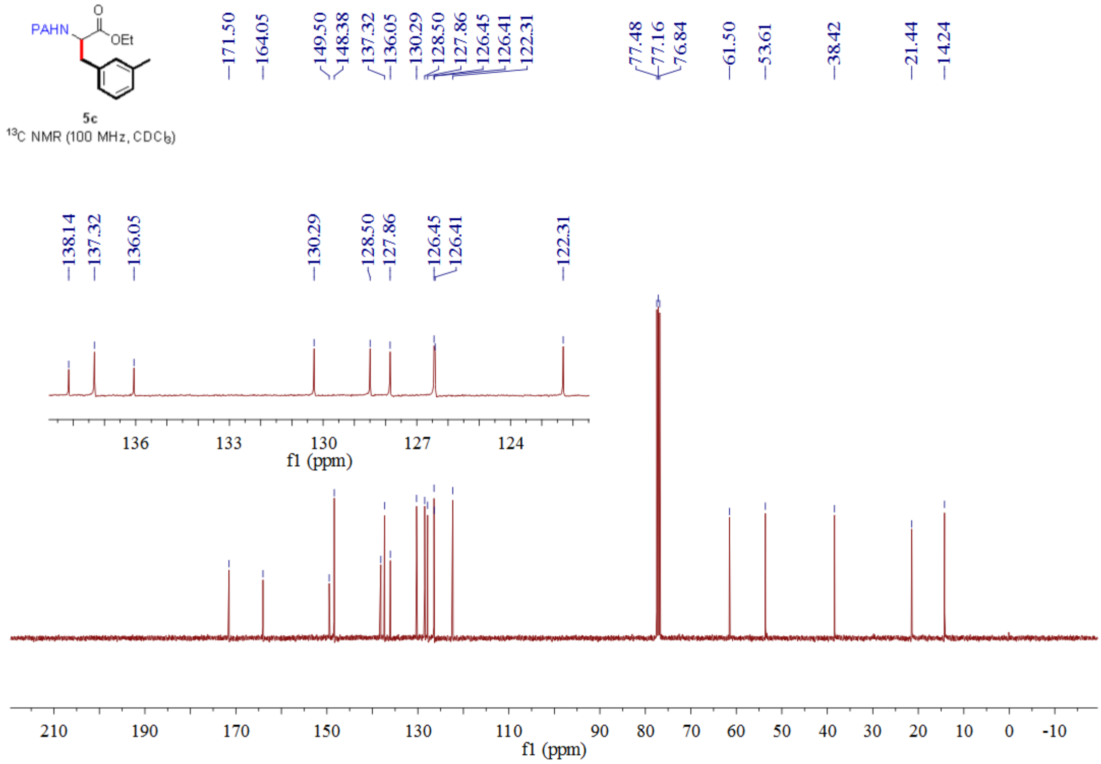

Supplementary Figure 81. ^{1}H NMR and ^{13}C NMR spectra for compound 5c

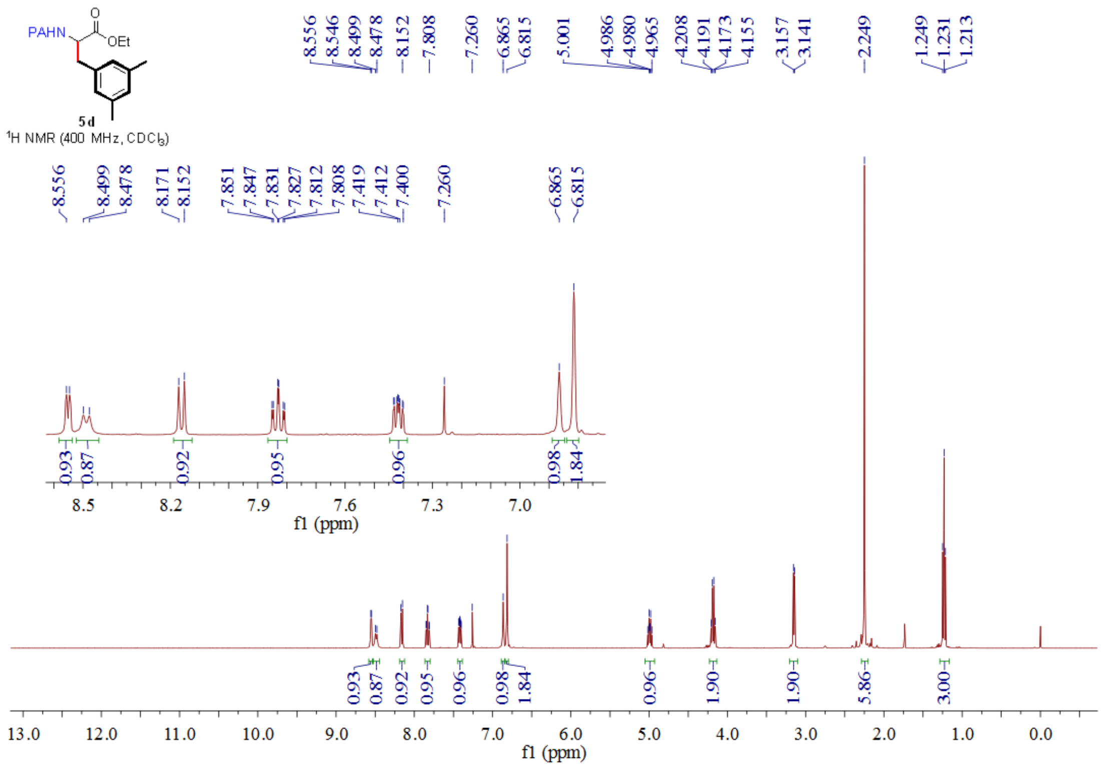

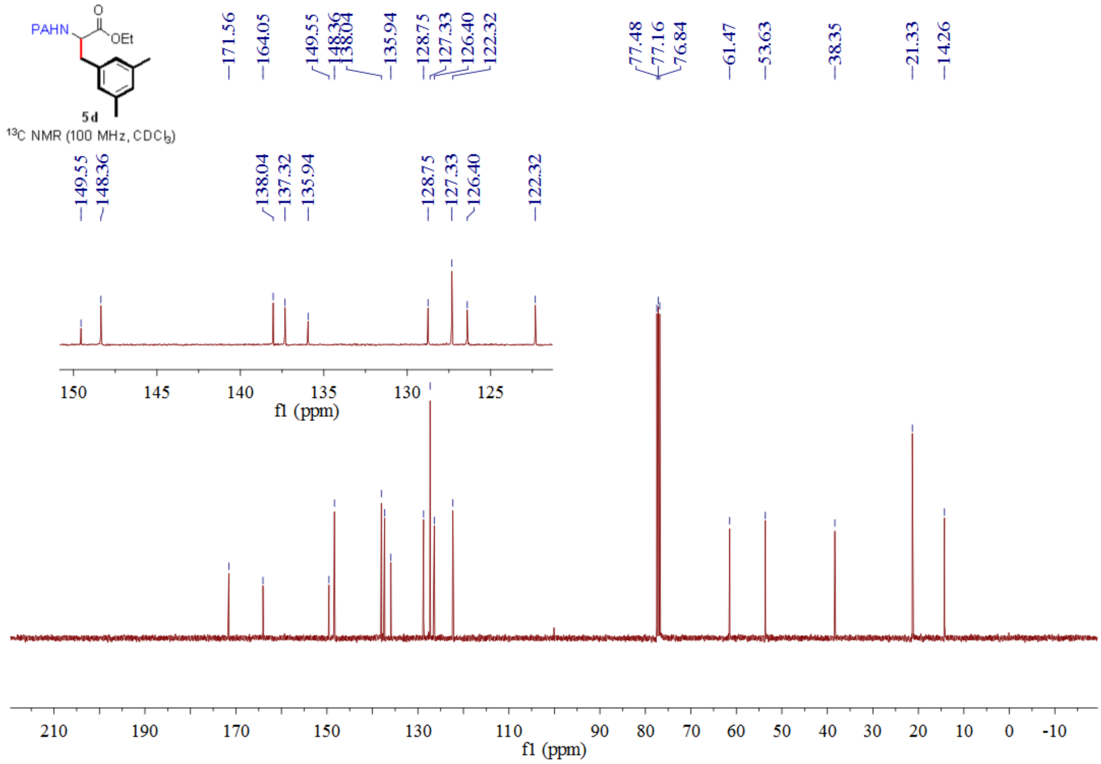

Supplementary Figure 82. ^{1}H **NMR and** ^{13}C **NMR spectra for compound 5d**

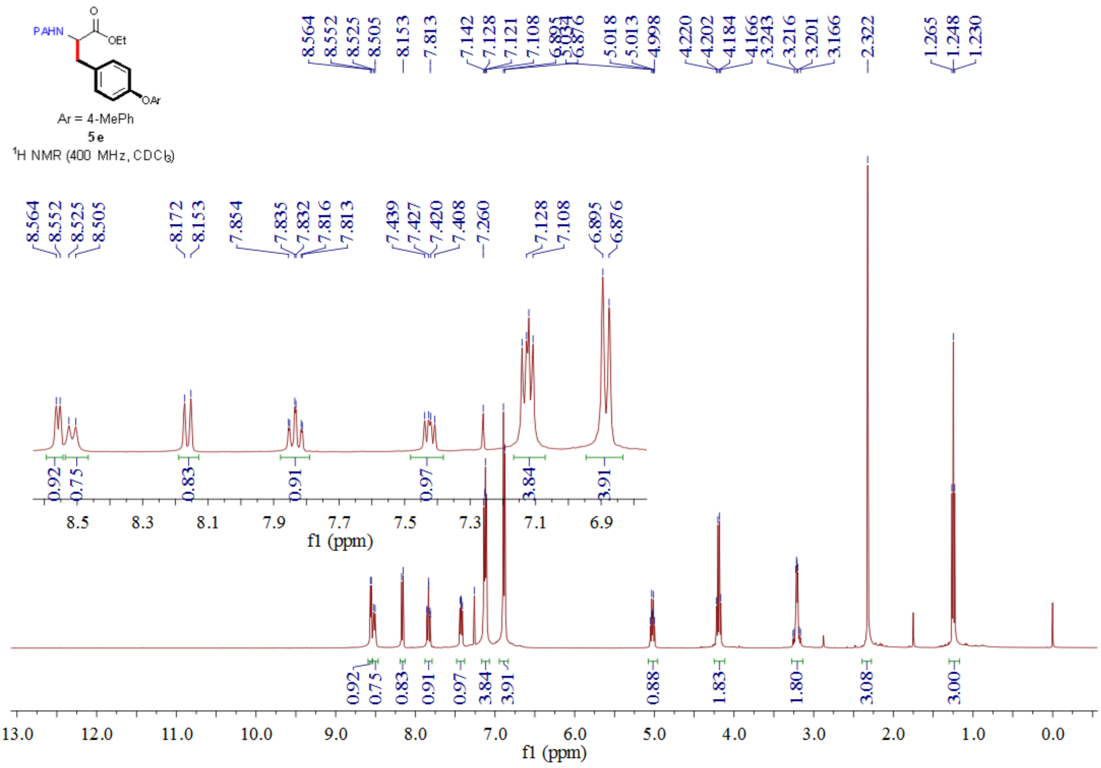

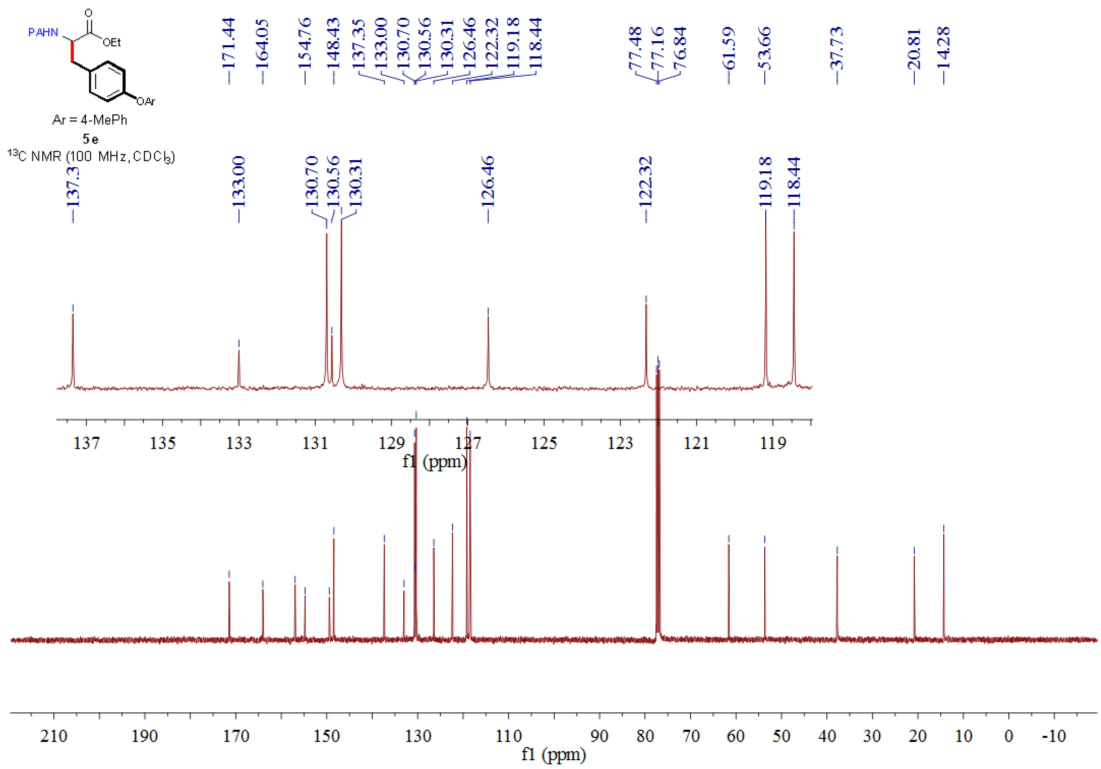

Supplementary Figure 83. ^{1}**H NMR and** ^{13}**C NMR spectra for compound 5e**

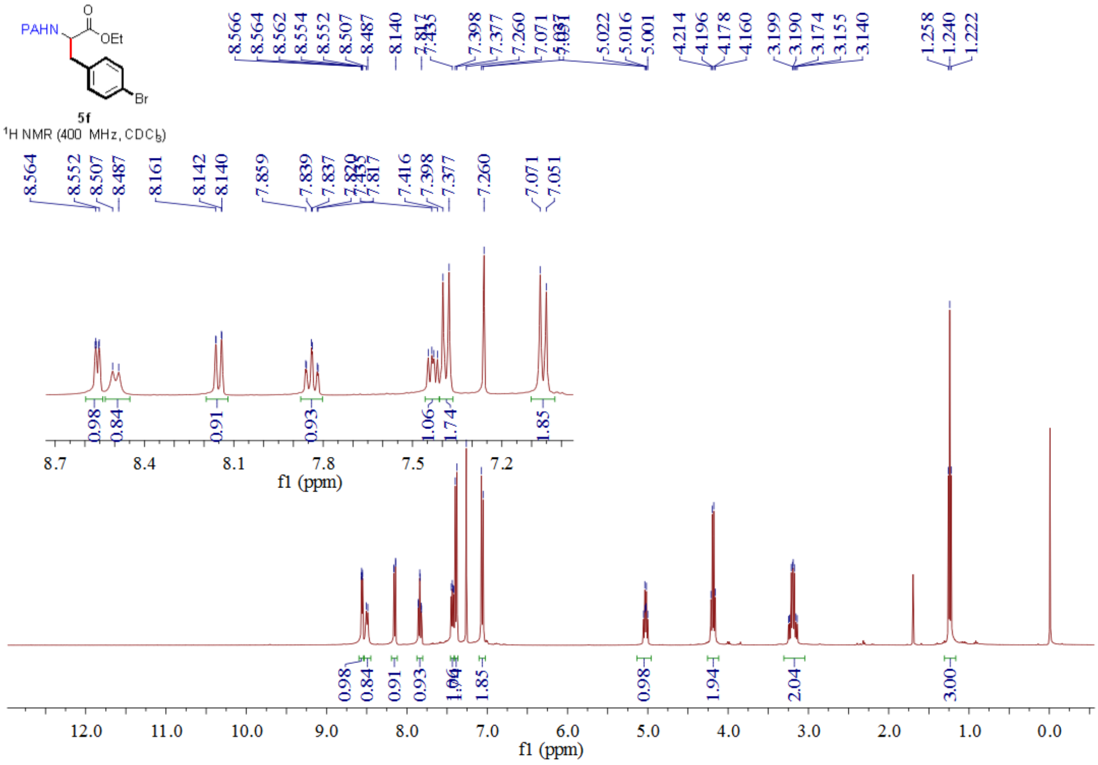

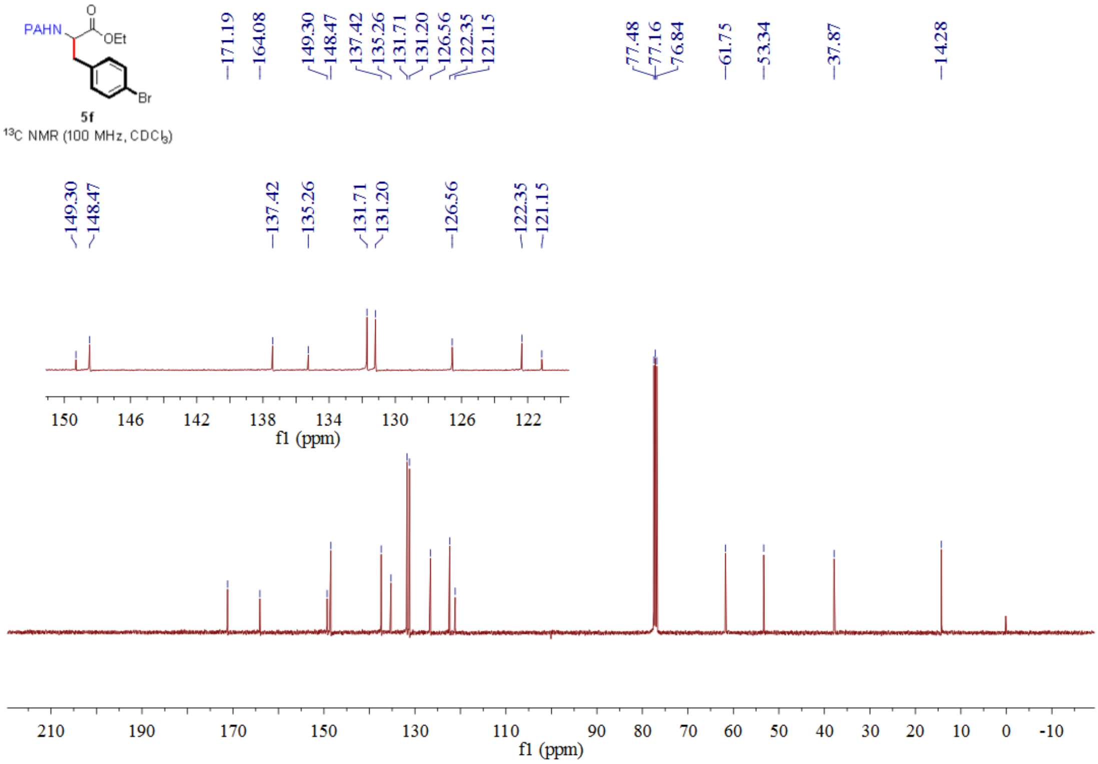

Supplementary Figure 84. ^{1}H NMR and ^{13}C NMR spectra for compound 5f

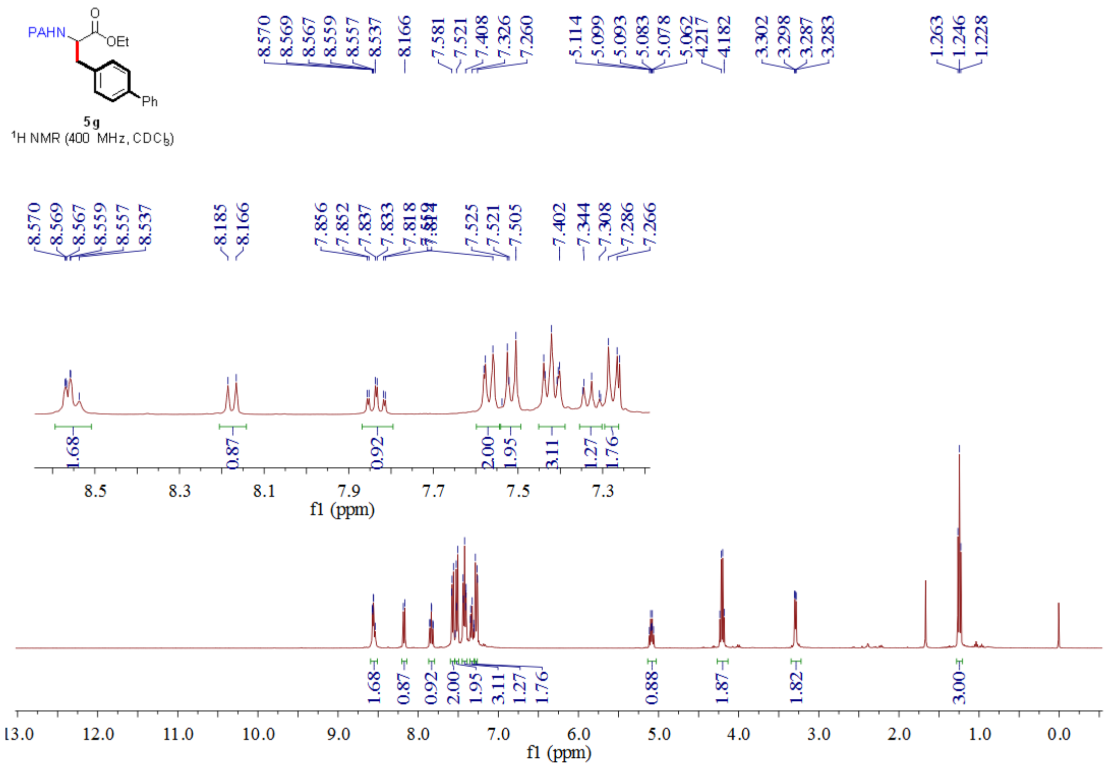

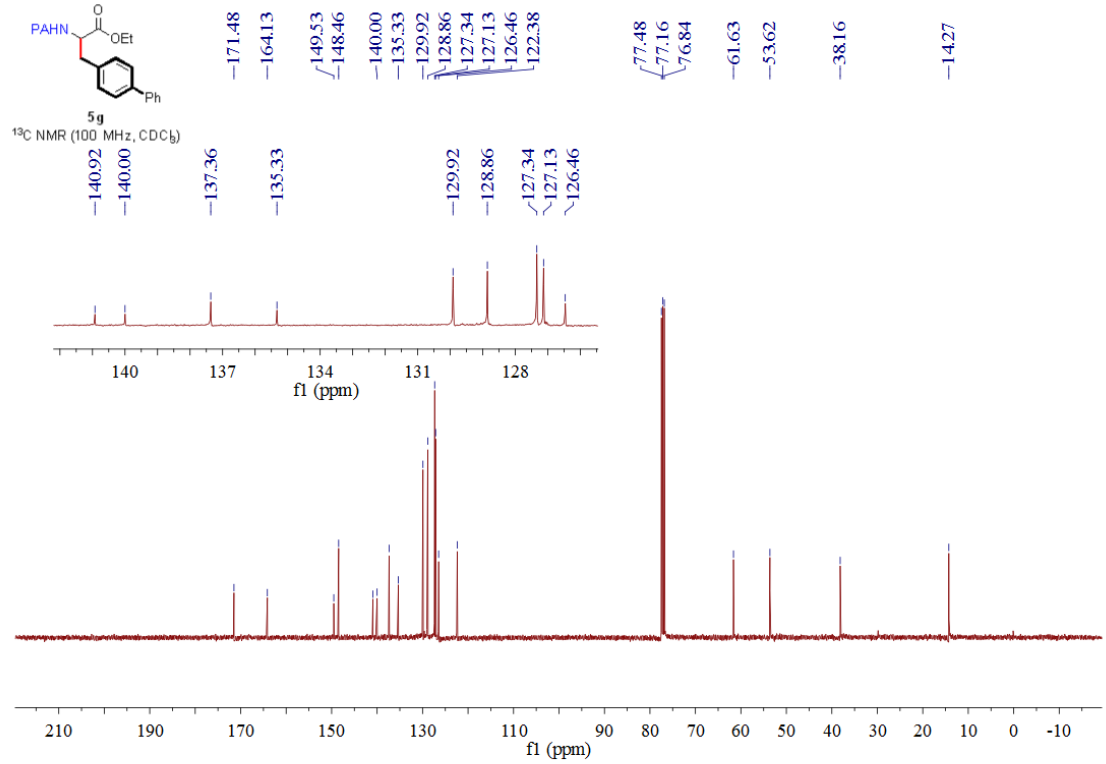

Supplementary Figure 85. ^{1}H NMR and ^{13}C NMR spectra for compound 5g

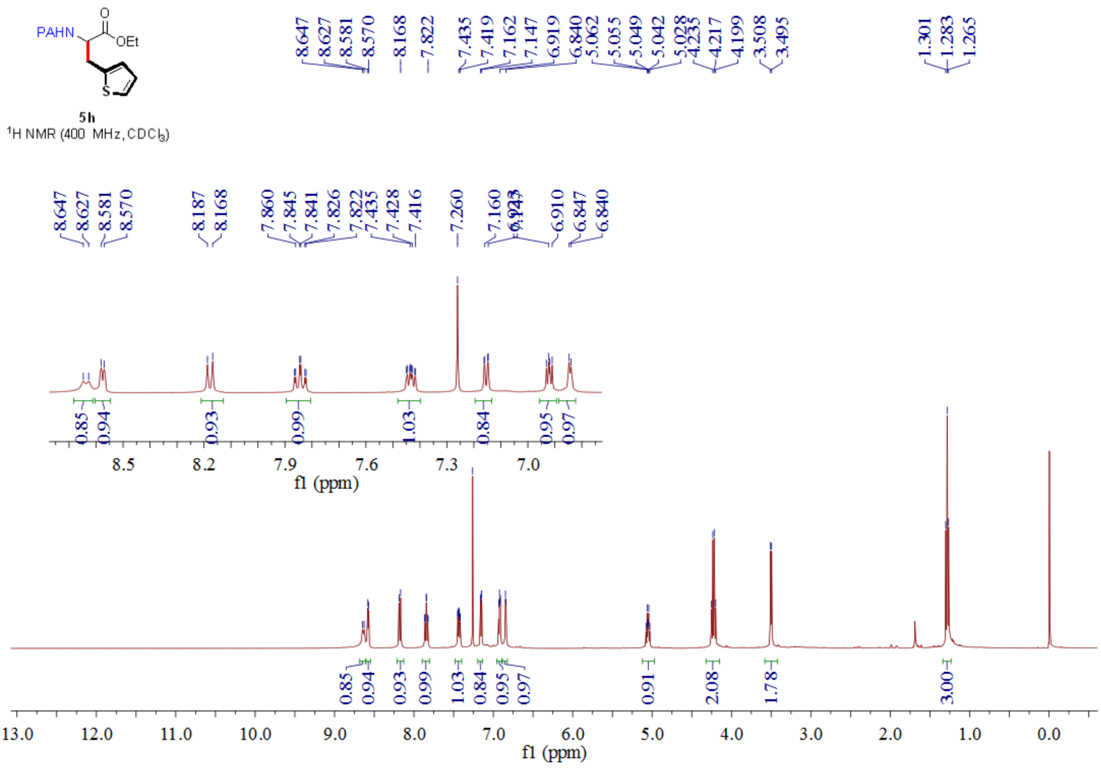

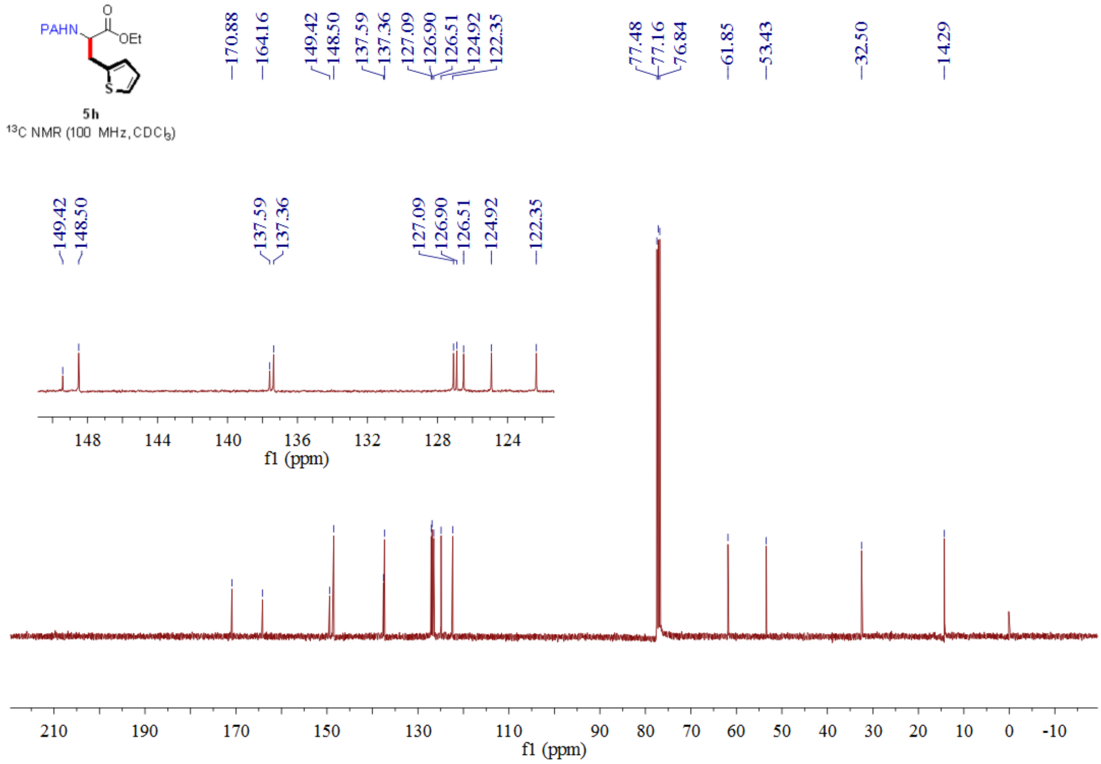

Supplementary Figure 86. ^{1}H NMR and ^{13}C NMR spectra for compound 5h

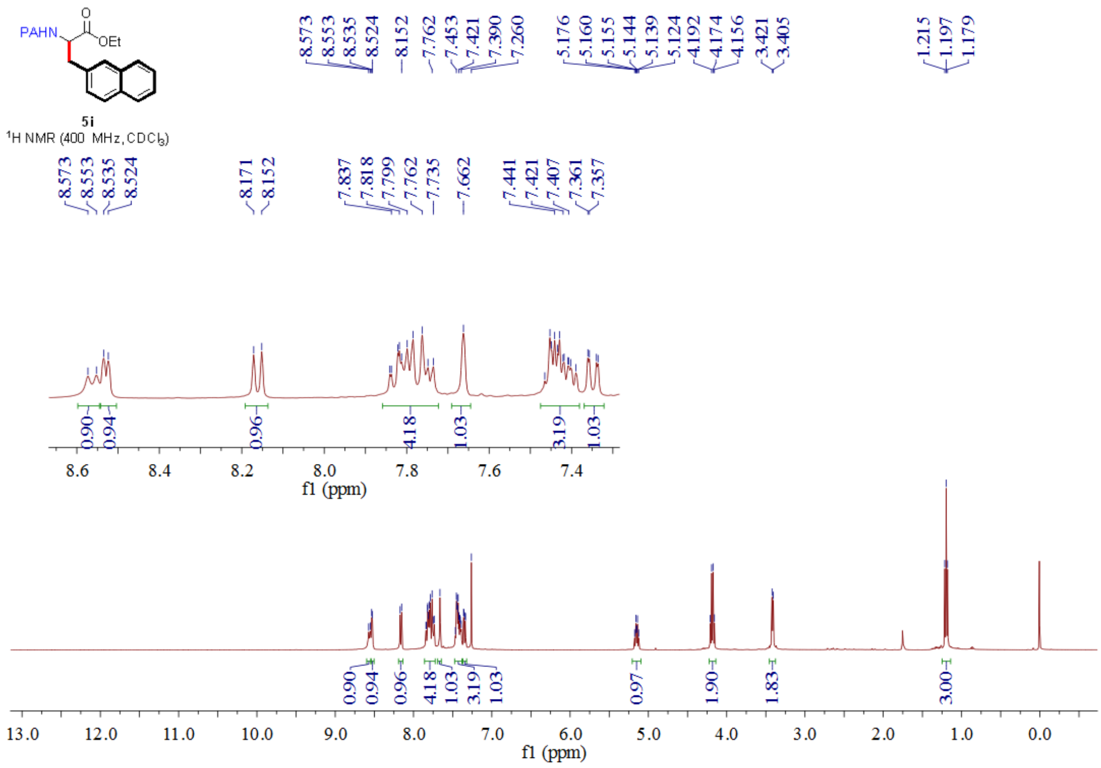

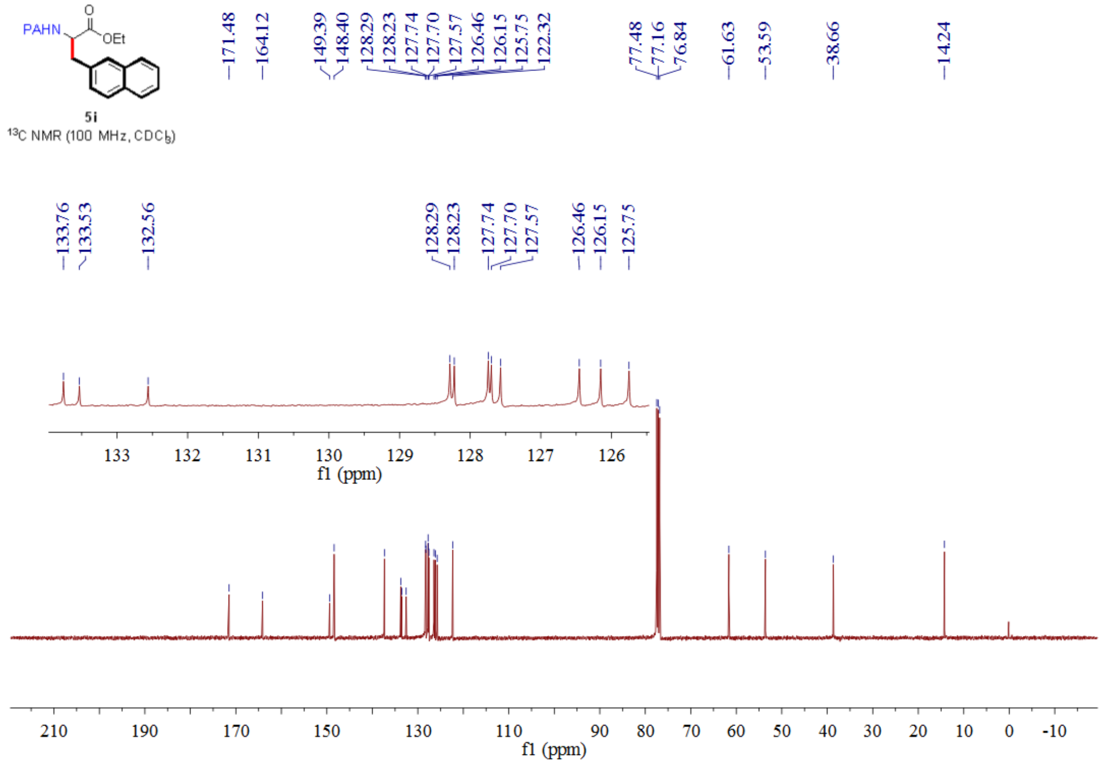

Supplementary Figure 87. ^{1}H NMR and ^{13}C NMR spectra for compound 5i

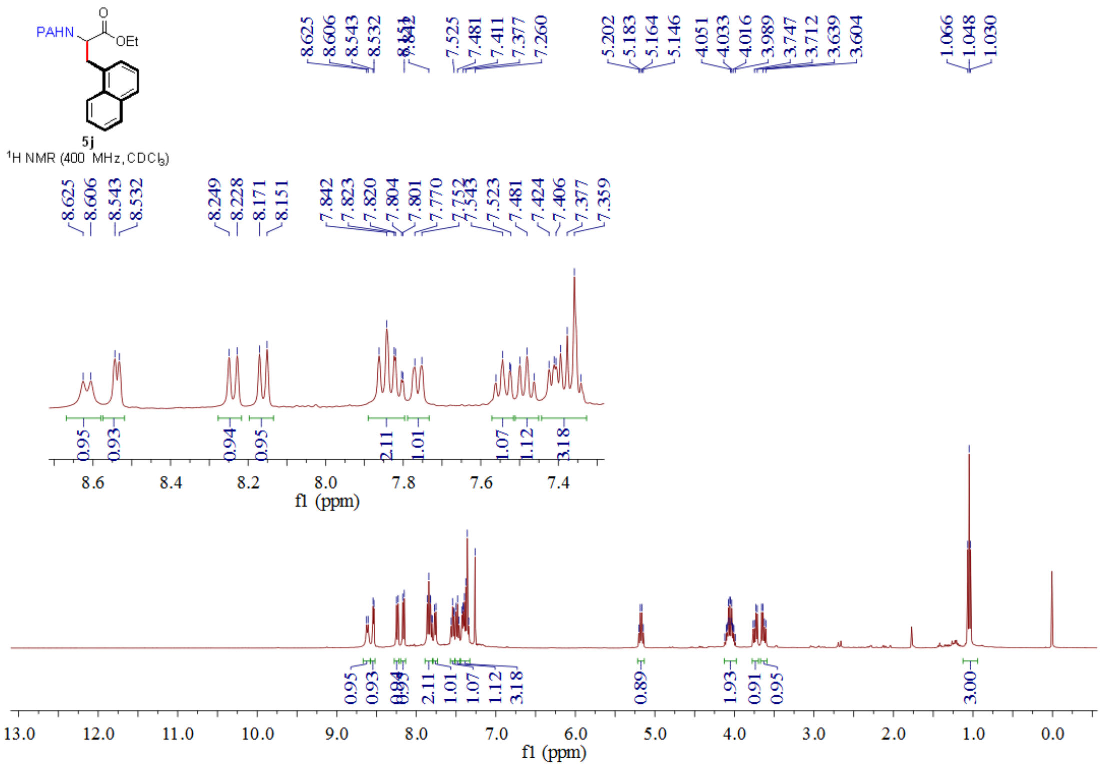

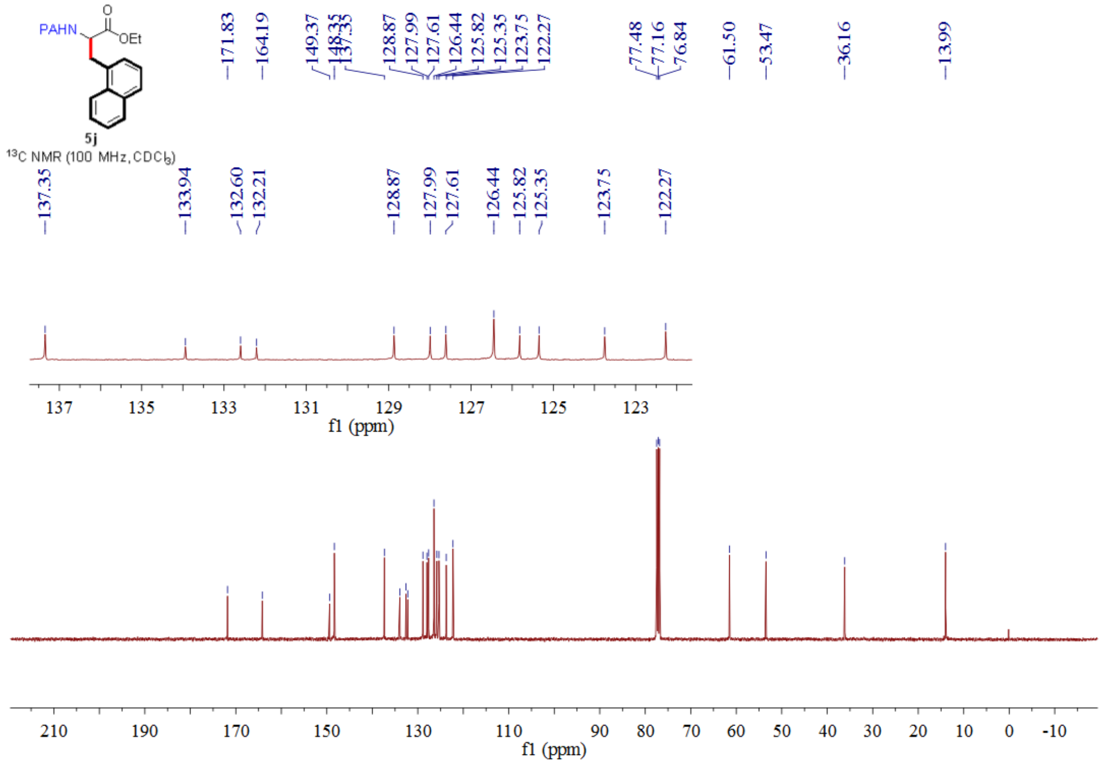

Supplementary Figure 88. ^{1}H NMR and ^{13}C NMR spectra for compound 5j

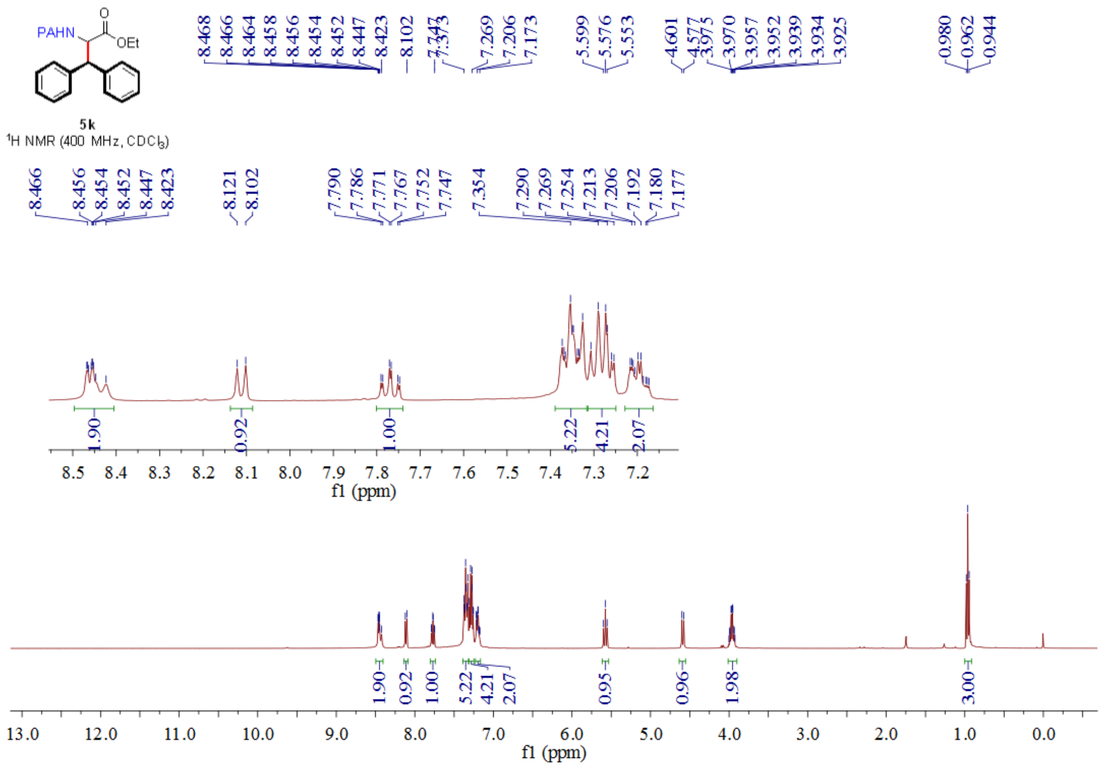

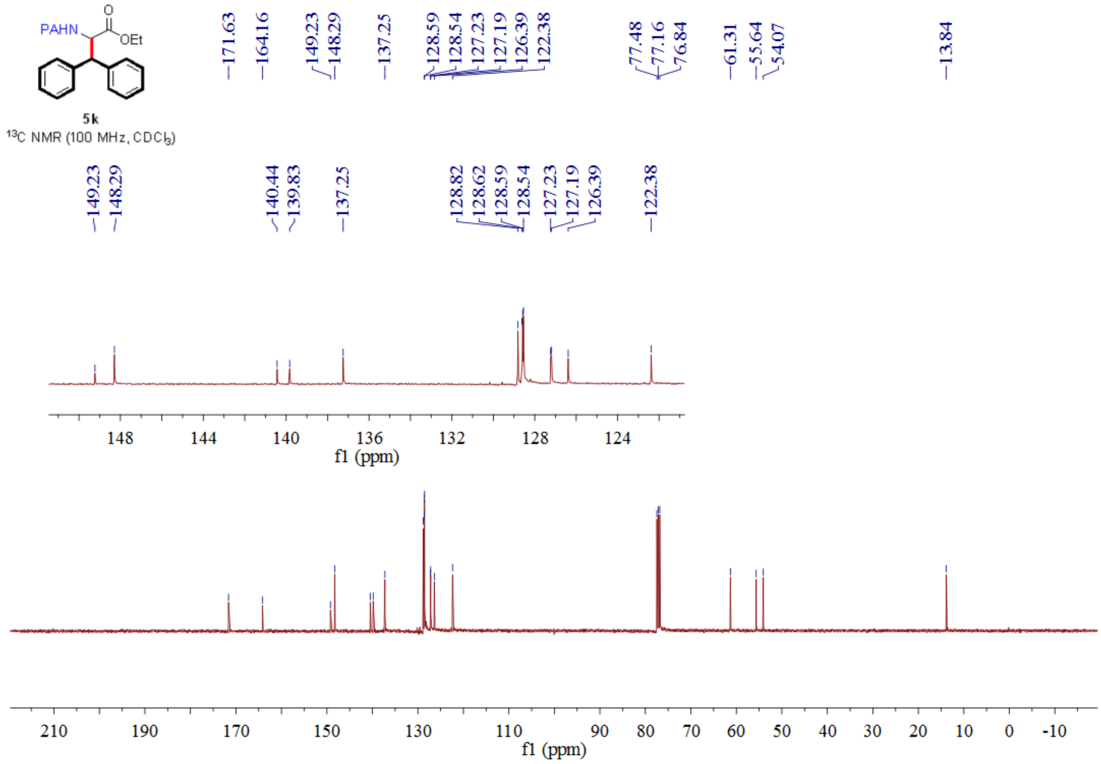

Supplementary Figure 89. ^{1}H NMR and ^{13}C NMR spectra for compound 5k

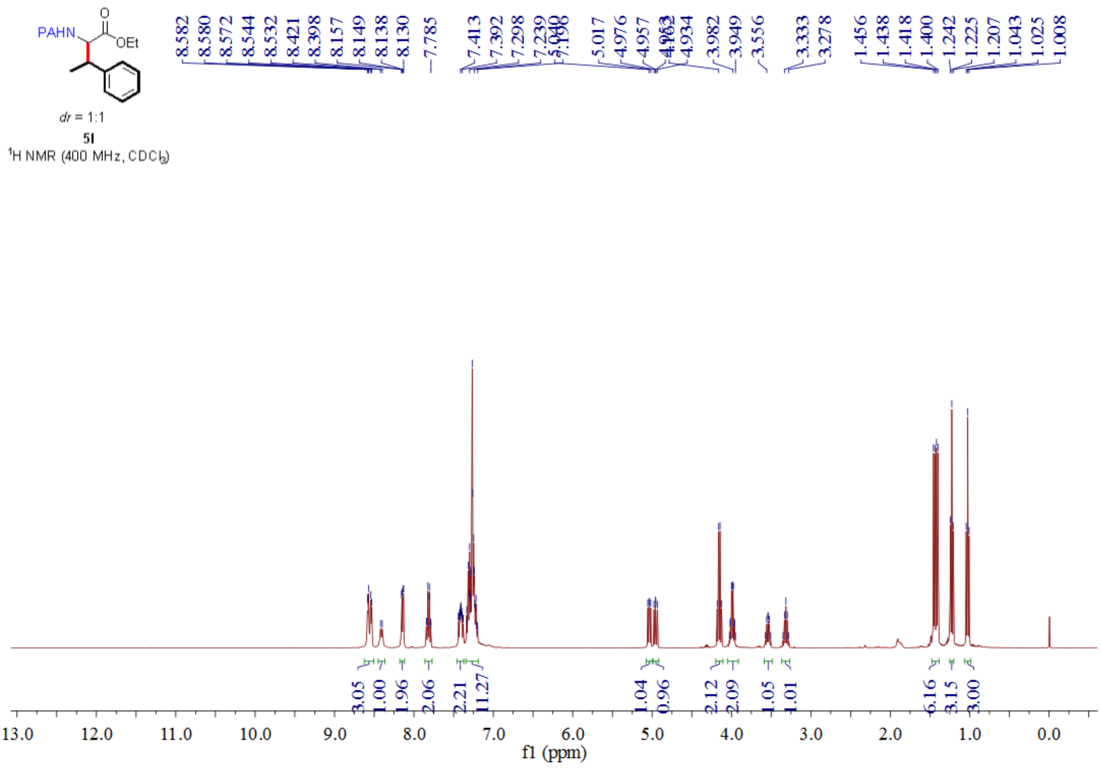

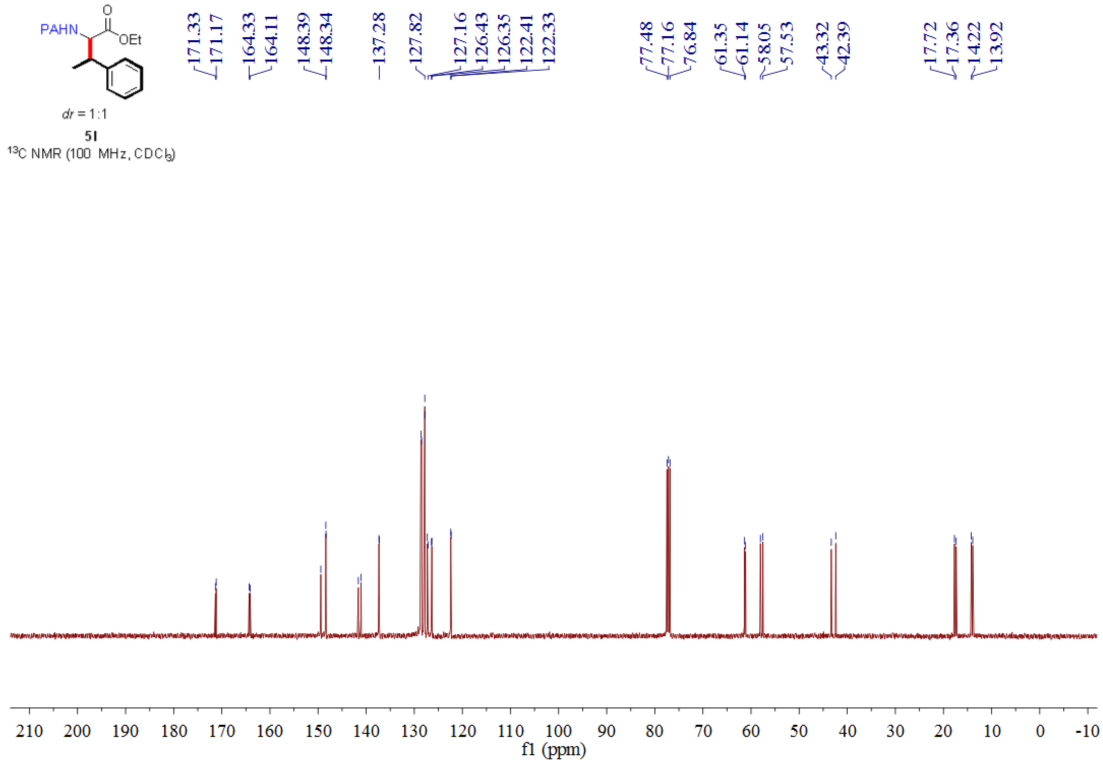

Supplementary Figure 90. ^{1}H NMR and ^{13}C NMR spectra for compound 5l

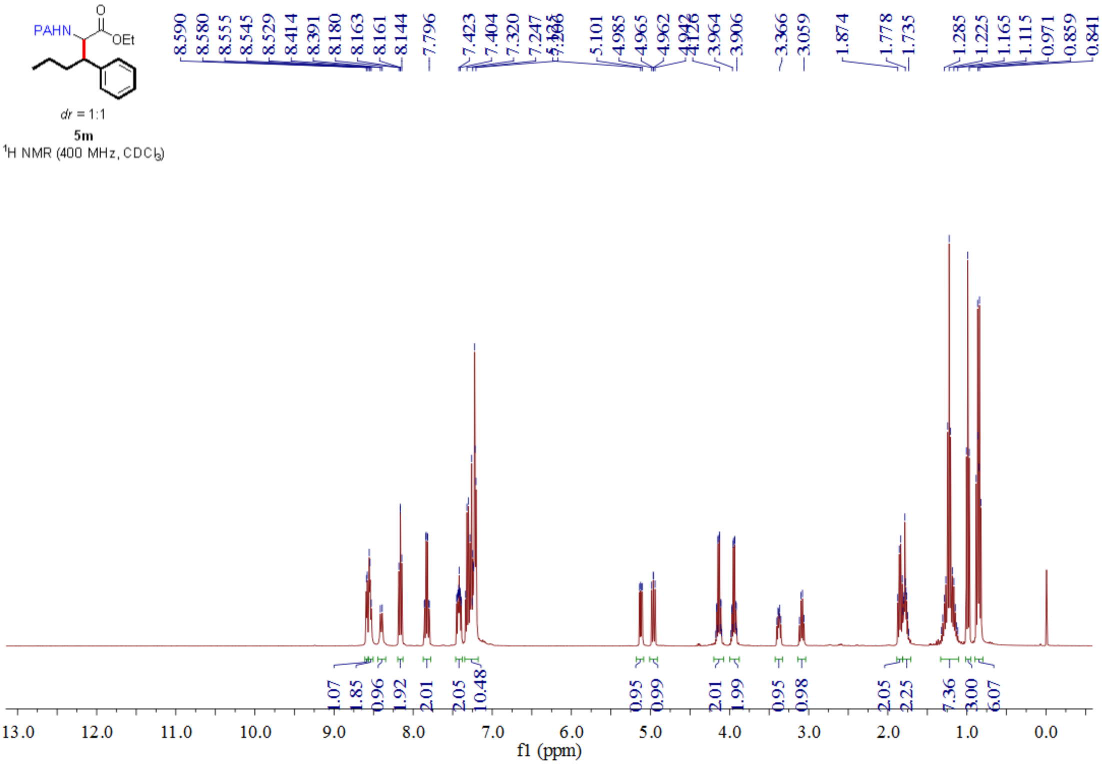

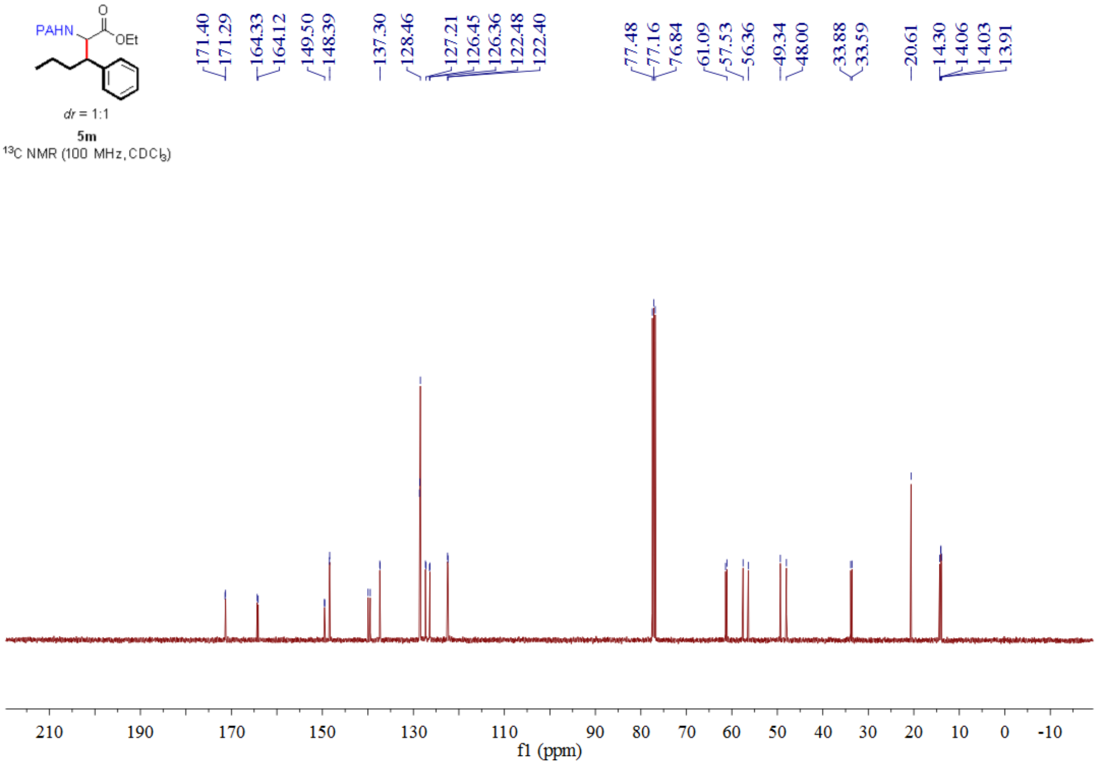

Supplementary Figure 91. ^{1}H NMR and ^{13}C NMR spectra for compound 5m

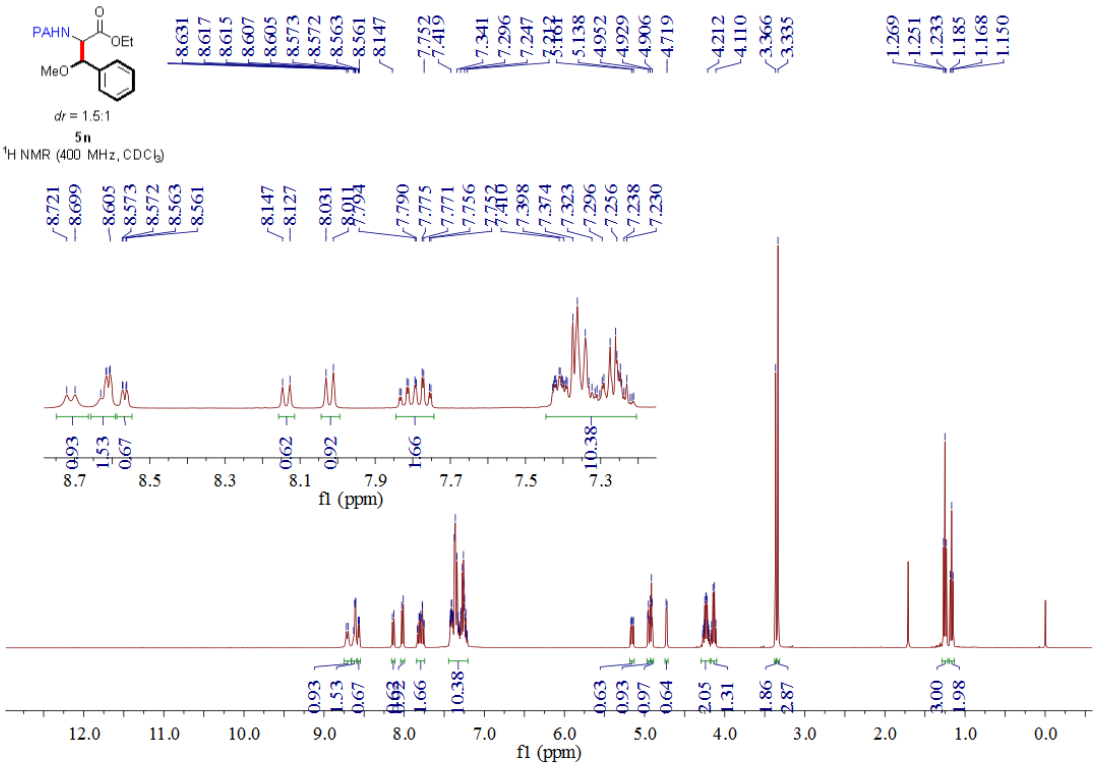

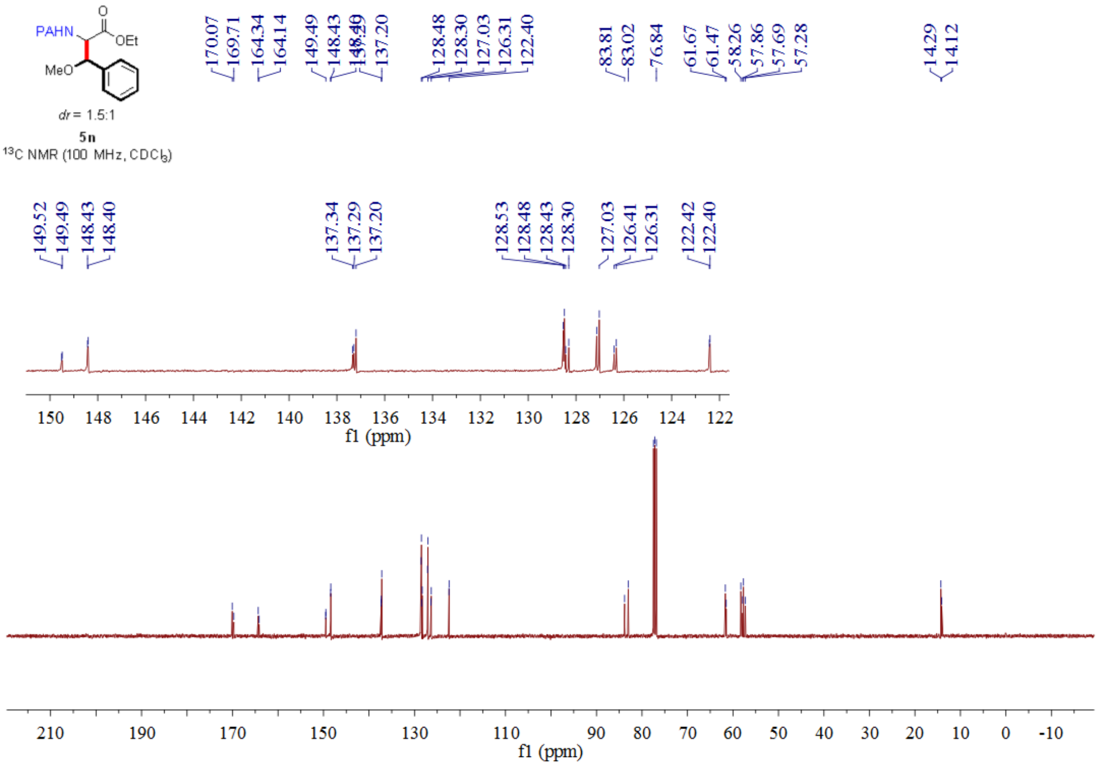

Supplementary Figure 92. ^{1}H NMR and ^{13}C NMR spectra for compound 5n

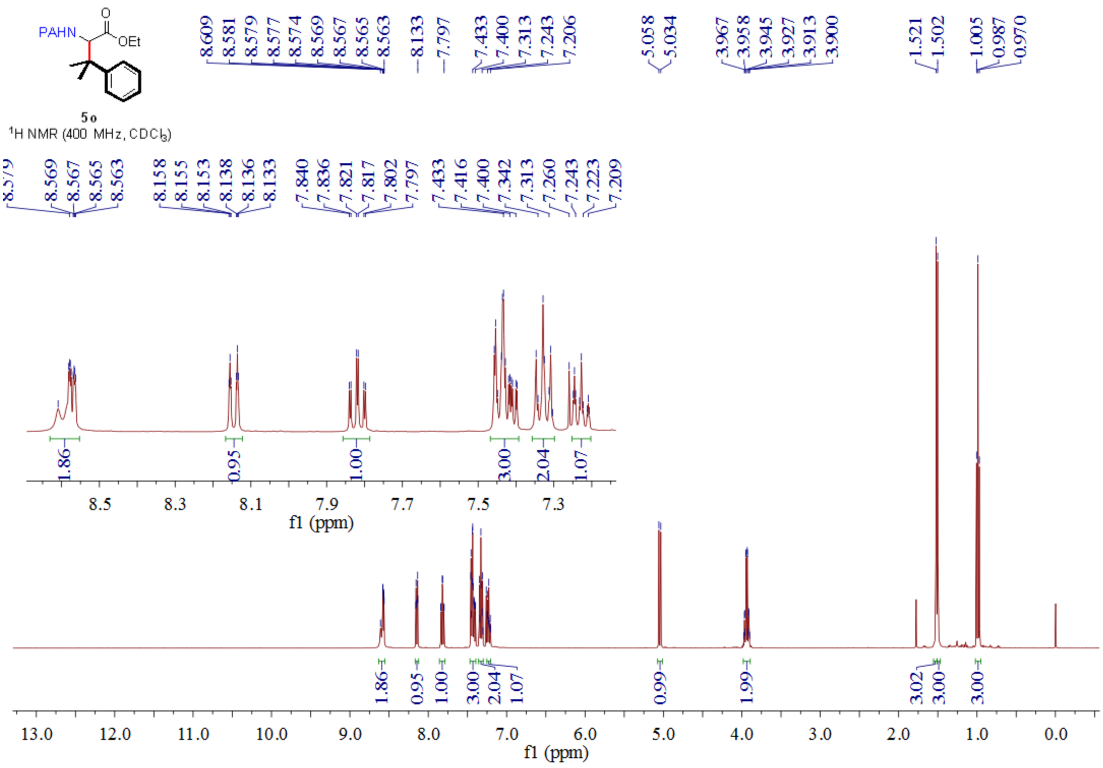

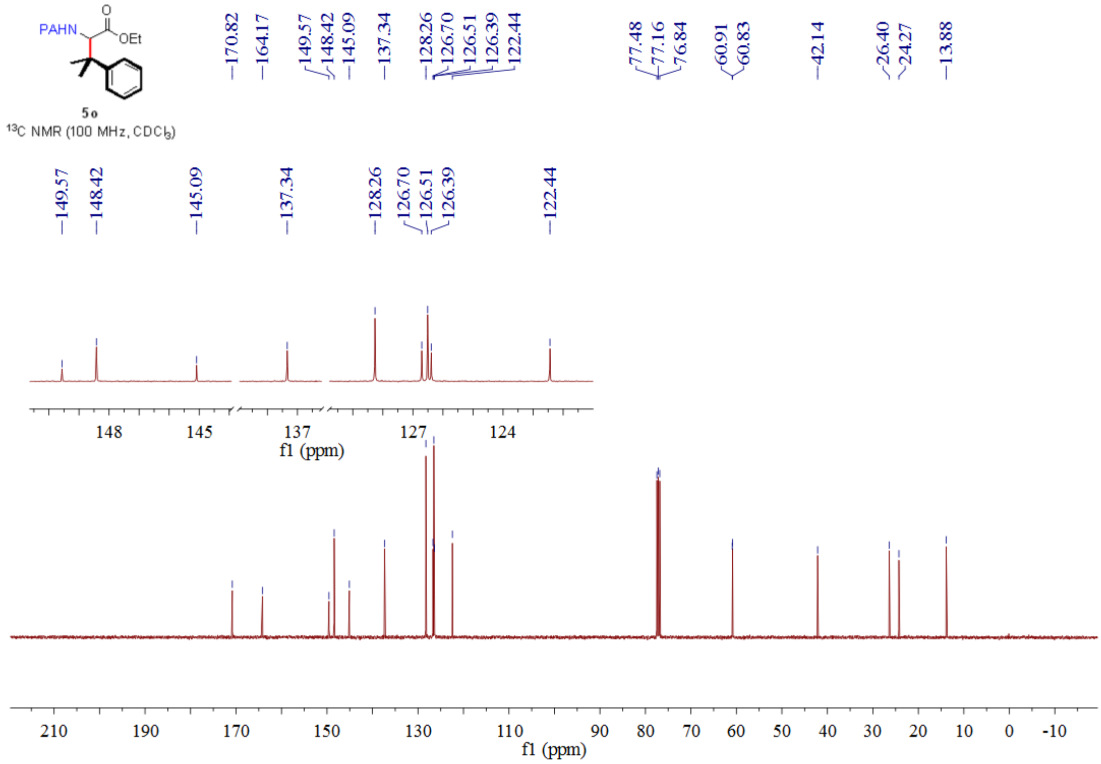

Supplementary Figure 93. ^{1}H NMR and ^{13}C NMR spectra for compound 5o

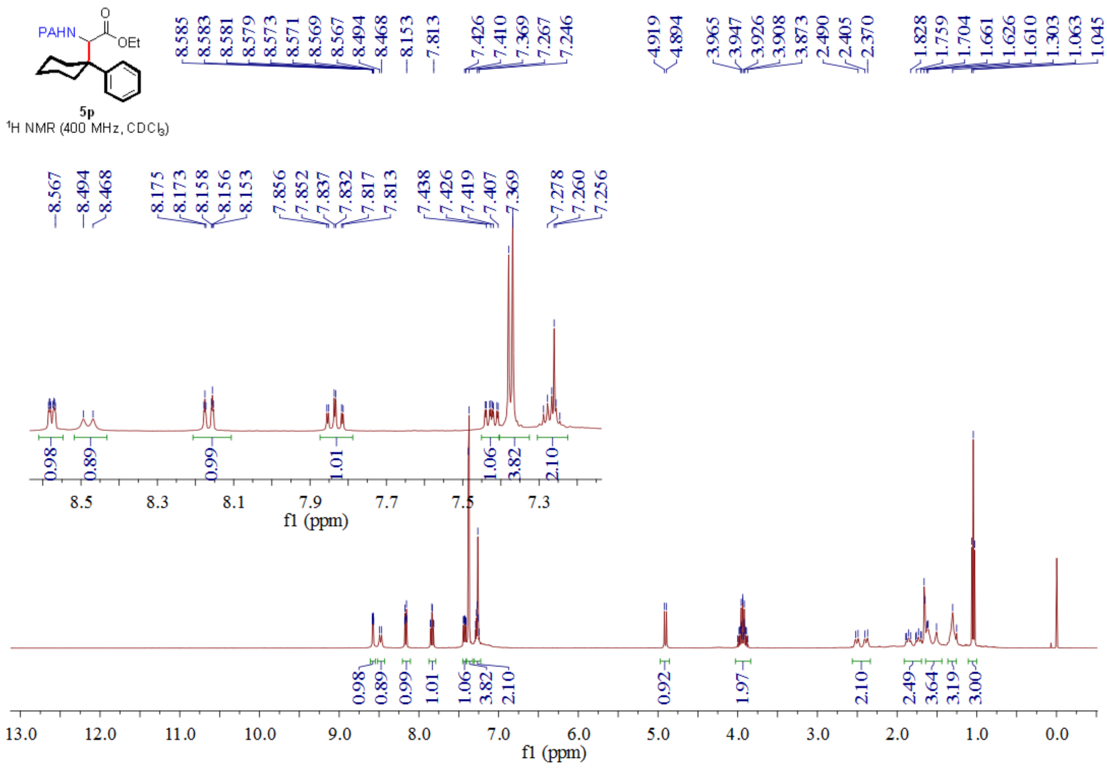

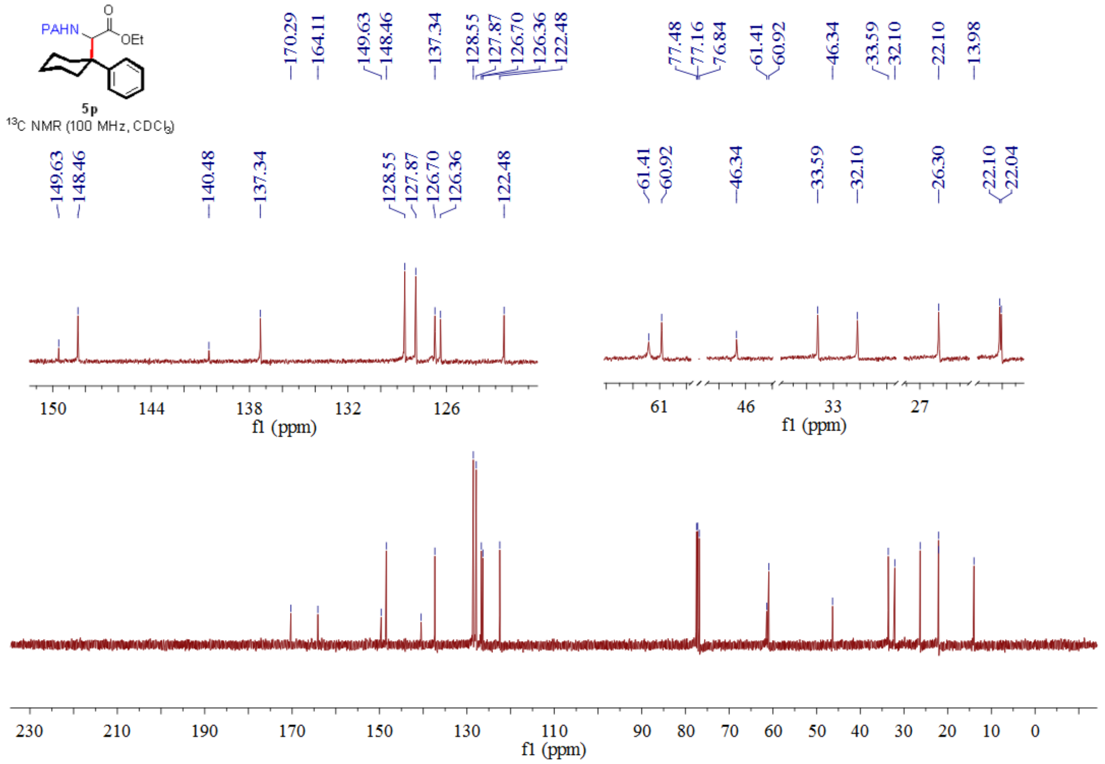

Supplementary Figure 94 ^{1}H NMR and ^{13}C NMR spectra for compound 5p

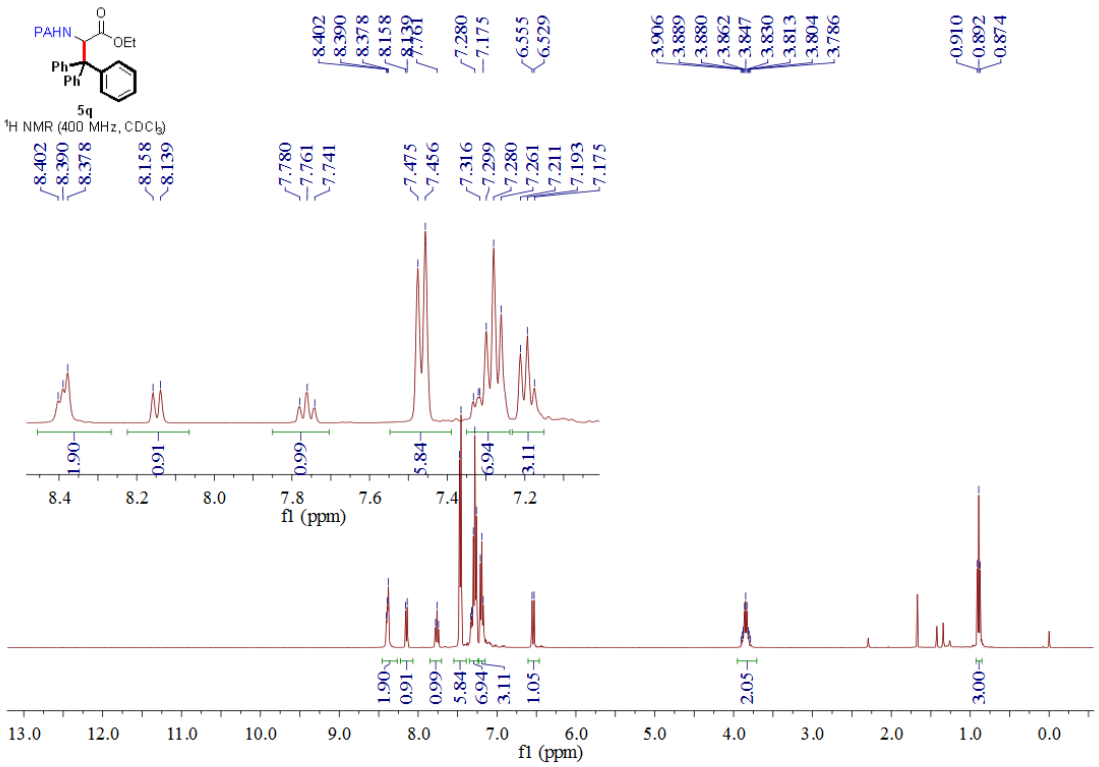

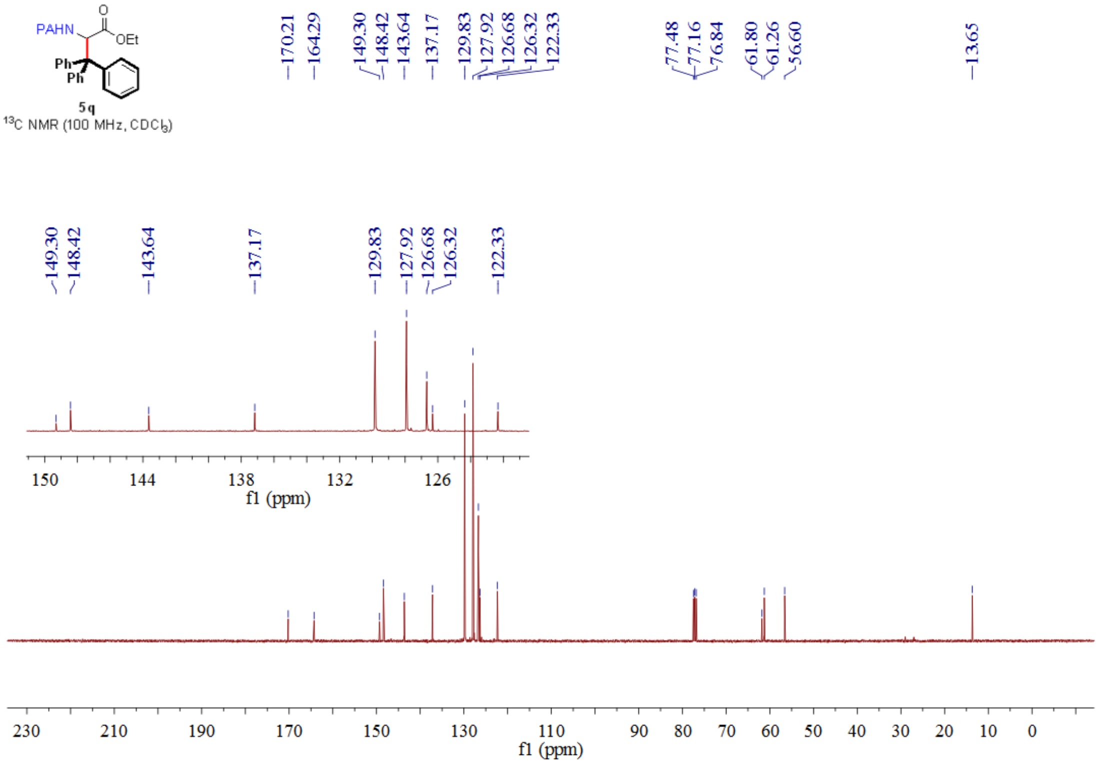

Supplementary Figure 95. ^{1}H NMR and ^{13}C NMR spectra for compound 5q

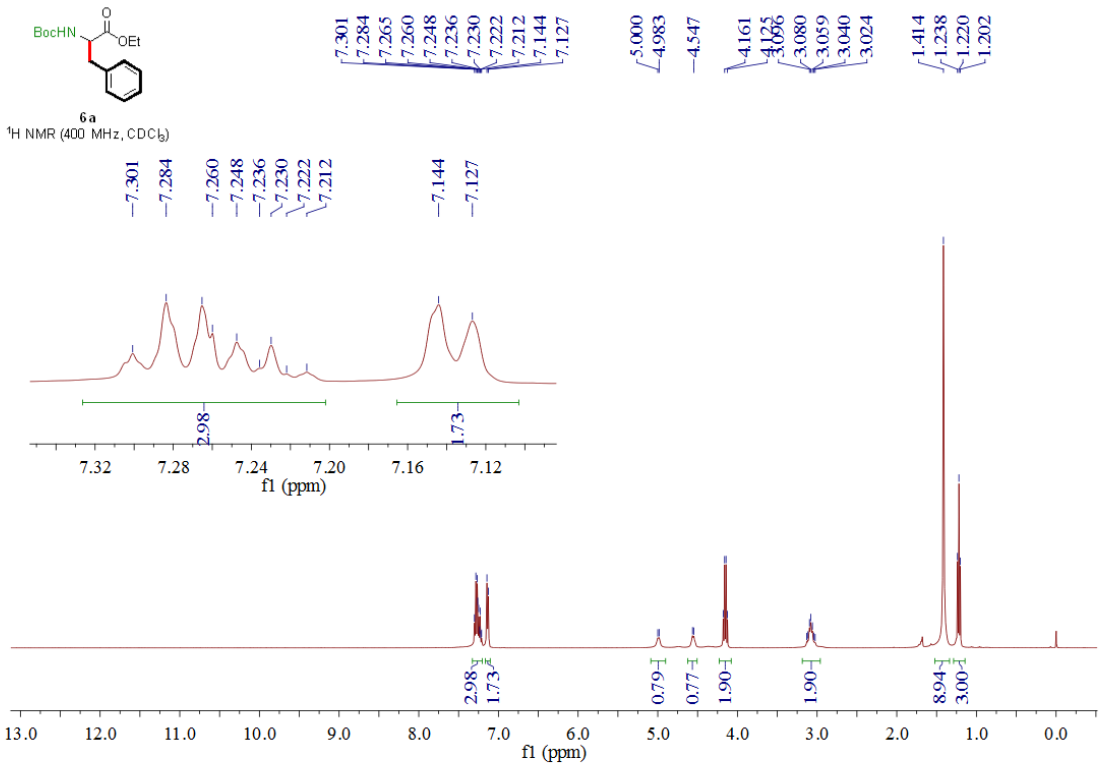

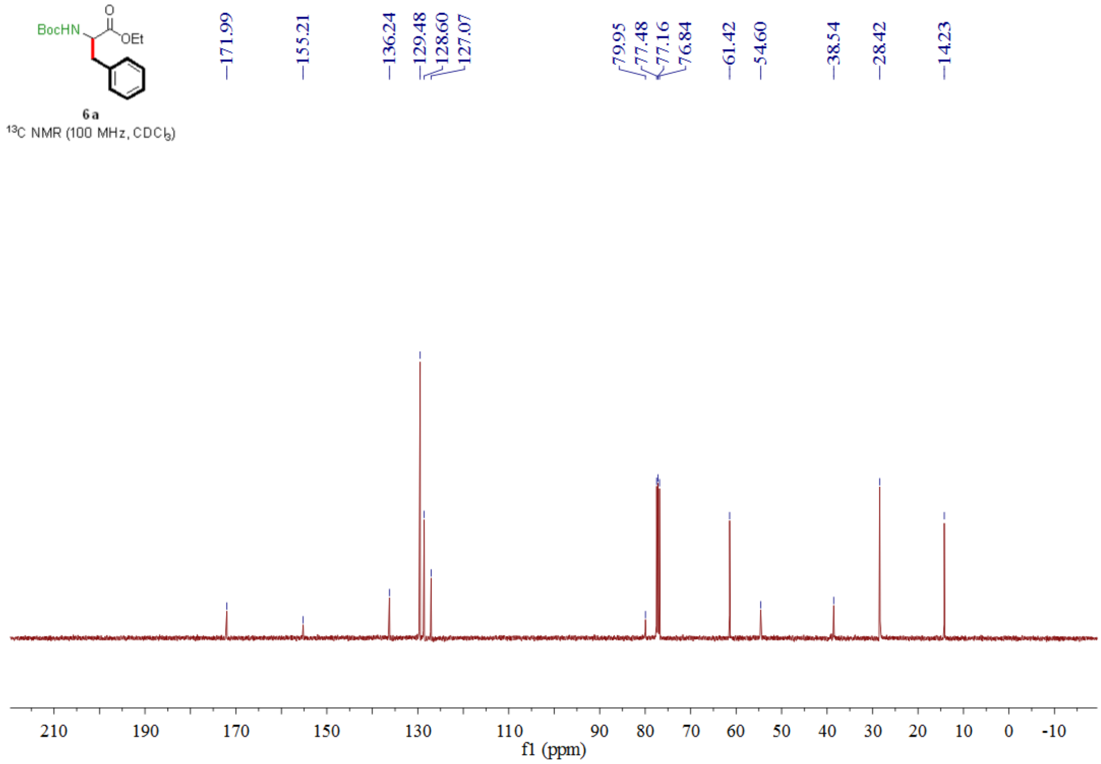

Supplementary Figure 96. ^{1}H NMR and ^{13}C NMR spectra for compound 6a

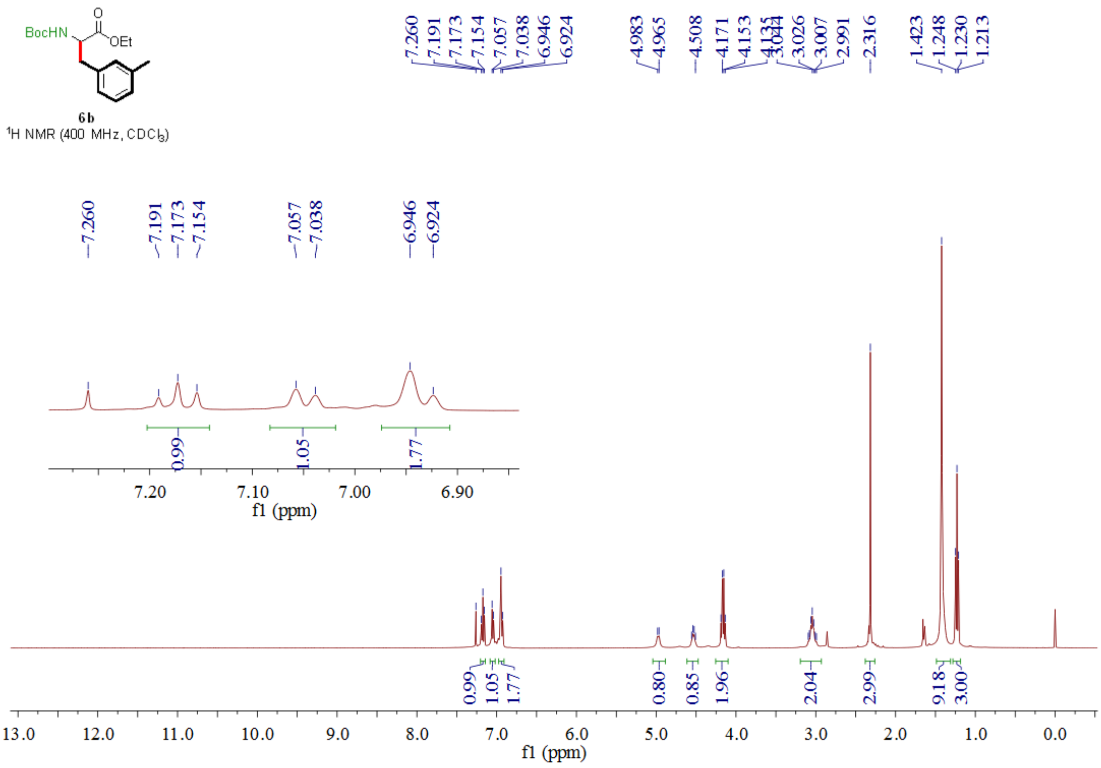

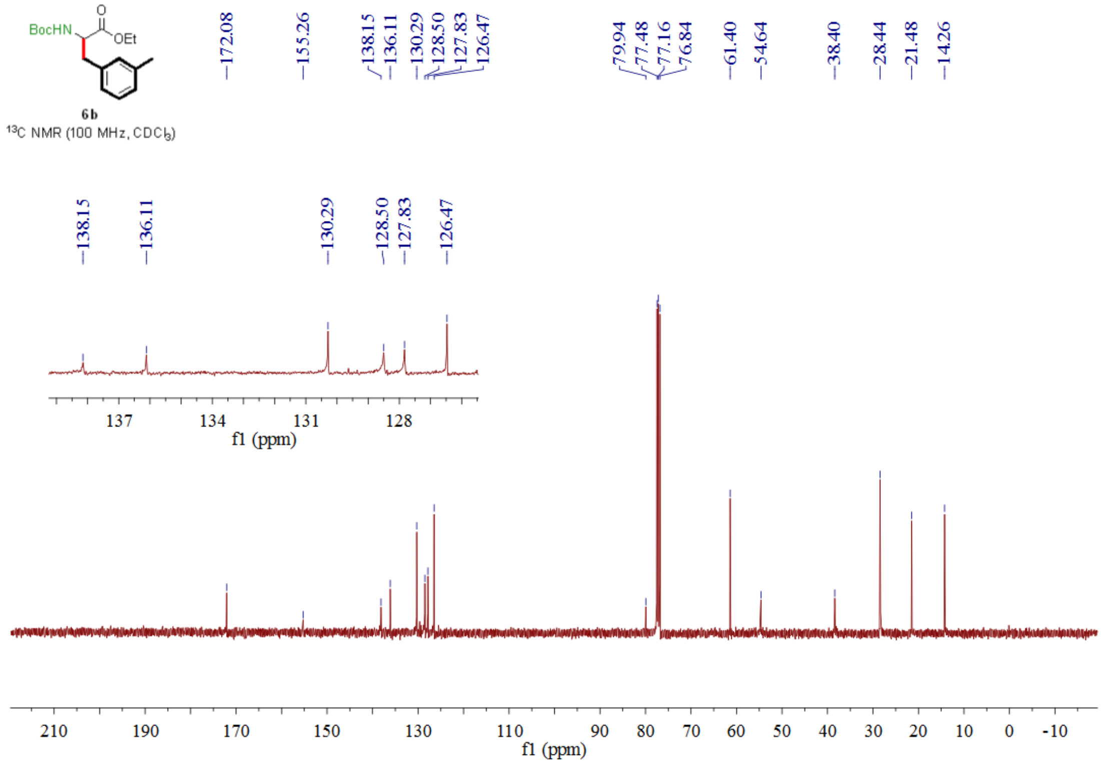

Supplementary Figure 97 ^{1}H NMR and ^{13}C NMR spectra for compound 6b

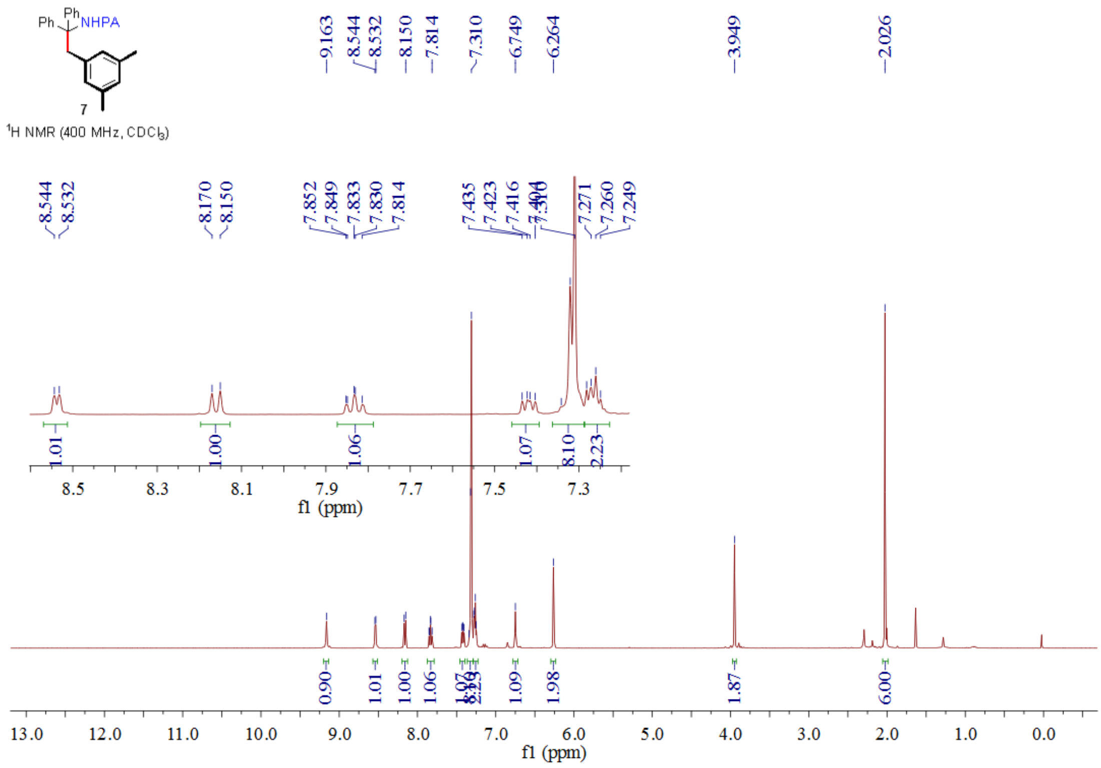

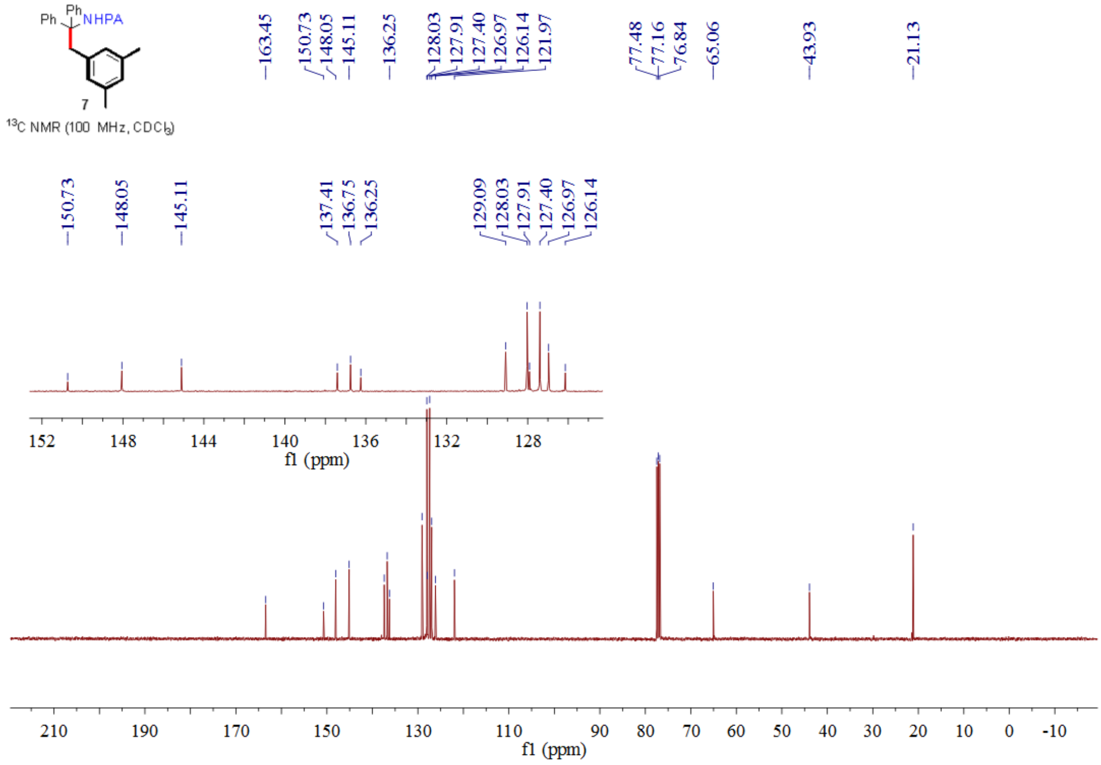

Supplementary Figure 98. ^{1}H NMR and ^{13}C NMR spectra for compound 7

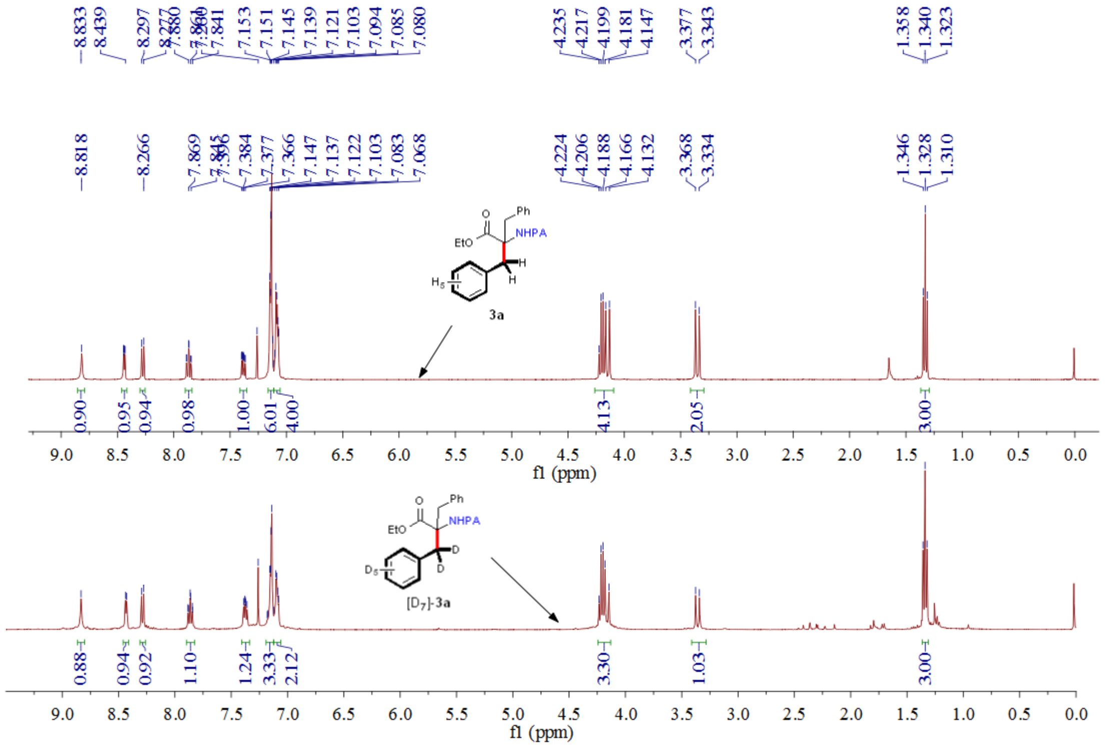

Supplementary Figure 99. ^{1}H NMR spectra for compound 3a and [D$_7$]-3a

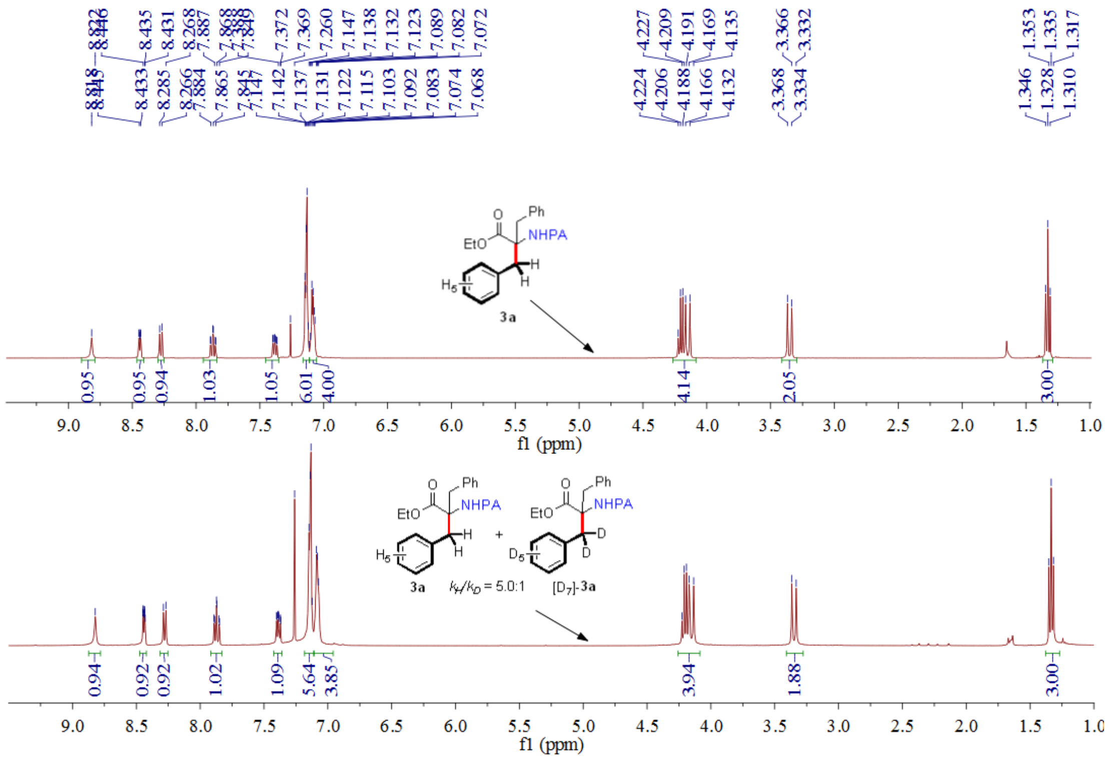

Supplementary Figure 100. ^{1}H NMR spectra for mixture of 3a and [D$_7$]-3a

Supplementary Table 1 | Optimization of the oxidative cross-coupling of ethyl 3-phenyl-2-(picolinamido) propanoate 1a with toluene 2a.

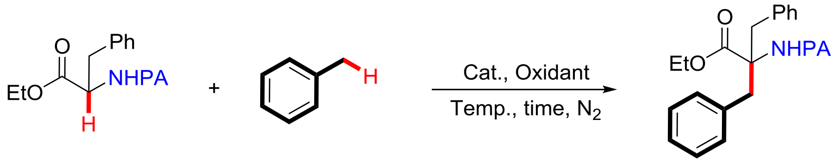

1a **2a** **3a**

Entry	Catalyst (equiv)	Oxidant (equiv)	Temp (°C)	time (h)	Yield (%)[*]
1	$FeCl_3.6H_2O$ (0.2)	DTBP (2.0)	120	12	trace
2	$Cu(OAc)_2$(0.2)	DTBP (2.0)	120	12	trace
3	$Cu(acac)_2$ (0.2)	DTBP (2.0)	120	12	trace
4	$Ru(PPh_3)_2Cl_2$ (0.2)	DTBP (2.0)	120	12	trace
5	$Ni(OAc)_2$ (0.2)	DTBP (2.0)	120	12	trace
6	$Ni(PPh_3)_2Cl_2$ (0.2)	DTBP (2.0)	120	12	17
7	$Ni(acac)_2$ (0.2)	DTBP (2.0)	120	12	31
8	-	DTBP (2.0)	120	12	trace
9	$Ni(acac)_2$ (0.2)	DTBP (2.0)	130	12	36
10	$Ni(acac)_2$ (0.2)	DTBP (3.0)	130	12	60
11	$Ni(acac)_2$ (0.2)	DTBP (4.0)	130	12	77
12	$Ni(acac)_2$ (0.2)	DTBP (4.0)	140	12	74
**13**	**$Ni(acac)_2$ (0.2)**	**DTBP (4.0)**	**140**	**18**	**83**
14	$Ni(acac)_2$ (0.1)	DTBP (4.0)	140	18	76
15	$Ni(acac)_2$ (0.2)	TBHP (4.0)	140	18	N. D.
16	$Ni(acac)_2$ (0.2)	TBPB (4.0)	140	18	N. D.
17	$Ni(acac)_2$ (0.2)	DCP (4.0)	140	18	31
18	$Ni(acac)_2$ (0.2)	$K_2S_2O_8$ (4.0)	140	18	N. D.
19	$Ni(acac)_2$ (0.2)	$(NH_4)_2S_2O_8$ (4.0)	140	18	N. D.
20	$Ni(acac)_2$ (0.2)	$PhI(OAc)_2$ (4.0)	140	18	N. D.
21	$Ni(acac)_2$ (0.2)	DDQ (4.0)	140	18	N. D.
22	$Ni(acac)_2$ (0.2)	BPO (4.0)	140	18	N. D.
23[†]	$Ni(acac)_2$ (0.2)	DTBP (4.0)	140	18	48

24	$Ni(acac)_2$ (0.2)	--	140	18	N. D.

Reaction conditions: **1a** (0.25 mmol), **2a** (1.0 mL), catalyst, and oxidant at indicated temperature under N_2. *Isolated yield after chromatographic purification. †The reaction was carried under air. N. D. = Not detected, DTBP = di-*tert*-butyl peroxide, TBHP = *tert*-butyl hydroperoxide, TBPB = *tert*-butyl peroxybenzoate, DCP = dicumyl peroxide, DDQ = 2,3-dichloro-5,6-dicyano-1,4-benzoquinone, BPO = benzoyl peroxide.

Supplementary Methods

I. General remarks

NMR spectra were obtained on a Bruker AMX-400. The ^{1}H NMR (400 MHz) chemical shifts were measured relative to $CDCl_3$ or TMS as the internal reference ($CDCl_3$: δ = 7.26 ppm; TMS: δ = 0.00 ppm). The ^{13}C NMR (100 MHz) chemical shifts were given using $CDCl_3$ as the internal standard ($CDCl_3$: δ = 77.16 ppm). The following abbreviations (or combinations thereof) were used to explain multiplicities: s = singlet, d = doublet, t = triplet, q = quartet, m = multiplet, br = broad. High-resolution mass spectra (HRMS) were obtained with a Waters-Q-TOF-Premier (ESI). Melting points were determined with XRC-1 and are uncorrected.

Unless otherwise noted, all reagents were obtained from commercial suppliers and used without further purification. Compounds **1a**-**1v** were prepared according to the literature procedure[1-2]. All solvents were purified and dried according to standard methods prior to use.

II. General procedure for the oxidative cross-coupling of α-amino acid esters with arylmethanes

Reaction conditions A: An oven-dried Schlenk tube with a magnetic stir bar was charged with amino acid derivative **1** (0.25 mmol), arylmethane **2** (1.0 mL) and $Ni(acac)_2$ (12.8 mg, 0.05 mmol) under N_2 atmosphere. The reaction solution was stirred at room temperature for several minutes and DTBP (182.6 μL, 1.0 mmol) was then added. The tube was sealed with a teflon-coated cap and the mixture was stirred at 140 °C for 18 h. After being cooled to ambient temperature, the solution was diluted with 20 mL of CH_2Cl_2, filtered through a celite pad, and washed with 10-20

mL of CH_2Cl_2. The combined organic phases were concentrated and the residue was purified by column chromatography on silica gel to provide the desired product.

Reaction conditions B: An oven-dried Schlenk tube with a magnetic stir bar was charged with amino acid derivative **1** (0.25 mmol), arylmethane **2** (2.5 mmol), $Ni(acac)_2$ (12.8 mg, 0.05 mmol) and benzene (0.5 mL) under an N_2 atmosphere. The reaction solution was stirred at room temperature for several minutes and DTBP (182.6 μL, 1.0 mmol) was then added. The tube was sealed with a teflon-coated cap and the mixture was stirred at 140 °C for 18 h. After being cooled to ambient temperature, the solution was diluted with 20 mL of CH_2Cl_2, filtered through a celite pad, and washed with 10-20 mL of CH_2Cl_2. The combined organic phases were concentrated and the residue was purified by column chromatography on silica gel to provide the desired product.

III. Procedure for the synthesis of 3a and 5c on gram scale

a) Procedure for the synthesis of 3a on gram scale

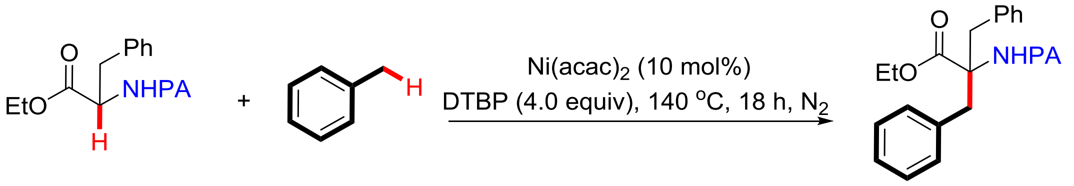

1a **2a** **3a**

86% on 1.0 mmol scale
78% on 5.0 mmol scale

An oven-dried Schlenk tube with a magnetic stir bar was charged with ethyl 3-phenyl-2-(picolinamido)propanoate **1a** (1.4917 g, 5.0 mmol), toluene **2a** (25.0 mL) and $Ni(acac)_2$ (128.0 mg, 0.5 mmol) under an N_2 atmosphere. The reaction solution was stirred at room temperature for several minutes and DTBP (3.65 mL, 20.0 mmol) was then added. The tube was sealed with a teflon-coated cap and the mixture was stirred at 140 °C for 18 h. After being cooled to ambient temperature, the solution was diluted with 40 mL of CH_2Cl_2, filtered through a celite pad, and washed with 20-30 mL of CH_2Cl_2. The combined organic phases were concentrated and the residue was purified by column chromatography on silica gel to provide the product **3a** (1.5224 g, 78%).

b) Procedure for the synthesis of 5c on gram scale

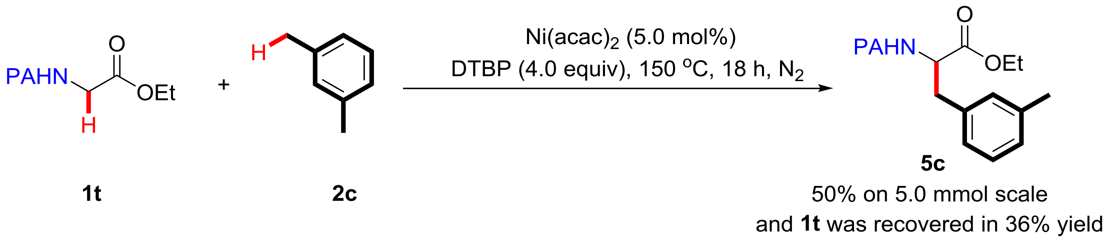

An oven-dried Schlenk tube with a magnetic stir bar was charged with ethyl 2-(picolinamido)acetate **1t** (1.041 g, 5.0 mmol), *m*-xylene **2c** (25.0 mL) and $Ni(acac)_2$ (64.0 mg, 0.25 mmol) under an N_2 atmosphere. The reaction solution was stirred at room temperature for several minutes and DTBP (3.65 mL, 20.0 mmol) was then added. The tube was sealed with a teflon-coated cap and the mixture was stirred at 150 °C for 18 h. After being cooled to ambient temperature, the solution was diluted with 40 mL of CH_2Cl_2, filtered through a celite pad, and washed with 20-30 mL of CH_2Cl_2. The combined organic phases were concentrated and the residue was purified by column chromatography on silica gel to provide the product **5c** (786.7 mg, 50%) and **1t** was recovered in 36% yield.

IV. Removal of picolinic acid auxiliary.

a) Procedure for the synthesis of 6a

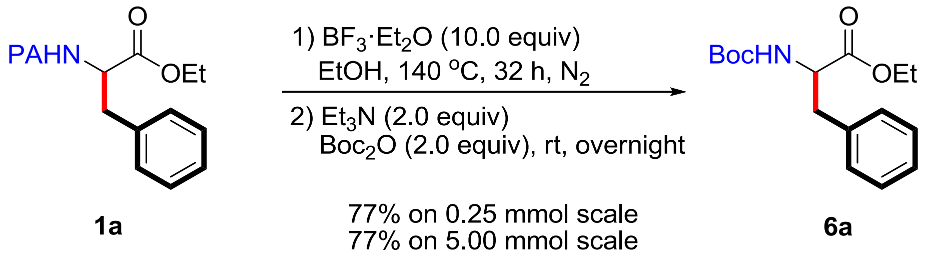

An oven-dried Schlenk tube with a magnetic stir bar was charged with product **1a** (1.49 g, 5.0 mmol, 1.0 equiv) and ethanol (30.0 mL) under an N_2 atmosphere. The reaction solution was stirred at room temperature and $BF_3{\cdot}Et_2O$ (6.3 mL, 50.0 mmol, 10.0 equiv) was added dropwise to the stirred solution. The tube was sealed with a teflon-coated cap and the mixture was stirred at 140 °C for 32 h. After being cooled to ambient temperature, the mixture was quenched by slow addition of saturated Na_2CO_3

solution. The aqueous phase was extracted with ethyl acetate (3 × 30 mL). The combined organic layers were next washed with brine, dried over anhydrous Na_2SO_4, filtered and concentrated *in vacuo*. The residue was dissolved in DCM (30.0 mL). Et_3N (1.4 mL, 10.0 mmol, 2.0 equiv) and Boc_2O (2.18 g, 10.0 mmol, 2.0 equiv) were then added. The solution was stirred overnight at room temperature. After concentration, the mixture was purified by column chromatography using petroleum ether/EtOAc (15/1) as the eluent, and the product **6a** was obtained as colorless oil (1.13 g, 77%).

b) Procedure for the synthesis of 6b

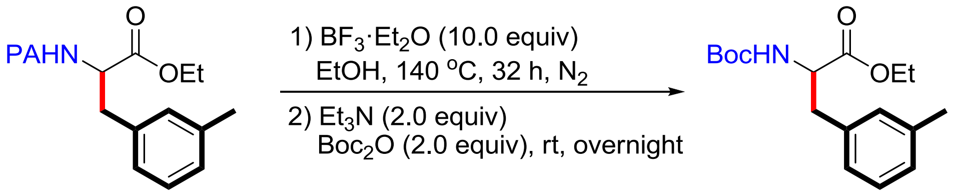

5c **6b**

73% on 2.5 mmol scale

An oven-dried Schlenk tube with a magnetic stir bar was charged with product **5c** (786.7 mg, 2.5 mmol, 1.0 equiv) and ethanol (20.0 mL) under an N_2 atmosphere. The reaction solution was stirred at room temperature and $BF_3{\cdot}Et_2O$ (3.2 mL, 25.0 mmol, 10.0 equiv) was added dropwise to the stirred solution. The tube was sealed with a teflon-coated cap and the mixture was stirred at 140 °C for 32 h. After being cooled to ambient temperature, the mixture was quenched by slow addition of saturated Na_2CO_3 solution. The aqueous phase was extracted with ethyl acetate (3 × 30 mL). The combined organic layers were next washed with brine, dried over anhydrous Na_2SO_4, filtered and concentrated *in vacuo*. The residue was dissolved in DCM (30.0 mL). Et_3N (0.7 mL, 5.0 mmol, 2.0 equiv) and Boc_2O (1.09 g, 5.0 mmol, 2.0 equiv) were then added. The solution was stirred overnight at room temperature. After concentration, the mixture was purified by column chromatography using petroleum ether/EtOAc (15/1) as the eluent, and the product **6b** was obtained as colorless oil (563.9 mg, 73%).

V. Investigation of the reaction mechanism

a) The effect of radical scavengers on the reaction of α-amino acid ester with arylmethane

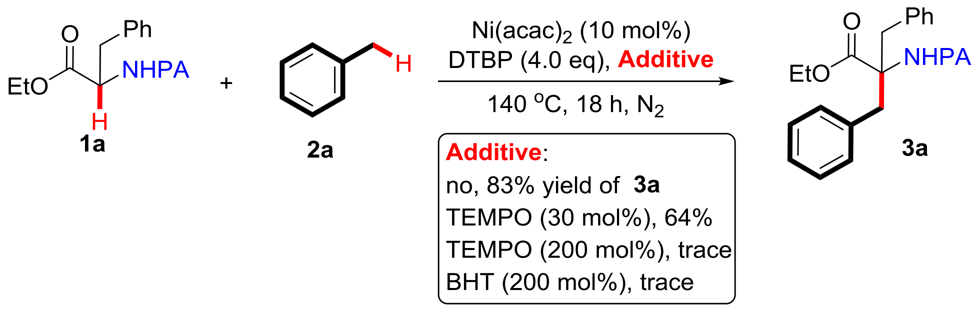

TEMPO = 2,2,6,6-Tetramethyl-1-piperidinyloxy, BHT = Butylated hydroxytoluene

b) Intermolecular kinetic isotope effect (KIE) experiment

An oven-dried Schlenk tube with a magnetic stir bar was charged with ethyl 3-phenyl-2-(picolinamido)propanoate **1a** (74.6 mg, 0.25 mmol), **2a** (1.0 mL), [D$_8$]-**2a** (1.0 mL), and Ni(acac)$_2$ (12.8 mg, 0.05 mmol) under an N_2 atmosphere. The reaction solution was stirred at room temperature for several minutes and DTBP (182.6 μL, 1.0 mmol) was then added. The tube was sealed with a teflon-coated cap and the mixture was stirred at 140 °C for 18 h. After being cooled to ambient temperature, the solution was diluted with 20 mL of CH_2Cl_2, filtered through a celite pad, and washed with 10-20 mL of CH_2Cl_2. The combined organic phases were concentrated and the residue was purified by column chromatography on silica gel (ethyl acetate/petroleum ether = 1/4, v/v) to provide the desired product. The product was analysized by ^{1}H NMR (see Supplementary Figures 99 and 100).

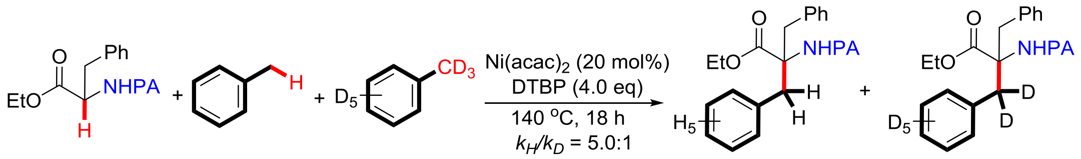

1a **2a** [D$_8$]-**2a** **3a** [D$_7$]-**3a**

c) The reaction of N-benzhydrylpicolinamide 1x and N-(diphenylmethylene)picolinamide 1w with mesitylene 2e

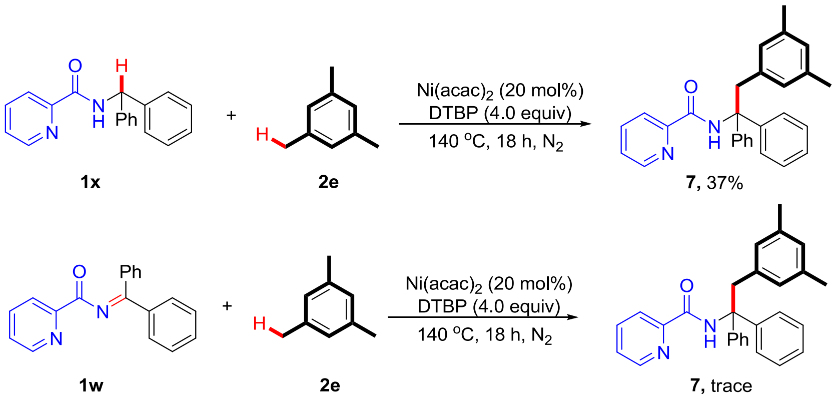

An oven-dried Schlenk tube with a magnetic stir bar was charged with N-benzhydrylpicolinamide **1x** (72.1 mg, 0.25 mmol) or N-(diphenylmethylene)picolinamide **1w** (71.6 mg, 0.25 mmol), **2e** (1.0 mL), and Ni(acac)$_2$ (12.8 mg, 0.05 mmol) under an N_2 atmosphere. The reaction solution was stirred at room temperature for several minutes and DTBP (182.6 µL, 1.0 mmol) was then added. The tube was sealed with a teflon-coated cap and the mixture was stirred at 140 ^{o}C for 18 h. After being cooled to ambient temperature, the solution was diluted with 20 mL of CH_2Cl_2, filtered through a celite pad, and washed with 10-20 mL of CH_2Cl_2. The combined organic phases were concentrated and the residue was purified by column chromatography on silica gel (ethyl acetate/petroleum ether = 1/6, v/v) to provide the desired product **7**.

VI. Experimental data for the described substances

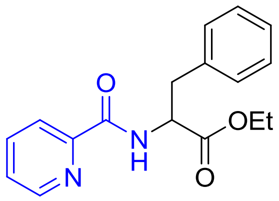

Ethyl 3-phenyl-2-(picolinamido)propanoate (1a)[1]

^{1}H NMR (400 MHz, $CDCl_3$): δ = 1.14 (t, J = 7.2 Hz, 3H), 3.16 (dd, J = 6.4 Hz, 2.8Hz, 2H), 4.10 (q, J = 7.2 Hz, 2H), 4.94-4.99 (m, 1H), 7.11-7.21 (m, 5H), 7.32-7.35 (m, 1H), 7.75 (t, J = 7.6 Hz, 1H), 8.08 (d, J = 8.0 Hz, 1H), 8.44-8.47 (m, 2H) ppm. ^{13}C NMR (100 MHz, $CDCl_3$): δ = 14.2, 38.4, 53.5, 61.5, 122.2, 126.4, 127.1, 128.6, 129.4, 136.1, 137.3, 148.4, 149.4, 164.0, 171.4 ppm. HRMS (ESI$^+$): calcd for $C_{17}H_{18}N_2NaO_3$

$[M+Na]^+$ 321.1215, found 321.1215.

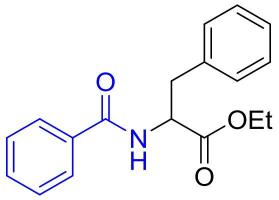

Ethyl 2-benzamido-3-phenylpropanoate (1a-1)[1]

^{1}H NMR (400 MHz, $CDCl_3$): δ = 1.27 (t, J = 7.2 Hz, 3H), 3.21-3.32 (m, 2H), 4.21 (q, J = 7.2 Hz, 2H), 5.05-5.09 (m, 1H), 6.64-6.70 (m, 1H), 7.15-7.17 (m, 2H), 7.23-7.30 (m, 3H), 7.40-7.43 (m, 2H), 7.48-7.52 (m, 1H), 7.72-7.74 (m, 2H) ppm. ^{13}C NMR (100 MHz, $CDCl_3$): δ = 14.2, 38.0, 53.7, 61.7, 127.1, 127.2, 128.6, 128.7, 129.5, 131.8, 134.1, 136.0, 166.9, 171.7 ppm. HRMS (ESI$^+$): calcd for $C_{18}H_{19}NNaO_3$ $[M+Na]^+$ 320.1263, found 320.1260.

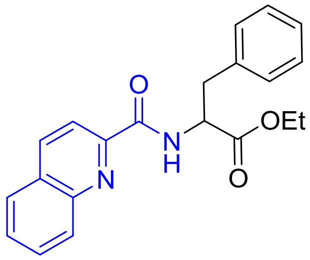

Ethyl 3-phenyl-2-(quinoline-2-carboxamido)propanoate (1a-2)[1]

^{1}H NMR (400 MHz, $CDCl_3$): δ = 1.27 (t, J = 7.2 Hz, 3H), 3.33 d, J = 6.0 Hz, 2H), 4.23 (q, J = 7.2 Hz, 2H), 5.11-5.16 (m, 1H), 7.25-7.34 (m, 5H), 7.60-7.65 (m, 1H), 7.75-7.79 (m, 1H), 7.86-7.87 (m, 1H), 8.12 (d, J = 8.4 Hz, 1H), 8.27-8.32 (m, 2H), 8.76 (d, J = 8.0 Hz, 1H) ppm. ^{13}C NMR (100 MHz, $CDCl_3$): δ = 14.2, 38.6, 53.7, 61.6, 118.8, 127.2, 127.7, 128.1, 128.6, 129.47, 129.52, 130.06, 130.12, 136.2, 137.5, 146.6, 149.3, 164.2, 171.5 ppm. HRMS (ESI$^+$): calcd for $C_{21}H_{20}N_2NaO_3$ $[M+Na]^+$ 371.1372, found 371.1372.

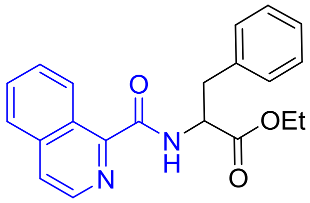

Ethyl 2-(isoquinoline-1-carboxamido)-3-phenylpropanoate (1a-3)[1]

^{1}H NMR (400 MHz, CDCl$_3$): δ = 1.24 (t, J = 7.2 Hz, 3H), 3.24-3.34 (m, 2H), 4.20 (q, J = 7.2 Hz, 2H), 5.06-5.12 (m, 1H), 7.21-7.30 (m, 5H), 7.64-7.72 (m, 2H), 7.79 (d, J = 5.6 Hz, 1H), 7.84 (d, J = 7.6 Hz, 1H), 8.47 (d, J = 5.6 Hz, 1H), 8.70 (d, J = 8.0 Hz, 1H), 9.53 (d, J = 8.4 Hz, 1H) ppm. ^{13}C NMR (100 MHz, CDCl$_3$): δ = 14.3, 38.6, 53.8, 61.6, 124.6, 126.9, 127.1, 127.2, 127.8, 128.6, 128.8, 129.6, 130.5, 136.4, 137.5, 140.6, 147.6, 165.8, 171.7 ppm. HRMS (ESI$^+$): calcd for $C_{21}H_{20}N_2NaO_3$ [M+Na]$^+$ 371.1372, found 371.1370.

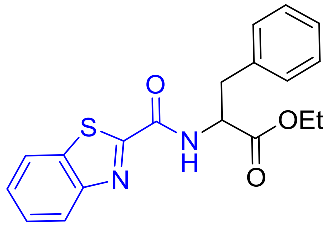

Ethyl 2-(benzo[*d*]thiazole-2-carboxamido)-3-phenylpropanoate (1a-4)[1]

^{1}H NMR (400 MHz, CDCl$_3$): δ = 1.25 (t, J = 7.2 Hz, 3H), 3.23-3.33 (m, 2H), 4.21 (q, J = 7.2 Hz, 2H), 5.04-5.09 (m, 1H), 7.20-7.22 (m, 2H), 7.24-7.32 (m, 3H), 7.46-7.50 (m, 1H), 7.52-7.56 (m, 1H), 7.92 (d, J = 8.0 Hz, 1H), 7.96 (d, J = 8.0 Hz, 1H), 8.08 (d, J = 8.0 Hz, 1H) ppm. ^{13}C NMR (100 MHz, CDCl$_3$): δ = 14.2, 38.4, 53.9, 61.8, 122.4, 124.7, 126.91, 126.92, 127.3, 128.7, 129.5, 135.8, 137.3, 153.0, 159.6, 163.0, 170.9 ppm. HRMS (ESI$^+$): calcd for $C_{19}H_{18}N_2NaO_3S$ [M+Na]$^+$ 377.0936, found 377.0942.

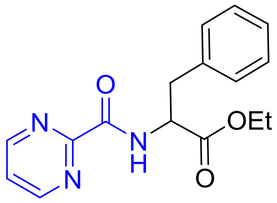

Ethyl 3-phenyl-2-(pyrimidine-2-carboxamido)propanoate (1a-5)[1]

^{1}H NMR (400 MHz, $CDCl_3$): δ = 1.24 (t, J = 7.2 Hz, 3H), 3.22-3.31 (m, 2H), 4.15-4.21 (m, 2H), 5.10-5.15 (m, 1H), 7.16-7.18 (m, 2H), 7.20-7.28 (m, 3H), 7.43 (t, J = 4.8 Hz, 1H), 8.45 (d, J = 8.0 Hz, 1H), 8.86 (d, J = 4.8 Hz, 2H) ppm. ^{13}C NMR (100 MHz, $CDCl_3$): δ = 14.2, 38.3, 53.7, 61.7, 122.8, 127.2, 128.6, 129.5, 136.0, 157.4, 157.6, 161.8, 171.2 ppm. HRMS (ESI$^+$): calcd for $C_{16}H_{18}N_3O_3$ $[M+H]^+$ 300.1348, found 300.1349.

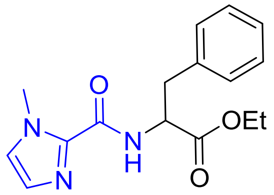

Ethyl 2-(1-methyl-1*H*-imidazole-2-carboxamido)-3-phenylpropanoate (1a-6)[1]

^{1}H NMR (400 MHz, $CDCl_3$): δ = 1.20 (t, J = 7.2 Hz, 3H), 3.12-3.22 (m, 2H), 4.00 (s, 3H), 4.16 (q, J = 7.2 Hz, 2H), 4.89-4.95 (m, 1H), 6.94 (s, 1H), 7.00 (s, 1H), 7.20-7.24 (m, 3H), 7.26-7.30 (m, 2H), 7.76 (d, J = 8.0 Hz, 1H) ppm. ^{13}C NMR (100 MHz, $CDCl_3$): δ = 14.2, 35.6, 38.6, 53.4, 61.6, 125.7, 127.2, 128.1, 128.7, 129.4, 136.2, 138.7, 158.9, 171.3 ppm. HRMS (ESI$^+$): calcd for $C_{16}H_{19}N_3NaO_3$ $[M+Na]^+$ 324.1324, found 324.1319.

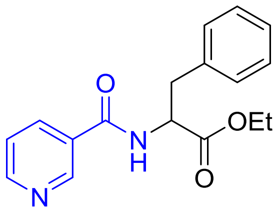

Ethyl 2-(nicotinamido)-3-phenylpropanoate (1a-7)

^{1}H NMR (400 MHz, $CDCl_3$): δ = 1.26 (t, J = 7.2 Hz, 3H), 3.17-3.29 (m, 2H), 4.20 (q, J = 7.2 Hz, 2H), 5.01-5.06 (m, 1H), 6.91 (d, J = 7.6 Hz, 1H), 7.12-7.14 (m, 2H), 7.20-7.28 (m, 3H), 7.31 (dd, J = 8.0 Hz, 4.8 Hz, 1H), 8.01 (dt, J = 8.0 Hz, 2.0 Hz, 1H), 8.66 (dd, J = 4.8 Hz, 1.6 Hz, 1H), 8.88 (d, J = 2.0 Hz, 1H) ppm. ^{13}C NMR (100 MHz, $CDCl_3$): δ = 14.2, 37.8, 53.7, 61.8, 123.5, 127.3, 128.7, 129.4, 129.7, 135.1, 135.8, 148.2, 152.5, 165.2, 171.5 ppm. HRMS (ESI$^+$): calcd for $C_{17}H_{18}N_2NaO_3$ $[M+Na]^+$ 321.1215, found 321.1218.

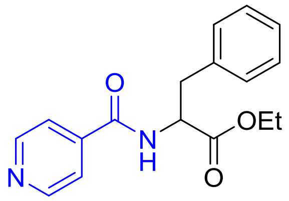

Ethyl 2-(isonicotinamido)-3-phenylpropanoate (1a-8)

^{1}H NMR (400 MHz, $CDCl_3$): δ = 1.29 (t, J = 7.2 Hz, 3H), 3.20-3.32 (m, 2H), 4.23 (q, J = 7.2 Hz, 2H), 5.02-5.06 (m, 1H), 6.73 (d, J = 7.2 Hz, 1H), 7.11-7.13 (m, 2H), 7.24-7.31 (m, 3H), 7.53-7.54 (m, 2H), 8.71-8.72 (m, 2H) ppm. ^{13}C NMR (100 MHz, $CDCl_3$): δ = 14.3, 37.9, 53.7, 62.0, 120.9, 127.5, 128.8, 129.5, 135.7, 141.1, 150.8, 165.0, 171.4 ppm. HRMS (ESI$^+$): calcd for $C_{17}H_{18}N_2NaO_3$ $[M+Na]^+$ 321.1215, found 321.1209.

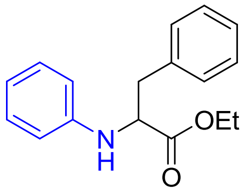

Ethyl phenylphenylalaninate (1a-9)[1]

^{1}H NMR (400 MHz, $CDCl_3$): δ = 1.18 (t, J = 7.2 Hz, 3H), 3.10-3.19 (m, 2H), 4.09-4.17 (m, 2H), 4.36 (t, J = 6.4 Hz, 1H), 6.62 (d, J = 7.6 Hz, 2H), 6.75 (t, J = 7.2 Hz, 1H), 7.16-7.23 (m, 4H), 7.24-7.32 (m, 3H) ppm. ^{13}C NMR (100 MHz, $CDCl_3$): δ = 14.2, 38.8, 57.9, 61.2, 113.7, 118.5, 127.1, 128.6, 129.5, 136.5, 146.5, 173.3 ppm.

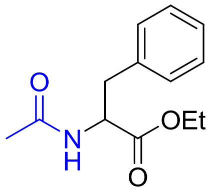

Ethyl acetylphenylalaninate (1a-10)[1]

^{1}H NMR (400 MHz, $CDCl_3$): δ = 1.4 (t, J = 7.2 Hz, 3H), 1.99 (s, 3H), 3.07-3.16 (m, 2H), 4.17 (q, J = 7.2 Hz, 2H), 4.84-4.89 (m, 1H), 5.96 (br s, 1H), 7.09-7.11 (m, 2H), 7.22-7.31 (m, 3H) ppm. ^{13}C NMR (100 MHz, $CDCl_3$): δ = 14.1, 23.0, 37.9, 53.2, 61.4, 127.0, 128.5, 129.3, 136.0, 169.7, 171.8 ppm.

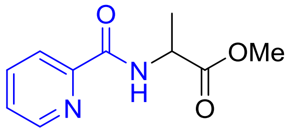

Methyl 2-(picolinamido)propanoate (1b)

^{1}H NMR (400 MHz, $CDCl_3$): δ = 1.53 (d, J = 7.2 Hz, 3H), 3.76 (m, 3H), 4.74-4.82 (m, 1H), 7.40-7.43 (m, 1H), 7.82 (t, J = 7.6 Hz, 1H), 8.15 (d, J = 7.6 Hz, 1H), 8.47 (d, J = 5.6 Hz, 1H), 8.55-8.56 (m, 1H) ppm. ^{13}C NMR (100 MHz, $CDCl_3$): δ = 18.5, 48.2, 52.6, 122.4, 126.5, 137.4, 148.3, 149.5, 164.0, 173.3 ppm. HRMS (ESI$^+$): calcd for $C_{10}H_{12}N_2NaO_3$ [M+Na]$^+$ 231.0746, found 231.0749.

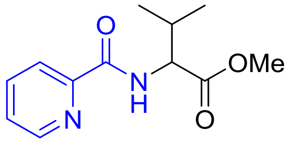

Methyl 3-methyl-2-(picolinamido)butanoate (1c)

^{1}H NMR (400 MHz, $CDCl_3$): δ = 0.98-1.02 (m, 6H), 2.25-2.34 (m, 1H), 3.745-3.752 (m, 3H), 4.70-4.74 (m, 1H), 7.40-7.43 (m, 1H), 7.80-7.85 (m, 1H), 8.15 (d, J = 7.6 Hz, 1H), 8.50 (d, J = 8.4 Hz, 1H), 8.57-8.58 (m, 1H) ppm. ^{13}C NMR (100 MHz, $CDCl_3$): δ = 18.0, 19.3, 31.6, 52.3, 57.4, 122.4, 126.4, 137.4, 148.4, 149.5, 164.3, 172.3 ppm. HRMS (ESI$^+$): calcd for $C_{12}H_{16}N_2NaO_3$ [M+Na]$^+$ 259.1059, found 259.1063.

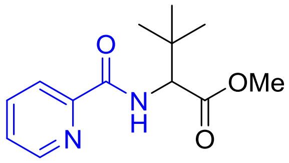

Methyl 3,3-dimethyl-2-(picolinamido)butanoate (1d)

^{1}H NMR (400 MHz, $CDCl_3$): δ = 1.05 (s, 9H), 3.73 (s, 3H), 4.62 (d, J = 9.6 Hz, 1H), 7.42 (ddd, J = 7.6 Hz, 4.8 Hz, 0.8 Hz, 1H), 7.82 (td, J = 7.6 Hz, 1.6 Hz, 1H), 8.15 (d, J = 7.6 Hz, 1H), 8.57 (dq, J = 4.8 Hz, 0.8 Hz, 1H), 8.61 (d, J = 9.6 Hz, 1H) ppm. ^{13}C NMR (100 MHz, $CDCl_3$): δ = 26.7, 35.1, 51.9, 60.4, 122.4, 126.4, 137.4, 148.4, 149.5, 164.1, 171.9 ppm. HRMS (ESI$^+$): calcd for $C_{13}H_{18}N_2NaO_3$ [M+Na]$^+$ 273.1215, found 273.1215.

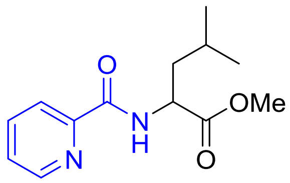

Methyl 4-methyl-2-(picolinamido)pentanoate (1e)

^{1}H NMR (400 MHz, CDCl$_3$): δ = 0.95-0.98 (m, 6H), 1.69-1.79 (m, 3H), 3.75 (s, 3H), 4.80-4.85 (m, 1H), 7.42 (ddd, J = 7.6 Hz, 4.8 Hz, 0.8 Hz, 1H), 7.83 (td, J = 7.6 Hz, 1.6 Hz, 1H), 8.16 (d, J = 8.0 Hz, 1H), 8.35 (d, J = 8.4 Hz, 1H), 8.57 (d, J = 4.8 Hz, 1H) ppm. ^{13}C NMR (100 MHz, CDCl$_3$): δ = 22.0, 23.0, 25.0, 41.8, 50.9, 52.4, 122.5, 126.5, 137.4, 148.3, 149.5, 164.2, 173.3 ppm. HRMS (ESI$^+$): calcd for $C_{13}H_{19}N_2O_3$ [M+H]$^+$ 251.1396, found 251.1397.

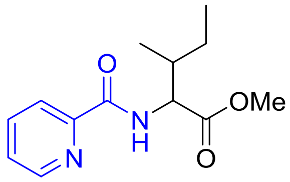

Methyl 3-methyl-2-(picolinamido)pentanoate (1f)

^{1}H NMR (400 MHz, CDCl$_3$): δ = 0.92-0.99 (m, 6H), 1.21-1.33 (m, 1H), 1.49-1.59 (m, 1H), 1.99-2.08 (m, 1H), 3.75 (s, 3H), 4.74-4.78 (m, 1H), 7.40-7.44 (m, 1H), 7.80-7.85 (m, 1H), 8.14-8.17 (m, 1H), 8.51 (d, J = 8.4 Hz, 1H), 8.57-8.58 (m, 1H) ppm. ^{13}C NMR (100 MHz, CDCl$_3$): δ = 11.7, 15.8, 25.3, 38.2, 52.2, 56.8, 122.4, 126.4, 137.4, 148.4, 149.6, 164.2, 172.3 ppm. HRMS (ESI$^+$): calcd for $C_{13}H_{18}N_2NaO_3$ [M+Na]$^+$ 273.1215, found 273.1214.

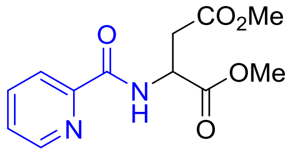

Dimethyl 2-(picolinamido)succinate (1g)[1]

^{1}H NMR (400 MHz, CDCl$_3$): δ = 2.95 (dd, J = 16.8 Hz, 4.8 Hz, 1H), 3.13 (dd, J = 17.2 Hz, 4.8 Hz, 1H), 3.70 (s, 3H), 3.78 (s, 3H), 5.05-5.10 (m, 1H), 7.41-7.44 (m, 1H),

7.83 (t, J = 7.6 Hz, 1H), 8.16 (d, J = 7.6 Hz, 1H), 8.58 (d, J = 4.4 Hz, 1H), 8.86 (d, J = 8.0 Hz, 1H) ppm. ^{13}C NMR (100 MHz, $CDCl_3$): δ = 36.4, 48.6, 52.2, 53.0, 122.4, 126.6, 137.4, 148.5, 149.2, 164.3, 171.2, 171.4 ppm. HRMS (ESI$^+$): calcd for $C_{12}H_{15}N_2O_5$ $[M+H]^+$ 267.0981, found 267.0984.

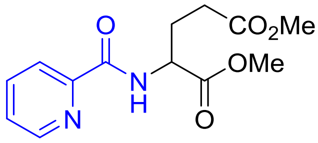

Dimethyl 2-(picolinamido)pentanedioate (1h)

^{1}H NMR (400 MHz, $CDCl_3$): δ = 2.09-2.19 (m, 1H), 2.31-2.39 (m, 1H), 2.42-2.53 (m, 2H), 3.63 (s, 3H), 3.76 (s, 3H), 4.81-4.86 (m, 1H), 7.43 (ddd, J = 7.6 Hz, J = 4.8 Hz, J = 1.2 Hz, 1H), 7.83 (td, J = 7.6 Hz, 1.6 Hz, 1H), 8.15 (d, J = 8.0 Hz, 1H), 8.52 (d, J = 8.4 Hz, 1H), 8.57-8.58 (m, 1H) ppm. ^{13}C NMR (100 MHz, $CDCl_3$): δ = 27.8, 30.3, 51.7, 51.9, 52.7, 122.4, 126.6, 137.4, 148.4, 149.3, 164.4, 172.1, 173.1 ppm. HRMS (ESI$^+$): calcd for $C_{13}H_{16}N_2NaO_5$ $[M+Na]^+$ 303.0957, found 303.0960.

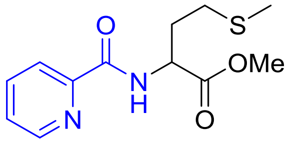

Methyl 4-(methylthio)-2-(picolinamido)butanoate (1i)

^{1}H NMR (400 MHz, $CDCl_3$): δ = 2.06-2.16 (m, 1H), 2.08 (s, 3H), 2.24-2.33 (m, 1H), 2.55-2.59 (m, 2H), 3.77 (s, 3H), 4.88-4.94 (m, 1H), 7.43 (ddd, J =7.6 Hz, 4.8 Hz, 1.2 Hz, 1H), 7.83 (td, J = 7.6 Hz, 1.6 Hz, 1H), 8.15 (d, J = 7.6 Hz, 1H), 8.54 (d, J = 8.4 Hz, 1H), 8.57 (d, J = 4.8 Hz, 1H) ppm. ^{13}C NMR (100 MHz, $CDCl_3$): δ = 15.6, 30.2, 32.1, 51.6, 52.7, 122.4, 126.5, 137.4, 148.4, 149.3, 164.3, 172.2 ppm. HRMS (ESI$^+$): calcd for $C_{12}H_{16}N_2NaO_3S$ $[M+Na]^+$ 291.0779, found 291.0781.

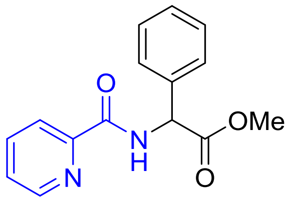

Methyl 2-phenyl-2-(picolinamido)acetate (1j)[1]

^{1}H NMR (400 MHz, $CDCl_3$): δ = 3.77 (s, 3H), 5.77 (d, J = 7.6 Hz, 1H), 7.32-7.40 (m, 3H), 7.42-7.49 (m, 3H), 7.83 (t, J = 7.6 Hz, 1H), 8.16 (d, J = 8.0 Hz, 1H), 8.59 (d, J = 4.4 Hz, 1H), 8.95 (d, J = 6.8 Hz, 1H) ppm. ^{13}C NMR (100 MHz, $CDCl_3$): δ = 52.9, 56.7, 122.4, 126.5, 127.5, 128.7, 129.1, 136.6, 137.4, 148.3, 149.3, 163.8, 171.2 ppm. HRMS (ESI$^+$): calcd for $C_{15}H_{15}N_2O_3$ $[M+H]^+$ 271.1083, found 271.1077.

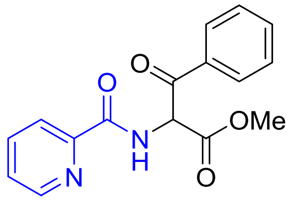

Methyl 3-oxo-3-phenyl-2-(picolinamido)propanoate (1k)

^{1}H NMR (400 MHz, $CDCl_3$): δ = 3.75 (s, 3H), 6.42 (d, J = 8.0 Hz, 1H), 7.43-7.46 (m, 1H), 7.51 (t, J = 7.6 Hz, 2H), 7.63 (t, J = 7.6 Hz, 1H), 7.84 (td, J = 7.6 Hz, 1.6 Hz, 1H), 8.15-8.18 (m, 3H), 8.62 (d, J = 3.6 Hz, 1H), 9.22 (d, J = 7.2 Hz, 1H) ppm. ^{13}C NMR (100 MHz, $CDCl_3$): δ = 53.4, 58.0, 122.6, 126.8, 129.0, 129.6, 134.4, 134.5, 137.4, 148.6, 149.0, 164.2, 167.3, 191.5 ppm. HRMS (ESI$^+$): calcd for $C_{16}H_{14}N_2NaO_4$ $[M+Na]^+$ 321.0851, found 321.0857.

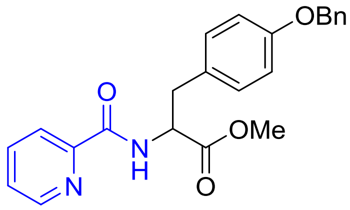

Methyl 3-(4-(benzyloxy)phenyl)-2-(picolinamido)propanoate (1l)[1]

^{1}H NMR (400 MHz, $CDCl_3$): δ = 3.19 (s, 2H), 3.73 (s, 3H), 5.02-5.06 (m, 3H), 6.90 (d, J = 8.0 Hz, 2H), 7.10 (d, J = 8.0 Hz, 2H), 7.32-7.43 (m, 6H), 7.83 (t, J = 7.6 Hz, 1H), 8.16 (d, J = 7.6 Hz, 1H), 8.49 (d, J = 7.6 Hz, 1H), 8.55 (d, J = 3.6 Hz, 1H) ppm. ^{13}C NMR (100 MHz, $CDCl_3$): δ = 37.5, 52.4, 53.7, 70.0, 115.0, 122.3, 126.5, 127.6, 128.0, 128.4, 128.7, 130.4, 137.0, 137.3, 148.4, 149.4, 158.0, 164.0, 171.9 ppm. HRMS (ESI$^+$): calcd for $C_{23}H_{22}N_2NaO_4$ $[M+Na]^+$ 413.1477, found 413.1479.

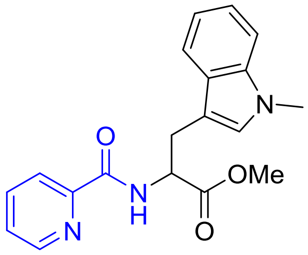

Methyl 3-(1-methyl-1*H*-indol-3-yl)-2-(picolinamido)propanoate (1m)

^{1}H NMR (400 MHz, $CDCl_3$): δ = 3.38-3.49 (m, 2H), 3.69 (s, 3H), 3.73 (s, 3H), 5.10-5.14 (m, 1H), 6.92 (s, 1H), 7.05-7.09 (m, 1H), 7.21 (t, J = 7.6 Hz, 1H), 7.28 (d, J = 8.4 Hz, 1H), 7.41 (ddd, J = 7.6 Hz, 4.8 Hz, 0.8 Hz, 1H), 7.57 (dd, J = 8.0 Hz, 0.8 Hz, 1H), 7.83 (td, J = 7.6 Hz, 1.6 Hz, 1H), 8.18 (dd, J = 8.0 Hz, 0.8 Hz, 1H), 8.51 (d, J = 4.8 Hz, 1H), 8.58 (d, J = 7.6 Hz, 1H) ppm. ^{13}C NMR (100 MHz, $CDCl_3$): δ = 28.1, 32.8, 52.5, 53.2, 108.7, 109.3, 119.0, 119.1, 121.8, 122.4, 126.4, 127.7, 128.1, 137.0, 137.3, 148.3, 149.5, 164.2, 172.3 ppm. HRMS (ESI$^+$): calcd for $C_{19}H_{19}N_3NaO_3$ $[M+Na]^+$ 360.1324, found 360.1321.

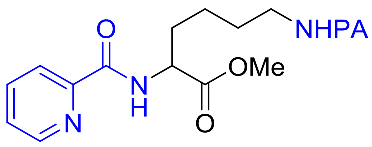

Methyl 2,6-bis(picolinamido)hexanoate (1n)

^{1}H NMR (400 MHz, $CDCl_3$): δ = 1.44-1.59 (m, 2H), 1.63-1.74 (m, 2H), 1.83-1.90 (m, 1H), 1.99-2.07 (m, 1H), 3.43-3.48 (m, 2H), 3.75 (s, 3H), 4.78-4.83 (m, 1H), 7.38-7.44 (m, 2H), 7.79-7.85 (m, 2H), 8.06 (br. s, 1H), 8.14 (d, J = 7.2 Hz, 1H), 8.16 (d, J = 7.2 Hz, 1H), 8.46-8.50 (m, 2H), 8.55-8.57 (m, 1H) ppm. ^{13}C NMR (100 MHz, $CDCl_3$): δ = 23.0, 29.3, 32.4, 39.1, 52.2, 52.5, 122.2, 122.4, 126.2, 126.5, 137.4, 148.1, 148.3, 149.4, 150.0, 164.2, 164.4, 172.7 ppm. HRMS (ESI$^+$): calcd for $C_{19}H_{22}N_4NaO_4$ $[M+Na]^+$ 393.1539, found 393.1542.

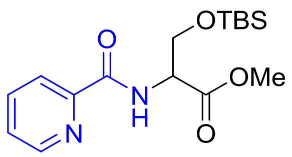

Methyl 3-(*tert*-butyldimethylsilyloxy)-2-(picolinamido)propanoate (1o)

^{1}H NMR (400 MHz, $CDCl_3$): δ = 0.02 (s, 3H), 0.03 (s, 3H), 0.87 (d, J = 2.4 Hz, 9H), 3.77 (d, J = 2.8 Hz, 3H), 3.91-3.95 (m, 1H), 4.18 (dt, J = 10.0 Hz, 2.4 Hz, 1H), 4.83-4.87 (m, 1H), 7.41-7.45 (m, 1H), 7.81-7.86 (m, 1H), 8.15-8.18 (m, 1H), 8.58-8.59 (m, 1H), 8.79 (d, J = 8.0 Hz, 1H) ppm. ^{13}C NMR (100 MHz, $CDCl_3$): δ = -5.5, -5.4, 18.3, 25.8, 52.6, 54.5, 63.8, 122.3, 126.4, 137.3, 148.5, 149.5, 164.3, 170.9 ppm. HRMS (ESI$^+$): calcd for $C_{16}H_{26}N_2NaO_4Si$ $[M+Na]^+$ 361.1560, found 361.1556.

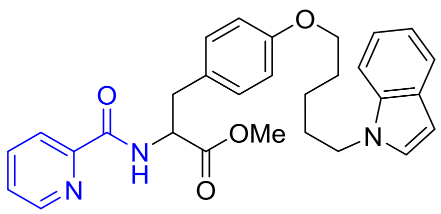

Methyl 3-(4-(5-(1*H*-indol-1-yl)pentyloxy)phenyl)-2-(picolinamido)propanoate (1p)

^{1}H NMR (400 MHz, $CDCl_3$): δ = 1.45-1.53 (m, 2H), 1.75-1.82 (m, 2H), 1.87-1.95 (m, 2H), 3.14-3.24 (m, 2H), 3.74 (s, 3H), 3.89 (t, J = 6.4 Hz, 2H), 4.15 (t, J = 7.2 Hz, 2H), 5.01-5.06 (m, 1H), 6.49 (d, J = 2.8 Hz, 1H), 6.78 (d, J = 8.4 Hz, 2H), 7.07-7.12 (m, 4H), 7.21 (t, J = 7.6 Hz, 1H), 7.35 (d, J = 8.0 Hz, 1H), 7.41-7.44 (m, 1H), 7.64 (d, J = 8.0 Hz, 1H), 7.83 (td, J = 7.6 Hz, 1.2 Hz, 1H), 8.17 (d, J = 8.0 Hz, 1H), 8.48 (d, J = 8.4 Hz, 1H), 8.56 (d, J = 4.4 Hz, 1H) ppm. ^{13}C NMR (100 MHz, $CDCl_3$): δ = 23.7, 29.0, 30.1, 37.5, 46.4, 52.4, 53.7, 67.6, 101.1, 109.4, 114.6, 119.3, 121.1, 121.5, 122.3, 126.5, 127.9, 128.0, 128.7, 130.4, 136.0, 137.4, 148.4, 149.4, 158.1, 164.1, 172.0 ppm. HRMS (ESI$^+$): calcd for $C_{29}H_{31}N_3NaO_4$ $[M+Na]^+$ 508.2212, found 508.2215.

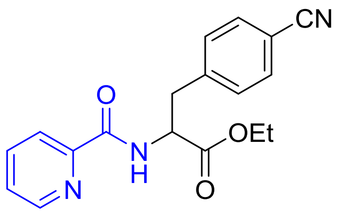

Ethyl 3-(4-cyanophenyl)-2-(picolinamido)propanoate (1q)[1]

^{1}H NMR (400 MHz, $CDCl_3$): δ = 1.23 (t, J = 6.8 Hz, 3H), 3.21-3.36 (m, 2H), 4.18 (q, J = 6.8 Hz, 2H), 5.03-5.08 (m, 1H), 7.30 (d, J = 7.6 Hz, 2H), 7.41-7.44 (m, 1H), 7.54 (d, J = 7.6 Hz, 2H), 7.83 (t, J = 7.6 Hz, 1H), 8.12 (d, J = 8.0 Hz, 1H), 8.51 (d, J = 8.4 Hz, 1H), 8.54 (d, J = 4.0 Hz, 1H) ppm. ^{13}C NMR (100 MHz, $CDCl_3$): δ = 14.2, 38.5, 53.1, 61.9, 111.1, 118.8, 122.3, 126.7, 130.3, 132.3, 137.5, 142.0, 148.5, 149.1, 164.1, 170.9 ppm. HRMS (ESI$^+$): calcd for $C_{18}H_{17}N_3NaO_3$ $[M+Na]^+$ 346.1168, found 346.1169.

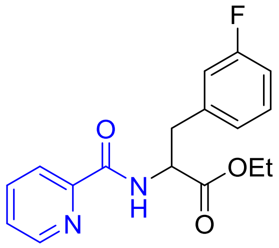

Ethyl 3-(3-fluorophenyl)-2-(picolinamido)propanoate (1r)[1]

^{1}H NMR (400 MHz, $CDCl_3$): δ = 1.23 (t, J = 7.2 Hz, 3H), 3.18-3.28 (m, 2H), 4.18 (q, J = 7.2 Hz, 2H), 5.01-5.06 (m, 1H), 6.89-6.98 (m, 3H), 7.20-7.25 (m, 1H), 7.40-7.43 (m, 1H), 7.81-7.84 (m, 1H), 8.15 (d, J = 8.0 Hz, 1H), 8.51-8.55 (m, 2H) ppm. ^{13}C NMR (100 MHz, $CDCl_3$): δ = 14.2, 37.6, 53.5, 61.6, 115.3, 115.6, 122.3, 126.5, 130.9, 131.0, 131.90, 131.93, 137.4, 148.4, 149.3, 160.8, 163.3, 164.0, 171.3 ppm. HRMS (ESI$^+$): calcd for $C_{17}H_{17}FN_2NaO_3$ $[M+Na]^+$ 339.1121, found 339.1121.

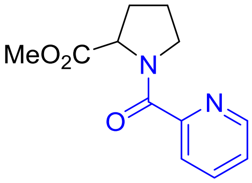

Methyl 1-picolinoylpyrrolidine-2-carboxylate (1s)[2]

The ratio of the two atropisomer is 1.2:1. ^{1}H NMR (400 MHz, $CDCl_3$, a mixture of two atropisomer): δ = 1.91-2.33 (m), 3.62 (s, $CO_2C\underline{H}_3$, major atropisomer), 3.76 (s, $CO_2C\underline{H}_3$, minor atropisomer), 3.78-4.04 (m), 4.67 (dd, *J* = 8.4 Hz, 4.0 Hz, $C\underline{H}CO_2CH_3$, minor atropisomer), 5.12 (dd, *J* = 8.4 Hz, 2.8 Hz, $C\underline{H}CO_2CH_3$, major atropisomer), 7.30 (ddd, *J* = 7.6 Hz, 4.8 Hz, 1.2 Hz, major atropisomer), 7.34 (ddd, *J* = 7.6 Hz, 4.8 Hz, 1.2 Hz, minor atropisomer), 7.75-7.80 (m), 7.89 (d, *J* = 7.6 Hz, minor atropisomer), 8.03 (d, *J* = 8.0 Hz, major atropisomer), 8.44-8.45 (m, major atropisomer), 8.57-8.59 (m, minor atropisomer) ppm. ^{13}C NMR (100 MHz, $CDCl_3$): δ = 22.1, 25.6, 29.0, 32.0, 48.4, 49.9, 52.1, 52.4, 60.2, 61.7, 124.5, 124.7, 125.2, 136.9, 137.0, 147.2, 148.0, 152.8, 153.5, 165.6, 166.4, 172.8, 173.6 ppm.

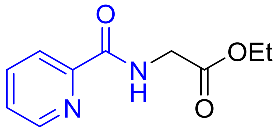

Ethyl 2-(picolinamido)acetate (1t)

^{1}H NMR (400 MHz, $CDCl_3$): δ = 1.30 (t, *J* = 7.2 Hz, 3H), 4.23-4.28 (m, 4H), 7.44 (ddd, *J* = 7.6 Hz, 4.8 Hz, 1.2 Hz, 1H), 7.85 (td, *J* = 8.0 Hz, 1.6 Hz, 1H), 8.18 (d, *J* = 7.6 Hz, 1H), 8.49 (br. s, 1H), 8.58-8.59 (m, 1H) ppm. ^{13}C NMR (100 MHz, $CDCl_3$): δ = 14.2, 41.4, 61.5, 122.3, 126.4, 137.3, 148.3, 149.3, 164.6, 169.8 ppm. HRMS (ESI$^+$): calcd for $C_{10}H_{13}N_2O_3$ $[M+H]^+$ 209.0926, found 209.0925.

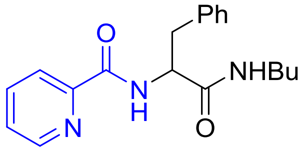

***N*-(1-(Butylamino)-1-oxo-3-phenylpropan-2-yl)picolinamide (1u)**

^{1}H NMR (400 MHz, $CDCl_3$): δ = 0.81 (t, *J* = 7.2 Hz, 3H), 1.11-1.20 (m, 2H), 1.27-1.34 (m, 2H), 3.05-3.25 (m, 4H), 4.73-4.79 (m, 1H), 5.93 (br. s, 1H), 7.17-7.26 (m, 5H), 7.41 (dd, *J* = 7.6 Hz, 4.8 Hz, 1H), 7.81 (td, *J* = 7.6 Hz, 1.2 Hz, 1H), 8.09 (d, *J* = 7.6 Hz, 1H), 8.54 (d, *J* = 4.4 Hz, 1H), 8.58 (d, *J* = 8.4 Hz, 1H) ppm. ^{13}C NMR (100 MHz, $CDCl_3$): δ = 13.8, 20.0, 31.5, 38.6, 39.4, 55.2, 122.3, 126.6, 127.0, 128.7,

129.5, 137.0, 137.4, 148.5, 149.4, 164.5, 170.5 ppm. HRMS (ESI$^+$): calcd for $C_{19}H_{23}N_3NaO_2$ $[M+Na]^+$ 348.1688, found 348.1685.

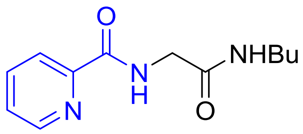

***N*- (2-(Butylamino)-2-oxoethyl)picolinamide (1v)**

^{1}H NMR (400 MHz, $CDCl_3$): δ = 0.87 (t, J = 7.2 Hz, 3H), 1.26-1.35 (m, 2H), 1.43-1.50 (m, 2H), 3.22-3.28 (m, 2H), 4.12 (d, J = 6.0 Hz, 2H), 6.33 (br. s, 1H), 7.41-7.44 (m, 1H), 7.83 (td, J = 7.6 Hz, 1.6 Hz, 1H), 8.14 (d, J = 7.6 Hz, 1H), 8.55-8.56 (m, 1H), 8.60 (br. s, 1H) ppm. ^{13}C NMR (100 MHz, $CDCl_3$): δ = 13.8, 20.2, 31.7, 39.5, 43.8, 122.4, 126.7, 137.5, 148.5, 149.3, 165.2, 168.8 ppm. HRMS (ESI$^+$): calcd for $C_{12}H_{17}N_3NaO_2$ $[M+Na]^+$ 258.1218, found 258.1214.

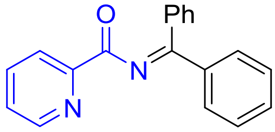

***N*-(diphenylmethylene)picolinamide (1w)**

^{1}H NMR (400 MHz, $CDCl_3$): δ = 7.33-7.39 (m, 5H), 7.41-7.45 (m, 2H), 7.59-7.61 (m, 4H), 7.75-7.79 (m, 1H), 8.06 (d, J = 7.6 Hz, 1H), 8.65 (d, J = 4.8 Hz, 1H) ppm. ^{13}C NMR (100 MHz, $CDCl_3$): δ = 124.3, 126.6, 128.3, 129.6, 131.1, 136.9, 137.0, 149.6, 151.1, 168.5, 178.4 ppm. HRMS (ESI$^+$): calcd for $C_{19}H_{15}N_2O$ $[M+H]^+$ 287.1184, found 287.1184.

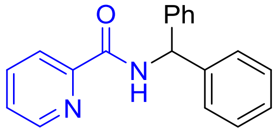

***N*-benzhydrylpicolinamide (1x)**

^{1}H NMR (400 MHz, $CDCl_3$): δ = 6.47 (dd, J = 8.4 Hz, 3.2 Hz, 1H), 7.27-7.31 (m, 2H), 7.33-7.35 (m, 8H), 7.41-7.44 (m, 1H), 7.84 (tt, J = 7.6 Hz, 1.6 Hz, 1H), 8.23 (d, J = 8.0 Hz, 1H), 8.55 (dq, J = 4.8 Hz, 0.8 Hz, 1H), 8.75 (d, J = 5.2 Hz, 1H) ppm. ^{13}C

NMR (100 MHz, $CDCl_3$): δ = 57.1, 122.6, 126.4, 127.58, 127.64, 128.8, 137.5, 141.7, 148.3, 149.9, 163.6 ppm. HRMS (ESI^+): calcd for $C_{19}H_{16}N_2NaO$ $[M+Na]^+$ 311.1160, found 311.1161.

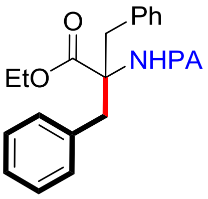

Ethyl 2-benzyl-3-phenyl-2-(picolinamido)propanoate (3a)

Purification via column chromatography on silica gel (ethyl acetate/petroleum ether = 1/4, v/v) afforded **3a** as a pale yellow solid (81.2 mg, 83% yield). M.p.: 118-120 °C. ^1H NMR (400 MHz, $CDCl_3$): δ = 1.33 (t, J = 7.2 Hz, 3H), 3.35 (d, J = 13.6 Hz, 2H), 4.15 (d, J = 13.6 Hz, 2H), 4.20 (q, J = 7.2 Hz, 2H), 7.07-7.10 (m, 4H), 7.12-7.15 (m, 6H), 7.38 (ddd, J = 7.6 Hz, 4.8 Hz, 1.2 Hz, 1H), 7.87 (td, J = 7.6 Hz, 1.6 Hz, 1H), 8.28 (d, J = 7.6 Hz, 1H), 8.43-8.45 (m, 1H), 8.82 (s, 1H) ppm. ^{13}C NMR (100 MHz, $CDCl_3$): δ = 14.3, 41.1, 62.0, 67.3, 121.7, 126.2, 126.9, 128.2, 129.9, 136.4, 137.2, 148.4, 150.2, 164.1, 172.0 ppm. HRMS (ESI^+): calcd for $C_{24}H_{24}N_2NaO_3$ $[M+Na]^+$ 411.1685, found 411.1683.

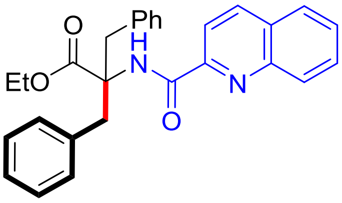

Ethyl 2-benzyl-3-phenyl-2-(quinoline-2-carboxamido)propanoate (3a-2)

Purification via column chromatography on silica gel (ethyl acetate/petroleum ether = 1/4, v/v) afforded **3a-2** as yellow oil (53.5 mg, 49% yield). ^1H NMR (400 MHz, $CDCl_3$): δ = 1.37 (t, J = 7.2 Hz, 3H), 3.41 (d, J = 13.2 Hz, 2H), 4.21 (d, J = 13.6 Hz, 2H), 4.24 (q, J = 7.2 Hz, 2H), 7.14 (m, 10H), 7.56-7.60 (m, 1H), 7.66-7.70 (m, 1H), 7.86-7.88 (m, 1H), 7.97 (d, J = 8.8 Hz, 1H), 8.35 (d, J = 8.4 Hz, 1H), 8.41 (d, J = 8.4 Hz, 1H), 9.07 (s, 1H) ppm. ^{13}C NMR (100 MHz, $CDCl_3$): δ = 14.3, 41.1, 62.0, 67.4, 118.4, 126.9, 127.6, 127.9, 128.3, 129.4, 129.9, 130.3, 136.4, 137.5, 146.6, 149.9,

164.3, 172.1 ppm. HRMS (ESI$^+$): calcd for $C_{28}H_{27}N_2O_3$ $[M+H]^+$ 439.2022, found 439.2018.

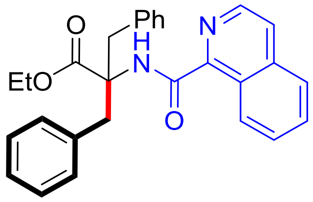

Ethyl 2-benzyl-2-(isoquinoline-1-carboxamido)-3-phenylpropanoate (3a-3)

Purification via column chromatography on silica gel (ethyl acetate/petroleum ether = 1/4, v/v) afforded **3a-3** as yellow oil (49.6 mg, 45% yield). ^{1}H NMR (400 MHz, $CDCl_3$): δ = 1.35 (t, J = 7.2 Hz, 3H), 3.41 (d, J = 13.2 Hz, 2H), 4.20-4.27 (m, 4H), 7.15-7.21 (m, 10H), 7.74-7.78 (m, 3H), 7.86-7.88 (m, 1H), 8.37 (d, J = 5.6 Hz, 1H), 8.91 (s, 1H), 9.68-9.70 (m, 1H) ppm. ^{13}C NMR (100 MHz, $CDCl_3$): δ = 14.3, 41.0, 62.0, 67.5, 124.2, 126.9, 127.0, 127.1, 127.6, 128.3, 128.7, 130.0, 130.4, 136.5, 137.5, 140.7, 148.5, 166.1, 172.1 ppm. HRMS (ESI$^+$): calcd for $C_{28}H_{27}N_2O_3$ $[M+H]^+$ 439.2022, found 439.2019.

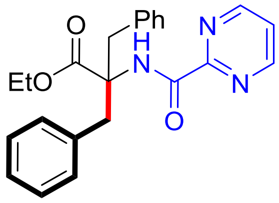

Ethyl 2-benzyl-3-phenyl-2-(pyrimidine-2-carboxamido)propanoate (3a-5)

Purification via column chromatography on silica gel (ethyl acetate/petroleum ether = 1/1, v/v) afforded **3a-5** as yellow oil (16.2 mg, 17% yield). ^{1}H NMR (400 MHz, $CDCl_3$): δ = 1.39 (t, J = 7.2 Hz, 3H), 3.36 (d, J = 13.6 Hz, 2H), 4.20 (d, J = 13.6 Hz, 2H), 4.24 (q, J = 7.2 Hz, 2H), 7.08-7.15 (m, 10H), 7.40 (t, J = 4.8 Hz, 1H), 8.75 (s, 1H), 8.82 (d, J = 4,8 Hz, 2H) ppm. ^{13}C NMR (100 MHz, $CDCl_3$): δ = 14.3, 40.9, 62.3, 67.9, 122.6, 127.0, 128.3, 129.9, 136.3, 157.6, 157.9, 161.8, 172.1 ppm. HRMS (ESI$^+$): calcd for $C_{23}H_{24}N_3O_3$ $[M+H]^+$ 390.1818, found 390.1819.

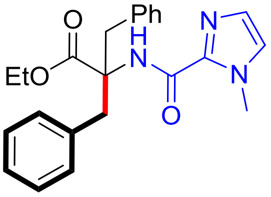

Ethyl 2-benzyl-2-(1-methyl-1*H*-imidazole-2-carboxamido)-3-phenylpropanoate (3a-6)

Purification via column chromatography on silica gel (ethyl acetate/petroleum ether = 1/2, v/v) afforded **3a-5** as yellow oil (36.2 mg, 37% yield). ^{1}H NMR (400 MHz, $CDCl_3$): δ = 1.24 (t, J = 7.2 Hz, 3H), 3.33 (d, J = 13.6 Hz, 2H), 4.00 (d, J = 13.6 Hz, 2H), 4.11-4.17 (m, 5H), 6.93-6.95 (m, 2H), 7.09-7.11 (m, 4H), 7.16-7.19 (m, 6H), 8.02 (s, 1H) ppm. ^{13}C NMR (100 MHz, $CDCl_3$): δ = 14.2, 35.9, 41.3, 62.0, 67.2, 125.6, 127.0, 128.0, 128.3, 129.9, 136.3, 139.3, 159.2, 171.5 ppm. HRMS (ESI$^+$): calcd for $C_{23}H_{26}N_3O_3$ $[M+H]^+$ 392.1974, found 392.1974.

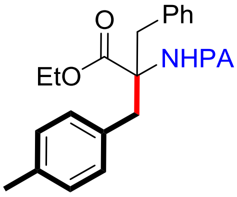

Ethyl 2-benzyl-2-(picolinamido)-3-*p*-tolylpropanoate (3b)

Purification via column chromatography on silica gel (ethyl acetate/petroleum ether = 1/4, v/v) afforded **3b** as pale yellow oil (84.6 mg, 84% yield). ^{1}H NMR (400 MHz, $CDCl_3$): δ = 1.35 (t, J = 7.2 Hz, 3H), 2.23 (s, 3H), 3.34 (t, J = 13.6 Hz, 2H), 4.13 (t, J = 13.2 Hz, 2H), 4.21 (q, J = 7.2 Hz, 2H), 6.94-6.99 (m, 4H), 7.08-7.10 (m, 2H), 7.13-7.16 (m, 3H), 7.36-7.39 (m, 1H), 7.86 (td, J = 7.6 Hz, 1.6 Hz, 1H), 8.28 (d, J = 8.0 Hz, 1H), 8.44-8.45 (m, 1H), 8.83 (s, 1H) ppm. ^{13}C NMR (100 MHz, $CDCl_3$): δ = 14.3, 21.1, 40.7, 41.0, 62.0, 67.4, 121.7, 126.2, 126.9, 128.2, 129.0, 129.7, 129.9, 133.2, 136.4, 136.5, 137.2, 148.4, 150.3, 164.1, 172.1 ppm. HRMS (ESI$^+$): calcd for $C_{25}H_{26}N_2NaO_3$ $[M+Na]^+$ 425.1841, found 425.1838.

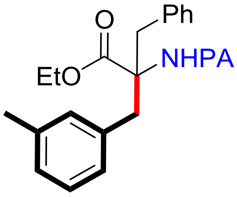

Ethyl 2-benzyl-2-(picolinamido)-3-*m*-tolylpropanoate (3c)

Purification via column chromatography on silica gel (ethyl acetate/petroleum ether = 1/4, v/v) afforded **3c** as pale yellow oil (73.3 mg, 73% yield). ^{1}H NMR (400 MHz, $CDCl_3$): δ = 1.35 (t, J = 7.2 Hz, 3H), 2.15 (s, 3H), 3.33 (d, J = 13.6 Hz, 1H), 3.37 (d, J = 13.6 Hz, 1H), 4.11 (d, J = 13.2 Hz, 1H), 4.17 (d, J = 13.6 Hz, 1H), 4.22 (q, J = 7.2 Hz, 2H), 6.88-6.90 (m, 2H), 6.95 (d, J = 7.6 Hz, 1H), 7.04 (t, J = 7.6 Hz, 1H), 7.10-7.13 (m, 2H), 7.14-7.17 (m, 3H), 7.37 (ddd, J = 7.6 Hz, 4.8 Hz, 1.2 Hz, 1H), 7.86 (td, J = 7.6 Hz, 1.6 Hz, 1H), 8.29 (d, J = 8.0 Hz, 1H), 8.44 (dq, J = 4.8 Hz, 0.8 Hz, 1H), 8.82 (s, 1H) ppm. ^{13}C NMR (100 MHz, $CDCl_3$): δ = 14.3, 21.3, 41.0, 41.1, 62.0, 67.3, 121.6, 126.1, 126.8, 126.9, 127.6, 128.1, 128.2, 129.9, 130.8, 136.2, 136.5, 137.2, 137.6, 148.4, 150.3, 164.1, 172.0 ppm. HRMS (ESI^{+}): calcd for $C_{25}H_{26}N_2NaO_3$ $[M+Na]^{+}$ 425.1841, found 425.1836.

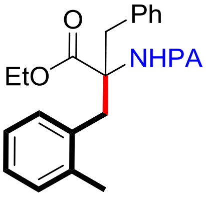

Ethyl 2-benzyl-2-(picolinamido)-3-*o*-tolylpropanoate (3d)

Purification via column chromatography on silica gel (ethyl acetate/petroleum ether = 1/4, v/v) afforded **3d** as pale yellow oil (84.7 mg, 84% yield). ^{1}H NMR (400 MHz, $CDCl_3$): δ = 1.23 (t, J = 7.2 Hz, 3H), 2.31 (s, 3H), 3.44 (d, J = 9.6 Hz, 1H), 3.47 (d, J = 10.4 Hz, 1H), 4.08 (d, J = 14.4 Hz, 1H), 4.08-4.19 (m, 2H), 4.24 (d, J = 13.2 Hz, 1H), 6.98-7.02 (m, 1H), 7.04-7.08 (m, 2H), 7.10-7.16 (m, 6H), 7.38 (ddd, J = 7.6 Hz, 4.8 Hz, 0.8 Hz, 1H), 7.84 (td, J = 7.6 Hz, 1.6 Hz, 1H), 8.25 (d, J = 8.0 Hz, 1H), 8.47 (d, J = 4.4 Hz, 1H), 9.00 (s, 1H) ppm. ^{13}C NMR (100 MHz, $CDCl_3$): δ = 14.0, 19.9, 38.0, 41.1, 62.0, 66.5, 121.7, 125.7, 126.2, 126.7, 126.9, 128.2, 130.0, 130.1, 130.5, 134.9, 136.4, 137.2, 148.4, 150.3, 164.1, 172.3 ppm. HRMS (ESI^{+}): calcd for

$C_{25}H_{26}N_2NaO_3$ $[M+Na]^+$ 425.1841, found 425.1840.

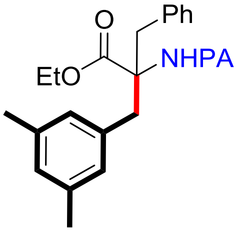

Ethyl 2-benzyl-3-(3,5-dimethylphenyl)-2-(picolinamido)propanoate (3e)

Purification via column chromatography on silica gel (ethyl acetate/petroleum ether = 1/4, v/v) afforded **3e** as pale yellow oil (85.1 mg, 82% yield). ^{1}H NMR (400 MHz, $CDCl_3$): δ = 1.37 (t, J = 7.2 Hz, 3H), 2.11 (s, 6H), 3.28 (d, J = 13.2 Hz, 1H), 3.38 (d, J = 13.6 Hz, 1H), 4.04 (d, J = 13.6 Hz, 1H), 4.17 (d, J = 13.6 Hz, 1H), 4.23 (qd, J = 7.2 Hz, 0.4 Hz, 2H), 6.70 (s, 2H), 6.78 (s, 1H), 7.11-7.18 (m, 5H), 7.37 (ddd, J = 7.6 Hz, 4.8 Hz, 0.4 Hz, 1H), 7.87 (td, J = 7.6 Hz, 1.6 Hz, 1H), 8.30 (d, J = 7.6 Hz, 1H), 8.43-8.45 (m, 1H), 8.80 (s, 1H) ppm. ^{13}C NMR (100 MHz, $CDCl_3$): δ = 14.3, 21.2, 40.9, 41.0, 61.9, 67.4, 121.6, 126.1, 126.8, 127.8, 128.2, 128.5, 129.9, 136.1, 136.5, 137.2, 137.4, 148.4, 150.3, 164.2, 172.0 ppm. HRMS (ESI$^+$): calcd for $C_{26}H_{29}N_2O_3$ $[M+H]^+$ 417.2178, found 417.2177.

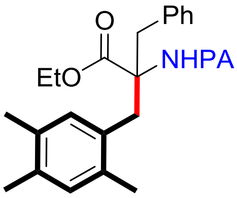

Ethyl 2-benzyl-2-(picolinamido)-3-(2,4,5-trimethylphenyl)propanoate (3f)

Purification via column chromatography on silica gel (ethyl acetate/petroleum ether = 1/4, v/v) afforded **3f** as pale yellow oil (75.7 mg, 70% yield). ^{1}H NMR (400 MHz, $CDCl_3$): δ = 1.29 (t, J = 7.2 Hz, 3H), 2.02 (s, 3H), 2.13 (s, 3H), 2.20 (s, 3H), 3.38 (d, J = 14.4 Hz, 1H), 3.45 (d, J = 13.2 Hz, 1H), 3.93 (d, J = 14.4 Hz, 1H), 4.08-4.23 (m, 2H), 4.23 (d, J = 14.0 Hz, 1H), 6.84 (s, 1H), 6.86 (s, 1H), 7.12-7.18 (m, 5H), 7.38 (ddd, J = 7.6 Hz, 4.8 Hz, 1.2 Hz, 1H), 7.86 (td, J = 7.6 Hz, 1.6 Hz, 1H), 8.26 (d, J = 8.0 Hz, 1H), 8.47-8.48 (m, 1H), 8.93 (s, 1H) ppm. ^{13}C NMR (100 MHz, $CDCl_3$): δ =

14.1, 19.12, 19.14, 19.2, 37.9, 40.8, 61.9, 66.7, 121.7, 126.1, 126.8, 128.2, 130.0, 131.8, 131.9, 132.0, 133.4, 134.4, 134.8, 136.6, 137.2, 148.4, 150.4, 164.2, 172.4 ppm. HRMS (ESI$^+$): calcd for $C_{27}H_{30}N_2NaO_3$ [M+Na]$^+$ 453.2154, found 453.2153.

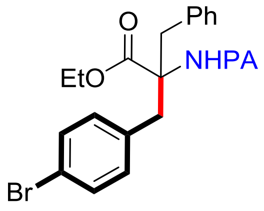

Ethy 2-benzyl-3-(4-bromophenyl)-2-(picolinamido)propanoate (3g)

Purification via column chromatography on silica gel (ethyl acetate/petroleum ether = 1/4, v/v) afforded **3g** as yellow oil (61.5 mg, 52% yield). ^{1}H NMR (400 MHz, $CDCl_3$): δ = 1.33 (t, J = 7.2 Hz, 3H), 3.31 (dd, J = 13.6 Hz, 2.8 Hz, 2H), 4.12 (dd, J = 13.6 Hz, 6.0 Hz, 2H), 4.20 (q, J = 7.2 Hz, 2H), 6.94-6.96 (m, 2H), 7.04-7.08 (m, 2H), 7.12-7.15 (m, 3H), 7.24-7.28 (m, 2H), 7.40 (ddd, J = 7.6 Hz, 4.8 Hz, 0.8 Hz, 1H), 7.87 (td, J = 7.6 Hz, 1.6 Hz, 1H), 8.26 (d, J = 8.0 Hz, 1H), 8.45-8.46 (m, 1H), 8.81 (s, 1H) ppm. ^{13}C NMR (100 MHz, $CDCl_3$): δ = 14.3, 40.4, 41.2, 62.2, 67.2, 121.0, 121.7, 126.4, 127.0, 128.3, 129.9, 131.4, 131.6, 135.5, 136.2, 137.3, 148.6, 150.0, 164.2, 171.8 ppm. HRMS (ESI$^+$): calcd for $C_{24}H_{24}BrN_2O_3$ [M+H]$^+$ 467.0970, found 467.0973.

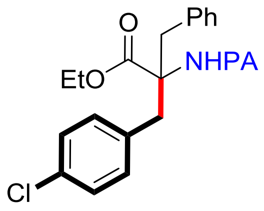

Ethyl 2-benzyl-3-(4-chlorophenyl)-2-(picolinamido)propanoate (3h)

Purification via column chromatography on silica gel (ethyl acetate/petroleum ether = 1/4, v/v) afforded **3h** as yellow oil (73.9 mg, 70% yield). ^{1}H NMR (400 MHz, $CDCl_3$): δ = 1.33 (t, J = 7.2 Hz, 3H), 3.32 (d, J = 13.6 Hz, 2H), 4.13 (dd, J = 13.6 Hz, 10. 0 Hz, 2H), 4.20 (q, J = 7.2 Hz, 2H), 6.98-7.02 (m, 2H), 7.05-7.07 (m, 2H), 7.10-7.15 (m, 5H), 7.40 (dd, J = 7.2 Hz, 4.8 Hz, 1H), 7.87 (t, J = 7.6 Hz, 1H), 8.26 (d, J = 8.0 Hz,

1H), 8.45 (d, J = 4.4 Hz, 1H), 8.81 (s, 1H) ppm. ^{13}C NMR (100 MHz, $CDCl_3$): δ = 14.3, 40.3, 41.1, 62.2, 67.3, 121.7, 126.3, 127.0, 128.3, 128.4, 129.8, 131.2, 132.8, 134.9, 136.2, 137.3, 148.5, 150.0, 164.2, 171.8 ppm. HRMS (ESI$^+$): calcd for $C_{24}H_{24}ClN_2O_3$ $[M+H]^+$ 423.1475, found 423.1473.

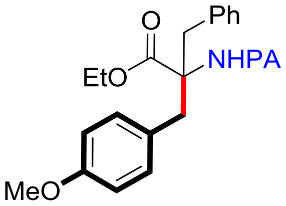

Ethyl 2-benzyl-3-(4-methoxyphenyl)-2-(picolinamido)propanoate (3i)

Purification via column chromatography on silica gel (ethyl acetate/petroleum ether = 1/4, v/v) afforded **3i** as yellow oil (57.1 mg, 55% yield). ^{1}H NMR (400 MHz, $CDCl_3$): δ = 1.34 (t, J = 7.2 Hz, 3H), 3.31 (dd, J = 15.2 Hz, 14.0 Hz, 2H), 3.70 (s, 3H), 4.10 (t, J = 14.4 Hz, 2H), 4.20 (q, J = 7.2 Hz, 2H), 6.66-6.69 (m, 2H), 6.98-7.00 (m, 2H), 7.06-7.09 (m, 2H), 7.12-7.14 (m, 3H), 7.38 (ddd, J = 7.2 Hz, 4.8 Hz, 1.2 Hz, 1H), 7.87 (td, J = 8.0 Hz, 1.6 Hz, 1H), 8.27 (d, J = 8.0 Hz, 1H), 8.44-8.45 (m, 1H), 8.81 (s, 1H) ppm. ^{13}C NMR (100 MHz, $CDCl_3$): δ = 14.3, 40.3, 41.0, 55.2, 62.0, 67.5, 113.7, 121.7, 126.2, 126.9, 128.3, 128.5, 129.9, 130.9, 136.5, 137.3, 148.5, 150.3, 158.5, 164.1, 172.1 ppm. HRMS (ESI$^+$): calcd for $C_{25}H_{26}N_2NaO_4$ $[M+Na]^+$ 441.1790, found 441.1797.

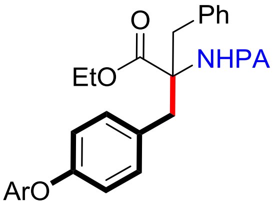

Ethyl 2-benzyl-2-(picolinamido)-3-(4-(*p*-tolyloxy)phenyl)propanoate (3j)

Purification via column chromatography on silica gel (ethyl acetate/petroleum ether = 1/4, v/v) afforded **3j** as yellow oil (75.5 mg, 61% yield). ^{1}H NMR (400 MHz, $CDCl_3$): δ = 1.34 (t, J = 7.2 Hz, 3H), 2.31 (s, 3H), 3.34 (dd, J = 13.6 Hz, 5.2 Hz, 2H), 4.14 (dd, J = 13.6 Hz, 4.4 Hz, 2H), 4.21 (q, J = 7.2 Hz, 2H), 6.76 (d, J = 8.4 Hz, 2H), 6.85 (d, J

= 8.4 Hz, 2H), 7.03 (d, J = 8.8 Hz, 2H), 7.08-7.10 (m, 4H), 7.14-7.16 (m, 3H), 7.37-7.40 (m, 1H), 7.86 (td, J = 7.6 Hz, 1.2 Hz, 1H), 8.27 (d, J = 8.0 Hz, 1H), 8.46 (d, J = 4.4 Hz, 1H), 8.85 (s, 1H) ppm. ^{13}C NMR (100 MHz, $CDCl_3$): δ = 14.3, 20.8, 40.3, 41.1, 62.0, 67.4, 117.9, 119.3, 121.7, 126.2, 126.9, 128.3, 129.9, 130.2, 130.8, 131.1, 132.9, 136.4, 137.3, 148.5, 150.2, 154.7, 156.8, 164.1, 172.0 ppm. HRMS (ESI$^+$): calcd for $C_{31}H_{30}N_2NaO_4$ [M+Na]$^+$ 517.2103, found 517.2103.

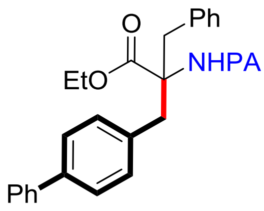

Ethyl 2-benzyl-3-(biphenyl-4-yl)-2-(picolinamido)propanoate (3k)

Purification via column chromatography on silica gel (ethyl acetate/petroleum ether = 1/4, v/v) afforded **3k** as yellow oil (75.3 mg, 65% yield). ^{1}H NMR (400 MHz, $CDCl_3$): δ = 1.35 (t, J = 7.2 Hz, 3H), 3.39 (dd, J = 13.6 Hz, 11.2 Hz, 2H), 4.16-4.26 (m, 4H), 7.09-7.13 (m, 2H), 7.14-7.17 (m, 5H), 7.27-7.31 (m, 1H), 7.36-7.40 (m, 5H), 7.50-7.52 (m, 2H), 7.88 (td, J = 7.6 Hz, 1.6 Hz, 1H), 8.30 (d, J = 8.0 Hz, 1H), 8.45-8.46 (m, 1H), 8.87 (s, 1H) ppm. ^{13}C NMR (100 MHz, $CDCl_3$): δ = 14.3, 40.8, 41.2, 62.1, 67.4, 121.7, 126.2, 126.96, 126.98, 127.1, 127.2, 128.3, 128.8, 129.9, 130.3, 135.6, 136.4, 137.3, 139.7, 141.0, 148.5, 150.3, 164.2, 172.0 ppm. HRMS (ESI$^+$): calcd for $C_{30}H_{28}N_2NaO_3$ [M+Na]$^+$ 487.1998, found 487.2001.

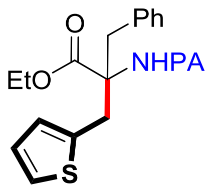

Ethyl 2-benzyl-2-(picolinamido)-3-(thiophen-2-yl)propanoate (3l)

Purification via column chromatography on silica gel (ethyl acetate/petroleum ether = 1/4, v/v) afforded **3l** as yellow oil (60.4 mg, 61% yield). ^{1}H NMR (400 MHz, $CDCl_3$): δ = 1.34 (t, J = 7.2 Hz, 3H), 3.30 (d, J = 13.6 Hz, 1H), 3.60 (d, J = 14.8 Hz, 1H), 4.07

(d, J = 13.2 Hz, 1H), 4.19-4.25 (m, 2H), 4.42 (d, J = 14.4 Hz, 1H), 6.75-6.76 (m, 1H), 6.80-6.82 (m, 1H), 7.05 (dd, J = 5.2 Hz, 1.2 Hz, 1H), 7.07-7.10 (m, 2H), 7.14-7.17 (m, 3H), 7.39 (ddd, J = 7.6 Hz, 4.8 Hz, 1.2 Hz, 1H), 7.86 (dd, J = 7.6 Hz, 1.6 Hz, 1H), 8.27 (d, J = 7.6 Hz, 1H), 8.47-8.48 (m, 1H), 8.91 (s, 1H) ppm. ^{13}C NMR (100 MHz, $CDCl_3$): δ = 14.3, 35.3, 40.9, 62.2, 67.1, 121.7, 124.7, 126.3, 126.6, 127.01, 127.04, 128.3, 129.8, 136.1, 137.3, 137.8, 148.5, 150.2, 164.0, 171.7 ppm. HRMS (ESI$^+$): calcd for $C_{22}H_{23}N_2O_3S$ $[M+H]^+$ 395.1429, found 395.1428.

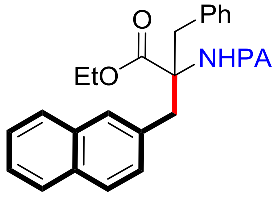

Ethyl 2-benzyl-3-(naphthalen-2-yl)-2-(picolinamido)propanoate (3m)

Purification via column chromatography on silica gel (ethyl acetate/petroleum ether = 1/4, v/v) afforded **3m** as yellow oil (95.0 mg, 87% yield). ^{1}H NMR (400 MHz, $CDCl_3$): δ = 1.34 (t, J = 7.2 Hz, 3H), 3.43 (d, J = 13.6 Hz, 1H), 3.55 (d, J = 13.6 Hz, 1H), 4.16-4.28 (m, 3H), 4.33 (d, J = 13.2 Hz, 1H), 7.11-7.19 (m, 5H), 7.23 (dd, J = 8.4 Hz, 1.6 Hz, 1H), 7.35-7.40 (m, 3H), 7.58-7.64 (m, 3H), 7.72-7.74 (m, 1H), 7.88 (td, J = 7.6 Hz, 1.6 Hz, 1H), 8.33 (d, J = 8.0 Hz, 1H), 8.39-8.40 (m, 1H), 8.85 (s, 1H) ppm. ^{13}C NMR (100 MHz, $CDCl_3$): δ = 14.3, 41.16, 41.20, 62.1, 67.5, 121.7, 125.6, 125.9, 126.2, 126.9, 127.6, 127.7, 127.8, 128.2, 128.3, 128.8, 129.9, 132.5, 133.4, 134.1, 136.4, 137.2, 148.5, 150.2, 164.3, 172.0 ppm. HRMS (ESI$^+$): calcd for $C_{28}H_{26}N_2NaO_3$ $[M+Na]^+$ 461.1841, found 461.1841.

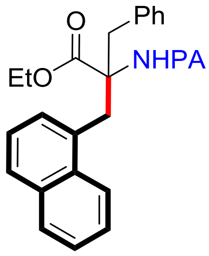

Ethyl 2-benzyl-3-(naphthalen-1-yl)-2-(picolinamido)propanoate (3n)

Purification via column chromatography on silica gel (ethyl acetate/petroleum ether =

1/4, v/v) afforded **3n** as yellow oil (73.9 mg, 67% yield). ^{1}H NMR (400 MHz, $CDCl_3$): δ = 1.13 (t, J = 7.2 Hz, 3H), 3.55 (d, J = 13.6 Hz, 1H), 3.84-3.92 (m, 1H), 3.97-4.05 (m, 2H), 4.36 (d, J = 13.6 Hz, 1H), 4.43 (d, J = 14.4 Hz, 1H), 7.15 (m, 5H), 7.29 (d, J = 7.6 Hz, 1H), 7.34-7.40 (m, 4H), 7.69 (d, J = 8.0 Hz, 1H), 7.78 (dd, J = 6.4 Hz, 3.6 Hz, 1H), 7.86 (td, J = 8.0 Hz, 1.6 Hz, 1H), 8.20 (dd, J = 6.4 Hz, 3.2 Hz, 1H), 8.29 (d, J = 7.6 Hz, 1H), 8.39 (d, J = 4.4 Hz, 1H), 8.94 (s, 1H) ppm. ^{13}C NMR (100 MHz, $CDCl_3$): δ = 13.9, 37.4, 40.7, 62.1, 66.9, 121.7, 124.2, 125.2, 125.4, 125.6, 126.2, 126.9, 127.7, 128.2, 128.3, 128.7, 130.0, 132.7, 132.8, 133.8, 136.5, 137.2, 148.4, 150.2, 164.4, 172.1 ppm. HRMS (ESI^{+}): calcd for $C_{28}H_{27}N_2O_3$ $[M+H]^{+}$ 439.2022, found 439.2020.

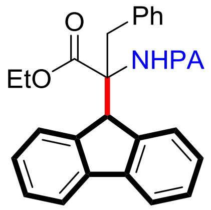

Ethyl 2-(9*H*-fluoren-9-yl)-3-phenyl-2-(picolinamido)propanoate (3o)

Purification via column chromatography on silica gel (ethyl acetate/petroleum ether = 1/4, v/v) afforded **3o** as yellow oil (57.7 mg, 50% yield). ^{1}H NMR (400 MHz, $CDCl_3$): δ = 0.67 (t, J = 7.2 Hz, 3H), 3.52-3.67 (m, 2H), 3.80 (d, J = 12.8 Hz, 1H), 4.68 (d, J = 12.8 Hz, 1H), 5.56 (s, 1H), 7.00-7.02 (m, 2H), 7.07-7.11 (m, 3H), 7.14 (t, J = 7.6 Hz, 1H), 7.32 (t, J = 7.6 Hz, 1H), 7.37 (t, J = 7.6 Hz, 1H), 7.42-7.50 (m, 3H), 7.70 (d, J = 7.6 Hz, 1H), 7.77 (d, J = 7.6 Hz, 1H), 7.90-7.94 (m, 2H), 8.34 (d, J = 7.6 Hz, 1H), 8.60-8.62 (m, 1H), 9.46 (s, 1H) ppm. ^{13}C NMR (100 MHz, $CDCl_3$): δ = 13.3, 40.2, 51.1, 61.5, 67.8, 119.4, 119.6, 122.0, 126.2, 126.4, 126.6, 126.9, 127.06, 127.10, 127.5, 127.9, 128.3, 129.8, 136.4, 137.4, 141.7, 142.5, 143.0, 143.8, 148.8, 150.4, 164.1, 170.2 ppm. HRMS (ESI^{+}): calcd for $C_{30}H_{27}N_2O_3$ $[M+H]^{+}$ 463.2022, found 463.2017.

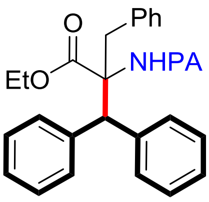

Ethyl 2-benzyl-3,3-diphenyl-2-(picolinamido)propanoate (3p)

Purification via column chromatography on silica gel (ethyl acetate/petroleum ether = 1/4, v/v) afforded **3p** as yellow oil (82.1 mg, 70% yield). ^{1}H NMR (400 MHz, $CDCl_3$): δ = 1.18 (t, J = 7.2 Hz, 3H), 3.37 (d, J = 13.6 Hz, 1H), 4.15 (q, J = 7.2 Hz, 2H), 4.25 (d, J = 13.6 Hz, 1H), 5.53 (s, 1H), 7.02-7.05 (m, 2H), 7.08-7.13 (m, 4H), 7.16-7.19 (m, 2H), 7.25-7.29 (m, 1H), 7.32-7.37 (m, 5H), 7.66 (d, J = 7.6 Hz, 2H), 7.80 (td, J = 8.0 Hz, 1.6 Hz, 1H), 8.16 (d, J = 7.6 Hz, 1H), 8.43 (d, J = 4.4 Hz, 1H), 9.18 (s, 1H) ppm. ^{13}C NMR (100 MHz, $CDCl_3$): δ = 13.8, 39.7, 57.6, 62.1, 69.2, 121.6, 126.0, 126.7, 126.8, 127.0, 128.1, 128.2, 129.6, 130.1, 131.1, 136.6, 137.2, 140.09, 140.13, 148.3, 150.3, 163.6, 172.2 ppm. HRMS (ESI$^+$): calcd for $C_{30}H_{28}N_2NaO_3$ [M+Na]$^+$ 487.1998, found 487.1994.

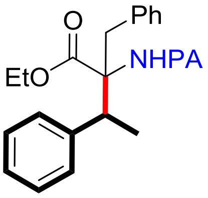

dr = 1:1

Ethyl 2-benzyl-3-phenyl-2-(picolinamido)butanoate (3q)

The ratio of the two diastereoisomers is 1:1. Purification via column chromatography on silica gel (ethyl acetate/petroleum ether = 1/4, v/v) afforded **3q** as yellow oil (92.5 mg, 92% yield). ^{1}H NMR (400 MHz, $CDCl_3$) of the mixture: δ = 1.28 (t, J = 7.2 Hz, 3H), 1.35 (t, J = 7.2 Hz, 3H), 1.45 (d, J = 7.2 Hz, 3H), 1.71 (d, J = 7.6 Hz, 3H), 3.01 (d, J = 13.6 Hz, 1H), 3.64 (d, J = 13.6 Hz, 1H), 4.09 (d, J = 13.2 Hz, 1H), 4.14-4.33 (m, 7H), 7.02-7.15 (m, 15H), 7.26-7.35 (m, 4H), 7.38-7.42 (m, 3H), 7.81-7.89 (m, 2H), 8.24 (d, J = 7.6 Hz, 1H), 8.27 (d, J = 8.0 Hz, 1H), 8.36-8.38 (m, 1H), 8.52-8.54 (m, 1H), 8.77 (s, 1H), 9.21 (s, 1H) ppm. ^{13}C NMR (100 MHz, $CDCl_3$) of the mixture: δ = 14.1, 14.2, 16.40, 16.41, 38.2, 38.4, 43.4, 45.6, 61.89, 61.94, 69.56, 69.64, 121.6,

121.7, 126.0, 126.2, 126.6, 126.7, 127.0, 127.1, 127.8, 128.01, 128.04, 128.2, 128.4, 129.8, 129.9, 130.0, 136.9, 137.0, 137.1, 137.3, 141.6, 142.0, 148.3, 148.4, 150.3, 150.4, 163.8, 163.9, 171.4, 171.9 ppm. HRMS (ESI^+): calcd for $C_{25}H_{26}N_2NaO_3$ $[M+Na]^+$ 425.1841, found 425.1835.

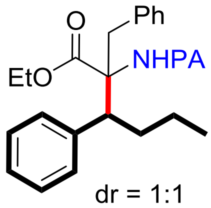

Ethyl 2-benzyl-3-phenyl-2-(picolinamido)hexanoate (3r)

The ratio of the two diastereoisomers is 1:1. Purification via column chromatography on silica gel (ethyl acetate/petroleum ether = 1/4, v/v) afforded **3r** as yellow oil (107.6 mg, 88% yield). ^1H NMR (400 MHz, $CDCl_3$) of the mixture: δ = 0.79 (t, J = 7.2 Hz, 3H), 0.92 (t, J = 7.2 Hz, 3H), 0.99-1.19 (m, 4H), 1.32 (t, J = 7.2 Hz, 3H), 1.36 (t, J = 7.2 Hz, 3H), 1.73-1.83 (m, 1H), 1.95-2.03 (m, 1H), 2.11-2.17 (m, 2H), 2.88 (d, J = 13.6 Hz, 1H), 3.69 (d, J = 13.6 Hz, 1H), 3.97 (dd, J = 12.4 Hz, 2.8 Hz, 1H), 4.03-4.07 (m, 2H), 4.16-4.33 (m, 5H), 7.00-7.07 (m, 7H), 7.09-7.14 (m, 8H), 7.25-7.34 (m, 4H), 7.37-7.42 (m, 3H), 7.80 (td, J = 7.6 Hz, 1.6 Hz, 1H), 7.87 (d, J = 7.6 Hz, 1.6 Hz, 1H), 8.20 (d, J = 8.0 Hz, 1H), 8.28 (d, J = 8.0 Hz, 1H), 8.33-8.34 (m, 1H), 8.53-8.54 (m, 1H), 8.67 (s, 1H), 9.23 (s, 1H) ppm. ^{13}C NMR (100 MHz, $CDCl_3$) of the mixture: δ = 14.0, 14.1, 14.3, 21.1, 21.5, 31.9, 32.0, 38.56, 38.58, 49.9, 51.6, 61.9, 69.3, 69.4, 121.5, 121.7, 125.9, 126.2, 126.6, 126.7, 126.9, 127.1, 127.9, 128.0, 128.1, 129.0, 129.9, 130.0, 130.5, 136.9, 137.0, 137.1, 137.3, 139.5, 139.9, 148.2, 148.4, 150.3, 150.5, 163.8, 171.6, 172.2 ppm. HRMS (ESI^+): calcd for $C_{27}H_{30}N_2NaO_3$ $[M+Na]^+$ 453.2154, found 453.2147.

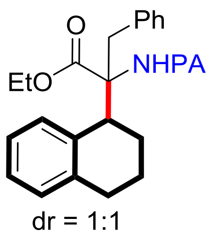

Ethyl 3-phenyl-2-(picolinamido)-2-(1,2,3,4-tetrahydronaphthalen-1-yl)propanoate (3s)

The ratio of the two diastereoisomers is 1:1. Purification via column chromatography on silica gel (ethyl acetate/petroleum ether = 1/4, v/v) afforded **3s** as yellow oil (82.4 mg, 77% yield). ^{1}H NMR (400 MHz, $CDCl_3$) of the mixture: δ = 0.97-1.03 (m, 6H), 1.49-1.58 (m, 1H), 1.60-1.76 (m, 2H), 1.81-1.89 (m, 1H), 2.02-2.16 (m, 2H), 2.27-2.42 (m, 2H), 2.55-2.62 (m, 1H), 2.66-2.78 (m, 3H), 3.48 (d, J = 13.2 Hz, 1H), 3.53 (d, J = 13.2 Hz, 1H), 3.87-3.97 (m, 3H), 3.99-4.05 (m, 1H), 4.19 (d, J = 13.2 Hz, 1H), 4.27 (d, J = 12.8 Hz, 1H), 4.41 (t, J = 8.0 Hz, 1H), 4.53 (dd, J = 8.0 Hz, 4.0 Hz, 1H), 6.94-6.97 (m, 3H), 6.99-7.04 (m, 4H), 7.05-7.10 (m, 7H), 7.17-7.22 (m, 2H), 7.38-7.44 (m, 3H), 7.48-7.50 (m, 1H), 7.84-7.90 (m, 2H), 8.23 (d, J = 7.6 Hz, 1H), 8.26 (d, J = 8.0 Hz, 1H), 8.54-8.56 (m, 2H), 9.26 (s, 1H), 9.32 (s, 1H) ppm. ^{13}C NMR (100 MHz, $CDCl_3$) of the mixture: δ = 13.5, 13.8, 20.4, 22.5, 24.7, 26.7, 29.5, 30.8, 37.9, 39.7, 42.4, 42.9, 61.7, 62.0, 69.3, 69.4, 121.8, 124.9, 125.6, 126.0, 126.2, 126.3, 126.5, 126.8, 128.1, 128.2, 128.8, 129.0, 129.1, 130.0, 131.6, 136.3, 136.7, 136.75, 136.81, 137.31, 137.34, 139.4, 139.8, 148.5, 148.6, 150.4, 150.6, 163.5, 163.6, 172.0, 173.1 ppm. HRMS (ESI$^+$): calcd for $C_{27}H_{28}N_2NaO_3$ $[M+Na]^+$ 451.1998, found 451.1998.

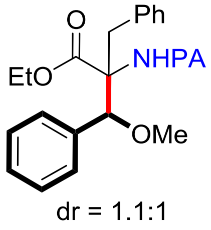

Ethyl 2-benzyl-3-methoxy-3-phenyl-2-(picolinamido)propanoate (3t)

The ratio of the two diastereoisomers is 1.1:1. Purification via column chromatography on silica gel (ethyl acetate/petroleum ether = 1/4, v/v) afforded **3t** as

yellow oil (65.9 mg, 63% yield). ^{1}H NMR (400 MHz, $CDCl_3$) of the mixture: δ = 1.25 (t, J = 7.2 Hz, 3H), 1.29 (t, J = 7.2 Hz, 3H), 2.91 (d, J = 13.6 Hz, 1H), 3.22 (s, 3H), 3.39 (s, 3H), 3.75 (d, J = 14.0 Hz, 1H), 4.00 (d, J = 13.6 Hz, 1H), 4.13 (d, J = 13.6 Hz, 1H), 4.13-4.27 (m, 4H), 5.18 (s, 1H), 5.37 (s, 1H), 6.99-7.02 (m, 2H), 7.07-7.11 (m, 5H), 7.13-7.16 (m, 3H), 7.22-7.25 (m, 5H), 7.35-7.43 (m, 5H), 7.51-7.53 (m, 2H), 7.81-7.89 (m, 2H), 8.16 (d, J = 8.0 Hz, 1H), 8.24 (d, J = 8.0 Hz, 1H), 8.46 (d, J = 4.8 Hz, 1H), 8.56 (d, J = 4.0 Hz, 1H), 8.86 (s, 1H), 9.29 (s, 1H) ppm. ^{13}C NMR (100 MHz, $CDCl_3$) of the mixture: δ = 14.0, 14.3, 36.4, 37.5, 58.1, 58.2, 61.9, 62.1, 69.6, 69.8, 84.1, 84.8, 121.7, 121.8, 126.17, 126.22, 126.6, 126.7, 127.77, 127.80, 128.08, 128.10, 128.2, 128.3, 129.0, 130.0, 130.3, 136.2, 136.7, 137.0, 137.25, 137.27, 148.4, 148.5, 150.2, 150.3, 163.8, 164.1, 170.47, 170.53 ppm. HRMS (ESI^{+}): calcd for $C_{25}H_{26}N_2NaO_4$ $[M+Na]^+$ 441.1790, found 441.1786.

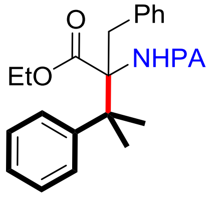

Ethyl 2-benzyl-3-methyl-3-phenyl-2-(picolinamido)butanoate (3u)

Purification via column chromatography on silica gel (ethyl acetate/petroleum ether = 1/5, v/v) afforded **3u** as pale yellow oil (68.3 mg, 66% yield). ^{1}H NMR (400 MHz, $CDCl_3$): δ = 1.07 (t, J = 7.2 Hz, 3H), 1.66 (s, 3H), 1.80 (s, 3H), 3.50 (d, J = 13.6 Hz, 1H), 3.91 (q, J = 7.2 Hz, 2H), 4.59 (d, J = 14.0 Hz, 1H), 7.02-7.09 (m, 3H), 7.18-7.19 (m, 2H), 7.22-7.30 (m, 3H), 7.39 (ddd, J = 7.6 Hz, 4.8 Hz, 0.8 Hz, 1H), 7.42-7.44 (m, 2H), 7.83 (td, J = 7.6 Hz, 1.6 Hz, 1H), 8.17 (d, J = 7.6 Hz, 1H), 8.53-8.54 (m, 1H), 9.32 (s, 1H) ppm. ^{13}C NMR (100 MHz, $CDCl_3$): δ = 13.7, 26.1, 26.3, 34.4, 48.2, 61.7, 72.2, 121.9, 126.0, 126.3, 126.7, 127.7, 127.8, 127.9, 130.1, 137.3, 137.5, 145.3, 148.3, 150.9, 163.7, 171.7 ppm. HRMS (ESI^{+}): calcd for $C_{26}H_{29}N_2O_3$ $[M+H]^+$ 417.2178, found 417.2170.

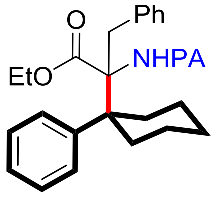

Ethyl 3-phenyl-2-(1-phenylcyclohexyl)-2-(picolinamido)propanoate (3v)

Purification via column chromatography on silica gel (ethyl acetate/petroleum ether = 1/6, v/v) afforded **3v** as pale yellow oil (59.4 mg, 52% yield). ^{1}H NMR (400 MHz, $CDCl_3$): δ = 1.05 (t, J = 7.2 Hz, 3H), 1.13-1.33 (m, 3H), 1.49-1.64 (m, 3H), 1.95-2.14 (m, 2H), 2.48 (d, J = 13.6 Hz, 1H), 2.91 (d, J = 13.6 Hz, 1H), 3.46 (d, J = 14.0 Hz, 1H), 3.88 (q, J = 7.2 Hz, 2H), 4.60 (d, J = 14.0 Hz, 1H), 7.00-7.07 (m, 3H), 7.16-7.18 (m, 2H), 7.22-7.32 (m, 5H), 7.38 (ddd, J = 7.2 Hz, 5.6 Hz, 1H), 7.83 (t, J = 7.6 Hz, 1H), 8.17 (d, J = 7.6 Hz, 1H), 8.52 (d, J = 4.8 Hz, 1H), 9.27 (s, 1H) ppm. ^{13}C NMR (100 MHz, $CDCl_3$): δ = 13.7, 22.6, 22.7, 26.4, 31.6, 31.7, 33.8, 53.6, 61.7, 73.3, 121.9, 125.9, 126.2, 126.5, 127.87, 127.92, 130.1, 137.3, 137.7, 139.3, 148.2, 151.0, 163.7, 171.6 ppm. HRMS (ESI^{+}): calcd for $C_{29}H_{32}N_2NaO_3$ $[M+Na]^+$ 479.2311, found 479.2311.

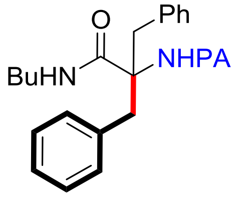

***N*-(2-Benzyl-1-(butylamino)-1-oxo-3-phenylpropan-2-yl)picolinamide (3w)**

Purification via column chromatography on silica gel (ethyl acetate/petroleum ether = 1/2, v/v) afforded **3w** as white soild (35.2 mg, 34% yield). M.p.: 170-172 °C. ^{1}H NMR (400 MHz, $CDCl_3$): δ = 0.85 (t, J = 7.2 Hz, 3H), 1.18-1.25 (m, 2H), 1.30-1.37 (m, 2H), 3.17 (q, J = 6.8 Hz, 2H), 3.43 (d, J = 13.6 Hz, 2H), 3.61 (d, J = 13.6 Hz, 2H), 5.93 (br. s, 1H), 7.19-7.20 (m, 10H), 7.41-7.44 (m, 1H), 7.87 (t, J = 7.6 Hz, 1H), 8.22 (d, J = 7.6 Hz, 1H), 8.49 (d, J = 4.0 Hz, 1H), 8.69 (s, 1H) ppm. ^{13}C NMR (100 MHz, $CDCl_3$): δ = 13.9, 20.2, 31.4, 39.8, 41.2, 65.5, 122.0, 126.5, 127.0, 128.4, 130.6, 136.2, 137.5, 148.4, 150.0, 164.6, 171.4 ppm. HRMS (ESI^{+}): calcd for $C_{26}H_{29}N_3NaO_2$ $[M+Na]^+$ 438.2157, found 438.2162.

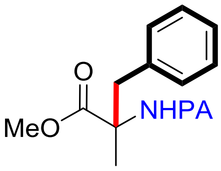

Methyl 2-methyl-3-phenyl-2-(picolinamido)propanoate (4b)

Purification via column chromatography on silica gel (ethyl acetate/petroleum ether = 1/4, v/v) afforded **4b** as yellow oil (52.5 mg, 70% yield). ^{1}H NMR (400 MHz, $CDCl_3$): δ = 1.73 (s, 3H), 3.35 (d, J = 13.6 Hz, 1H), 3.57 (d, J = 13.2 Hz, 1H), 3.78 (s, 3H), 7.07-7.09 (m, 2H), 7.19-7.23 (m, 3H), 7.39-7.42 (m, 1H), 7.84 (td, J = 7.6 Hz, 1.6 Hz, 1H), 8.19 (d, J = 7.6 Hz, 1H), 8.51 (d, J = 4.4 Hz, 1H), 8.61 (s, 1H) ppm. ^{13}C NMR (100 MHz, $CDCl_3$): δ = 23.2, 41.9, 52.7, 60.9, 122.0, 126.3, 127.0, 128.3, 130.2, 136.3, 137.4, 148.3, 150.0, 163.8, 174.1 ppm. HRMS (ESI$^+$): calcd for $C_{17}H_{18}N_2NaO_3$ $[M+Na]^+$ 321.1215, found 321.1215.

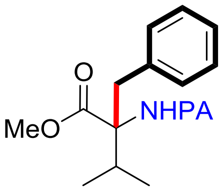

Methyl 2-benzyl-3-methyl-2-(picolinamido)butanoate (4c)

Purification via column chromatography on silica gel (ethyl acetate/petroleum ether = 1/4, v/v) afforded **4c** as yellow oil (67.4 mg, 83% yield). ^{1}H NMR (400 MHz, $CDCl_3$): δ = 0.98 (d, J = 7.2 Hz, 3H), 1.18 (d, J = 6.8 Hz, 3H), 2.84-2.91 (m, 1H), 3.45 (d, J = 13.6 Hz, 1H), 3.83 (s, 3H), 3.95 (d, J = 13.6 Hz, 1H), 7.05-7.08 (m, 2H), 7.10-7.15 (m, 3H), 7.39 (ddd, J = 7.6 Hz, 4.8 Hz, 0.8 Hz, 1H), 7.85 (td, J = 7.6 Hz, 1.6 Hz, 1H), 8.20 (d, J = 7.6 Hz, 1H), 8.50 (d, J = 4.8 Hz, 1H), 8.91 (s, 1H) ppm. ^{13}C NMR (100 MHz, $CDCl_3$): δ = 18.1, 18.3, 33.5, 37.5, 52.4, 69.2, 121.8, 126.2, 126.7, 128.2, 130.0, 137.2, 137.3, 148.4, 150.4, 163.6, 172.6 ppm. HRMS (ESI$^+$): calcd for $C_{19}H_{22}N_2NaO_3$ $[M+Na]^+$ 349.1528, found 349.1522.

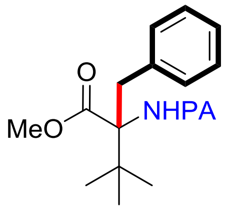

Meth 2-benzyl-3,3-dimethyl-2-(picolinamido)butanoate (4d)

Purification via column chromatography on silica gel (ethyl acetate/petroleum ether = 1/4, v/v) afforded **4d** as yellow oil (75.7 mg, 89% yield). ^{1}H NMR (400 MHz, $CDCl_3$): δ = 1.18 (s, 9H), 3.43 (d, J = 14.0 Hz, 1H), 3.76 (s, 3H), 4.40 (d, J = 14.0 Hz, 1H), 7.05-7.13 (m, 3H), 7.20-7.22 (m, 2H), 7.39 (ddd, J = 7.6 Hz, 5.2 Hz, 1.2 Hz, 1H), 7.82 (td, J = 7.6 Hz, 1.6 Hz, 1H), 8.18 (d, J = 8.0 Hz, 1H), 8.55-8.56 (m, 1H), 9.35 (s, 1H) ppm. ^{13}C NMR (100 MHz, $CDCl_3$): δ = 27.5, 33.7, 41.2, 52.3, 71.8, 121.9, 126.0, 126.3, 128.0, 130.0, 137.4, 137.8, 148.2, 150.9, 163.7, 173.1 ppm. HRMS (ESI$^+$): calcd for $C_{20}H_{24}N_2NaO_3$ $[M+Na]^+$ 363.1685, found 363.1690.

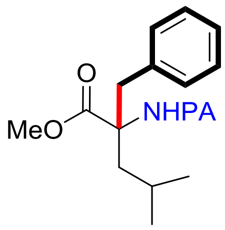

Methyl 2-benzyl-4-methyl-2-(picolinamido)pentanoate (4e)

Purification via column chromatography on silica gel (ethyl acetate/petroleum ether = 1/4, v/v) afforded **4e** as yellow oil (76.8 mg, 90% yield). ^{1}H NMR (400 MHz, $CDCl_3$): δ = 0.81 (d, J = 6.8 Hz, 3H), 0.90 (d, J = 6.4 Hz, 3H), 1.60-1.70 (m, 1H), 1.94 (dd, J = 14.0 Hz, 7.6 Hz, 1H), 2.81 (dd, J = 14.4 Hz, 5.6 Hz, 1H), 3.14 (d, J = 13.6 Hz, 1H), 3.78 (s, 3H), 3.94 (d, J = 13.2 Hz, 1H), 6.98-7.01 (m, 2H), 7.12-7.14 (m, 3H), 7.39 (dd, J = 7.6 Hz, 4.8 Hz, 1H), 7.84 (td, J = 8.0 Hz, 1.6 Hz, 1H), 8.22 (d, J = 7.6 Hz, 1H), 8.51 (d, J = 4.8 Hz, 1H), 8.99 (s, 1H) ppm. ^{13}C NMR (100 MHz, $CDCl_3$): δ = 22.7, 23.8, 25.0, 41.8, 44.1, 52.4, 65.6, 121.7, 126.2, 126.8, 128.2, 129.8, 136.4, 137.3, 148.4, 150.2, 163.6, 174.0 ppm. HRMS (ESI$^+$): calcd for $C_{20}H_{24}N_2NaO_3$ $[M+Na]^+$ 363.1685, found 363.1680.

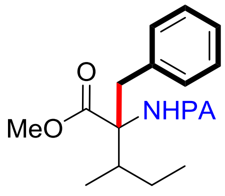

Methyl 2-benzyl-3-methyl-2-(picolinamido)pentanoate (4f)

The ratio of the two diastereoisomers is 1:1. Purification via column chromatography on silica gel (ethyl acetate/petroleum ether = 1/4, v/v) afforded **4f** as yellow oil (63.2 mg, 74% yield). ^{1}H NMR (400 MHz, $CDCl_3$) of the mixture: δ = 0.88 (t, J = 6.8 Hz, 3H), 0.91-0.95 (m, 1H), 0.96-1.00 (m, 6H), 1.17 (d, J = 7.2 Hz, 3H), 1.21-1.30 (m, 1H), 1.74-1.83 (m, 1H), 1.87-1.96 (m, 1H), 2.48-2.57 (m, 1H), 2.58-2.66 (m, 1H), 3.44 (d, J = 13.2 Hz, 1H), 3.48 (d, J = 13.6 Hz, 1H), 3.80 (s, 3H), 3.81 (s, 3H), 3.96 (d, J = 11.2 Hz, 1H), 3.99 (d, J = 10.8 Hz, 1H), 7.04-7.08 (m, 4H), 7.09-7.13 (m, 6H), 7.36-7.40 (m, 2H), 7.81-7.85 (m, 2H), 8.20 (dd, J = 8.0 Hz, 0.8 Hz, 2H), 8.49 (d, J = 4.4 Hz, 2H), 8.90 (s, 1H), 8.96 (s, 1H) ppm. ^{13}C NMR (100 MHz, $CDCl_3$) of the mixture: δ = 12.6, 12.8, 14.1, 14.2, 24.6, 24.7, 37.3, 37.6, 40.4, 52.3, 69.1, 69.2, 121.73, 121.74, 126.11, 126.14, 126.6, 128.1, 129.8, 130.0, 137.10, 137.13, 137.27, 137.29, 148.3, 150.27, 150.34, 163.4, 163.5, 172.7, 172.8 ppm. HRMS (ESI$^+$): calcd for $C_{20}H_{24}N_2NaO_3$ $[M+Na]^+$ 363.1685, found 363.1682.

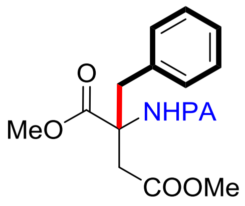

Dimethyl 2-benzyl-2-(picolinamido)succinate (4g)

Purification via column chromatography on silica gel (ethyl acetate/petroleum ether = 1/3, v/v) afforded **4g** as a pale yellow solid (84.6 mg, 94% yield). ^{1}H NMR (400 MHz, $CDCl_3$): δ = 3.17 (d, J = 11.2 Hz, 1H), 3.20 (d, J = 8.0 Hz, 1H), 3.58 (s, 3H), 3.78 (s, 3H), 3.88 (d, J = 9.6 Hz, 1H), 3.92 (d, J = 12.8 Hz, 1H), 7.00-7.03 (m, 2H), 7.16-7.18 (m, 3H), 7.39 (dd, J = 6.8 Hz, 4.8 Hz, 1H), 7.83 (td, J = 7.6 Hz, 1.2 Hz, 1H), 8.18 (d, J = 8.0 Hz, 1H), 8.51-8.52 (m, 1H), 9.04 (s, 1H) ppm. ^{13}C NMR (100 MHz, $CDCl_3$): δ

= 39.7, 40.9, 51.9, 52.9, 62.9, 121.8, 126.3, 127.2, 128.3, 129.9, 135.2, 137.2, 148.5, 149.8, 164.0, 170.8, 172.1 ppm. HRMS (ESI$^+$): calcd for $C_{19}H_{20}N_2NaO_5$ $[M+Na]^+$ 379.1270, found 379.1269.

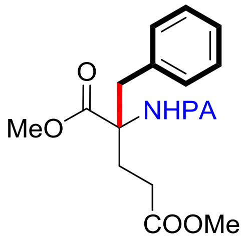

Dimethyl 2-benzyl-2-(picolinamido)pentanedioate (4h)

Purification via column chromatography on silica gel (ethyl acetate/petroleum ether = 1/3, v/v) afforded **4h** as yellow oil (67.9 mg, 73% yield). ^{1}H NMR (400 MHz, $CDCl_3$): δ = 2.13-2.24 (m, 1H), 2.33-2.46 (m, 2H), 3.02-3.11 (m, 1H), 3.24 (d, J = 13.6 Hz, 1H), 3.59 (s, 3H), 3.79 (s, 3H), 3.89 (d, J = 13.6 Hz, 1H), 6.99-7.01 (m, 2H), 7.13-7.15 (m, 3H), 7.39-7.42 (m, 1H), 7.84 (td, J = 7.6 Hz, 0.8 Hz, 1H), 8.20 (d, J = 7.6 Hz, 1H), 8.50-8.51 (m, 1H), 8.84 (s, 1H) ppm. ^{13}C NMR (100 MHz, $CDCl_3$): δ = 29.5, 30.6, 40.9, 51.8, 52.9, 65.3, 121.8, 126.4, 127.0, 128.3, 129.8, 136.0, 137.3, 148.4, 149.8, 163.7, 172.8, 173.0 ppm. HRMS (ESI$^+$): calcd for $C_{20}H_{22}N_2NaO_5$ $[M+Na]^+$ 393.1426, found 393.1432.

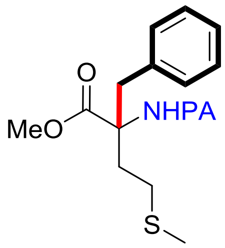

Methyl 2-benzyl-4-(methylthio)-2-(picolinamido)butanoate (4i)

Purification via column chromatography on silica gel (ethyl acetate/petroleum ether = 1/4, v/v) afforded **4i** as yellow oil (54.0 mg, 60% yield). ^{1}H NMR (400 MHz, $CDCl_3$): δ = 2.05 (s, 3H), 2.25-2.39 (m, 2H), 2.46-2.54 (m, 1H), 3.06-3.14 (m, 1H), 3.19 (d, J = 13.6 Hz, 1H), 3.79 (s, 3H), 3.89 (d, J = 13.6 Hz, 1H), 6.99-7.01 (m, 2H), 7.14-7.16 (m, 3H), 7.41 (ddd, J = 7.6 Hz, 4.8 Hz, 1.2 Hz, 1H), 7.85 (td, J = 7.6 Hz, 1.6 Hz, 1H), 8.19-8.21 (m, 1H), 8.52 (dq, J = 4.8 Hz, 0.8 Hz, 1H), 8.90 (s, 1H) ppm. ^{13}C NMR

(100 MHz, $CDCl_3$): δ = 15.7, 29.1, 34.9, 41.1, 52.8, 65.6, 121.8, 126.4, 127.0, 128.3, 129.8, 136.0, 137.4, 148.4, 149.9, 163.7, 173.0 ppm. HRMS (ESI^+): calcd for $C_{19}H_{22}N_2NaO_3S$ $[M+Na]^+$ 381.1249, found 381.1252.

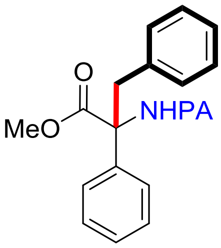

Methyl 2,3-diphenyl-2-(picolinamido)propanoate (4j)

Purification via column chromatography on silica gel (ethyl acetate/petroleum ether = 1/4, v/v) afforded **4j** as yellow oil (62.0 mg, 69% yield). ^{1}H NMR (400 MHz, $CDCl_3$): δ = 3.75 (s, 3H), 3.96 (d, J = 13.2 Hz, 1H), 4.23 (d, J = 13.2 Hz, 1H), 7.01-7.04 (m, 2H), 7.12-7.19 (m, 3H), 7.30-7.33 (m, 1H), 7.36-7.40 (m, 2H), 7.42 (ddd, J = 7.6 Hz, 4.8 Hz, 1.2 Hz, 1H), 7.54-7.56 (m, 2H), 7.83 (td, J = 8.0 Hz, 1.6 Hz, 1H), 8.15 (dd, J = 8.0 Hz, 0.8 Hz, 1H), 8.55 (dq, J = 4.8 Hz, 0.8 Hz, 1H), 9.37 (s, 1H) ppm. ^{13}C NMR (100 MHz, $CDCl_3$): δ = 38.9, 53.2, 66.6, 122.0, 126.1, 126.4, 127.0, 128.0, 128.2, 128.7, 130.2, 136.2, 137.3, 139.7, 148.4, 150.0, 163.4, 172.6 ppm. HRMS (ESI^+): calcd for $C_{22}H_{21}N_2O_3$ $[M+H]^+$ 361.1552, found 361.1548.

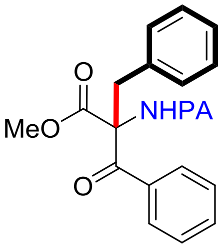

Methyl 2-benzyl-3-oxo-3-phenyl-2-(picolinamido)propanoate (4k)

Purification via column chromatography on silica gel (ethyl acetate/petroleum ether = 1/3, v/v) afforded **4k** as yellow oil (62.6 mg, 64% yield). ^{1}H NMR (400 MHz, $CDCl_3$): δ = 3.79 (s, 3H), 3.86-3.94 (m, 2H), 6.94-6.96 (m, 2H), 7.15-7.22 (m, 3H), 7.34 (t, J = 7.6 Hz, 2H), 7.40-7.47 (m, 2H), 7.78 (td, J = 8.0 Hz, 1.6 Hz, 1H), 7.97-8.00 (m, 2H), 8.03 (d, J = 7.6 Hz, 1H), 8.55 (d, J = 4.4 Hz, 1H), 9.37 (s, 1H) ppm. ^{13}C NMR (100 MHz, $CDCl_3$): δ = 39.1, 53.7, 71.0, 122.3, 126.6, 127.3, 128.4, 128.62, 128.64, 130.2,

133.1, 134.9, 135.3, 137.3, 148.6, 149.1, 163.2, 169.6, 191.1 ppm. HRMS (ESI$^+$): calcd for $C_{23}H_{21}N_2O_4$ $[M+H]^+$ 389.1501, found 389.1501.

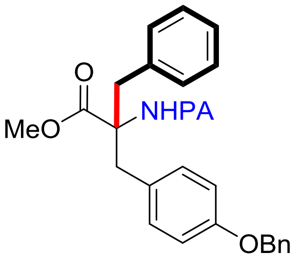

Methyl 2-benzyl-3-(4-(benzyloxy)phenyl)-2-(picolinamido)propanoate (4l)

Purification via column chromatography on silica gel (ethyl acetate/petroleum ether = 1/4, v/v) afforded **4l** as yellow oil (69.0 mg, 57% yield). ^{1}H NMR (400 MHz, $CDCl_3$): δ = 3.30 (d, J = 14.0 Hz, 1H), 3.34 (d, J = 13.6 Hz, 1H), 3.77 (s, 3H), 4.10 (t, J = 13.6 Hz, 2H), 4.95 (s, 2H), 6.76-6.78 (m, 2H), 6.98-7.00 (m, 2H), 7.05-7.08 (m, 2H), 7.12-7.16 (m, 3H), 7.30-7.41 (m, 6H), 7.88 (td, J = 7.6 Hz, 1.6 Hz, 1H), 8.28 (d, J = 8.0 Hz, 1H), 8.45 (d, J = 4.4 Hz, 1H), 8.78 (s, 1H) ppm. ^{13}C NMR (100 MHz, $CDCl_3$): δ = 40.3, 41.0, 52.5, 67.8, 70.0, 114.6, 121.7, 126.2, 126.9, 127.6, 128.0, 128.3, 128.6, 128.7, 129.8, 130.8, 136.4, 137.1, 137.3, 148.5, 150.2, 157.8, 164.1, 172.6 ppm. HRMS (ESI$^+$): calcd for $C_{30}H_{29}N_2O_4$ $[M+H]^+$ 481.2127, found 481.2123.

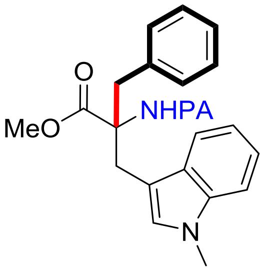

Methyl 2-benzyl-3-(1-methyl-1*H*-indol-3-yl)-2-(picolinamido)propanoate (4m)

Purification via column chromatography on silica gel (ethyl acetate/petroleum ether = 1/4, v/v) afforded **4m** as yellow oil (70.0 mg, 65% yield). ^{1}H NMR (400 MHz, $CDCl_3$): δ = 3.47 (d, J = 13.2 Hz, 1H), 3.59 (s, 3H), 3.61 (d, J = 13.6 Hz, 1H), 3.66 (s, 3H), 4.19 (d, J = 13.6 Hz, 1H), 4.25 (d, J = 14.4 Hz, 1H), 6.75 (s, 1H), 7.01 (t, J = 7.6 Hz, 1H), 7.10-7.03 (m, 3H), 7.15-7.21 (m, 4H), 7.37 (ddd, J = 7.6 Hz, 4.8 Hz, 0.8 Hz,

1H), 7.58 (d, J = 8.0 Hz, 1H), 7.88 (td, J = 7.6 Hz, 1.6 Hz, 1H), 8.31 (d, J = 8.0 Hz, 1H), 8.43 (d, J = 4.8 Hz, 1H), 8.87 (s, 1H) ppm. ^{13}C NMR (100 MHz, $CDCl_3$): δ = 31.3, 32.7, 40.8, 52.6, 67.4, 108.7, 109.1, 118.8, 119.1, 121.3, 121.7, 126.2, 126.9, 128.2, 128.3, 128.4, 129.8, 136.5, 136.7, 137.3, 148.4, 150.4, 164.1, 173.0 ppm. HRMS (ESI$^+$): calcd for $C_{26}H_{25}N_3NaO_3$ [M+Na]$^+$ 450.1794, found 450.1792.

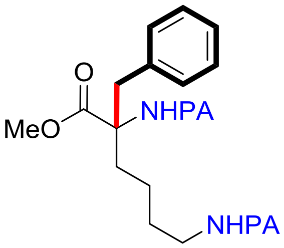

Methyl 2-benzyl-2,6-bis(picolinamido)hexanoate (4n)

Purification via column chromatography on silica gel (ethyl acetate/petroleum ether = 1/1, v/v) afforded **4n** as yellow oil (56.2 mg, 49% yield). ^{1}H NMR (400 MHz, $CDCl_3$): δ = 1.13-1.25 (m, 1H), 1.37-1.48 (m, 1H), 1.61-1.68 (m, 2H), 2.05 (td, J = 13.2 Hz, 4.4 Hz, 1H), 2.78 (td, J = 13.2 Hz, 4.4 Hz, 1H), 3.21 (d, J = 13.6 Hz, 1H), 3.42 (q, J = 6.8 Hz, 2H), 3.77 (s, 3H), 3.86 (d, J = 13.6 Hz, 1H), 6.99-7.01 (m, 2H), 7.13-7.14 (m, 3H), 7.37-7.42 (m, 2H), 7.79-7.87 (m, 2H), 8.02 (m, 1H), 8.16 (d, J = 7.6 Hz, 1H), 8.19 (d, J = 8.0 Hz, 1H), 8.47 (dq, J = 4.4 Hz, 0.8 Hz, 1H), 8.51 (dq, J = 4.8 Hz, 0.8 Hz, 1H), 8.84 (s, 1H) ppm. ^{13}C NMR (100 MHz, $CDCl_3$): δ = 21.9, 29.5, 35.1, 39.1, 40.9, 52.8, 66.0, 121.8, 122.2, 126.1, 126.3, 126.9, 128.3, 129.8, 136.4, 137.3, 137.4, 148.1, 148.4, 150.1, 163.6, 164.3, 173.4 ppm. HRMS (ESI$^+$): calcd for $C_{26}H_{29}N_4O_4$ [M+H]$^+$ 461.2189, found 461.2187.

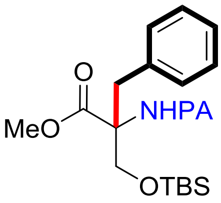

Methyl 2-benzyl-3-(*tert*-butyldimethylsilyloxy)-2-(picolinamido)propanoate (4o)

Purification via column chromatography on silica gel (ethyl acetate/petroleum ether = 1/4, v/v) afforded **4o** as yellow oil (58.4 mg, 54% yield). ^{1}H NMR (400 MHz, $CDCl_3$):

δ = -0.06 (s, 3H), -0.03 (s, 3H), 0.81 (s, 9H), 3.29 (d, *J* = 13.2 Hz, 1H), 3.66 (d, *J* = 13.2 Hz, 1H), 3.77 (s, 3H), 4.01 (d, *J* = 9.6 Hz, 1H), 4.35 (d, *J* = 9.6 Hz, 1H), 7.09-7.12 (m, 2H), 7.16-7.21 (m, 3H), 7.40 (ddd, *J* = 7.6 Hz, 4.8 Hz, 1.2 Hz, 1H), 7.83 (td, *J* = 7.6 Hz, 1.6 Hz, 1H), 8.18 (d, *J* = 7.6 Hz, 1H), 8.52 (dq, *J* = 4.8 Hz, 0.8 Hz, 1H), 8.70 (s, 1H) ppm. ^{13}C NMR (100 MHz, $CDCl_3$): *δ* = -5.51, -5.45, 18.1, 25.7, 36.9, 52.5, 63.9, 66.6, 121.8, 126.2, 126.9, 128.3, 130.1, 135.8, 137.3, 148.3, 150.0, 163.9, 172.0 ppm. HRMS (ESI$^+$): calcd for $C_{23}H_{32}N_2NaO_4Si$ $[M+Na]^+$ 451.2029, found 451.2025.

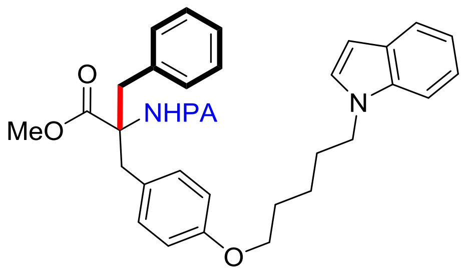

Methyl 2-(4-(5-(1*H*-indol-1-yl)pentyloxy)benzyl)-3-phenyl-2-(picolinamido) propanoate (4p)

Purification via column chromatography on silica gel (ethyl acetate/petroleum ether = 1/4, v/v) afforded **4p** as yellow oil (90.7 mg, 63% yield). ^{1}H NMR (400 MHz, $CDCl_3$): *δ* = 1.42-1.50 (m, 2H), 1.72-1.79 (m, 2H), 1.86-1.93 (m, 2H), 3.33 (d, *J* = 13.6 Hz, 1H), 3.37 (d, *J* = 13.2 Hz, 1H), 3.73-3.88 (m, 2H), 3.79 (s, 3H), 4.10-4.17 (m, 4H), 6.51 (d, *J* = 2.8 Hz, 1H), 6.68 (d, *J* = 8.4 Hz, 2H), 7.00 (d, *J* = 8.4 Hz, 2H), 7.10-7.24 (m, 8H), 7.35-7.41 (m, 2H), 7.66 (d, *J* = 7.6 Hz, 1H), 7.88 (t, *J* = 7.2 Hz, 1H), 8.31 (d, *J* = 8.0 Hz, 1H), 8.46 (dd, *J* = 4.8 Hz, 0.8 Hz, 1H), 8.81 (s, 1H) ppm. ^{13}C NMR (100 MHz, $CDCl_3$): *δ* = 23.7, 29.0, 30.1, 40.2, 40.9, 46.3, 52.5, 67.4, 67.8, 101.0, 109.4, 114.2, 119.3, 121.0, 121.4, 121.7, 126.2, 126.9, 127.9, 128.26, 128.28, 128.6, 129.8, 130.7, 135.9, 136.4, 137.3, 148.4, 150.1, 157.9, 164.0, 172.5 ppm. HRMS (ESI$^+$): calcd for $C_{36}H_{37}N_3NaO_4$ $[M+Na]^+$ 598.2682, found 598.2685.

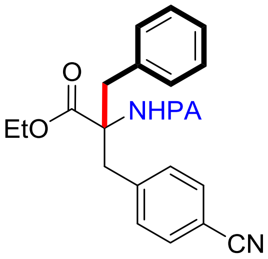

Ethyl 2-benzyl-3-(4-cyanophenyl)-2-(picolinamido)propanoate (4q)

Purification via column chromatography on silica gel (ethyl acetate/petroleum ether = 1/4, v/v) afforded **4q** as yellow oil (66.9 mg, 65% yield). ^{1}H NMR (400 MHz, $CDCl_3$): δ = 1.31 (t, J = 7.2 Hz, 3H), 3.32 (d, J = 13.6 Hz, 1H), 3.41 (d, J = 13.6 Hz, 1H), 4.12 (d, J = 13.6 Hz, 1H), 4.20 (q, J = 7.2 Hz, 2H), 4.26 (d, J = 13.2 Hz, 1H), 7.03-7.06 (m, 2H), 7.12-7.15 (m, 3H), 7.19 (d, J = 8.0 Hz, 2H), 7.39-7.44 (m, 3H), 7.88 (td, J = 7.6 Hz, 1.6 Hz, 1H), 8.25 (d, J = 8.0 Hz, 1H), 8.43-8.45 (m, 1H), 8.80 (s, 1H) ppm. ^{13}C NMR (100 MHz, $CDCl_3$): δ = 14.3, 41.0, 41.3, 62.3, 67.0, 110.9, 118.9, 121.7, 126.5, 127.1, 128.3, 129.8, 130.6, 132.0, 135.8, 137.4, 142.1, 148.5, 149.8, 164.3, 171.5 ppm. HRMS (ESI$^+$): calcd for $C_{25}H_{23}N_3NaO_3$ $[M+Na]^+$ 436.1637, found 436.1638.

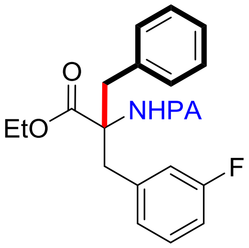

Ethyl 2-benzyl-3-(3-fluorophenyl)-2-(picolinamido)propanoate (4r)

Purification via column chromatography on silica gel (ethyl acetate/petroleum ether = 1/4, v/v) afforded **4q** as yellow oil (75.3 mg, 74% yield). ^{1}H NMR (400 MHz, $CDCl_3$): δ = 1.34 (t, J = 7.2 Hz, 3H), 3.33 (d, J = 8.0 Hz, 1H), 3.36 (d, J = 8.0 Hz, 1H), 4.14 (d, J = 13.2 Hz, 1H), 4.19 (d, J = 13.2 Hz, 1H), 4.22 (q, J = 7.2 Hz, 2H), 6.80-6.88 (m, 3H), 7.07-7.10 (m, 3H), 7.12-7.15 (m, 3H), 7.38 (dd, J = 7.2 Hz, 4.8 Hz, 1H), 7.86 (td, J = 8.0 Hz, 1.2 Hz, 1H), 8.27 (d, J = 7.6 Hz, 1H), 8.44 (d, J = 4.4 Hz, 1H), 8.84 (s, 1H) ppm. ^{13}C NMR (100 MHz, $CDCl_3$): δ = 14.3, 40.7, 41.2, 62.2, 67.2, 113.9 (d, J = 20.0 Hz), 116.7 (d, J = 22.0 Hz), 121.7, 125.6 (d, J = 3.0 Hz), 126.3, 127.0, 128.3, 129.6 (d, J = 8.0 Hz), 129.9, 136.2, 137.3, 138.9 (d, J = 8.0 Hz), 148.5, 150.1, 162.7 (d, J =

243.0 Hz), 164.2, 171.8 ppm. HRMS (ESI$^+$): calcd for $C_{24}H_{23}FN_2NaO_3$ [M+Na]$^+$ 429.1590, found 429.1595.

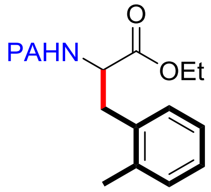

Ethyl 2-(picolinamido)-3-*o*-tolylpropanoate (5b)

Purification via column chromatography on silica gel (ethyl acetate/petroleum ether = 1/3, v/v) afforded **5b** as yellow oil (36.8 mg, 47% yield). ^{1}H NMR (400 MHz, $CDCl_3$): δ = 1.17 (t, J = 7.2 Hz, 3H), 2.39 (s, 3H), 3.17-3.27 (m, 2H), 4.08-4.20 (m, 2H), 4.98-5.04 (m, 1H), 7.07-7.17 (m, 4H), 7.42 (ddd, J = 7.6 Hz, 4.8 Hz, 1.2 Hz, 1H), 7.82 (td, J = 7.6 Hz, 1.6 Hz, 1H), 8.13 (d, J = 8.0 Hz, 1H), 8.52 (d, J = 8.0 Hz, 1H), 8.55-8.57 (m, 1H) ppm. ^{13}C NMR (100 MHz, $CDCl_3$): δ = 14.1, 19.5, 36.4, 52.8, 61.5, 122.3, 126.0, 126.4, 127.2, 130.0, 130.6, 134.6, 136.8, 137.3, 148.4, 149.4, 164.1, 171.9 ppm. HRMS (ESI$^+$): calcd for $C_{18}H_{20}N_2NaO_3$ [M+Na]$^+$ 335.1372, found 335.1372.

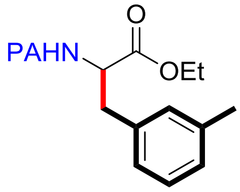

Ethyl 2-(picolinamido)-3-*m*-tolylpropanoate (5c)

Purification via column chromatography on silica gel (ethyl acetate/petroleum ether = 1/3, v/v) afforded **5c** as yellow oil (39.6 mg, 51% yield). ^{1}H NMR (400 MHz, $CDCl_3$): δ = 1.23 (t, J = 7.2 Hz, 3H), 2.29 (s, 3H), 3.19 (d, J = 6.0 Hz, 2H), 4.18 (q, J = 7.2 Hz, 2H), 4.99-5.04 (m, 1H), 6.98-7.05 (m, 3H), 7.16 (t, J = 7.6 Hz, 1H), 7.41 (ddd, J = 7.6 Hz, 4.8 Hz, 1.2 Hz, 1H), 7.83 (td, J = 7.6 Hz, 1.6 Hz, 1H), 8.16 (d, J = 7.6 Hz, 1H), 8.50 (d, J = 8.0 Hz, 1H), 8.54-8.56 (m, 1H) ppm. ^{13}C NMR (100 MHz, $CDCl_3$): δ = 14.2, 21.4, 38.4, 53.6, 61.5, 122.3, 126.4, 126.5, 127.9, 128.5, 130.3, 136.1, 137.3, 138.1, 148.4, 149.5, 164.1, 171.5 ppm. HRMS (ESI$^+$): calcd for $C_{18}H_{20}N_2NaO_3$

[M+Na]$^+$ 335.1372, found 335.1375.

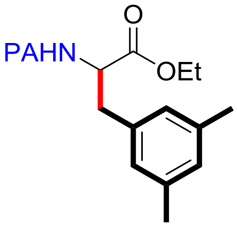

Ethyl 3-(3,5-dimethylphenyl)-2-(picolinamido)propanoate (5d)

Purification via column chromatography on silica gel (ethyl acetate/petroleum ether = 1/3, v/v) afforded **5d** as yellow oil (43.5 mg, 53% yield). ^{1}H NMR (400 MHz, $CDCl_3$): δ = 1.23 (t, J = 7.2 Hz, 3H), 2.25 (s, 6H), 3.15 (d, J = 6.4 Hz, 2H), 4.18 (q, J = 7.2 Hz, 2H), 4.97-5.02 (m, 1H), 6.82 (s, 2H), 6.87 (s, 1H), 7.42 (ddd, J = 7.6 Hz, 4.8 Hz, 1.2 Hz, 1H), 7.83 (td, J = 7.6 Hz, 1.6 Hz, 1H), 8.16 (d, J = 7.6 Hz, 1H), 8.49 (d, J = 8.4 Hz, 1H), 8.55 (d, J = 4.0 Hz, 1H) ppm. ^{13}C NMR (100 MHz, $CDCl_3$): δ = 14.3, 21.3, 38.4, 53.6, 61.5, 122.3, 126.4, 127.3, 128.8, 135.9, 137.3, 138.0, 148.4, 149.6, 164.1, 171.6 ppm. HRMS (ESI$^+$): calcd for $C_{19}H_{22}N_2NaO_3$ [M+Na]$^+$ 349.1528, found 349.1523.

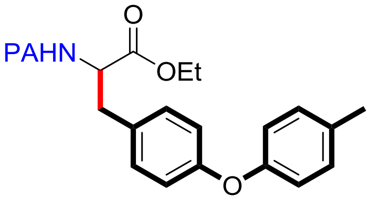

Ethyl 2-(picolinamido)-3-(4-(*p*-tolyloxy)phenyl)propanoate (5e)

Purification via column chromatography on silica gel (ethyl acetate/petroleum ether = 1/3, v/v) afforded **5e** as yellow oil (54.1 mg, 54% yield). ^{1}H NMR (400 MHz, $CDCl_3$): δ = 1.25 (t, J = 7.2 Hz, 3H), 2.32 (s, 3H), 3.17-3.26 (m, 2H), 4.19 (q, J = 7.2 Hz, 2H), 5.00-5.05 (m, 1H), 6.88-6.90 (m, 4H), 7.11-7.14 (m, 4H), 7.42 (dd, J = 7.6 Hz, 4.8 Hz, 1H), 7.83 (td, J = 7.6 Hz, 1.2 Hz, 1H), 8.16 (d, J = 7.6 Hz, 1H), 8.52 (d, J = 8.0 Hz, 1H), 8.56 (d, J = 4.8 Hz, 1H) ppm. ^{13}C NMR (100 MHz, $CDCl_3$): δ = 14.3, 20.8, 37.7, 53.7, 61.6, 118.4, 119.2, 122.3, 126.5, 130.3, 130.6, 130.7, 133.0, 137.4, 148.4, 149.5, 154.8, 157.0, 164.1, 171.4 ppm. HRMS (ESI$^+$): calcd for $C_{24}H_{25}N_2O_4$ [M+H]$^+$ 405.1814, found 405.1812.

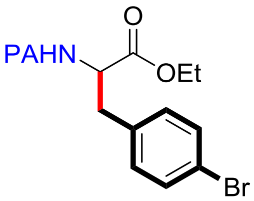

Ethyl 3-(4-bromophenyl)-2-(picolinamido)propanoate (5f)

Purification via column chromatography on silica gel (ethyl acetate/petroleum ether = 1/3, v/v) afforded **5f** as yellow oil (40.1 mg, 42% yield). ^{1}H NMR (400 MHz, $CDCl_3$): δ = 1.24 (t, J = 7.2 Hz, 3H), 3.14-3.25 (m, 2H), 4.19 (q, J = 7.2 Hz, 2H), 5.00-5.05 (m, 1H), 7.06 (d, J = 8.0 Hz, 2H), 7.39 (d, J = 8.4 Hz, 2H), 7.43 (dd, J = 7.6 Hz, 4.8 Hz, 1H), 7.84 (td, J = 8.0 Hz, 1.2 Hz, 1H), 8.15 (dd, J = 8.0 Hz, 0.8 Hz, 1H), 8.50 (d, J = 8.0 Hz, 1H), 8.56 (dt, J = 4.8 Hz, 0.8 Hz, 1H) ppm. ^{13}C NMR (100 MHz, $CDCl_3$): δ = 14.3, 37.9, 53.3, 61.8, 121.2, 122.4, 126.6, 131.2, 131.7, 135.3, 137.4, 148.5, 149.3, 164.1, 171.2 ppm. HRMS (ESI$^+$): calcd for $C_{17}H_{17}BrN_2NaO_3$ $[M+Na]^+$ 399.0320, found 399.0326.

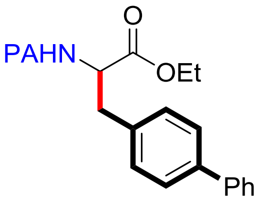

Ethyl 3-(biphenyl-4-yl)-2-(picolinamido)propanoate (5g)

Purification via column chromatography on silica gel (ethyl acetate/petroleum ether = 1/4, v/v) afforded **5g** as yellow oil (35.7 mg, 38% yield). ^{1}H NMR (400 MHz, $CDCl_3$): δ = 1.25 (t, J = 7.2 Hz, 3H), 3.29 (dd, J = 6.0 Hz, 1.6 Hz, 2H), 4.21 (q, J = 7.2 Hz, 2H), 5.06-5.11 (m, 1H), 7.27-7.29 (m, 2H), 7.31-7.35 (m, 1H), 7.40-7.44 (m, 3H), 7.51-7.53 (m, 2H), 7.56-7.58 (m, 2H), 7.84 (td, J = 7.6 Hz, 1.6 Hz, 1H), 8.18 (d, J = 7.6 Hz, 1H), 8.54-8.57 (m, 2H) ppm. ^{13}C NMR (100 MHz, $CDCl_3$): δ = 14.3, 38.2, 53.6, 61.6, 122.4, 126.5, 127.1, 127.3, 128.9, 129.9, 135.3, 137.4, 140.0, 140.9, 148.5, 149.5, 164.1, 171.5 ppm. HRMS (ESI$^+$): calcd for $C_{23}H_{22}N_2NaO_3$ $[M+Na]^+$ 397.1528, found 397.1532.

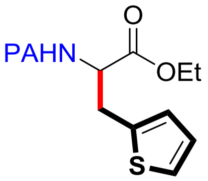

Ethyl 2-(picolinamido)-3-(thiophen-2-yl)propanoate (5h)

Purification via column chromatography on silica gel (ethyl acetate/petroleum ether = 1/3, v/v) afforded **5h** as yellow oil (31.0 mg, 42% yield). ^{1}H NMR (400 MHz, $CDCl_3$): δ = 1.28 (t, J = 7.2 Hz, 3H), 3.50 (d, J = 5.2 Hz, 2H), 4.23 (q, J = 7.2 Hz, 2H), 5.03-5.08 (m, 1H), 6.84 (d, J = 2.8 Hz, 1H), 6.92 (dd, J = 5.2 Hz, 3.6 Hz, 1H), 7.15 (dd, J = 5.2 Hz, 0.8 Hz, 1H), 7.43 (ddd, J = 7.6 Hz, 4.8 Hz, 1.2 Hz, 1H), 7.84 (td, J = 7.6 Hz, 1.6 Hz, 1H), 8.18 (d, J = 7.6 Hz, 1H), 8.58 (d, J = 4.4 Hz, 1H), 8.64 (d, J = 8.0 Hz, 1H) ppm. ^{13}C NMR (100 MHz, $CDCl_3$): δ = 14.3, 32.5, 53.4, 61.9, 122.4, 124.9, 126.5, 126.9, 127.1, 137.4, 137.6, 148.5, 149.4, 164.2, 170.9 ppm. HRMS (ESI^+): calcd for $C_{15}H_{16}N_2NaO_3S$ $[M+Na]^+$ 327.0779, found 327.0776.

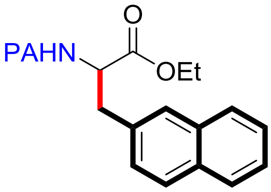

Ethyl 3-(naphthalen-2-yl)-2-(picolinamido)propanoate (5i)

Purification via column chromatography on silica gel (ethyl acetate/petroleum ether = 1/3, v/v) afforded **5i** as yellow oil (56.0 mg, 64% yield). ^{1}H NMR (400 MHz, $CDCl_3$): δ = 1.20 (t, J = 7.2 Hz, 3H), 3.41 (d, J = 6.4 Hz, 2H), 4.18 (q, J = 7.2 Hz, 2H), 5.12-5.18 (m, 1H), 7.35 (dd, J = 8.4 Hz, 1.6 Hz, 1H), 7.39-7.47 (m, 3H), 7.66 (s, 1H), 7.74-7.84 (m, 4H), 8.16 (d, J = 7.6 Hz, 1H), 8.53 (d, J = 4.4 Hz, 1H), 8.56 (d, J = 8.0 Hz, 1H) ppm. ^{13}C NMR (100 MHz, $CDCl_3$): δ = 14.2, 38.7, 53.6, 61.6, 122.3, 125.8, 126.2, 126.5, 127.6, 127.70, 127.74, 128.2, 128.3, 132.6, 133.5, 133.8, 137.3, 148.4, 149.4, 164.1, 171.5 ppm. HRMS (ESI^+): calcd for $C_{21}H_{20}N_2NaO_3$ $[M+Na]^+$ 371.1372, found 371.1369.

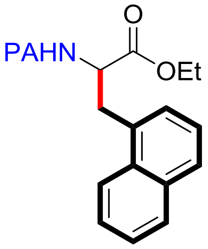

Ethyl 3-(naphthalen-1-yl)-2-(picolinamido)propanoate (5j)

Purification via column chromatography on silica gel (ethyl acetate/petroleum ether = 1/3, v/v) afforded **5j** as yellow oil (42.5 mg, 49% yield). ^{1}H NMR (400 MHz, $CDCl_3$): δ = 1.05 (t, J = 7.2 Hz, 3H), 3.63 (dd, J = 14.0 Hz, 7.6 Hz, 1H), 3.74 (dd, J = 14.0 Hz, 6.8 Hz, 1H), 3.99-4.12 (m, 2H), 5.17 (q, J = 7.6 Hz, 1H), 7.34-7.42 (m, 3H), 7.48 (t, J = 7.2 Hz, 1H), 7.52-7.56 (m, 1H), 7.76 (d, J = 7.2 Hz, 1H), 7.81 (dd, J = 7.6 Hz, 1.2 Hz, 1H), 7.85 (d, J = 8.0 Hz, 1H), 8.16 (d, J = 8.0 Hz, 1H), 8.24 (d, J = 8.4 Hz, 1H), 8.54 (d, J = 4.4 Hz, 1H), 8.62 (d, J = 7.6 Hz, 1H) ppm. ^{13}C NMR (100 MHz, $CDCl_3$): δ = 14.0, 36.2, 53.5, 61.5, 122.3, 123.8, 125.4, 125.8, 126.4, 127.6, 128.0, 128.9, 132.2, 132.6, 133.9, 137.4, 148.4, 149.4, 164.2, 171.8 ppm. HRMS (ESI^{+}): calcd for $C_{21}H_{20}N_2NaO_3$ $[M+Na]^{+}$ 371.1372, found 371.1379.

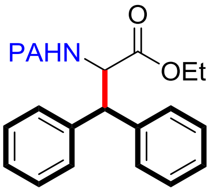

Ethyl 3,3-diphenyl-2-(picolinamido)propanoate (5k)

Purification via column chromatography on silica gel (ethyl acetate/petroleum ether = 1/4, v/v) afforded **5k** as yellow oil (44.8 mg, 48% yield). ^{1}H NMR (400 MHz, $CDCl_3$): δ = 0.96 (t, J = 7.2 Hz, 3H), 3.93-4.00 (m, 2H), 4.59 (d, J = 9.6 Hz, 1H), 5.58 (t, J = 9.2 Hz, 1H), 7.17-7.22 (m, 2H), 7.25-7.31 (m, 4H), 7.33-7.37 (m, 5H), 7.77 (td, J = 7.6 Hz, 1.6 Hz, 1H), 8.11 (d, J = 7.6 Hz, 1H), 8.44 (d, J = 9.6 Hz, 1H), 8.45-8.47 (m, 1H) ppm. ^{13}C NMR (100 MHz, $CDCl_3$): δ = 13.8, 54.1, 55.6, 61.3, 122.4, 126.4, 127.19, 127.23, 128.5, 128.59, 128.62, 128.8, 137.3, 139.8, 140.4, 148.3, 149.2, 164.2, 171.6 ppm. HRMS (ESI^{+}): calcd for $C_{23}H_{22}N_2NaO_3$ $[M+Na]^{+}$ 397.1528, found 397.1521.

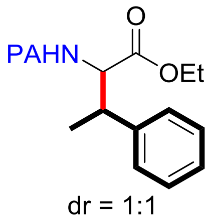

Ethyl 3-phenyl-2-(picolinamido)butanoate (5l)

The ratio of the two diastereoisomers is 1:1. Purification via column chromatography on silica gel (ethyl acetate/petroleum ether = 1/3, v/v) afforded **5l** as yellow oil (51.0 mg, 65% yield). ^{1}H NMR (400 MHz, $CDCl_3$) of the mixture: δ = 1.03 (t, J = 7.2 Hz, 3H), 1.23 (t, J = 7.2 Hz, 3H), 1.41 (d, J = 7.2 Hz, 3H), 1.45 (d, J = 7.2 Hz, 3H), 3.28-3.35 (m, 1H), 3.51-3.57 (m, 1H), 3.95-4.03 (m, 2H), 4.15 (q, J = 7.2 Hz, 2H), 4.96 (dd, J = 9.2 Hz, 7.6 Hz, 1H), 5.04 (dd, J = 9.2 Hz, 5.6 Hz, 1H), 7.20-7.34 (m, 10H), 7.38-7.44 (m, 2H), 7.79-7.84 (m, 2H), 8.13 (d, J = 3.2 Hz, 1H), 8.15 (d, J = 3.2 Hz, 1H), 8.41 (d, J = 9.2 Hz, 1H), 8.53-8.58 (m, 3H) ppm. ^{13}C NMR (100 MHz, $CDCl_3$) of the mixture: δ = 13.9, 14.2, 17.4, 17.7, 42.4, 43.3, 57.5, 58.1, 61.1, 61.4, 122.3, 122.4, 126.35, 126.43, 127.2, 127.3, 127.81, 127.82, 128.5, 128.6, 137.3, 137.4, 141.1, 141.6, 148.3, 148.4, 149.4, 164.1, 164.3, 171.2, 171.3 ppm. HRMS (ESI$^+$): calcd for $C_{18}H_{20}N_2NaO_3$ [M+Na]$^+$ 335.1372, found 335.1371.

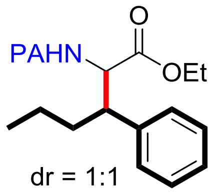

Ethyl 3-phenyl-2-(picolinamido)hexanoate (5m)

The ratio of the two diastereoisomers is 1:1. Purification via column chromatography on silica gel (ethyl acetate/petroleum ether = 1/4, v/v) afforded **5m** as yellow oil (60.8 mg, 71% yield). ^{1}H NMR (400 MHz, $CDCl_3$) of the mixture: δ = 0.84 (t, J = 7.2 Hz, 3H), 0.86 (t, J = 7.2 Hz, 3H), 0.99 (t, J = 7.2 Hz, 3H), 1.12-1.32 (m, 7H), 1.74-1.81 (m, 2H), 1.85 (q, J = 7.6 Hz, 2H), 3.09 (q, J = 7.6 Hz, 1H), 3.35-3.40 (m, 1H), 3.91-3.98 (m, 2H), 4.10-4.17 (m, 2H), 4.96 (dd, J = 9.2 Hz, 8.0 Hz, 1H), 5.12 (dd, J = 9.2 Hz, 4.8 Hz, 1H), 7.21-7.34 (m, 10H), 7.39-7.45 (m, 2H), 7.83 (qd, J = 7.6 Hz, 1.6

Hz, 2H), 8.14-8.18 (m, 2H), 8.40 (d, *J* = 9.2 Hz, 1H), 8.53-8.56 (m, 2H), 8.59 (d, *J* = 4.0 Hz, 1H) ppm. ^{13}C NMR (100 MHz, $CDCl_3$) of the mixture: δ = 13.9, 14.0, 14.1, 14.3, 20.6, 33.6, 33.9, 48.0, 49.3, 56.4, 57.5, 61.1, 61.4, 122.4, 122.5, 126.4, 126.5, 127.2, 127.4, 128.5, 128.6, 128.7, 137.3, 137.4, 139.5, 140.0, 148.4, 148.5, 149.50, 149.53, 164.1, 164.3, 171.3, 171.4 ppm. HRMS (ESI$^+$): calcd for $C_{20}H_{24}N_2NaO_3$ $[M+Na]^+$ 363.1685, found 363.1686.

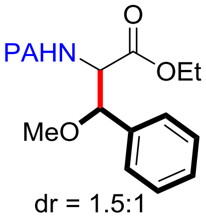

dr = 1.5:1

Ethyl 3-methoxy-3-phenyl-2-(picolinamido)propanoate (5n)

The ratio of the two diastereoisomers is 1.5:1. Purification via column chromatography on silica gel (ethyl acetate/petroleum ether = 1/3, v/v) afforded **5n** as yellow oil (60.5 mg, 73% yield). ^{1}H NMR (400 MHz, $CDCl_3$, a mixture of two isomers): δ = 1.17 (t, *J* = 7.2 Hz, $CO_2CH_2C\underline{H}_3$, minor isomer), 1.25 (t, *J* = 7.2 Hz, $CO_2CH_2C\underline{H}_3$, major isomer), 3.34 (s, $OC\underline{H}_3$, major isomer), 3.37 (s, $OC\underline{H}_3$, minor isomer), 4.14 (q, *J* = 7.2 Hz, $CO_2C\underline{H}_2CH_3$, minor isomer), 4.19-4.28 (m, $CO_2C\underline{H}_2CH_3$, major isomer), 4.73 (d, *J* = 5.2 Hz, MeOC$\underline{H}$Ph, minor isomer), 4.91 (d, *J* = 3.2 Hz, MeOC$\underline{H}$Ph, major isomer), 4.95 (dd, *J* = 9.2 Hz, 3.6 Hz, EtOOCC$\underline{H}$, major isomer), 5.16 (dd, *J* = 9.2 Hz, 4.8 Hz, MeOC$\underline{H}$Ph, minor isomer), 7.21-7.43 (m), 7.77 (td, *J* = 7.6 Hz, 1.6 Hz, major isomer), 7.81 (td, *J* = 7.6 Hz, 1.6 Hz, minor isomer), 8.02 (d, *J* = 8.0 Hz, major isomer), 8.14 (d, *J* = 8.0 Hz, minor isomer), 8.56-8.57 (m, minor isomer), 8.61-8.63 (m), 8.71 (d, *J* = 8.8 Hz, PAN$\underline{H}$, major isomer) ppm. ^{13}C NMR (100 MHz, $CDCl_3$) of the mixture: δ = 14.1, 14.3, 57.3, 57.7, 57.9, 58.3, 61.5, 61.7, 83.0, 83.8, 122.40, 122.42, 126.3, 126.4, 127.0, 127.1, 128.3, 128.4, 128.48, 128.53, 137.2, 137.29, 137.34, 148.40, 148.43, 149.49, 149.52, 164.1, 164.3, 169.7, 170.1 ppm. HRMS (ESI$^+$): calcd for $C_{18}H_{20}N_2NaO_4$ $[M+Na]^+$ 351.1321, found 351.1320.

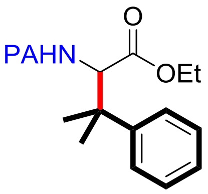

Ethyl 3-methyl-3-phenyl-2-(picolinamido)butanoate (5o)

Purification via column chromatography on silica gel (ethyl acetate/petroleum ether = 1/3, v/v) afforded **5o** as yellow oil (65.2 mg, 80% yield). ^{1}H NMR (400 MHz, $CDCl_3$): δ = 0.99 (t, J = 7.2 Hz, 3H), 1.50 (s, 3H), 1.52 (s, 3H), 3.90-3.98 (m, 2H), 5.05 (d, J = 9.6 Hz, 1H), 7.21-7.25 (m, 1H), 7.30-7.35 (m, 2H), 7.41 (ddd, J = 7.6 Hz, 4.8 Hz, 1.2 Hz, 1H), 7.43-7.46 (m, 2H), 7.82 (td, J = 7.6 Hz, 1.6 Hz, 1H), 8.15 (dt, J = 8.0 Hz, 1.2 Hz, 1H), 8.56-8.61 (m, 2H) ppm. ^{13}C NMR (100 MHz, $CDCl_3$): δ = 13.9, 24.3, 26.4, 42.1, 60.8, 60.9, 122.4, 126.4, 126.5, 126.7, 128.3, 137.3, 145.1, 148.4, 149.6, 164.2, 170.8 ppm. HRMS (ESI$^+$): calcd for $C_{19}H_{23}N_2O_3$ $[M+H]^+$ 327.1709, found 327.1718.

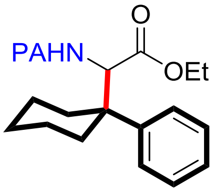

Ethyl 2-(1-phenylcyclohexyl)-2-(picolinamido)acetate (5p)

Purification via column chromatography on silica gel (ethyl acetate/petroleum ether = 1/4, v/v) afforded **5p** as pale yellow oil (54.8 mg, 60% yield). ^{1}H NMR (400 MHz, $CDCl_3$): δ = 1.05 (t, J = 7.2 Hz, 3H), 1.25-1.30 (m, 3H), 1.51-1.63 (m, 3H), 1.70-1.89 (m, 2H), 2.37-2.52 (m, 2H), 3.87-4.00 (m, 2H), 4.91 (d, J = 10.0 Hz, 1H), 7.25-7.29 (m, 1H), 7.37-7.38 (m, 4H), 7.42 (ddd, J = 7.6 Hz, 4.8 Hz, 1.2 Hz, 1H), 7.83 (td, J = 7.6 Hz, 1.6 Hz, 1H), 8.17 (dt, J = 8.0 Hz, 1.2 Hz, 1H), 8.48 (d, J = 10.4 Hz, 1H), 8.58 (dq, J = 4.8 Hz, 0.8 Hz, 1H) ppm. ^{13}C NMR (100 MHz, $CDCl_3$): δ = 14.0, 22.0, 22.1, 26.3, 32.1, 33.6, 46.3, 60.9, 61.4, 122.5, 126.4, 126.7, 127.9, 128.6, 137.3, 140.5, 148.5, 149.6, 164.1, 170.3 ppm. HRMS (ESI$^+$): calcd for $C_{22}H_{26}N_2NaO_3$ $[M+Na]^+$ 389.1841, found 389.1844.

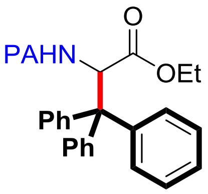

Ethyl 3,3,3-triphenyl-2-(picolinamido)propanoate (5q)

Purification via column chromatography on silica gel (ethyl acetate/petroleum ether = 1/4, v/v) afforded **5q** as yellow oil (87.3 mg, 77% yield). ^{1}H NMR (400 MHz, $CDCl_3$): δ = 0.89 (t, J = 7.2 Hz, 3H), 3.79-3.91 (m, 2H), 6.54 (d, J = 10.4 Hz, 1H), 7.18-7.21 (m, 3H), 7.26-7.32 (m, 7H), 7.46-7.48 (m, 6H), 7.76 (t, J = 8.0 Hz, 1H), 8.15 (d, J = 7.6 Hz, 1H), 8.38-8.40 (m, 2H) ppm. ^{13}C NMR (100 MHz, $CDCl_3$): δ = 13.7, 56.6, 61.3, 61.8, 122.3, 126.3, 126.7, 127.9, 129.8, 137.2, 143.6, 148.4, 149.3, 164.3, 170.2 ppm. HRMS (ESI$^+$): calcd for $C_{29}H_{26}N_2NaO_3$ $[M+Na]^+$ 473.1841, found 473.1841.

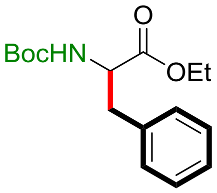

Ethyl 2-(*tert*-butoxycarbonylamino)-3-phenylpropanoate (6a)

^{1}H NMR (400 MHz, $CDCl_3$): δ = 1.22 (t, J = 7.2 Hz, 3H), 1.41 (s, 9H), 3.02-3.13 (m, 2H), 4.15 (q, J = 7.2 Hz, 2H), 4.53-4.58 (m, 1H), 4.99 (d, J = 6.8 Hz, 1H), 7.13-7.14 (m, 2H), 7.21-7.30 (m, 3H) ppm. ^{13}C NMR (100 MHz, $CDCl_3$): δ = 14.2, 28.4, 38.5, 54.6, 61.4, 80.0, 127.1, 128.6, 129.5, 136.2, 155.2, 172.0 ppm. HRMS (ESI$^+$): calcd for $C_{16}H_{23}NNaO_4$ $[M+Na]^+$ 316.1525, found 316.1520.

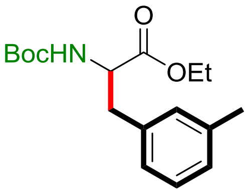

Ethyl 2-(*tert*-butoxycarbonylamino)-3-*m*-tolylpropanoate (6b)

^{1}H NMR (400 MHz, $CDCl_3$): δ = 1.23 (t, J = 7.2 Hz, 3H), 1.42 (s, 9H), 2.32 (s, 3H), 2.99-3.09 (m, 2H), 4.16 (q, J = 7.2 Hz, 2H), 4.51-4.56 (m, 1H), 4.97 (d, J = 7.2 Hz, 1H), 6.92-6.95 (m, 2H), 7.05 (d, J = 7.6 Hz, 1H), 7.17 (t, J = 7.6 Hz, 1H) ppm. ^{13}C

NMR (100 MHz, $CDCl_3$): δ = 14.3, 21.5, 28.4, 38.4, 54.6, 61.4, 79.9, 126.5, 127.8, 128.5, 130.3, 136.1, 138.2, 155.3, 172.1 ppm. HRMS (ESI$^+$): calcd for $C_{17}H_{25}NNaO_4$ $[M+Na]^+$ 330.1681, found 330.1680.

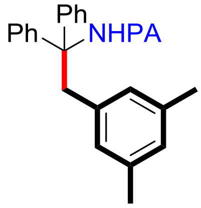

***N*-(2-(3,5-dimethylphenyl)-1,1-diphenylethyl)picolinamide (7)**

Purification via column chromatography on silica gel (ethyl acetate/petroleum ether = 1/6, v/v) afforded **7** as yellow oil (38.0 mg, 37% yield). ^1H NMR (400 MHz, $CDCl_3$): δ = 2.03 (s, 6H), 3.95 (s, 2H), 6.26 (s, 2H), 6.75 (s, 1H), 7.24-7.28 (m, 2H), 7.31-7.34 (m, 8H), 7.42 (dd, *J* = 7.6 Hz, 4.8 Hz, 1H), 7.83 (td, *J* = 7.6 Hz, 1.2 Hz, 1H), 8.16 (d, *J* = 8.0 Hz, 1H), 8.54 (d, *J* = 4.8 Hz, 1H), 9.16 (s, 1H) ppm. ^{13}C NMR (100 MHz, $CDCl_3$): δ = 21.1, 43.9, 65.1, 122.0, 126.1, 127.0, 127.4, 127.9, 128.0, 129.1, 136.3, 136.8, 137.4, 145.1, 148.1, 150.7, 163.5 ppm. HRMS (ESI$^+$): calcd for $C_{28}H_{26}N_2NaO$ $[M+Na]^+$ 429.1943, found 429.1941.

Supplementary References

1. Li, K., Tan, G., Huang, J., Song, F. & You, J. Iron-catalyzed oxidative C–H/C–H cross-coupling: An efficient route to α-quaternary α-amino acid derivatives. *Angew. Chem. Int. Ed.* **52**, 12942–12945 (2013).

2. Dungan,V. J., Wong, S. M., Barry, S. M. & Rutledge, P. J. L-Proline-derived ligands to mimic the ‘2-His-1-carboxylate’ triad of the non-haem iron oxidase active site. *Tetrahedron* **68**, 3231–3236 (2012).
